# The activity of indenylidene derivatives in olefin metathesis catalysts

**DOI:** 10.3762/bjoc.14.275

**Published:** 2018-11-30

**Authors:** Maria Voccia, Steven P Nolan, Luigi Cavallo, Albert Poater

**Affiliations:** 1 Institut de Química Computacional i Catàlisi and Departament de Química, Universitat de Girona, c/ Mª Aurèlia Capmany 69, 17003 Girona, Catalonia, Spain; 2 Department of Chemistry and Center for Sustainable Chemistry, Ghent University, Krijgslaan 281, S-3, B-9000 Ghent, Belgium; 3 Department of Chemistry, College of Science King Saud University, P. O. Box 2455, Riyadh 11451, Saudi Arabia; 4 King Abdullah University of Science & Technology, KAUST Catalysis Center (KCC), 23955-6900 Thuwal, Saudi Arabia

**Keywords:** activation, IMes, indenylidene, olefin metathesis, SIMes

## Abstract

The first turnover event of an olefin metathesis reaction using a new family of homogenous Ru-based catalysts bearing modified indenylidene ligands has been investigated, using methoxyethylene as a substrate. The study is carried out by means of density functional theory (DFT). The indenylidene ligands are decorated with *ortho*-methyl and isopropyl groups at both *ortho* positions of their phenyl ring. DFT results highlight the more sterically demanding indenylidenes have to undergo a more exothermic first phosphine dissociation step. Overall, the study emphasises advantages of increased steric hindrance in promoting the phosphine release, and the relative stability of the corresponding metallacycle over classical ylidene ligands. Mayer bond orders and steric maps provide structural reasons for these effects, whereas NICS aromaticity and conceptual DFT confirm that the electronic parameters do not play a significant role.

## Introduction

Olefin metathesis has been an intensely studied reaction due to its wide use [1], in industrial applications, especially in petrochemistry [2], i.e., the Phillips Triolefin (PTP) process or the Shell Higher Olefin Process (SHOP) [3–4]. Additionally, the olefin metathesis reaction has provided a useful tool in polymerisation [5–6], as well as in the pharmaceutical industry in the formation of C=C bonds. Early catalyst examples were ill-defined entities and it is not until Grubbs [7] and Schrock [8] developed well-defined homogeneous catalysts that the area truly blossomed. Using a metal carbene complex as a catalyst, making use of the Chauvin mechanism, olefin metathesis consists of the redistribution of two carbon–carbon double bonds [9]. The metal and its ligand environment in both ruthenium and molybdenum systems appear to confer the right environment that allows a productive alkene metathesis [10–11]. Little productive reactivity has been uncovered using other metals [12–14]. Apart from the metal, ruthenium-based olefin metathesis has seen several changes during the last decades, modifying the existing commercial catalysts, playing mainly with the electronic characteristics of the ligands (usually two chlorides and an ylidene ligand) [15–17], whereas basically the sterics of the substituents on the N-heterocyclic carbene (NHC) ligand have remained unchanged [18]. Overall, any modification of the available catalysts has been performed in order to increase the stability of the catalyst without losing any of its activity [19–20]. Although most of the olefin metathesis catalysts are based on ruthenium [21–22], because these are more stable to oxygen and moisture [23] than their molybdenum counterparts, they display sensitivity to decomposition while in solution [24–25]. Understanding and/or the elimination of potential pathways that leads to catalyst decomposition is extremely important [26–28], since any knowledge obtained in this area can guide the catalyst design efforts [29–31].

**Scheme 1:**
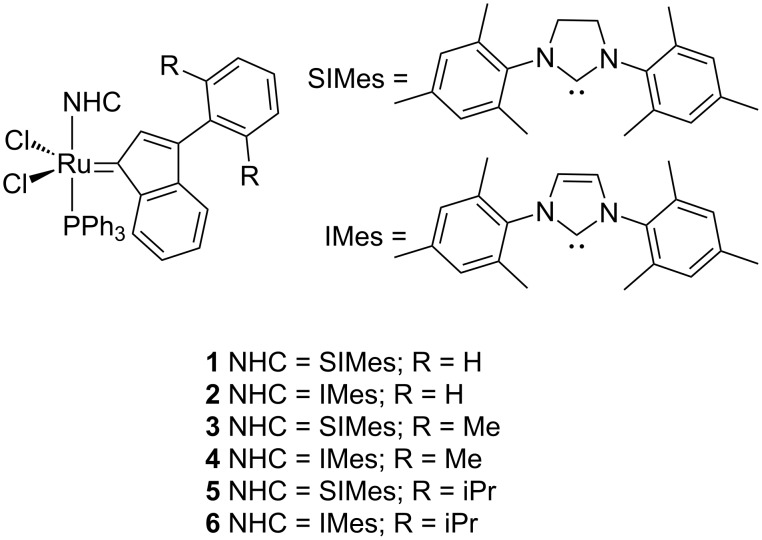
Catalysts studied by DFT calculations.

We are interested in evaluating, by density functional theory (DFT) calculations, the difference in the activation step between complexes **1**–**6** in Scheme 1, whose reactivity and properties have been reported already (for **1** and **2**) [24,32–33]. Predictive catalysis will be used here to generate and/or describe the activity in olefin metathesis of the new indenylidene derivatives. The phenyl substituent of the indenylidene is perpendicular to the indenyl moiety in the solid-state structure [34], as Nolan and co-workers first described in 1999 [35]. For complexes **3**–**6**, where the phenyl ring is *ortho-*substituted, there might be present steric repulsion with the NHCs, which might in turn facilitate the departure of the indenyl ligand [36]. Apart from reducing decomposition [37–38], this steric pressure should lead to faster rates for the initiation step of the metathesis reaction. This hypothesis will be examined computationally in order to assist catalyst design efforts.

## Results and Discussion

**Scheme 2:**
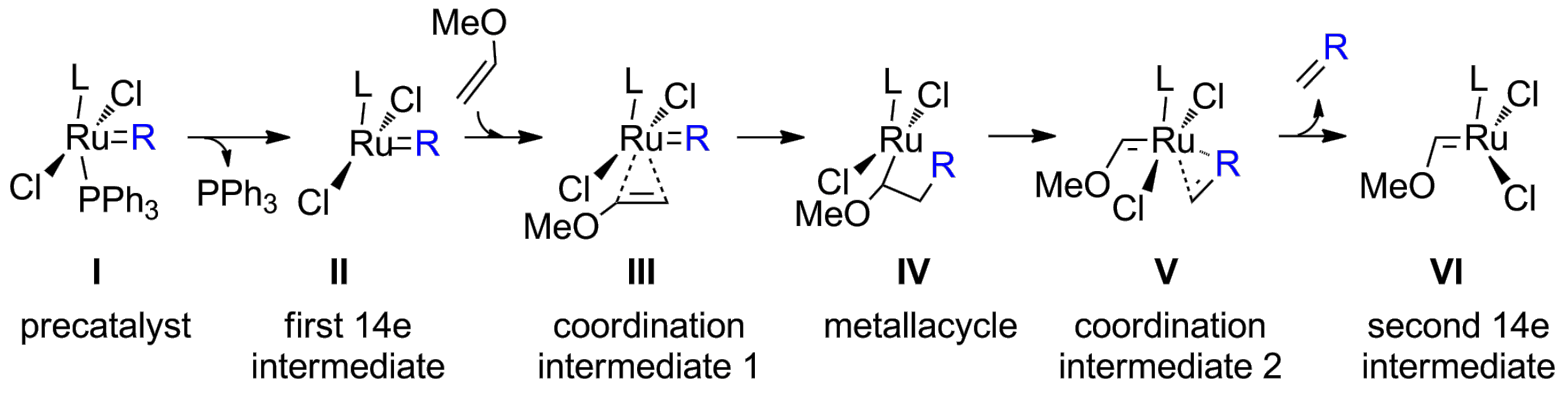
Precatalyst initiation in olefin metathesis (L = NHC ligand).

We have studied the initiation cycle involving the transformation of the indenylidene precatalysts into the active methylidene for a series of olefin metathesis relevant complexes **1**–**6**, using methoxyethene as a substrate (Scheme 2). This substrate was selected in order to facilitate our analysis [39]. Computationally no significant differences exist by using ethene or methoxyethene [40–41]. The saturation of the backbone of the NHC has also been taken into account, thus considering either the SIMes (1,3-bis(2,4,6-trimethylphenyl)-4,5-dihydroimidazol-2-ylidene) and the IMes (1,3-bis(2,4,6-trimethylphenyl)imidazol-2-ylidene) NHC ligands. The group *trans* to the NHC ligand is triphenylphosphine for all catalysts.

Table 1 includes the energy profiles for the substituted indenylidenes, bearing methyl or isopropyl groups at the *ortho* positions of the phenyl substituent, compared to the unsubstituted **1** and **2**. Comparing IMes vs SIMes, the activation is about 1 kcal/mol more favoured for the unsaturated system [42–43]. The absolute difference of 1 kcal/mol is maintained throughout the mechanism, however, we must point out that the opening of the metallacycle requires only 0.6 kcal/mol for the SIMes system versus 1.7 kcal/mol for the unsaturated system.

**Table 1:** Precatalyst initiation reaction pathway for catalysts **1**–**6** (M06/TZVP
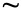
sdd//BP86/SVP
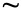
sdd; Gibbs free energies in kcal/mol).

	**1**	**2**	**3**	**4**	**5**	**6**

**I**	0.0	0.0	0.0	0.0	0.0	0.0
**I**–**II**	21.8	20.2	16.4	19.1^a^	19.8	19.8
**II**	20.0	18.2	16.1	19.5	17.6	19.6
**II**–**III**	22.8	21.8	20.0	22.4	21.0	22.1
**III**	15.0	12.2	13.0	14.6	17.2	17.8
**III**–**IV**	22.1	21.1	19.0	20.1	17.8	19.3
**IV**	16.7	15.9	12.5	15.1	13.3	13.0
**IV**–**V**	17.3	17.6	13.7	15.8	15.6	16.2
**V**	7.0	3.7	2.0	3.5	2.9	2.9
**V**–**VI**	10.5	10.2	8.6	10.0	5.9	9.6
**VI**	8.1	7.0	3.8	5.4	4.3	5.0

^a^The transition state is somewhat lower in energy than the next 14e species **II** once included the solvent effects.

The methyl and isopropyl-substituted indenylidene moieties reveal a different performance between the SIMes and the IMes congeners. When the saturated NHC is considered, the substitution reduces dramatically the barriers of the first two transition states. Then the cycle **I**→**VI** is more exothermic with the substituted systems. On the other hand, the unsaturated system does not reduce the energy barriers with the substituted indenylidene moieties, because of the rigidity of its backbone. And the same argument is valid for the entire catalytic system, which means that the substitution does not help to make the system significantly more exothermic than the saturated system, despite a slight stabilisation, especially for the second 14e species, **VI**.

Overall, among the catalysts with substituted indenylidene catalysts, the one bearing a SIMes NHC ligand and methyl groups in the indenylidene moiety is the most promising, in agreement with the experimental hypothesis that the release of the indenylidene ligand is more facile in such a case.

The concerted transition state that circumvents the formation of the 14e intermediate **II**, i.e., **I**–**III**, is higher in energy than **I**–**II** and **II**–**III** by 3.6 and 4.3 kcal/mol for systems **3** and **5**, compared to **1**, respectively. The unsaturated systems do not follow a concerted mechanism either, being 4.3 kcal/mol higher in energy for system **2**. Overall, for all substituted indenylidenes this concerted transition state **I**–**III** is confirmed to be higher in energy.

The structural analysis included in Table 2 supports the fact that the substituted indenylidenes display similar characteristics whatever the substituents are on both *ortho* positions of the corresponding phenyl ring in catalysts **1**–**6**. For instance, the Ru=C_ylidene_ bond changes by less than 0.008 Å, which is in perfect agreement with the insignificant changes in the Mayer Bond Order (MBO) results [44–45]. However, there is a clear difference that shows that the saturated NHC backbone of the SIMes ligand facilitates the phosphine dissociation since the Ru–P bond distance is longer [24,38]. This is corroborated via the corresponding lower MBO values. More interestingly, the Ru–C_NHC_ bond is much shorter for SIMes, with a corresponding MBOs at least 0.030 larger, being more accentuated for the system with the unsubstituted indenylidene ligand, by 0.041. This effect is completely in agreement with the *trans* effect along the C_NHC_–Ru–P axis [46]. On the other hand, the analysis of the substituted indenylidene systems **3**–**6** highlights that these are more difficult to activate since the MBO of the Ru–P is larger by 0.045, 0.042, 0.060 and 0.051, respectively. Overall, despite the unfavourable effect on the activation of the precatalytic species **I**, the substituted indenylidenes do not affect at all the 14e species **II** once generated through phosphine release.

**Table 2:** Structural analysis for species **I**–**III** for catalysts **1**–**6** (in kcal/mol), including selected bond distances (d) in Å and Mayer Bond Orders (MBO).

	**1**	**2**	**3**	**4**	**5**	**6**

d(Ru=C_ylidene_)						
**I**	1.882	1.882	1.883	1.883	1.886	1.885
**II**	1.869	1.868	1.863	1.862	1.863	1.861
**III**	1.888	1.885	1.885	1.883	1.885	1.884
MBO(Ru=C_ylidene_)						
**I**	1.464	1.469	1.476	1.484	1.475	1.480
**II**	1.444	1.446	1.467	1.470	1.465	1.471
**III**	1.445	1.453	1.453	1.460	1.448	1.452
d(Ru–P)						
**I**	2.457	2.444	2.436	2.430	2.431	2.424
MBO(Ru–P)						
**I**	0.540	0.560	0.585	0.592	0.600	0.611
d(Ru–C_NHC_)						
**I**	2.071	2.089	2.087	2.103	2.093	2.111
**II**	1.947	1.956	1.941	1.955	1.941	1.953
**III**	2.020	1.885	2.019	1.883	2.014	2.029
MBO(Ru–C_NHC_)						
**I**	0.847	0.806	0.817	0.787	0.808	0.775
**II**	1.199	1.151	1.211	1.160	1.212	1.162
**III**	0.961	0.915	0.969	0.924	0.983	0.933

**Figure 1:**
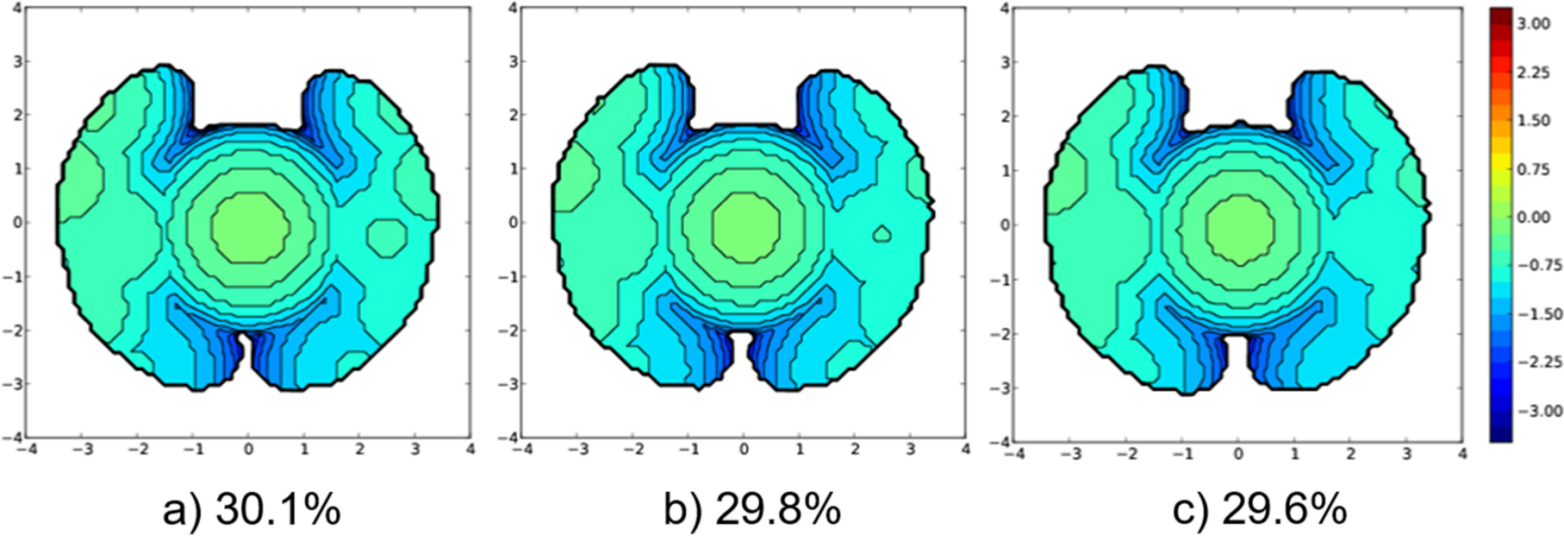
Topographic steric maps (plane xy) of the NHC ligands of species **I** for the studied SIMes–Ru complexes **1**, **3** and **5**, with a radius 3.5 Å. %V_Bur_ is the percent of buried volume. The Ru atom is at the origin and the Ru–C_NHC_ bond is aligned with the *z*-axis, and the Ru–C_ylidene_ with the *x*-axis. The isocontour curves of the steric maps are given in Å.

To further understand how substituent sterics affect the indenylidene moieties of catalysts **3**–**6**, steric maps were calculated by means of the SambVca package [47], analyzing the %V_Bur_ [48]. Taking into account the precatalytic intermediate **I**, the %V_Bur_ for the NHC ligand ranges from 30.1% to 29.8% and 29.6% for SIMes-based catalysts **1**, **3** and **5**, respectively (see Figure 1). The same trend applies to the IMes-based catalysts **2**, **4** and **6**, with %V_Bur_ values of 30.0%, 29.7% and 29.6%, respectively. On the other hand, as expected since there is similarity in the part bonded to the metal [49], no significant difference in the steric hindrance towards the metal sphere was observed for the indenylidene and any of its derivatives, and the %V_Bur_ was found identical (see Supporting Information File 1 for steric maps of those ligands).

Table 3 includes the relevant information obtained from the frontier molecular orbitals. From conceptual DFT we reach values for electrophilicity that describe a clear trend from species **II** that is prone to undergo nucleophilic attack by the entering olefin: the substituted indenylidene catalysts **3**–**6** exhibit lower electrophilicity, especially when bearing the saturated backbone NHC, systems **3** and **5**. Here the SIMes systems with lower electrophilicity are in perfect harmony with higher chemical hardness values [50], and the natural population analysis (NPA) on the C_ylidene_ confirms the more positive charge, which might favour the nucleophilic interaction with an olefin.

**Table 3:** Electronic analysis for species **I**–**III** for catalysts **1**–**6** (in kcal/mol) including energies of the frontier molecular orbitals (HOMO and LUMO); conceptual DFT parameters such as chemical hardness and electrophilicity; and natural population analysis (NPA) charges on ruthenium and ylidenic carbon.

	**1**	**2**	**3**	**4**	**5**	**6**

HOMO						
**I**	−0.142	−0.143	−0.146	−0.145	−0.147	−0.147
**II**	−0.168	−0.162	−0.169	−0.162	−0.168	−0.161
**III**	−0.149	−0.150	−0.152	−0.152	−0.152	−0.152
LUMO						
**I**	−0.121	−0.121	−0.119	−0.119	−0.118	−0.119
**II**	−0.127	−0.127	−0.125	−0.125	−0.125	−0.125
**III**	−0.122	−0.123	−0.120	−0.121	−0.119	−0.120
chemical hardness						
**I**	0.011	0.011	0.014	0.013	0.014	0.014
**II**	0.021	0.018	0.022	0.019	0.022	0.018
**III**	0.014	0.013	0.016	0.015	0.016	0.016
electrophilicity						
**I**	0.788	0.784	0.642	0.663	0.611	0.629
**II**	0.526	0.593	0.491	0.545	0.499	0.557
**III**	0.676	0.694	0.588	0.602	0.560	0.575
q(Ru)						
**I**	−0.414	−0.434	−0.414	−0.430	−0.416	−0.431
**II**	−0.101	−0.100	−0.099	−0.110	−0.119	−0.121
**III**	−0.259	−0.265	−0.256	−0.263	−0.260	−0.258
q(C_ylidene_)						
**I**	0.126	0.126	0.127	0.129	0.125	0.127
**II**	0.098	0.094	0.101	0.104	0.110	0.109
**III**	0.130	0.134	0.136	0.139	0.139	0.139

**Figure 2:**
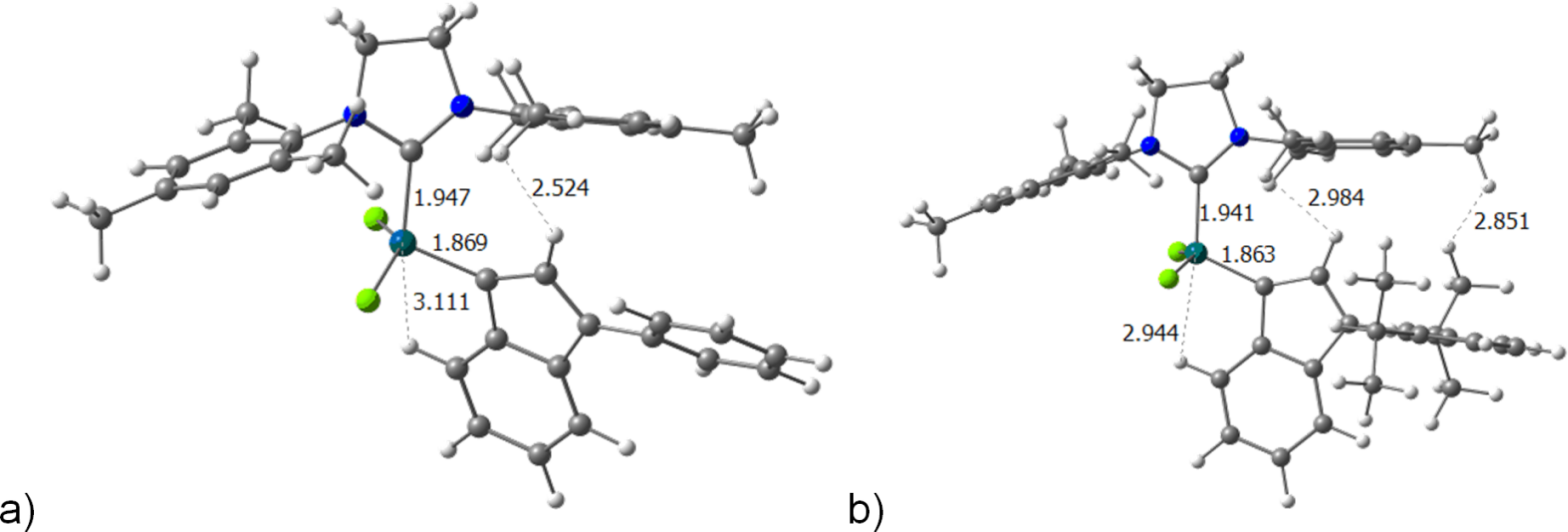
Intermediate **II** for catalysts a) **1** and b) **5** (important bond lengths are given in Å).

The NPA charges on ruthenium are not affected by the increase of steric hindrance on the phenyl rings of the indenylidene ligand, and only the IMes ligand shows a small decrease of the charge on the metal centre, on the precatalytic species **I**. Whereas **II** must be excluded from the discussion since there is a hydrogen bond (Ru···H) that affects the charge on the metal, especially strong when the indenylidene is substituted. The Ru···H distances for **1**–**6** are 3.110, 3.167, 3.004, 3.036 Å, 2.944, 2.929 Å, respectively (see Figure 2). This interaction is due to the rotation of 90° of the indenylidene ligand. But this Ru···H interaction deserves more attention since it is stronger for **3**–**6** and this in order to reduce the steric repulsion between the substituted phenyl ring of the indenylidene and the close mesityl group of the NHC ligand. One consequence for the more rigid unsaturated IMes systems **4** and **6** is that the next energy barrier for transition state **II**–**III** is larger, since the entering olefin requires a 90° rotation, and this is partially impeded when the phenyl group is substituted. However, the substituted indenylidene facilitates overcoming the energy barrier of the next transition state, the closure of the metallacycle in order to reduce the steric hindrance. Particularly, the latter transition state is 3.2 kcal/mol lower in energy for **5**, whereas only 0.7 kcal/mol more stable for **1**. And thermodynamically, the next metallacycle **IV** is also favoured with the indenylidene substitution, being 4.2 and 3.4 kcal/mol relatively more stable for systems **3** and **5**, respectively, with respect to **1**. This effect of reducing steric hindrance between the ylidene and the NHC ligands was examined previously, with the exchange of benzylidene by indenylidene [51]; and with larger NHC ligands [52].

In order to evaluate the change of the aromaticity of the phenyl ring of the indenylidene derivatives **3**–**6**, we evaluated the aromaticity of the six-membered aryl rings, as well as the ring current on the five-membered ring by means of magnetic-based aromaticity criterion NICS (GIAO, see all NICS values in Supporting Information File 1). The 5-membered rings turn out to be non-aromatic, whereas the changes of the aromaticity of the phenyl rings are minimal. Species **I**, **2** and **3** are 0.151 and 0.083 ppm more aromatic than **1**, nearly identical for the IMes systems **5** and **6**, 0.166 and 1.117 ppm and more aromatic than **4**. Once the phosphine has been dissociated, intermediate **II** becomes roughly 0.5 ppm more aromatic, but the trend for catalysts **1**–**6** is rather similar, being that **3**–**6** are approximately 0.1 ppm more aromatic than the non-substituted indenylidene systems **1** and **2**.

## Conclusion

We have investigated the reaction pathway of a series of Ru-based olefin metathesis catalysts that leads to the methylidene catalytic active species, i.e., the activation of the precatalyst. The indenylidene ligand is substituted by congener ligands with *ortho-*methyl or isopropyl group on its phenyl ring. It was shown that to describe the reactivity, structural, and electronic parameters must be taken into account. The indenylidene ligands, especially the more sterically demanding, impose higher electrophilicity on the ruthenium centre, but structurally favour the relative stability of the metallacycle in order to reduce the steric hindrance between the mesityl groups of either the SIMes or IMes ligands with the *ortho* substituents on the phenyl group of the indenylidene.

The steric hindrance of the indenylidene derivatives does not affect the metal centre structurally since the steric maps confirm that the effect is far removed from the metal core where the reactivity with the entering olefin takes place. On the other hand, electronically the effect is rather small, with insignificant changes of the aromaticity in the phenyl ring. However, the 14e species that will accommodate the entering olefin in the next reaction step imposes a stronger Ru···H interaction thanks to the substituents on the indenylidene pushing downward the indenylidene ligand itself, to minimise the steric hindrance with the NHC ligand.

## Computational Details

DFT static calculations were performed with the Gaussian 09 set of programs [53], using the BP86 functional of Becke and Perdew [54–56]. The electronic configuration of the molecular systems was described with the double-ζ basis set with polarisation of Ahlrichs for main group atoms (SVP keyword in Gaussian) [57], whereas for ruthenium the small-core quasi-relativistic Stuttgart/Dresden effective core potential, with an associated valence basis set (standard SDD keywords in Gaussian 09), was employed [58–60]. The geometry optimisations were performed without symmetry constraints. Analytical frequency calculations were performed to characterise the located stationary points, apart from calculating the unscaled zero-point energies (ZPEs) and the thermal corrections and entropy effects at 298 K, and all values at a pressure of 1354 atm using the approach of Martin and co-workers [61], excluding the potential overestimation of the entropy contribution [38,62–63]. Energies were obtained by single point calculations on the optimised geometries with the M06 functional [64] and the TZVP basis set [65], and solvent effects were estimated with the polarisable continuous solvation model PCM using dichloromethane as solvent [66–67]. The reported free energies in this work include energies obtained at the M06/TZVP
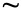
sdd level of theory corrected with zero-point energies, thermal corrections, and entropy effects evaluated at 298 K, achieved at the BP86/SVP
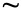
sdd level. This computational approach for olefin metathesis with Ru based catalysts turned out to display errors of less than 1 kcal/mol by Poater and co-workers [68].

The percent buried volume calculations were performed with the SambVca package developed by Cavallo et al. [42]. The radius of the sphere around the origin placed 2 Å below the metal centre was set to 3.5 Å, while for the atoms, we adopted the Bondi radii scaled by 1.17, and a mesh of 0.1 Å was used to scan the sphere for buried voxels. The steric maps were generated also with the SambVca package [69].

Aromaticity was evaluated by means of the nucleus independent chemical shift (NICS) [70–71], proposed by Schleyer et al., as a magnetic descriptor of aromaticity. NICS is defined as the negative value of the absolute shielding computed at a ring centre or at some other interesting point of the system. The more negative the NICS the higher the aromaticity of the ring is considered. NICS values were computed using the gauge-including atomic orbital method (GIAO), at the BP86/SVP level. The magnetic shielding tensor was calculated for ghost atoms located at the centre of the rings determined by the nonweighted mean of the heavy atom coordinates.

## Supporting Information

File 1 All Cartesian coordinates, 3D view and energies of all species, steric maps and NICS aromaticity values.
