# Supplementary material for: The activity of indenylidene derivatives in olefin metathesis catalysts
Source: Beilstein J Org Chem. 2018 Nov 30;14:2956–63. doi: 10.3762/bjoc.14.275 (PMC6278753; doi:10.3762/bjoc.14.275)
Supplement: File 1 — All Cartesian coordinates, 3D view and energies of all species, steric maps and NICS aromaticity values. [file Beilstein_J_Org_Chem-14-2956-s001.pdf]

BEILSTEIN JOURNAL OF ORGANIC CHEMISTRY

Supporting Information

for

The activity of indenylidene derivatives in olefin metathesis catalysts

Maria Voccia, Steven P. Nolan, Luigi Cavallo and Albert Poater

Beilstein J. Org. Chem. **2018,** *14,* 2956–2963. doi:10.3762/bjoc.14.275

All Cartesian coordinates, 3D view and energies of all species, steric maps and NICS aromaticity values

License and Terms: This is a supporting information file under the terms of the Creative Commons Attribution License (http://creativecommons.org/licenses/by/4.0). Please note that the reuse, redistribution and reproduction in particular requires that the authors and source are credited.
The license is subject to the *Beilstein Journal of Organic Chemistry* terms and conditions: (https://www.beilstein-journals.org/bjoc)

Figure S1. Steric maps for intermediates **I** and **II** of catalysts **1–6**.

1-I

NHC

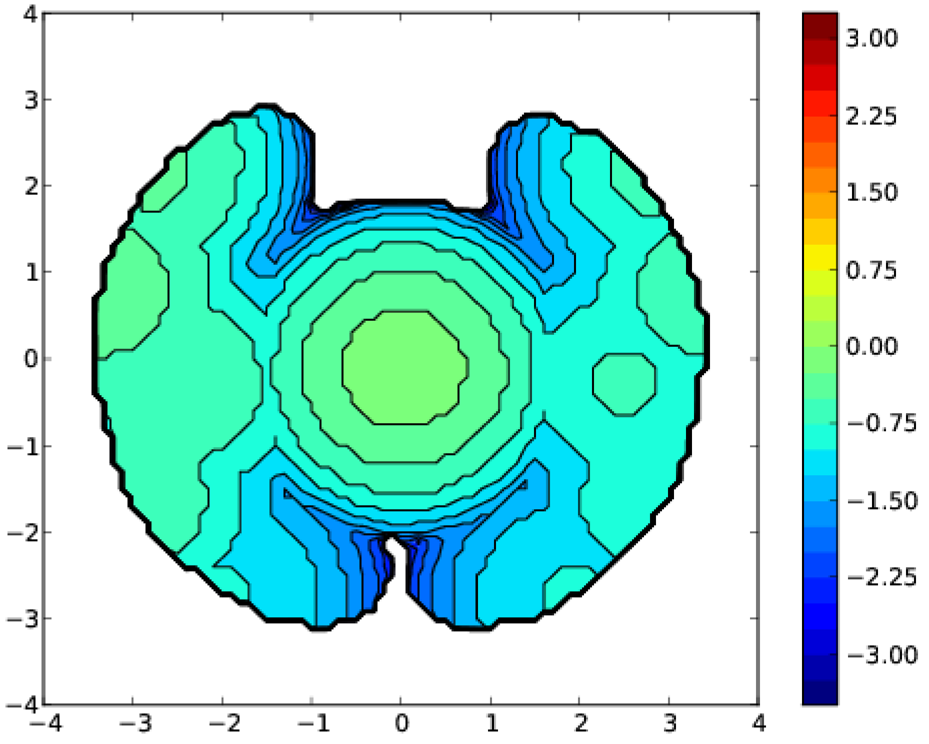

%V_Free	%V_Bur	% Tot/Ex
69.9	**30.1**	100.0

Quadrant	V_f	V_b	V_t	%V_f	%V_b
SW	30.2	14.6	44.9	67.4	**32.6**
NW	31.7	13.2	44.9	70.6	**29.4**
NE	32.6	12.2	44.8	72.8	**27.2**
SE	30.9	13.9	44.9	68.9	**31.1**

Ylidene

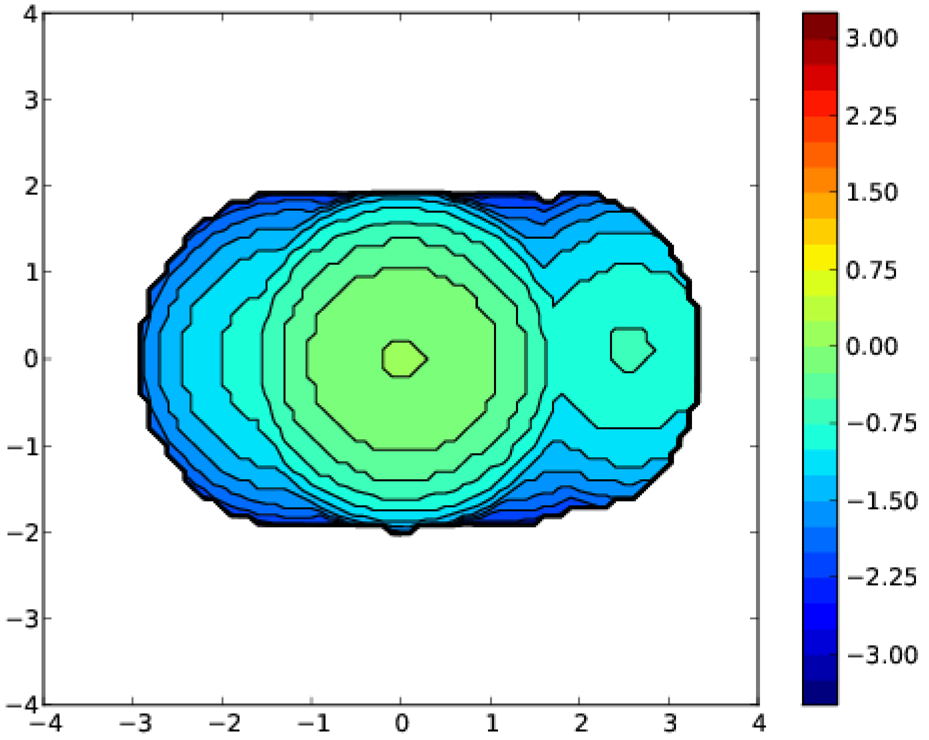

%V_Free	%V_Bur	% Tot/Ex
76.0	**24.0**	100.0

Quadrant	V_f	V_b	V_t	%V_f	%V_b
SW	34.6	10.3	44.9	77.1	**22.9**
NW	34.8	10.1	44.9	77.6	**22.4**
NE	33.3	11.6	44.8	74.2	**25.8**
SE	33.8	11.1	44.9	75.3	**24.7**

2-I

NHC

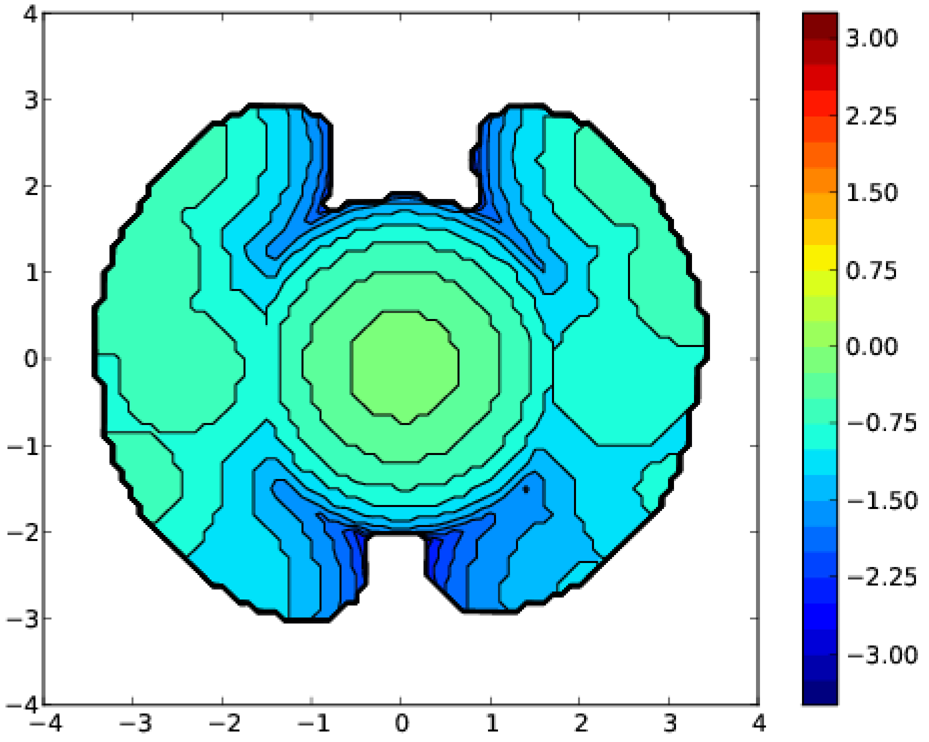

%V_Free	%V_Bur	% Tot/Ex
71.0	29.0	100.0

Quadrant	V_f	V_b	V_t	%V_f	%V_b
SW	31.3	13.6	44.9	69.7	30.3
NW	31.8	13.0	44.9	70.9	29.1
NE	31.9	12.9	44.8	71.2	28.8
SE	32.3	12.5	44.9	72.1	27.9

Ylidene

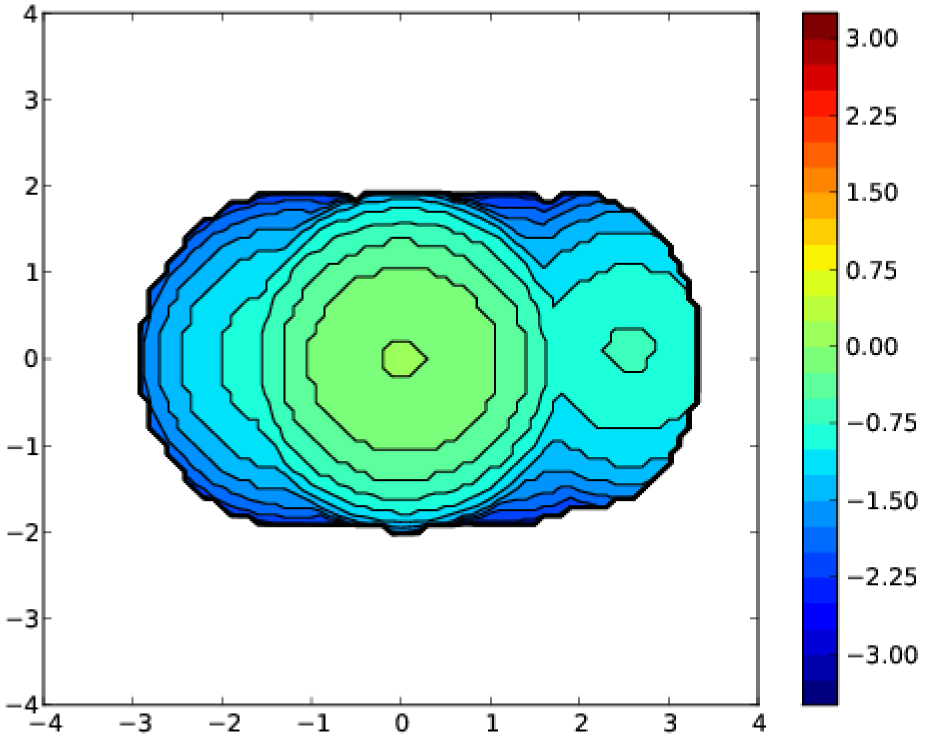

V Free	V Buried	V Total	V Exact
136.5	43.1	179.5	179.6

%V_Free	**%V_Bur**	% Tot/Ex
76.0	**24.0**	100.0

Quadrant	V_f	V_b	V_t	%V_f	**%V_b**
SW	34.6	10.3	44.9	77.1	**22.9**
NW	34.8	10.1	44.9	77.6	**22.4**
NE	33.3	11.6	44.8	74.2	**25.8**
SE	33.8	11.1	44.9	75.3	**24.7**

3-I

NHC

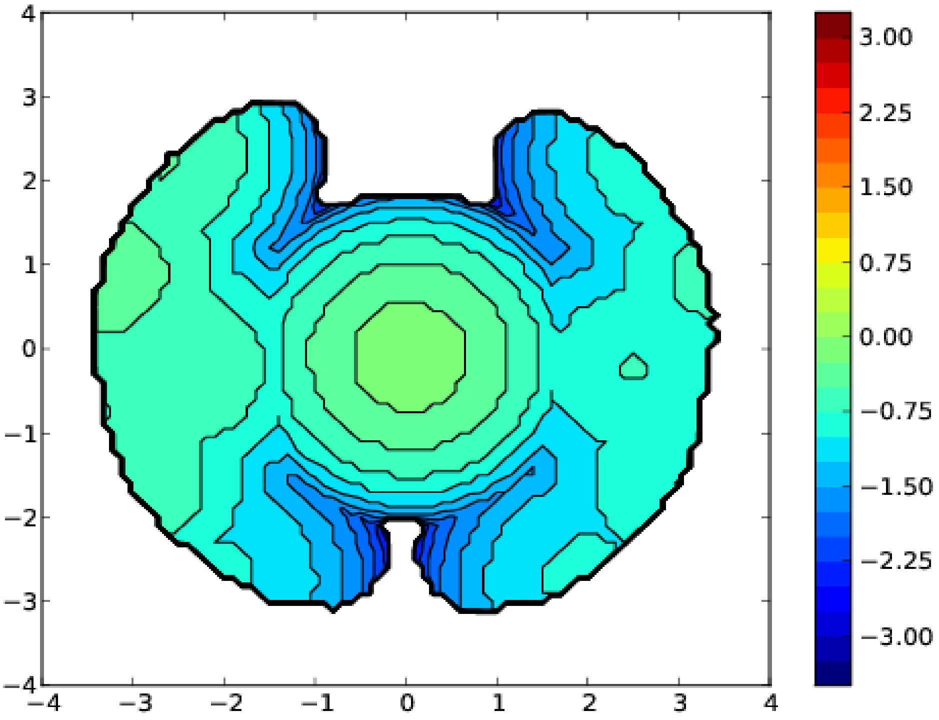

%V_Free	%V_Bur	% Tot/Ex
70.2	29.8	100.0

Quadrant	V_f	V_b	V_t	%V_f	%V_b
SW	30.5	14.4	44.9	68.0	32.0
NW	31.6	13.3	44.9	70.4	29.6
NE	32.9	12.0	44.8	73.3	26.7
SE	31.1	13.8	44.9	69.3	30.7

Ylidene

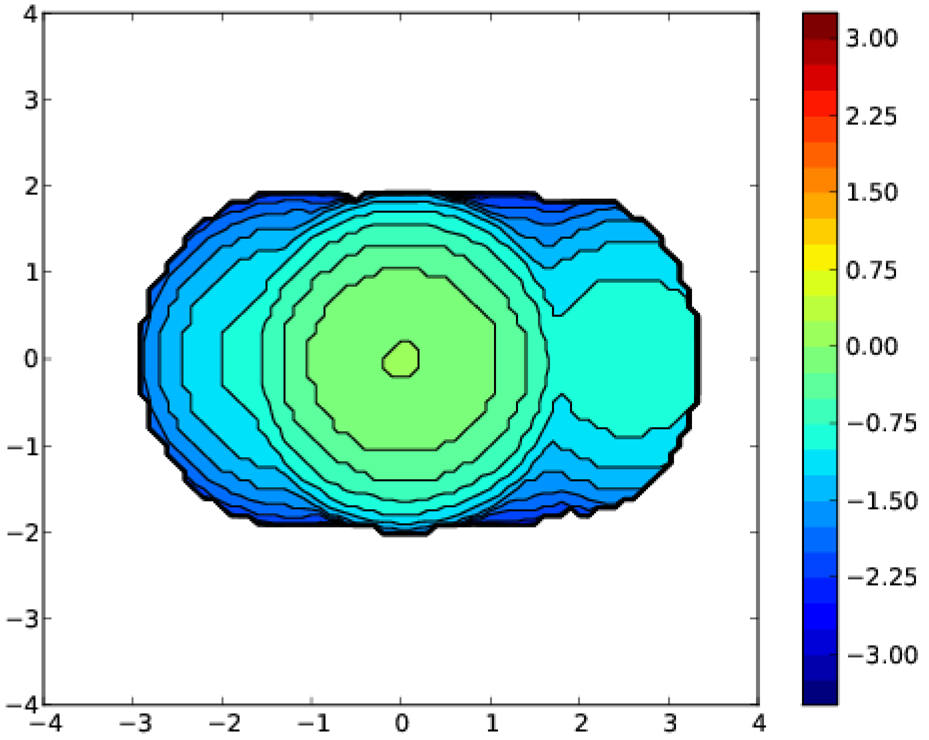

%V_Free	%V_Bur	% Tot/Ex
76.1	23.9	100.0

Quadrant	V_f	V_b	V_t	%V_f	%V_b
SW	34.6	10.3	44.9	77.0	23.0
NW	34.9	10.0	44.9	77.8	22.2
NE	33.5	11.3	44.8	74.7	25.3
SE	33.6	11.2	44.9	75.0	25.0

4-I

NHC

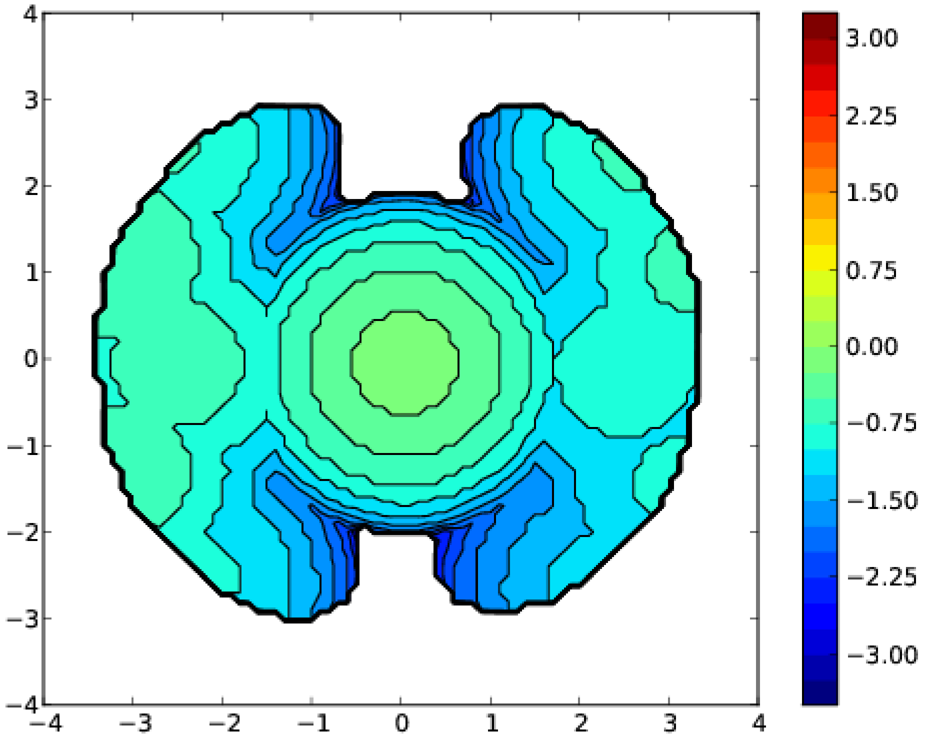

%V_Free	%V_Bur	% Tot/Ex
71.3	28.7	100.0

Quadrant	V_f	V_b	V_t	%V_f	%V_b
SW	31.4	13.5	44.9	69.9	30.1
NW	31.8	13.0	44.9	71.0	29.0
NE	32.1	12.8	44.8	71.5	28.5
SE	32.6	12.3	44.9	72.6	27.4

Ylidene

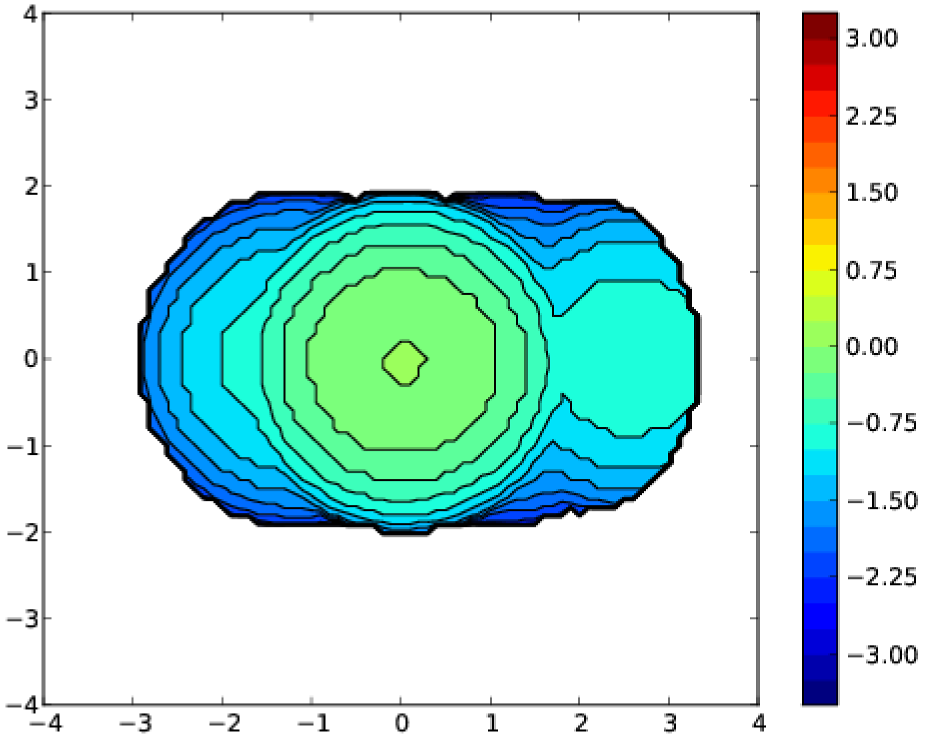

%V_Free	%V_Bur	% Tot/Ex
76.1	23.9	100.0

Quadrant	V_f	V_b	V_t	%V_f	%V_b
SW	34.5	10.3	44.9	77.0	23.0
NW	34.9	10.0	44.9	77.8	22.2
NE	33.5	11.3	44.8	74.7	25.3
SE	33.6	11.2	44.9	75.0	25.0

5-I

NHC

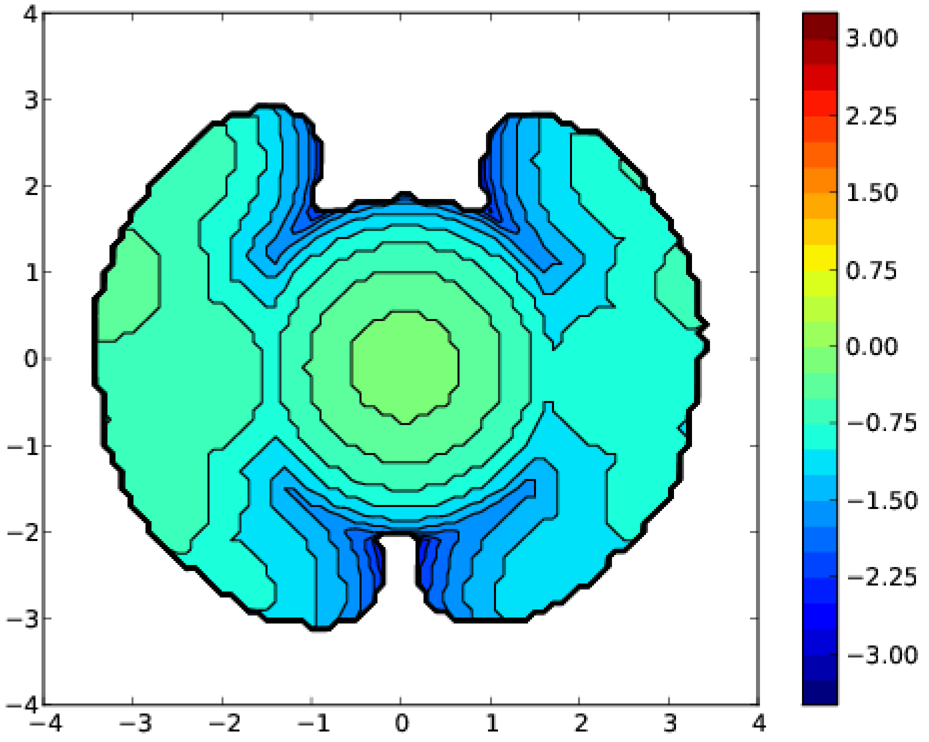

%V_Free	%V_Bur	% Tot/Ex
70.4	29.6	100.0

Quadrant	V_f	V_b	V_t	%V_f	%V_b
SW	30.3	14.6	44.9	67.5	32.5
NW	31.8	13.1	44.9	70.8	29.2
NE	32.7	12.2	44.8	72.9	27.1
SE	31.6	13.3	44.9	70.4	29.6

Ylidene

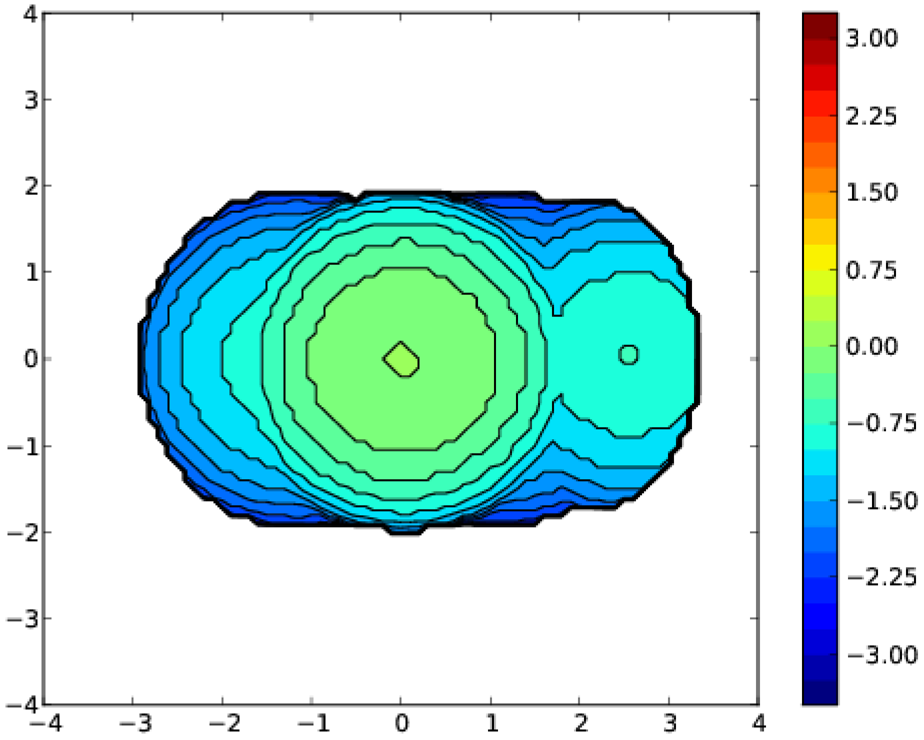

%V_Free	%V_Bur	% Tot/Ex
76.1	23.9	100.0

Quadrant	V_f	V_b	V_t	%V_f	%V_b
SW	34.6	10.3	44.9	77.1	22.9
NW	34.9	10.0	44.9	77.7	22.3
NE	33.4	11.4	44.8	74.5	25.5
SE	33.7	11.1	44.9	75.2	24.8

6-I

NHC

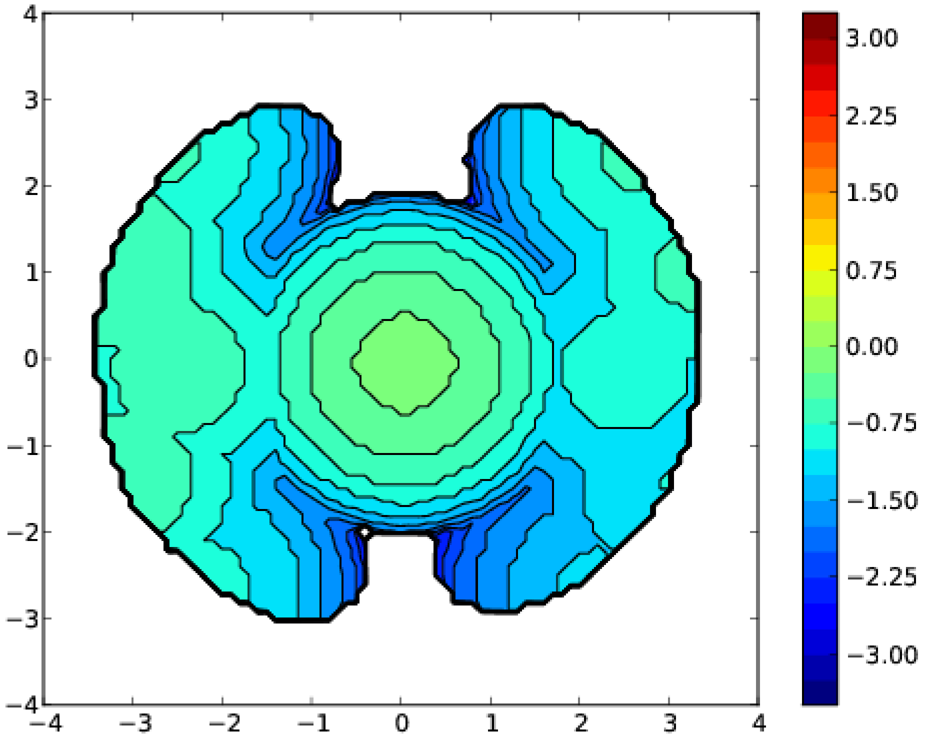

%V_Free	%V_Bur	% Tot/Ex
71.4	28.6	100.0

Quadrant	V_f	V_b	V_t	%V_f	%V_b
SW	31.2	13.7	44.9	69.6	30.4
NW	32.0	12.8	44.9	71.4	28.6
NE	32.3	12.6	44.8	71.9	28.1
SE	32.7	12.2	44.9	72.9	27.1

Ylidene

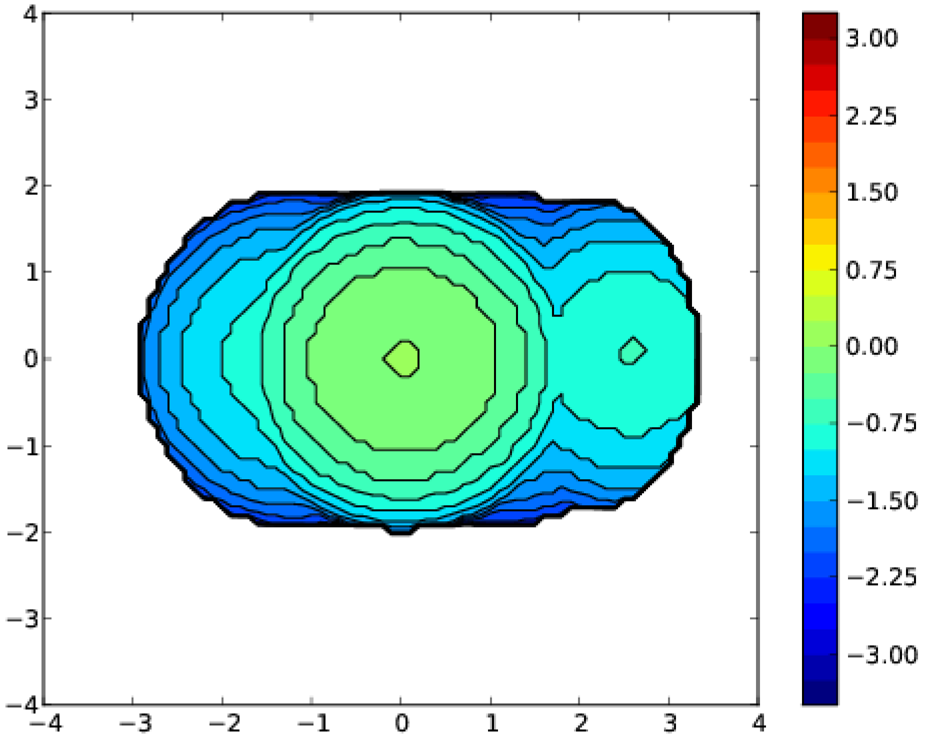

V Free	V Buried	V Total	V Exact
136.6	42.9	179.5	179.6

%V_Free	%V_Bur	% Tot/Ex
76.1	23.9	100.0

Quadrant	V_f	V_b	V_t	%V_f	%V_b
SW	34.6	10.3	44.9	77.1	22.9
NW	34.8	10.0	44.9	77.6	22.4
NE	33.4	11.4	44.8	74.5	25.5
SE	33.7	11.1	44.9	75.2	24.8

Table S1. NICS0, NICS1 and NICS-1 for for intermediates **I** and **II** of catalysts **1**–**6**, for the phenyl and the imidazole rings.

B3LYP/SVP	Phenyl			Imidazole		
	0	1	-1	0	1	-1
**1-I**	8.059	10.286	10.247	-14.845	-4.729	-2.120
**2-I**	8.004	10.248	10.188	-14.882	-4.704	-2.364
**3-I**	8.133	10.257	10.100	-14.618	-4.651	-3.277
**4-I**	8.097	10.210	10.071	-14.916	-4.762	-3.535
**5-I**	8.066	10.375	10.058	-14.686	-4.996	-3.462
**6-I**	8.048	10.378	10.027	-14.928	-5.087	-3.582

BP86/SVP	Phenyl			Imidazole		
	0	1	-1	0	1	-1
**1-I**	7.382	9.769	9.766	-16.032	-2.395	-5.386
**2-I**	7.328	9.732	9.707	-15.918	-2.617	-5.313
**3-I**	7.533	9.726	9.717	-15.269	-3.496	-5.130
**4-I**	7.493	9.690	9.668	-15.661	-3.800	-5.289
**5-I**	7.465	9.872	9.657	-15.133	-3.641	-5.392
**6-I**	7.445	9.875	9.618	-15.443	-5.515	-3.786

B3LYP/SVP	Phenyl			Imidazole		
	0	1	-1	0	1	-1
**1-II**	7.871	10.114	9.966	-11.358	-2.180	-3.061
**2-II**	7.824	10.052	9.973	-11.403	-2.263	-2.937
**3-II**	8.019	10.116	9.885	-11.487	-3.300	-2.201
**4-II**	7.952	9.994	9.866	-11.272	-3.180	-2.100
**5-II**	7.991	10.062	9.943	-11.501	-2.116	-3.481
**6-II**	7.944	10.024	9.875	-11.256	-1.993	-3.271

BP86/SVP	Phenyl			Imidazole		
	0	1	-1	0	1	-1
**1-I**	7.254	9.644	9.504	-10.424	-2.100	-2.699
**2-I**	7.198	9.567	9.510	-10.425	-2.172	-2.575
**3-I**	7.574	9.787	9.522	-10.652	-3.063	-2.157
**4-I**	7.501	9.639	9.503	-10.396	-2.900	-2.063
**5-I**	7.520	9.689	9.565	-10.678	-2.061	-3.247
**6-I**	7.478	9.651	9.498	-10.421	-1.940	-3.036

Table S2. 3D view and *xyz* coordinate data sets and absolute energies in a.u. for DFT optimized complexes.

Catalyst 1, intermediate I

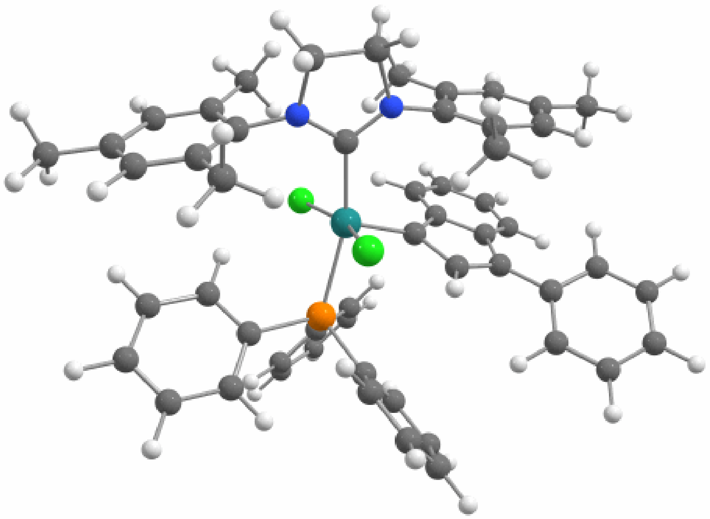

Zero-point correction= 0.873238 (Hartree/Particle)
Thermal correction to Energy= 0.933272
Thermal correction to Enthalpy= 0.934216
Thermal correction to Gibbs Free Energy= 0.780704
Sum of electronic and zero-point Energies= -3552.126094
Sum of electronic and thermal Energies= -3552.066060
Sum of electronic and thermal Enthalpies= -3552.065116
Sum of electronic and thermal Free Energies= -3552.218628

Solvent: -3553.6634659

Ru	-0,607400000	-0,313931000	-0,036808000
Cl	-1,533190000	-0,592341000	2,168959000
Cl	-0,416548000	0,000790000	-2,443287000
P	-1,077482000	2,090478000	0,155738000
N	-1,976945000	-2,864995000	-0,944152000
N	0,202875000	-3,198752000	-0,875641000
C	-0,784959000	-2,311253000	-0,556033000
C	-1,801181000	-4,126794000	-1,699336000
H	-2,044013000	-3,965072000	-2,772234000
H	-2,484138000	-4,909094000	-1,308080000
C	-0,317490000	-4,448245000	-1,478453000
H	-0,157337000	-5,302302000	-0,782722000
H	0,221877000	-4,676904000	-2,420311000
C	-3,324664000	-2,445605000	-0,643200000
C	-3,895431000	-2,856905000	0,595943000
C	-3,091420000	-3,618491000	1,619933000
H	-3,750163000	-4,051026000	2,398417000
H	-2,366964000	-2,934704000	2,113989000
H	-2,509222000	-4,444664000	1,161063000
C	-5,245235000	-2,549670000	0,845168000
H	-5,686510000	-2,860287000	1,807383000
C	-6,049223000	-1,881709000	-0,101248000
C	-7,502982000	-1,585389000	0,195947000
H	-7,978064000	-1,002753000	-0,618327000
H	-7,616903000	-1,011293000	1,140379000
H	-8,086957000	-2,522701000	0,323386000
C	-5,465850000	-1,530492000	-1,331056000
H	-6,080000000	-1,024337000	-2,094566000
C	-4,117073000	-1,814529000	-1,637766000
C	-3,584946000	-1,499904000	-3,015687000
H	-4,216187000	-0,737604000	-3,513845000
H	-3,603979000	-2,405198000	-3,663746000
H	-2,541500000	-1,123885000	-2,989693000
C	1,587942000	-3,200776000	-0,482565000
C	2,588752000	-2,882090000	-1,433845000
C	2,236987000	-2,440861000	-2,833922000
H	3,130842000	-2,054251000	-3,361688000
H	1,453064000	-1,654041000	-2,834071000
H	1,843234000	-3,289440000	-3,437671000
C	3,938219000	-3,016367000	-1,043728000
H	4,723550000	-2,755623000	-1,772774000
C	4,309019000	-3,478921000	0,232546000
C	5,761607000	-3,604322000	0,633767000
H	5,979321000	-4,602358000	1,069659000
H	6,023935000	-2,853017000	1,410502000
H	6,441228000	-3,452516000	-0,228410000
C	3,285069000	-3,815070000	1,141675000
H	3,551534000	-4,182883000	2,146608000
C	1,924926000	-3,688381000	0,809203000
C	0,852544000	-4,074528000	1,800503000
H	0,259474000	-4,947504000	1,448636000
H	0,133784000	-3,246400000	1,969083000
H	1,296907000	-4,349706000	2,776612000
H	1,859236000	0,980625000	-1,570260000
C	2,152960000	0,626066000	-0,575744000
C	3,434842000	0,641101000	-0,058484000
C	1,218955000	-0,052280000	0,334093000
C	3,386492000	0,029285000	1,296646000
C	2,042602000	-0,400800000	1,543966000
C	4,363751000	-0,075871000	2,293867000
C	1,702001000	-0,933560000	2,793186000
C	4,011760000	-0,633130000	3,546734000
H	5,386801000	0,294105000	2,125235000
C	2,696501000	-1,052867000	3,792957000
H	0,660303000	-1,222492000	2,995086000
H	4,775135000	-0,719042000	4,336931000
H	2,427801000	-1,469567000	4,776893000
C	4,630646000	1,160670000	-0,740515000
C	5,900379000	0,544003000	-0,589976000
C	4,531914000	2,284474000	-1,602569000
C	7,024391000	1,031795000	-1,274919000
H	5,993655000	-0,352097000	0,041324000
C	5,658177000	2,772698000	-2,279710000
H	3,559692000	2,788608000	-1,716424000
C	6,910444000	2,150349000	-2,119334000
H	7,997898000	0,530321000	-1,151984000
H	5,559539000	3,652328000	-2,935837000
H	7,794697000	2,536475000	-2,651092000
C	-2,836456000	2,425308000	-0,333103000
C	-3,220388000	3,501291000	-1,163771000
C	-3,833932000	1,591324000	0,223445000
C	-4,579648000	3,735048000	-1,438376000
H	-2,458143000	4,168511000	-1,592680000
C	-5,190426000	1,838253000	-0,046295000
H	-3,545011000	0,758531000	0,886725000
C	-5,567425000	2,906360000	-0,879169000
H	-4,865429000	4,577685000	-2,088381000

Catalyst 1, I-II TS

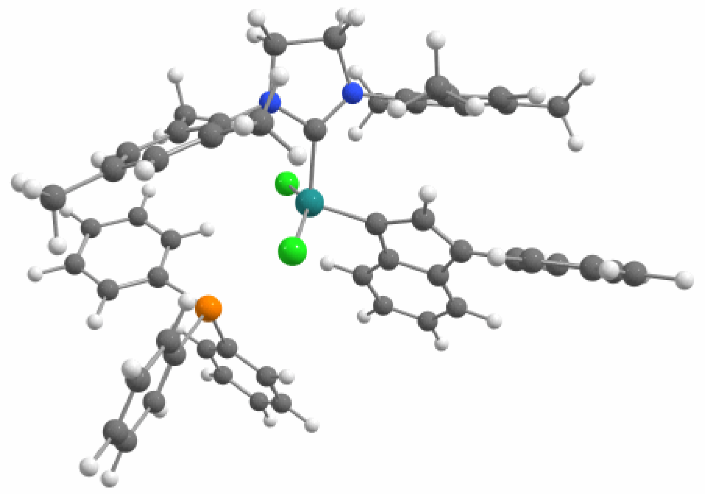

Zero-point correction= 0.871988 (Hartree/Particle)
Thermal correction to Energy= 0.932013
Thermal correction to Enthalpy= 0.932957
Thermal correction to Gibbs Free Energy= 0.774433
Sum of electronic and zero-point Energies= -3552.102840
Sum of electronic and thermal Energies= -3552.042815
Sum of electronic and thermal Enthalpies= -3552.041871
Sum of electronic and thermal Free Energies= -3552.200395

Solvent: -3553.6233015

Ru	-0,372392000	-0,559401000	-0,143162000
Cl	0,327467000	-0,420841000	-2,376432000
Cl	0,166598000	-0,495005000	2,123304000
P	3,377761000	1,281749000	-0,028263000
N	-0,448706000	-3,422133000	-0,344325000
N	-2,435638000	-2,662323000	-0,975211000
C	-1,220550000	-2,280907000	-0,446223000
C	-1,068591000	-4,588305000	-0,996389000
H	-0,971667000	-5,490319000	-0,357639000
H	-0,569994000	-4,802139000	-1,968803000
C	-2,520847000	-4,126518000	-1,176259000
H	-2,926677000	-4,350006000	-2,183924000
H	-3,206940000	-4,581751000	-0,427062000
C	0,825156000	-3,549831000	0,315586000
C	2,029890000	-3,559927000	-0,436297000
C	2,039903000	-3,494435000	-1,945653000
H	3,011534000	-3,110763000	-2,314271000
H	1,245276000	-2,834408000	-2,344861000
H	1,901776000	-4,509414000	-2,382173000
C	3,246332000	-3,703380000	0,263921000
H	4,187266000	-3,681744000	-0,310608000
C	3,294062000	-3,881191000	1,659052000
C	4,615566000	-3,987221000	2,386114000
H	4,530635000	-4,605649000	3,302919000
H	4,972481000	-2,981452000	2,700003000
H	5,404709000	-4,425701000	1,742266000
C	2,073154000	-3,944333000	2,361724000
H	2,084802000	-4,121388000	3,450406000
C	0,832979000	-3,790927000	1,717475000
C	-0,454221000	-3,886985000	2,501344000
H	-0,266147000	-4,266905000	3,524609000
H	-1,187531000	-4,563502000	2,013490000
H	-0,927884000	-2,886585000	2,592076000
C	-3,657423000	-1,919564000	-1,151133000
C	-4,645455000	-1,971114000	-0,130215000
C	-4,409327000	-2,698251000	1,176511000
H	-5,003525000	-2,243186000	1,994006000
H	-3,343126000	-2,686909000	1,478482000
H	-4,722350000	-3,764591000	1,113010000
C	-5,876840000	-1,327029000	-0,353005000
H	-6,637691000	-1,348884000	0,445357000
C	-6,156782000	-0,646327000	-1,553981000
C	-7,475555000	0,067742000	-1,746724000
H	-8,332803000	-0,571539000	-1,448335000
H	-7,625634000	0,374969000	-2,800886000
H	-7,524003000	0,983794000	-1,118564000
C	-5,169022000	-0,644359000	-2,556838000
H	-5,375735000	-0,138861000	-3,515107000
C	-3,923452000	-1,284074000	-2,391584000
C	-2,925024000	-1,305479000	-3,521470000
H	-2,625516000	-2,343616000	-3,781584000
H	-1,987985000	-0,774117000	-3,254373000
H	-3,348838000	-0,836698000	-4,430911000
H	-3,326969000	-0,274455000	1,285223000
C	-2,996789000	0,607569000	0,729764000
C	-3,620905000	1,845275000	0,765748000
C	-1,785630000	0,661631000	-0,073354000
C	-2,825394000	2,782359000	-0,071735000
C	-1,692884000	2,064104000	-0,577819000
C	-2,979971000	4,143279000	-0,358990000
C	-0,752487000	2,705604000	-1,393547000
C	-2,022130000	4,786311000	-1,181596000
H	-3,821394000	4,720059000	0,054324000
C	-0,928132000	4,077122000	-1,697540000
H	0,107902000	2,148465000	-1,793018000
H	-2,141000000	5,856941000	-1,413376000
H	-0,189495000	4,589011000	-2,333664000
C	-4,833524000	2,168009000	1,534010000
C	-5,823565000	3,050604000	1,027011000
C	-5,058000000	1,575627000	2,804940000
C	-6,989739000	3,323607000	1,759762000
H	-5,690199000	3,497687000	0,030448000
C	-6,221064000	1,854702000	3,536618000
H	-4,286745000	0,913616000	3,228982000
C	-7,193465000	2,730452000	3,018437000
H	-7,748558000	4,004562000	1,341726000
H	-6,366161000	1,393585000	4,526821000
H	-8,105008000	2,952365000	3,595825000
C	4,224257000	1,343558000	1,627165000
C	5,222285000	2,284730000	1,969896000
C	3,816559000	0,393971000	2,592907000
C	5,810932000	2,265779000	3,246567000
H	5,537092000	3,040855000	1,232976000
C	4,411948000	0,374528000	3,866840000
H	3,009571000	-0,316650000	2,349279000
C	5,410078000	1,308687000	4,196304000
H	6,586882000	3,005885000	3,501518000
H	4,079176000	-0,365580000	4,612389000
H	5,869033000	1,298734000	5,198165000
C	3,631704000	3,022486000	-0,637839000
C	4,547857000	3,394152000	-1,646973000
C	2,799331000	4,021100000	-0,075755000
C	4,634684000	4,731754000	-2,075815000
H	5,200634000	2,633159000	-2,102497000
C	2,898405000	5,357584000	-0,494508000
H	2,065153000	3,746372000	0,699975000
C	3,815380000	5,717524000	-1,499837000
H	5,354643000	5,003894000	-2,864825000
H	2,246732000	6,120821000	-0,039532000
H	3,887566000	6,764481000	-1,835695000
C	4,592983000	0,326814000	-1,064026000

C	5,891648000	-0,035016000	-0,641295000
C	4,153081000	-0,091047000	-2,343493000
C	6,734478000	-0,784658000	-1,482301000
H	6,249348000	0,272158000	0,353959000
C	5,003456000	-0,824884000	-3,188217000
H	3,127375000	0,147738000	-2,670034000
C	6,296887000	-1,176547000	-2,759578000
H	7,744295000	-1,059138000	-1,136000000
H	4,647635000	-1,132726000	-4,184863000
H	6,960870000	-1,758618000	-3,418858000

Catalyst 1, II intermediate

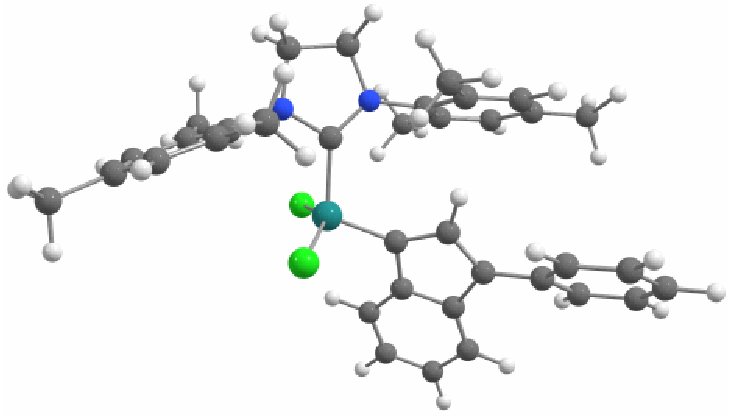

Zero-point correction= 0.604728 (Hartree/Particle)
Thermal correction to Energy= 0.646740
Thermal correction to Enthalpy= 0.647684
Thermal correction to Gibbs Free Energy= 0.531472
Sum of electronic and zero-point Energies= -2516.646700
Sum of electronic and thermal Energies= -2516.604688
Sum of electronic and thermal Enthalpies= -2516.603744
Sum of electronic and thermal Free Energies= -2516.719955

Solvent: -2517.6679136

Ru	1,022871000	0,842987000	-0,228882000
Cl	1,569718000	1,142018000	-2,476424000
Cl	1,452099000	1,638930000	1,902667000
N	2,775284000	-1,436085000	-0,203115000
N	0,731664000	-2,174340000	-0,648227000
C	1,450923000	-1,054744000	-0,303868000
C	2,989858000	-2,868818000	-0,471804000
H	3,352489000	-3,384460000	0,445755000
H	3,758998000	-3,009615000	-1,259742000
C	1,596161000	-3,345223000	-0,912632000
H	1,556825000	-3,605670000	-1,993357000
H	1,239303000	-4,230076000	-0,344762000
C	3,861022000	-0,653390000	0,333316000
C	4,820200000	-0,080475000	-0,544688000
C	4,761213000	-0,289702000	-2,039077000
H	5,466754000	0,387719000	-2,558990000
H	3,744787000	-0,104975000	-2,441160000
H	5,049706000	-1,330103000	-2,310289000
C	5,879809000	0,656523000	0,023493000
H	6,617158000	1,121491000	-0,652302000
C	6,032577000	0,803504000	1,414951000
C	7,150143000	1,640889000	1,994072000
H	7,522330000	1,222494000	2,951678000
H	6,797706000	2,674259000	2,207086000
H	8,006716000	1,724207000	1,295124000
C	5,106322000	0,153850000	2,255564000
H	5,228401000	0,222020000	3,349528000
C	4,025071000	-0,586190000	1,744650000
C	3,076204000	-1,294726000	2,682183000
H	3,447660000	-1,250858000	3,724496000
H	2,945652000	-2,364515000	2,413904000
H	2,075627000	-0,816110000	2,659809000
C	-0,687625000	-2,378120000	-0,753736000
C	-1,367718000	-2,914529000	0,371583000
C	-0,651522000	-3,134933000	1,685940000
H	-1,358458000	-3,468700000	2,470439000
H	-0,153379000	-2,208673000	2,042574000
H	0,142229000	-3,909565000	1,607013000
C	-2,737997000	-3,215334000	0,247946000
H	-3,273626000	-3,621206000	1,122360000
C	-3,441123000	-3,000440000	-0,953893000
C	-4,922882000	-3,283365000	-1,053219000
H	-5,197417000	-3,679893000	-2,052253000
H	-5,512009000	-2,353376000	-0,896669000
H	-5,253739000	-4,013457000	-0,287502000
C	-2,727258000	-2,490925000	-2,056396000
H	-3,254696000	-2,331120000	-3,011912000
C	-1,352755000	-2,185500000	-1,991354000
C	-0,621655000	-1,687199000	-3,213718000
H	0,187625000	-2,386040000	-3,519127000
H	-0,136324000	-0,705275000	-3,035917000
H	-1,313161000	-1,585465000	-4,072650000
H	-1,785848000	-0,606239000	0,975950000
C	-1,894438000	0,363508000	0,482960000
C	-3,057256000	1,122563000	0,480492000
C	-0,834748000	1,050177000	-0,232298000
C	-2,794049000	2,365861000	-0,288182000
C	-1,433252000	2,325352000	-0,734096000
C	-3,592278000	3,482528000	-0,565915000
C	-0,903676000	3,378977000	-1,491540000
C	-3,046248000	4,548117000	-1,320915000
H	-4,626529000	3,547379000	-0,194850000
C	-1,723415000	4,493945000	-1,785052000
H	0,131604000	3,335288000	-1,862678000
H	-3,670323000	5,428147000	-1,544305000
H	-1,313801000	5,327133000	-2,377472000
C	-4,317829000	0,762750000	1,149240000
C	-5,577786000	1,043101000	0,557573000
C	-4,304523000	0,100130000	2,404776000
C	-6,772237000	0,673046000	1,195766000
H	-5,615782000	1,524148000	-0,431434000
C	-5,499492000	-0,261131000	3,043341000
H	-3,337356000	-0,100676000	2,891546000
C	-6,740140000	0,023722000	2,442859000
H	-7,738011000	0,892171000	0,712548000
H	-5,463987000	-0,760819000	4,024781000
H	-7,678562000	-0,256933000	2,946936000

Catalyst 1, I-III TS

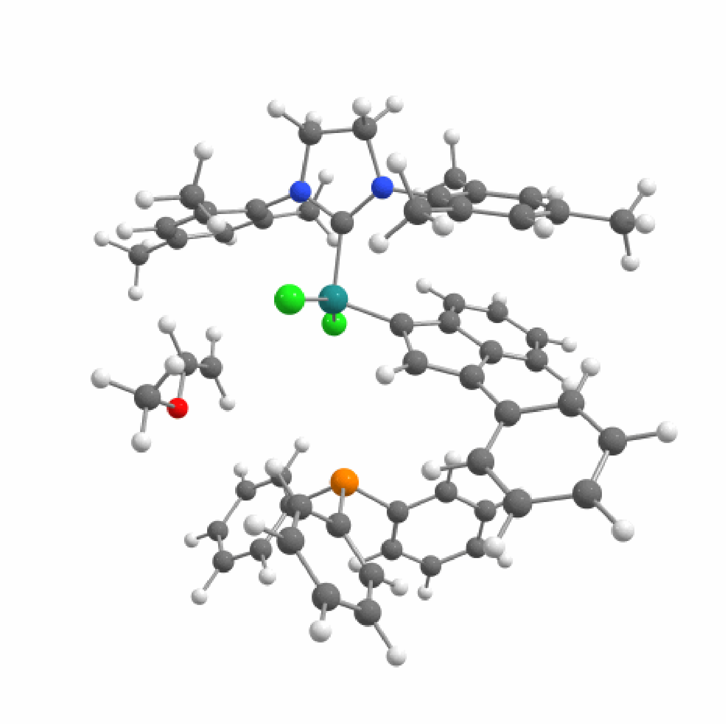

Zero-point correction= 0.954319 (Hartree/Particle)
Thermal correction to Energy= 1.021624
Thermal correction to Enthalpy= 1.022568
Thermal correction to Gibbs Free Energy= 0.844766
Sum of electronic and zero-point Energies= -3744.982228
Sum of electronic and thermal Energies= -3744.914923
Sum of electronic and thermal Enthalpies= -3744.913979
Sum of electronic and thermal Free Energies= -3745.091781

Solvent: -3746.6805068

Ru	-1.495409000	-0.324658000	0.087285000
Cl	-1.241249000	-1.183849000	-2.067722000
Cl	-1.355395000	-0.557265000	2.440898000
P	3.070077000	-1.470870000	-0.540716000
N	-4.371116000	-0.393405000	0.295733000
N	-3.734299000	1.730169000	0.386330000
C	-3.279373000	0.446590000	0.190138000
C	-5.636015000	0.326338000	0.526140000
H	-6.175642000	-0.094563000	1.400291000
H	-6.304927000	0.227322000	-0.358773000
C	-5.164511000	1.770720000	0.757799000
H	-5.709765000	2.508265000	0.133657000
H	-5.270116000	2.087536000	1.819278000
C	-4.407592000	-1.802588000	-0.003534000
C	-4.561805000	-2.218898000	-1.354060000
C	-4.655255000	-1.221214000	-2.483488000
H	-4.974048000	-1.717347000	-3.420942000
H	-3.665225000	-0.753101000	-2.666383000
H	-5.380254000	-0.409282000	-2.264944000
C	-4.639141000	-3.599521000	-1.620984000
H	-4.740708000	-3.928567000	-2.668667000
C	-4.607826000	-4.564501000	-0.595205000
C	-4.642561000	-6.042349000	-0.911517000
H	-5.190503000	-6.615539000	-0.135868000
H	-3.611671000	-6.457982000	-0.955146000
H	-5.118467000	-6.242132000	-1.892720000
C	-4.535556000	-4.111793000	0.737135000
H	-4.558789000	-4.848052000	1.558273000
C	-4.448349000	-2.743190000	1.062664000
C	-4.431472000	-2.305394000	2.507595000
H	-4.513392000	-3.179372000	3.183562000
H	-5.279914000	-1.626524000	2.741265000
H	-3.499615000	-1.751680000	2.753511000
C	-3.058628000	2.992875000	0.241459000
C	-2.372488000	3.574513000	1.338746000
C	-2.297101000	2.892285000	2.682060000
H	-1.651220000	3.463228000	3.377399000
H	-1.905044000	1.857243000	2.610080000
H	-3.303129000	2.818885000	3.151611000
C	-1.789927000	4.845713000	1.154990000
H	-1.235812000	5.294742000	1.996130000
C	-1.906708000	5.561072000	-0.051700000
C	-1.246329000	6.908655000	-0.235033000
H	-1.939973000	7.645739000	-0.691380000
H	-0.371726000	6.826636000	-0.917305000
H	-0.885501000	7.325019000	0.726577000
C	-2.648906000	4.979032000	-1.097999000
H	-2.768295000	5.527862000	-2.047188000
C	-3.232124000	3.705027000	-0.975946000
C	-4.049305000	3.139508000	-2.115748000
H	-5.132400000	3.365985000	-1.989296000
H	-3.952520000	2.039167000	-2.194403000
H	-3.739915000	3.583418000	-3.082348000
H	0.846543000	0.989333000	1.724640000
C	0.684473000	1.508236000	0.771901000
C	1.349322000	2.655462000	0.353346000
C	-0.401257000	1.183096000	-0.150053000
C	0.803949000	3.034294000	-0.978580000
C	-0.267086000	2.135147000	-1.285073000
C	1.247440000	3.954270000	-1.936630000
C	-0.858886000	2.146141000	-2.551171000
C	0.617715000	3.983728000	-3.205838000
H	2.100170000	4.620234000	-1.732276000
C	-0.414497000	3.085526000	-3.512580000
H	-1.636144000	1.409718000	-2.798145000
H	0.964083000	4.702374000	-3.965947000
H	-0.874780000	3.100279000	-4.513406000
C	2.385312000	3.369263000	1.110306000
C	2.523282000	4.780968000	1.026669000
C	3.258624000	2.662682000	1.981101000
C	3.492234000	5.456931000	1.783473000
H	1.832184000	5.353823000	0.390743000
C	4.228166000	3.341801000	2.731021000
H	3.190294000	1.565484000	2.044976000
C	4.351208000	4.741064000	2.636515000
H	3.573507000	6.553484000	1.711614000
H	4.900149000	2.769645000	3.389778000
H	5.115721000	5.271787000	3.226119000
C	3.341881000	-3.268671000	-0.928920000
C	4.255668000	-4.104827000	-0.249468000
C	2.543672000	-3.822662000	-1.958412000
C	4.376090000	-5.461753000	-0.599887000
H	4.875130000	-3.691712000	0.562093000
C	2.676344000	-5.174848000	-2.315678000
H	1.805136000	-3.186465000	-2.474369000
C	3.591918000	-5.998762000	-1.635582000
H	5.090499000	-6.103365000	-0.058759000
H	2.050008000	-5.590842000	-3.121234000
H	3.687776000	-7.062172000	-1.907868000
C	3.979831000	-1.271980000	1.069406000
C	5.276918000	-0.724391000	1.188659000
C	3.279891000	-1.632527000	2.247598000
C	5.865823000	-0.549764000	2.454770000
H	5.831130000	-0.430489000	0.283501000

C	3.875077000	-1.464454000	3.509264000
H	2.262366000	-2.053362000	2.174117000
C	5.168964000	-0.920468000	3.618033000
H	6.879688000	-0.123744000	2.529370000
H	3.319748000	-1.751846000	4.416674000
H	5.630998000	-0.783901000	4.609119000
C	4.217096000	-0.643728000	-1.750248000
C	5.242522000	-1.309531000	-2.459001000
C	4.004751000	0.733786000	-1.993012000
C	6.042466000	-0.610190000	-3.380361000
H	5.414208000	-2.384440000	-2.290845000
C	4.812632000	1.433227000	-2.905012000
H	3.190306000	1.258882000	-1.467028000
C	5.832761000	0.762253000	-3.603200000
H	6.836130000	-1.143618000	-3.928539000
H	4.632348000	2.505723000	-3.081126000
H	6.459637000	1.307242000	-4.327315000
C	-0.778215000	-3.977712000	1.642500000
C	-0.707036000	-4.006364000	0.296463000
H	-1.637272000	-3.992127000	-0.287492000
H	0.255361000	-4.042567000	-0.234911000
H	-1.750686000	-3.928894000	2.168171000
O	0.321498000	-4.003848000	2.448978000
C	0.057444000	-3.878021000	3.841506000
H	1.031276000	-3.950549000	4.363154000
H	-0.412036000	-2.896899000	4.075532000
H	-0.602359000	-4.698169000	4.209333000

Catalyst 1, II-III TS

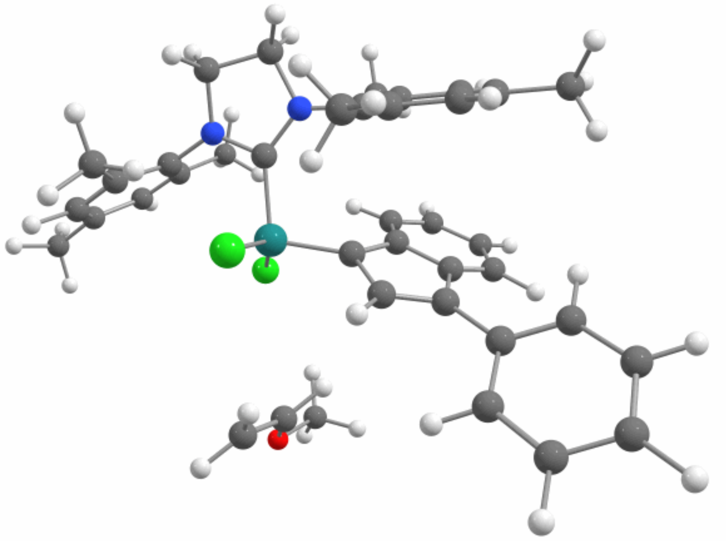

Zero-point correction= 0.687564 (Hartree/Particle)
Thermal correction to Energy= 0.736566
Thermal correction to Enthalpy= 0.737511
Thermal correction to Gibbs Free Energy= 0.604359
Sum of electronic and zero-point Energies= -2709.528982
Sum of electronic and thermal Energies= -2709.479980
Sum of electronic and thermal Enthalpies= -2709.479036
Sum of electronic and thermal Free Energies= -2709.612188

Solvent: -2710.7228011

Ru	-0.966720000	0.512815000	-0.279317000
Cl	-1.772010000	1.416680000	1.707910000
Cl	-0.840735000	0.753715000	-2.611610000
N	-2.853228000	-1.637256000	-0.538422000
N	-0.820797000	-2.530071000	-0.539245000
C	-1.512610000	-1.350592000	-0.381955000
C	-3.088810000	-3.016938000	-0.997994000
H	-3.372200000	-3.026946000	-2.074467000
H	-3.915287000	-3.484422000	-0.423822000
C	-1.726807000	-3.682038000	-0.747800000
H	-1.733759000	-4.339310000	0.150511000
H	-1.377563000	-4.291106000	-1.606562000
C	-3.951944000	-0.731899000	-0.318922000
C	-4.449977000	-0.588896000	1.005621000
C	-3.843806000	-1.361721000	2.152702000
H	-4.438779000	-1.228604000	3.077186000
H	-2.812337000	-1.009365000	2.360356000
H	-3.789229000	-2.449606000	1.934192000
C	-5.531673000	0.281935000	1.222399000
H	-5.909832000	0.409308000	2.250542000
C	-6.142773000	0.991167000	0.167890000
C	-7.264353000	1.968192000	0.438214000
H	-7.897479000	2.125587000	-0.458064000
H	-6.857933000	2.960969000	0.732493000
H	-7.916087000	1.626463000	1.268423000
C	-5.668130000	0.773345000	-1.138695000
H	-6.155252000	1.289909000	-1.982781000
C	-4.589192000	-0.092554000	-1.415554000
C	-4.186343000	-0.360499000	-2.846527000
H	-4.567282000	0.434375000	-3.517418000
H	-4.616146000	-1.322411000	-3.206245000
H	-3.085624000	-0.405641000	-2.969212000
C	0.580288000	-2.815405000	-0.369300000
C	1.457193000	-2.722299000	-1.485730000
C	0.964610000	-2.289100000	-2.843514000
H	1.772557000	-2.364318000	-3.597231000
H	0.584008000	-1.246212000	-2.838042000
H	0.119518000	-2.923039000	-3.189359000
C	2.804124000	-3.086797000	-1.303556000
H	3.489423000	-3.005964000	-2.164181000
C	3.295991000	-3.566237000	-0.071636000
C	4.752641000	-3.943727000	0.080434000
H	5.046633000	-4.725733000	-0.652111000
H	4.973676000	-4.328550000	1.095719000
H	5.414715000	-3.070653000	-0.103611000
C	2.389570000	-3.695567000	0.994847000
H	2.746156000	-4.085273000	1.962989000
C	1.030537000	-3.340496000	0.869082000
C	0.093584000	-3.579303000	2.031893000
H	-0.292346000	-4.623710000	2.023630000
H	-0.780274000	-2.900018000	2.018291000
H	0.619687000	-3.440654000	2.996732000
H	1.720995000	1.469081000	-1.643754000
C	1.890318000	1.079331000	-0.632163000
C	3.126494000	0.976976000	-0.001470000
C	0.853709000	0.429225000	0.164987000
C	2.908058000	0.335098000	1.321645000
C	1.520984000	-0.009514000	1.418334000
C	3.752167000	0.170686000	2.426966000
C	0.990226000	-0.472643000	2.625661000
C	3.216992000	-0.349294000	3.630801000
H	4.808357000	0.478759000	2.386294000
C	1.852159000	-0.658053000	3.732723000
H	-0.088929000	-0.660231000	2.712262000
H	3.876330000	-0.482166000	4.503464000
H	1.443881000	-1.030498000	4.685882000
C	4.410189000	1.420514000	-0.559555000
C	5.621476000	0.733133000	-0.280554000
C	4.465528000	2.546403000	-1.424711000
C	6.834711000	1.152900000	-0.846883000
H	5.598773000	-0.161722000	0.359151000
C	5.679842000	2.966173000	-1.983739000
H	3.542362000	3.109914000	-1.631779000
C	6.870780000	2.271786000	-1.698101000
H	7.760709000	0.598025000	-0.626208000
H	5.700024000	3.848395000	-2.643192000
H	7.825010000	2.603811000	-2.137086000
C	0.125209000	4.371508000	-0.010876000
C	-0.253779000	4.548130000	-1.294285000
H	0.075573000	3.835866000	-2.062615000
H	-0.914870000	5.379479000	-1.583949000
H	0.779810000	3.529362000	0.289998000
O	-0.259419000	5.204502000	0.992410000
C	0.155303000	4.824138000	2.301240000
H	-0.242330000	5.586964000	2.998094000
H	-0.250241000	3.826710000	2.581365000
H	1.266528000	4.805479000	2.389829000

Catalyst 1, III intermediate

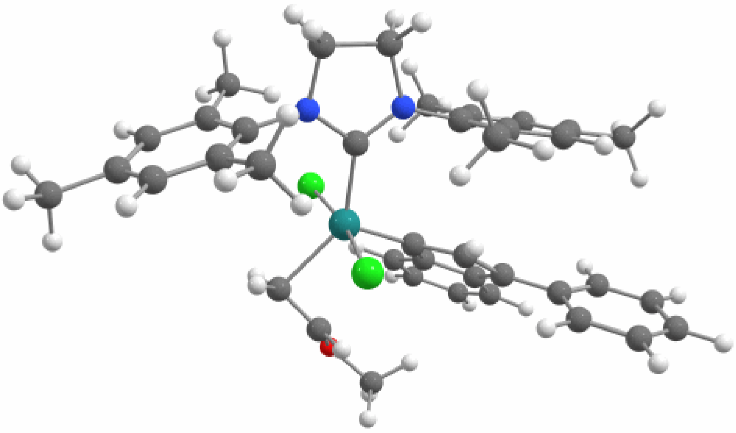

Zero-point correction= 0.690244 (Hartree/Particle)
Thermal correction to Energy= 0.738170
Thermal correction to Enthalpy= 0.739114
Thermal correction to Gibbs Free Energy= 0.613907
Sum of electronic and zero-point Energies= -2709.546396
Sum of electronic and thermal Energies= -2709.498471
Sum of electronic and thermal Enthalpies= -2709.497527
Sum of electronic and thermal Free Energies= -2709.622734

Solvent: -2710.7449141

Ru	-1.066042000	0.638385000	-0.154667000
Cl	-1.783924000	1.155805000	2.078716000
Cl	-0.732840000	0.194810000	-2.539011000
N	-2.720796000	-1.766149000	0.243854000
N	-0.623149000	-2.348826000	0.608736000
C	-1.426404000	-1.307899000	0.250162000
C	-2.829704000	-3.200950000	0.577017000
H	-3.207285000	-3.771815000	-0.300154000
H	-3.544790000	-3.354090000	1.411566000
C	-1.386453000	-3.572032000	0.952480000
H	-1.266918000	-3.796986000	2.035085000
H	-0.999011000	-4.443891000	0.385329000
C	-3.909795000	-1.025862000	-0.105142000
C	-4.740472000	-0.510633000	0.929437000
C	-4.471499000	-0.789812000	2.388903000
H	-4.967175000	-0.036703000	3.032317000
H	-3.390079000	-0.768880000	2.620490000
H	-4.883044000	-1.784367000	2.675727000
C	-5.894146000	0.208889000	0.560097000
H	-6.527246000	0.630115000	1.359104000
C	-6.270171000	0.388701000	-0.785141000
C	-7.487680000	1.207191000	-1.152152000
H	-7.946624000	0.857193000	-2.099109000
H	-7.217837000	2.276817000	-1.296500000
H	-8.260578000	1.172091000	-0.357635000
C	-5.482923000	-0.227199000	-1.777316000
H	-5.795128000	-0.161043000	-2.833278000
C	-4.317595000	-0.958583000	-1.468231000
C	-3.573147000	-1.681554000	-2.563066000
H	-4.173348000	-1.702237000	-3.493857000
H	-3.346382000	-2.731451000	-2.282070000
H	-2.601297000	-1.186570000	-2.784159000
C	0.804787000	-2.406254000	0.760849000
C	1.572539000	-2.949335000	-0.303880000
C	0.930766000	-3.292304000	-1.629173000
H	1.689919000	-3.639770000	-2.357056000
H	0.402785000	-2.415741000	-2.065387000
H	0.173740000	-4.101062000	-1.531018000
C	2.950785000	-3.140345000	-0.098812000
H	3.556062000	-3.558277000	-0.920662000
C	3.576398000	-2.807810000	1.120929000
C	5.059049000	-3.036030000	1.308780000
H	5.377709000	-2.827639000	2.349650000
H	5.650398000	-2.380374000	0.633905000
H	5.340312000	-4.083436000	1.068656000
C	2.783131000	-2.262406000	2.148087000
H	3.254192000	-1.996730000	3.109392000
C	1.394173000	-2.063871000	2.002910000
C	0.569928000	-1.518352000	3.144399000
H	-0.201156000	-2.247518000	3.477149000
H	0.023551000	-0.592762000	2.861656000
H	1.210066000	-1.292690000	4.019457000
H	1.627222000	-0.467150000	-1.517957000
C	1.836940000	0.256455000	-0.726099000
C	3.088935000	0.739230000	-0.392681000
C	0.790918000	0.853863000	0.109029000
C	2.915091000	1.720029000	0.711480000
C	1.516601000	1.791326000	1.029445000
C	3.831137000	2.566185000	1.347458000
C	1.070404000	2.690811000	2.006212000
C	3.369025000	3.464303000	2.341121000
H	4.897008000	2.556223000	1.073706000
C	2.008616000	3.517637000	2.671441000
H	-0.000150000	2.743402000	2.249049000
H	4.087849000	4.126293000	2.850492000
H	1.657405000	4.217449000	3.446452000
C	4.349009000	0.382676000	-1.065029000
C	5.585803000	0.326393000	-0.370125000
C	4.352243000	0.068864000	-2.450652000
C	6.772307000	-0.027189000	-1.033380000
H	5.607694000	0.525861000	0.711405000
C	5.538085000	-0.282341000	-3.110531000
H	3.407881000	0.129912000	-3.013808000
C	6.756022000	-0.330319000	-2.406291000
H	7.718587000	-0.070511000	-0.470260000
H	5.514581000	-0.512120000	-4.187924000
H	7.688675000	-0.600608000	-2.926553000
C	-2.109725000	2.558891000	-0.691770000
C	-0.922003000	3.116277000	-1.149570000
H	-2.731364000	2.032216000	-1.438345000
H	-2.610621000	2.994848000	0.187166000
H	-0.463254000	2.782703000	-2.100582000
O	-0.334549000	4.129812000	-0.499599000
C	0.938103000	4.558469000	-1.006459000
H	0.870649000	4.789945000	-2.091641000
H	1.206568000	5.473691000	-0.448329000
H	1.710817000	3.781950000	-0.830847000

Catalyst 1, III-IV TS

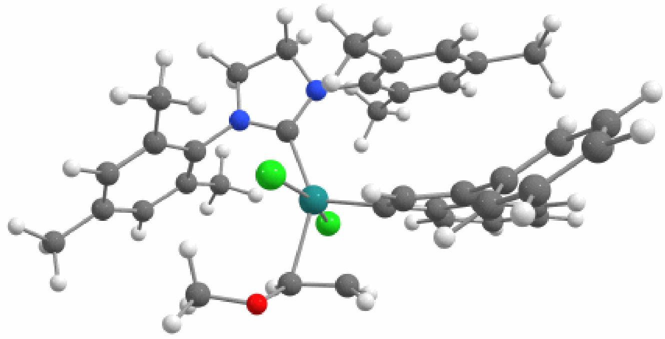

Zero-point correction= 0.689868 (Hartree/Particle)
Thermal correction to Energy= 0.736837
Thermal correction to Enthalpy= 0.737781
Thermal correction to Gibbs Free Energy= 0.615097
Sum of electronic and zero-point Energies= -2709.536632
Sum of electronic and thermal Energies= -2709.489662
Sum of electronic and thermal Enthalpies= -2709.488718
Sum of electronic and thermal Free Energies= -2709.611403

Solvent: -2710.7347563

Ru	-0,789582000	0,276019000	0,690649000
Cl	-1,268136000	-1,384660000	2,408863000
Cl	-0,918905000	1,991353000	-1,030373000
N	-2,765797000	-1,138131000	-1,183417000
N	-0,722615000	-1,949091000	-1,384295000
C	-1,479687000	-1,022602000	-0,737611000
C	-2,937857000	-2,286767000	-2,104478000
H	-3,510800000	-1,983092000	-3,003992000
H	-3,505166000	-3,098744000	-1,597218000
C	-1,488638000	-2,686863000	-2,415291000
H	-1,307687000	-3,777264000	-2,326752000
H	-1,161264000	-2,369863000	-3,430801000
C	-3,918891000	-0,347649000	-0,822244000
C	-4,724531000	-0,732975000	0,285282000
C	-4,431372000	-1,973112000	1,093813000
H	-5,178783000	-2,099795000	1,901457000
H	-3,422022000	-1,941250000	1,557486000
H	-4,472934000	-2,886024000	0,460447000
C	-5,859891000	0,048700000	0,583957000
H	-6,480892000	-0,240798000	1,448636000
C	-6,239989000	1,155657000	-0,199575000
C	-7,445023000	1,992533000	0,168637000
H	-7,863548000	2,519506000	-0,712430000
H	-7,177794000	2,769804000	0,918411000
H	-8,250033000	1,375657000	0,618051000
C	-5,468005000	1,451614000	-1,339981000
H	-5,773964000	2,280731000	-2,000109000
C	-4,319740000	0,710279000	-1,684993000
C	-3,570814000	1,024390000	-2,957007000
H	-4,042288000	1,872356000	-3,491240000
H	-3,560606000	0,157141000	-3,652746000
H	-2,516363000	1,291283000	-2,732154000
C	0,692519000	-2,191563000	-1,261630000
C	1,600839000	-1,444821000	-2,055253000
C	1,129118000	-0,351878000	-2,984651000
H	1,988326000	0,130348000	-3,490193000
H	0,555615000	0,434998000	-2,447589000
H	0,460351000	-0,750284000	-3,779389000
C	2,970820000	-1,766440000	-1,966567000
H	3,685768000	-1,187420000	-2,574873000
C	3,446716000	-2,805985000	-1,144674000
C	4,924441000	-3,110720000	-1,046137000
H	5,114793000	-4,202080000	-0,982709000
H	5,356867000	-2,652590000	-0,129389000
H	5,485192000	-2,712072000	-1,915599000
C	2,508138000	-3,546520000	-0,398861000
H	2,857256000	-4,373331000	0,242091000
C	1,129797000	-3,263527000	-0,439987000
C	0,152188000	-4,076856000	0,374410000
H	-0,613570000	-4,570333000	-0,263171000
H	-0,394049000	-3,433789000	1,098738000
H	0,675995000	-4,872913000	0,939038000
H	1,431437000	2,329119000	-0,454765000
C	1,894887000	1,570464000	0,185494000
C	3,246351000	1,355372000	0,343086000
C	1,101782000	0,556911000	0,915445000
C	3,425333000	0,213878000	1,279315000
C	2,131484000	-0,277127000	1,640849000
C	4,580396000	-0,322506000	1,862094000
C	2,018091000	-1,323416000	2,563040000
C	4,453959000	-1,376937000	2,796738000
H	5,574822000	0,083873000	1,622620000
C	3,187982000	-1,871941000	3,139470000
H	1,022480000	-1,703764000	2,835649000
H	5,357732000	-1,802312000	3,262168000
H	3,096959000	-2,691433000	3,870046000
C	4,317086000	2,126599000	-0,312817000
C	5,520155000	1,510753000	-0,745242000
C	4,151390000	3,514175000	-0,561611000
C	6,515097000	2,253485000	-1,400715000
H	5,656945000	0,429264000	-0,596941000
C	5,149013000	4,254744000	-1,210678000
H	3,230949000	4,012919000	-0,220235000
C	6,336986000	3,628648000	-1,632424000
H	7,436524000	1,751979000	-1,737761000
H	5,001563000	5,332570000	-1,384975000
H	7,121329000	4,211859000	-2,140596000
C	-0,048412000	1,569697000	2,477947000
C	-1,419770000	1,800676000	2,147071000
H	0,172826000	0,855061000	3,284207000
H	0,626750000	2,433276000	2,371780000
H	-2,202223000	1,190510000	2,649612000
O	-1,792608000	3,052149000	1,780529000
C	-3,129458000	3,190978000	1,295506000
H	-3,852789000	2,669668000	1,963157000
H	-3,353134000	4,274339000	1,284365000
H	-3,204487000	2,781559000	0,266167000

Catalyst 1, IV intermediate

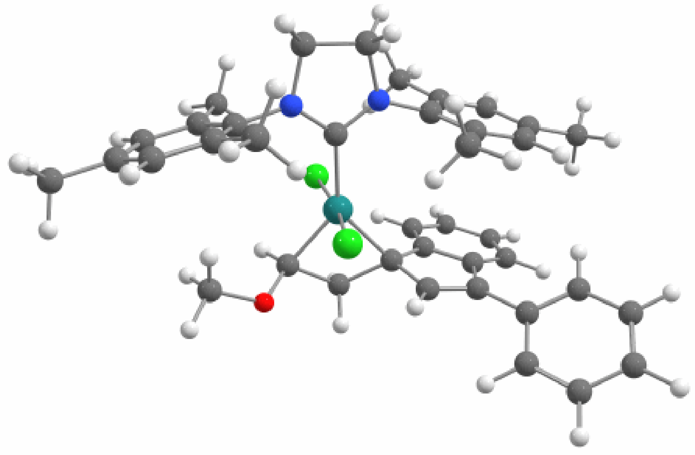

Zero-point correction= 0.691594 (Hartree/Particle)
Thermal correction to Energy= 0.738637
Thermal correction to Enthalpy= 0.739581
Thermal correction to Gibbs Free Energy= 0.616466
Sum of electronic and zero-point Energies= -2709.546034
Sum of electronic and thermal Energies= -2709.498991
Sum of electronic and thermal Enthalpies= -2709.498047
Sum of electronic and thermal Free Energies= -2709.621163

Solvent: -2710.7447535

Ru	0.758535	-0.260147	0.655605
Cl	1.182900	1.459251	2.320892
Cl	0.760219	-1.974251	-1.070408
N	2.743804	1.121619	-1.200978
N	0.681282	1.887760	-1.433964
C	1.456799	0.989415	-0.774258
C	2.898264	2.269336	-2.127979
H	3.491945	1.971694	-3.015742
H	3.437766	3.097580	-1.617243
C	1.443925	2.631251	-2.463520
H	1.234957	3.717368	-2.388588
H	1.137826	2.293863	-3.478822
C	3.911205	0.352926	-0.838053
C	4.721644	0.768423	0.252646
C	4.401178	1.998946	1.065164
H	5.133993	2.128587	1.885583
H	3.383923	1.953620	1.510893
H	4.444021	2.917480	0.439531
C	5.885924	0.021918	0.534959
H	6.513098	0.334085	1.387006
C	6.284567	-1.077428	-0.247987
C	7.528762	-1.870122	0.086396
H	8.125359	-2.093940	-0.822312
H	7.270059	-2.848246	0.548224
H	8.179997	-1.328882	0.801450
C	5.493659	-1.413215	-1.365856
H	5.806501	-2.243472	-2.021377
C	4.317547	-0.710231	-1.692918
C	3.535210	-1.074442	-2.930296
H	4.034424	-1.892665	-3.485102
H	3.434906	-0.211902	-3.624204
H	2.509148	-1.404002	-2.657232
C	-0.740858	2.106305	-1.309187
C	-1.636613	1.352681	-2.112364
C	-1.153899	0.275011	-3.052229
H	-2.003229	-0.168211	-3.607375
H	-0.627436	-0.541683	-2.509996
H	-0.438387	0.674386	-3.804146
C	-3.011339	1.656430	-2.027266
H	-3.714784	1.070661	-2.642136
C	-3.504237	2.686999	-1.205220
C	-4.985004	2.977874	-1.115429
H	-5.188130	4.068964	-1.106874
H	-5.407373	2.563895	-0.173525
H	-5.544412	2.529484	-1.960943
C	-2.578000	3.440805	-0.457224
H	-2.939335	4.267389	0.177069
C	-1.195606	3.179661	-0.495190
C	-0.237785	4.030016	0.303550
H	0.548037	4.488013	-0.335674
H	0.283314	3.422552	1.075508
H	-0.775827	4.854242	0.811243
H	-1.402142	-2.615683	-0.007760
C	-1.867267	-1.799343	0.556524
C	-3.193359	-1.439674	0.517503
C	-1.077166	-0.877548	1.416362
C	-3.380774	-0.287249	1.433659
C	-2.112328	0.058208	1.994198
C	-4.547855	0.362503	1.867030
C	-2.027217	1.058885	2.971314
C	-4.450533	1.373580	2.846871
H	-5.533965	0.065468	1.478984
C	-3.202863	1.718664	3.391447
H	-1.049075	1.344309	3.385074
H	-5.363039	1.884889	3.193968
H	-3.136205	2.506789	4.158325
C	-4.239266	-2.109383	-0.280693
C	-5.310639	-1.383840	-0.861620
C	-4.181712	-3.507558	-0.515812
C	-6.280130	-2.029148	-1.646712
H	-5.361286	-0.293823	-0.723359
C	-5.151240	-4.150813	-1.298352
H	-3.369289	-4.094110	-0.059151
C	-6.207745	-3.415445	-1.867171
H	-7.097665	-1.441352	-2.094589
H	-5.086228	-5.238731	-1.460730
H	-6.971391	-3.921943	-2.478675
C	-0.006307	-1.518934	2.399270
C	1.522910	-1.529864	1.998519
H	-0.088928	-0.999867	3.371580
H	-0.259890	-2.594489	2.466598
H	2.173459	-0.982190	2.719684
O	1.964034	-2.797282	1.734266
C	3.366167	-2.920094	1.511559
H	3.947377	-2.391304	2.302016
H	3.602329	-4.000680	1.540774
H	3.641034	-2.507118	0.517861

Catalyst 1, IV-V TS

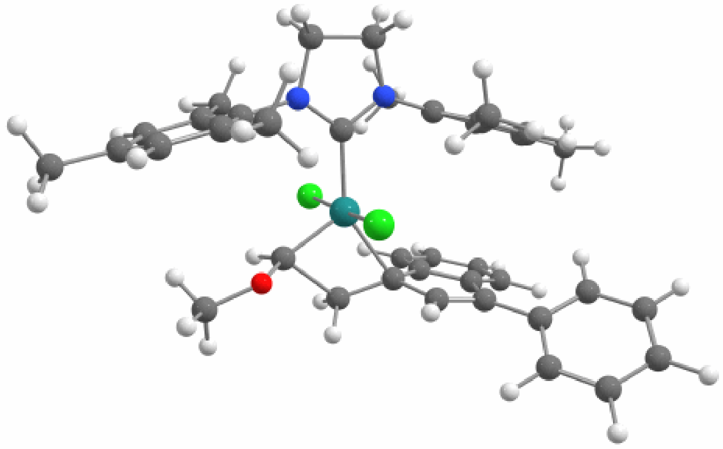

Zero-point correction= 0.689853 (Hartree/Particle)
Thermal correction to Energy= 0.736971
Thermal correction to Enthalpy= 0.737915
Thermal correction to Gibbs Free Energy= 0.612564
Sum of electronic and zero-point Energies= -2709.542985
Sum of electronic and thermal Energies= -2709.495867
Sum of electronic and thermal Enthalpies= -2709.494923
Sum of electronic and thermal Free Energies= -2709.620274

Solvent: -2710.7398402

Ru	0.701906	-0.334873	0.504050
Cl	1.236032	1.039859	2.439904
Cl	0.404556	-1.663298	-1.506102
N	2.786462	1.200816	-1.159508
N	0.755645	2.049459	-1.352125
C	1.493386	1.077746	-0.755721
C	2.985162	2.365039	-2.057106
H	3.494314	2.048011	-2.990681
H	3.631305	3.122528	-1.561629
C	1.549931	2.870220	-2.294429
H	1.424300	3.949245	-2.067276
H	1.201870	2.699961	-3.336268
C	3.929175	0.404136	-0.781539
C	4.711156	0.795542	0.338244
C	4.358920	2.003480	1.170797
H	5.140822	2.199432	1.930475
H	3.387844	1.860495	1.694763
H	4.260147	2.918935	0.549022
C	5.849661	0.025250	0.654772
H	6.454739	0.319900	1.528987
C	6.246308	-1.082665	-0.119628
C	7.499856	-1.860969	0.216784
H	8.380971	-1.450730	-0.324776
H	7.411349	-2.927721	-0.073818
H	7.734600	-1.815055	1.299565
C	5.473985	-1.408008	-1.251905
H	5.779000	-2.254055	-1.890810
C	4.324950	-0.676524	-1.615350
C	3.558851	-1.037713	-2.862943
H	4.063994	-1.856226	-3.412206
H	3.470272	-0.173608	-3.556485
H	2.523080	-1.360485	-2.614501
C	-0.647988	2.320035	-1.148475
C	-1.605134	1.791206	-2.055513
C	-1.203656	0.938939	-3.234945
H	-2.098498	0.549986	-3.758841
H	-0.583068	0.072430	-2.919583
H	-0.617615	1.523819	-3.978365
C	-2.961325	2.116758	-1.846145
H	-3.712551	1.699847	-2.537343
C	-3.378868	2.962093	-0.801223
C	-4.841178	3.274044	-0.578200
H	-5.005250	4.357718	-0.401378
H	-5.221221	2.740478	0.320299
H	-5.464403	2.968075	-1.442163
C	-2.393493	3.514578	0.040866
H	-2.694949	4.205835	0.845473
C	-1.025280	3.225322	-0.117415
C	-0.002194	3.883092	0.776494
H	0.831734	4.329375	0.194086
H	0.451354	3.147509	1.477342
H	-0.467834	4.690981	1.374101
H	-1.556438	-2.718694	-0.289620
C	-1.960190	-1.961518	0.391451
C	-3.261867	-1.522893	0.436661
C	-1.112303	-1.228077	1.361734
C	-3.361729	-0.494406	1.500727
C	-2.067049	-0.309079	2.078777
C	-4.480475	0.158283	2.043068
C	-1.909586	0.527506	3.191727
C	-4.308755	1.009800	3.155168
H	-5.486705	-0.019550	1.633742
C	-3.036593	1.191172	3.723047
H	-0.912620	0.684083	3.627390
H	-5.182786	1.522429	3.588647
H	-2.912791	1.852149	4.595631
C	-4.367475	-2.026111	-0.401791
C	-5.383775	-1.163977	-0.886889
C	-4.426949	-3.395148	-0.767939
C	-6.414348	-1.652606	-1.706369
H	-5.340703	-0.091195	-0.645916
C	-5.457452	-3.881839	-1.584977
H	-3.657826	-4.084146	-0.385055
C	-6.458884	-3.013277	-2.056960
H	-7.188073	-0.961944	-2.078961
H	-5.484352	-4.951011	-1.850233
H	-7.271262	-3.396464	-2.694826
C	0.044799	-1.895296	2.009659
C	1.787853	-1.655519	1.326771
H	0.200723	-1.603156	3.062945
H	0.064048	-2.983473	1.824747
H	2.354943	-1.354593	2.240917
O	2.268637	-2.768060	0.741258
C	3.288878	-3.475799	1.462511
H	2.878356	-3.931514	2.390702
H	3.647624	-4.277652	0.790983
H	4.135903	-2.803448	1.716670

Catalyst 1, V intermediate

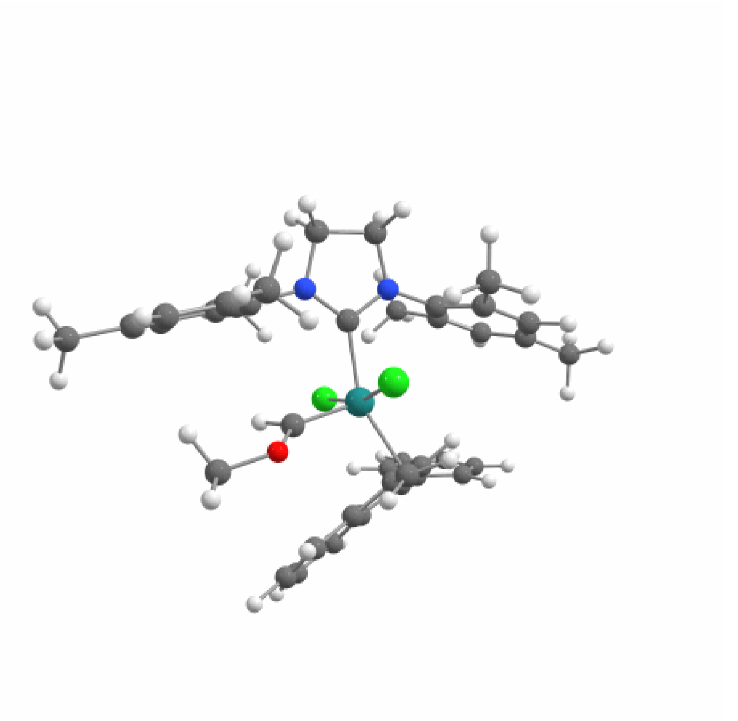

Zero-point correction= 0.690166 (Hartree/Particle)
Thermal correction to Energy= 0.737947
Thermal correction to Enthalpy= 0.738891
Thermal correction to Gibbs Free Energy= 0.612372
Sum of electronic and zero-point Energies= -2709.559583
Sum of electronic and thermal Energies= -2709.511802
Sum of electronic and thermal Enthalpies= -2709.510858
Sum of electronic and thermal Free Energies= -2709.637377

Solvent: -2710.7561014

Ru	0.414550	0.045266	-0.786195
Cl	-0.359994	-0.935171	1.296584
Cl	1.570486	1.308072	-2.541440
N	2.635510	1.426596	0.993386
N	0.831151	2.645511	0.642629
C	1.410278	1.436301	0.405824
C	2.945464	2.701307	1.687435
H	3.885674	3.134321	1.286607
H	3.095854	2.516553	2.772710
C	1.709502	3.571338	1.391264
H	1.198425	3.929367	2.309704
H	1.950403	4.459068	0.768827
C	3.601436	0.360117	1.083593
C	3.564571	-0.512114	2.207560
C	2.498604	-0.392688	3.268369
H	2.668742	-1.124107	4.082385
H	1.487837	-0.570561	2.838384
H	2.484729	0.619614	3.727051
C	4.565054	-1.497995	2.316137
H	4.533123	-2.182817	3.180349
C	5.607923	-1.618954	1.374952
C	6.670622	-2.684188	1.533126
H	7.432538	-2.627161	0.730498
H	6.229038	-3.703745	1.511045
H	7.196284	-2.587380	2.507036
C	5.636696	-0.707930	0.301705
H	6.458415	-0.762616	-0.432212
C	4.657556	0.296545	0.137269
C	4.764650	1.283096	-0.999253
H	5.610731	1.024806	-1.665785
H	4.946550	2.314215	-0.622902
H	3.831534	1.319696	-1.603186
C	-0.500555	3.053738	0.256061
C	-0.689165	3.860299	-0.900706
C	0.467125	4.400171	-1.707284
H	0.120157	4.764726	-2.694111
H	1.242919	3.628443	-1.883887
H	0.933916	5.267817	-1.188430
C	-2.007430	4.224950	-1.245611
H	-2.161141	4.829988	-2.154893
C	-3.118978	3.865598	-0.460527
C	-4.524888	4.240618	-0.872330
H	-5.119557	4.603126	-0.008328
H	-5.065689	3.361980	-1.287163
H	-4.528463	5.029419	-1.650915
C	-2.881461	3.159540	0.735722
H	-3.727851	2.921278	1.401124
C	-1.588177	2.763912	1.130882
C	-1.379169	2.108515	2.473169
H	-0.597700	2.632099	3.064454
H	-1.055358	1.050459	2.360675
H	-2.315981	2.123702	3.063272
H	-2.914320	0.427650	-1.037349
C	-2.630908	-0.634681	-1.020759
C	-3.274387	-1.628864	-0.309765
C	-1.443635	-1.173640	-1.694596
C	-2.549228	-2.899222	-0.540617
C	-1.436726	-2.628289	-1.393092
C	-2.830871	-4.222369	-0.153280
C	-0.619578	-3.668159	-1.855687
C	-1.992076	-5.260005	-0.603215
H	-3.704120	-4.451669	0.476769
C	-0.896941	-4.988638	-1.448255
H	0.219596	-3.460049	-2.539048
H	-2.204292	-6.298914	-0.303422
H	-0.264209	-5.816806	-1.806190
C	-4.483274	-1.456239	0.517688
C	-4.615335	-2.105451	1.771594
C	-5.539312	-0.612017	0.090107
C	-5.757078	-1.912912	2.565745
H	-3.790853	-2.735530	2.138508
C	-6.680436	-0.422215	0.884577
H	-5.464260	-0.120869	-0.893136
C	-6.796255	-1.073222	2.126405
H	-5.832816	-2.419381	3.541608
H	-7.492647	0.231200	0.526623
H	-7.694252	-0.928347	2.748064
C	-0.661637	-0.490600	-2.649089
C	1.672503	-1.293096	-0.815459
H	0.020213	-1.042701	-3.314680
H	-1.022101	0.469205	-3.053747
H	1.832570	-1.928309	0.091617
O	2.391665	-1.665890	-1.869067
C	3.271149	-2.802313	-1.704565
H	2.698310	-3.690785	-1.366924
H	3.713157	-2.996221	-2.699045
H	4.072564	-2.560855	-0.976990

Catalyst 1, V-VI TS

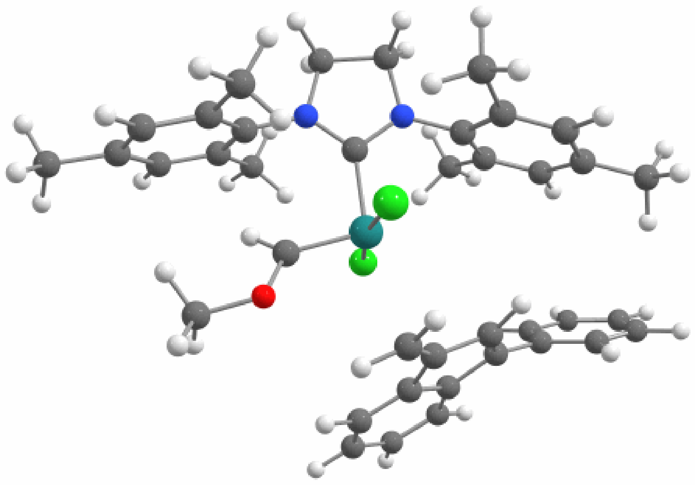

Zero-point correction= 0.688883 (Hartree/Particle)
Thermal correction to Energy= 0.736909
Thermal correction to Enthalpy= 0.737853
Thermal correction to Gibbs Free Energy= 0.609333
Sum of electronic and zero-point Energies= -2709.550257
Sum of electronic and thermal Energies= -2709.502232
Sum of electronic and thermal Enthalpies= -2709.501287
Sum of electronic and thermal Free Energies= -2709.629807

Solvent: -2710.7473982

Ru	-0,741361000	0,094204000	-0,640940000
Cl	0,117879000	1,196961000	1,315294000
Cl	-1,172061000	-1,054400000	-2,698249000
N	-2,844914000	-1,426649000	0,990610000
N	-0,906114000	-2,458152000	0,744530000
C	-1,598227000	-1,308853000	0,427350000
C	-2,996550000	-2,650310000	1,804983000
H	-3,932832000	-3,182409000	1,536335000
H	-3,055963000	-2,391448000	2,886282000
C	-1,729254000	-3,449464000	1,461524000
H	-1,196662000	-3,824090000	2,360289000
H	-1,944975000	-4,323146000	0,806855000
C	-3,953953000	-0,520329000	0,917241000
C	-4,155238000	0,423374000	1,959785000
C	-3,161086000	0,565563000	3,087975000
H	-3,455390000	1,384516000	3,773073000
H	-2,139250000	0,781439000	2,702069000
H	-3,085973000	-0,362814000	3,694468000
C	-5,285905000	1,261857000	1,885644000
H	-5,445049000	2,003004000	2,687190000
C	-6,211031000	1,181667000	0,824011000
C	-7,438985000	2,065407000	0,798049000
H	-7,825411000	2,203338000	-0,231942000
H	-7,233085000	3,066860000	1,228251000
H	-8,262361000	1,619147000	1,398591000
C	-5,977125000	0,231490000	-0,191070000
H	-6,688420000	0,150899000	-1,030559000
C	-4,864035000	-0,634952000	-0,165679000
C	-4,638465000	-1,639090000	-1,270465000
H	-5,480874000	-1,633221000	-1,989579000
H	-4,541621000	-2,671551000	-0,871217000
H	-3,698839000	-1,429008000	-1,828686000
C	0,434784000	-2,801869000	0,338615000
C	0,639679000	-3,598296000	-0,821851000
C	-0,507411000	-4,162409000	-1,626253000
H	-0,169790000	-4,454548000	-2,639909000
H	-1,327537000	-3,429393000	-1,749009000
H	-0,911542000	-5,079898000	-1,141276000
C	1,964125000	-3,927637000	-1,176779000
H	2,128850000	-4,524009000	-2,090050000
C	3,070512000	-3,536426000	-0,400294000
C	4,481239000	-3,912936000	-0,794716000
H	4,893289000	-4,690138000	-0,114316000
H	5,162696000	-3,039071000	-0,731104000
H	4,525636000	-4,314874000	-1,826647000
C	2,824204000	-2,821530000	0,789067000
H	3,671267000	-2,542740000	1,437747000
C	1,523329000	-2,464643000	1,194275000
C	1,304732000	-1,791409000	2,526048000
H	0,545213000	-2,328856000	3,133362000
H	0,940270000	-0,748755000	2,395540000
H	2,246646000	-1,762417000	3,107876000
H	2,928745000	-0,142583000	-1,678751000
C	2,838199000	0,881850000	-1,291715000
C	3,686691000	1,481361000	-0,384926000
C	1,732244000	1,788251000	-1,626668000
C	3,171948000	2,847790000	-0,100383000
C	1,973869000	3,030876000	-0,850459000
C	3,684655000	3,898347000	0,674844000
C	1,279921000	4,243049000	-0,808314000
C	2,976260000	5,119001000	0,719869000
H	4,628040000	3,786423000	1,231122000
C	1,785036000	5,290288000	-0,007774000
H	0,355033000	4,383160000	-1,390265000
H	3,366346000	5,949485000	1,329967000
H	1,247130000	6,250335000	0,041476000
C	4,901354000	0,879775000	0,195937000
C	5,220551000	1,044813000	1,567908000
C	5,774173000	0,100368000	-0,605097000
C	6,365550000	0,445188000	2,117020000
H	4,540123000	1,620506000	2,213828000
C	6,922398000	-0,490993000	-0,055665000
H	5,553371000	-0,015577000	-1,677934000
C	7,223286000	-0,321921000	1,308583000
H	6,588349000	0,576175000	3,188205000
H	7,594866000	-1,080191000	-0,699900000
H	8,126342000	-0,783460000	1,738894000
C	0,721343000	1,547479000	-2,519551000
C	-2,151028000	1,255002000	-0,764173000
H	-0,046058000	2,305518000	-2,734075000
H	0,700071000	0,634058000	-3,130864000
H	-2,870748000	1,466898000	0,060405000
O	-2,372346000	2,019077000	-1,845147000
C	-3,427779000	2,999501000	-1,766997000
H	-3,101522000	3,869885000	-1,159584000
H	-3,632070000	3,325963000	-2,803618000
H	-4,343438000	2,553686000	-1,325127000

Catalyst 1, VI intermediate

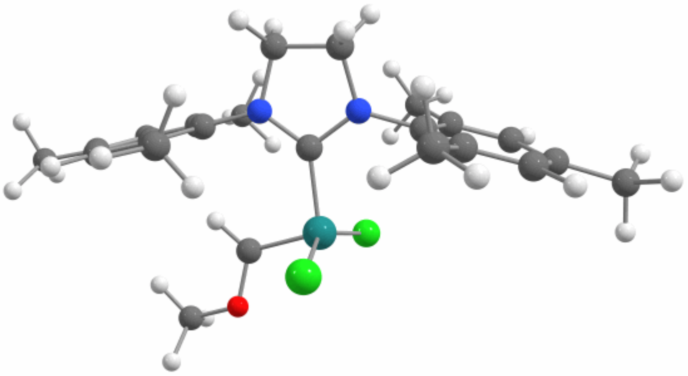

Zero-point correction= 0.467227 (Hartree/Particle)
Thermal correction to Energy= 0.502158
Thermal correction to Enthalpy= 0.503103
Thermal correction to Gibbs Free Energy= 0.403372
Sum of electronic and zero-point Energies= -2093.309243
Sum of electronic and thermal Energies= -2093.274312
Sum of electronic and thermal Enthalpies= -2093.273368
Sum of electronic and thermal Free Energies= -2093.373099

Solvent: -2094.1305286

Ru	-0.226657000	1.234456000	0.175649000
Cl	-1.052062000	1.944157000	-1.899760000
Cl	-0.257273000	1.337230000	2.521771000
N	0.944143000	-1.561156000	0.027095000
N	-1.268234000	-1.451835000	0.055983000
C	-0.118770000	-0.682092000	0.036831000
C	0.513413000	-2.970206000	-0.078000000
H	1.070106000	-3.604086000	0.642444000
H	0.715847000	-3.365908000	-1.100513000
C	-0.989176000	-2.886779000	0.230965000
H	-1.609788000	-3.495441000	-0.458668000
H	-1.220433000	-3.209890000	1.271706000
C	2.343431000	-1.266048000	-0.072247000
C	2.932370000	-1.103347000	-1.353978000
C	2.084676000	-1.110240000	-2.605381000
H	2.707689000	-0.963396000	-3.509090000
H	1.322759000	-0.301476000	-2.573514000
H	1.530655000	-2.064463000	-2.731715000
C	4.320260000	-0.867794000	-1.424707000
H	4.784634000	-0.734834000	-2.416620000
C	5.125086000	-0.792082000	-0.268744000
C	6.619680000	-0.582424000	-0.374846000
H	7.153601000	-1.556421000	-0.439030000
H	7.023666000	-0.049717000	0.509843000
H	6.890404000	-0.004277000	-1.281564000
C	4.502570000	-0.948331000	0.986584000
H	5.112573000	-0.882751000	1.903338000
C	3.119273000	-1.190917000	1.113253000
C	2.476656000	-1.331375000	2.472274000
H	3.236849000	-1.292913000	3.276821000
H	1.927861000	-2.292229000	2.570741000
H	1.733502000	-0.522994000	2.651397000
C	-2.625177000	-0.972618000	-0.015262000
C	-3.378565000	-0.772523000	1.174197000
C	-2.814477000	-1.079437000	2.541334000
H	-3.497828000	-0.719974000	3.335539000
H	-1.827653000	-0.597886000	2.700812000
H	-2.691200000	-2.175485000	2.689134000
C	-4.708753000	-0.322496000	1.046893000
H	-5.293440000	-0.147942000	1.965824000
C	-5.313954000	-0.106515000	-0.205752000
C	-6.726737000	0.422350000	-0.308756000
H	-7.252096000	0.018768000	-1.198427000
H	-6.726140000	1.530474000	-0.406854000
H	-7.325589000	0.174256000	0.590851000
C	-4.561562000	-0.390641000	-1.362646000
H	-5.028513000	-0.269297000	-2.354518000
C	-3.227246000	-0.832736000	-1.296542000
C	-2.468384000	-1.151388000	-2.561978000
H	-1.895212000	-2.097981000	-2.477672000
H	-1.742411000	-0.340611000	-2.785759000
H	-3.158915000	-1.241121000	-3.423518000
C	1.519350000	1.720056000	-0.076019000
H	2.370535000	1.087516000	-0.417393000
O	1.854625000	3.000540000	0.135326000
C	3.178637000	3.448751000	-0.204797000
H	3.162720000	3.922712000	-1.208210000
H	3.478590000	4.197774000	0.552613000
H	3.894880000	2.599558000	-0.200919000

Catalyst 2, I intermediate

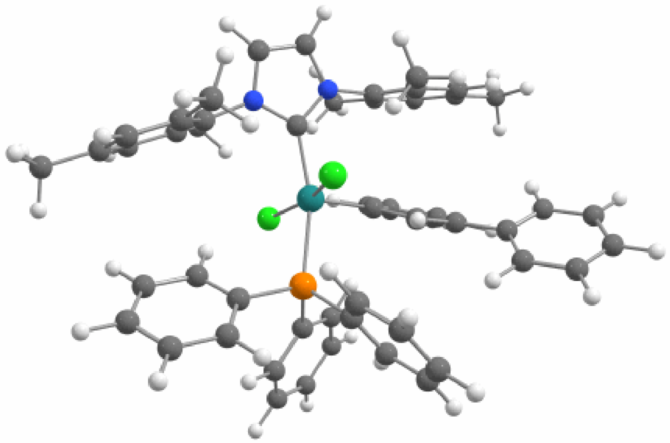

Zero-point correction= 0.850903 (Hartree/Particle)
Thermal correction to Energy= 0.909319
Thermal correction to Enthalpy= 0.910263
Thermal correction to Gibbs Free Energy= 0.753710
Sum of electronic and zero-point Energies= -3550.951160
Sum of electronic and thermal Energies= -3550.892744
Sum of electronic and thermal Enthalpies= -3550.891800
Sum of electronic and thermal Free Energies= -3551.048353

Solvent: -3552.45828261

Ru	-0,606527000	-0,302442000	-0,034251000
Cl	-1,453507000	-0,606023000	2,202316000
Cl	-0,490207000	0,040267000	-2,434251000
P	-1,031675000	2,091096000	0,208545000
N	-2,042880000	-2,941310000	-0,811900000
N	0,100338000	-3,309675000	-0,748127000
C	-0,841049000	-2,329581000	-0,486941000
C	-1,841590000	-4,245770000	-1,271194000
H	-2,680172000	-4,879415000	-1,575892000
C	-0,498569000	-4,477554000	-1,228036000
H	0,095748000	-5,360274000	-1,483255000
C	-3,398603000	-2,446656000	-0,651802000
C	-4,071428000	-2,721639000	0,568807000
C	-3,386977000	-3,435284000	1,708052000
H	-4,108296000	-3,669395000	2,514985000
H	-2,580427000	-2,798214000	2,131714000
H	-2,922080000	-4,387015000	1,373579000
C	-5,425556000	-2,347345000	0,670919000
H	-5,956975000	-2,557837000	1,614278000
C	-6,124432000	-1,755650000	-0,400507000
C	-7,580721000	-1,371764000	-0,258129000
H	-8,087293000	-1,315139000	-1,242541000
H	-7,690460000	-0,374553000	0,222862000
H	-8,133679000	-2,095527000	0,375225000
C	-5,433310000	-1,550845000	-1,609474000
H	-5,970312000	-1,126943000	-2,474233000
C	-4,077259000	-1,903964000	-1,773346000
C	-3,413952000	-1,764152000	-3,120913000
H	-4,126788000	-1,364573000	-3,868260000
H	-3,044690000	-2,744201000	-3,492952000
H	-2,529538000	-1,091869000	-3,075862000
C	1,516280000	-3,314100000	-0,447549000
C	2,448691000	-2,954485000	-1,448114000
C	2,005941000	-2,508024000	-2,818845000
H	2,869165000	-2,154950000	-3,415874000
H	1,252990000	-1,692373000	-2,766324000
H	1,530558000	-3,344569000	-3,377360000
C	3,818766000	-3,072274000	-1,131324000
H	4,559490000	-2,782831000	-1,895150000
C	4,263906000	-3,558306000	0,112406000
C	5,737725000	-3,660273000	0,434408000
H	5,999566000	-4,662681000	0,834248000
H	6,022070000	-2,920566000	1,214398000
H	6,367813000	-3,472028000	-0,457630000
C	3,297317000	-3,944835000	1,063527000
H	3,624276000	-4,338427000	2,040171000
C	1,919052000	-3,836283000	0,807999000
C	0,901307000	-4,286306000	1,829917000
H	0,360183000	-5,196702000	1,490540000
H	0,132291000	-3,507807000	2,013238000
H	1,389128000	-4,525313000	2,794588000
H	1,826658000	1,030415000	-1,587748000
C	2,145125000	0,634583000	-0,616352000
C	3,441161000	0,625578000	-0,133762000
C	1,234253000	-0,078787000	0,289659000
C	3,426760000	-0,041658000	1,195856000
C	2,089941000	-0,486249000	1,456750000
C	4,426362000	-0,182518000	2,166476000
C	1,780933000	-1,075760000	2,688368000
C	4,104985000	-0,792591000	3,402863000
H	5,443515000	0,200525000	1,991478000
C	2,797892000	-1,231277000	3,659883000
H	0,745943000	-1,379934000	2,900467000
H	4,886043000	-0,905472000	4,172128000
H	2,553584000	-1,691125000	4,630869000
C	4,619726000	1,169886000	-0,826330000
C	5,895086000	0,555018000	-0,721697000
C	4,498227000	2,317972000	-1,653029000
C	7,002062000	1,067625000	-1,416147000
H	6,006310000	-0,358302000	-0,118659000
C	5,607429000	2,830527000	-2,340086000
H	3,522151000	2,821450000	-1,731161000
C	6,865439000	2,209621000	-2,224962000
H	7,979978000	0,567283000	-1,328888000
H	5,490895000	3,728141000	-2,968303000
H	7,736316000	2,615021000	-2,764378000
C	-2,815586000	2,435671000	-0,174342000
C	-3,245411000	3,537552000	-0,946519000
C	-3,781129000	1,578970000	0,403697000
C	-4,617808000	3,773893000	-1,142951000
H	-2,507980000	4,222747000	-1,390414000
C	-5,150030000	1,828829000	0,213244000
H	-3,456814000	0,725154000	1,022373000
C	-5,573224000	2,922293000	-0,562385000
H	-4,939248000	4,636700000	-1,748296000
H	-5,888486000	1,158614000	0,679238000
H	-6,648001000	3,113354000	-0,712463000
C	-0,085030000	3,293661000	-0,845740000
C	0,852335000	4,179194000	-0,265891000

C	-0,254641000	3,305146000	-2,252038000
C	1,596474000	5,060634000	-1,069894000
H	1,002362000	4,187245000	0,823845000
C	0,488613000	4,192547000	-3,049403000
H	-0,957164000	2,604434000	-2,724335000
C	1,415021000	5,073226000	-2,463673000
H	2,318874000	5,745285000	-0,597502000
H	0,340940000	4,190230000	-4,141270000
H	1,992596000	5,769760000	-3,092502000
C	-0,846829000	2,834237000	1,903148000
C	-1,689621000	3,879023000	2,342689000
C	0,185049000	2,379699000	2,751537000
C	-1,495628000	4,463119000	3,606203000
H	-2,505758000	4,239430000	1,697521000
C	0,381391000	2,972019000	4,010938000
H	0,832765000	1,550770000	2,431899000
C	-0,458687000	4,013053000	4,442311000
H	-2,162295000	5,275137000	3,938387000
H	1,190163000	2,603871000	4,661742000
H	-0,309726000	4,470319000	5,433729000

Catalyst 2, I-II TS

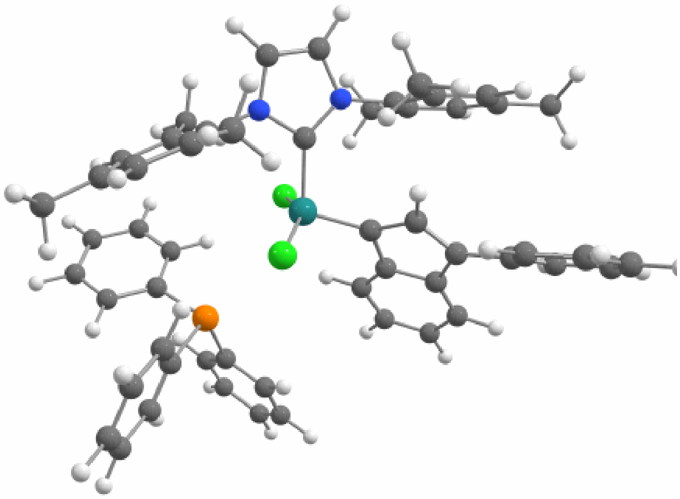

Zero-point correction= 0.849398 (Hartree/Particle)
Thermal correction to Energy= 0.908921
Thermal correction to Enthalpy= 0.909865
Thermal correction to Gibbs Free Energy= 0.745793
Sum of electronic and zero-point Energies= -3550.927093
Sum of electronic and thermal Energies= -3550.867569
Sum of electronic and thermal Enthalpies= -3550.866625
Sum of electronic and thermal Free Energies= -3551.030698

Solvent: -3552.41819005

Ru	-0.377922	-0.513674	-0.130007
Cl	0.310353	-0.378124	-2.364619
Cl	0.135782	-0.464589	2.143741
P	3.351831	1.285748	-0.013705
N	-0.440616	-3.442233	-0.226256
N	-2.409745	-2.736383	-0.878013
C	-1.193658	-2.275325	-0.371723
C	-1.159329	-4.565313	-0.635618
H	-0.723823	-5.568462	-0.594636
C	-2.386284	-4.127643	-1.036987
H	-3.256636	-4.666460	-1.423668
C	0.892317	-3.548343	0.329975
C	2.012177	-3.568006	-0.540051
C	1.858642	-3.552540	-2.041951
H	2.850268	-3.527190	-2.533421
H	1.279885	-2.673679	-2.393746
H	1.325463	-4.461089	-2.398088
C	3.291007	-3.673119	0.045635
H	4.172598	-3.662506	-0.615960
C	3.471508	-3.805895	1.435500
C	4.855807	-3.866758	2.039557
H	4.890460	-4.540935	2.919940
H	5.174107	-2.859176	2.386544
H	5.609772	-4.213173	1.304714
C	2.324112	-3.873598	2.252218
H	2.441247	-4.025346	3.338317
C	1.024080	-3.757141	1.727444
C	-0.183694	-3.869492	2.626477
H	0.113711	-4.185405	3.645414
H	-0.920446	-4.604611	2.239101
H	-0.695678	-2.887685	2.710126
C	-3.651260	-2.028443	-1.108032
C	-4.643343	-2.091492	-0.094988
C	-4.388623	-2.808183	1.213107
H	-5.182855	-2.578794	1.949876
H	-3.410785	-2.528212	1.658176
H	-4.364729	-3.911569	1.079189
C	-5.881284	-1.470452	-0.339642
H	-6.652332	-1.497353	0.448229
C	-6.155811	-0.809842	-1.554734
C	-7.481704	-0.116317	-1.770845
H	-8.332953	-0.758235	-1.461493
H	-7.629677	0.165055	-2.832434
H	-7.542500	0.813189	-1.163978
C	-5.160392	-0.811302	-2.549528
H	-5.367352	-0.329075	-3.519478
C	-3.904233	-1.426420	-2.363285
C	-2.896385	-1.464238	-3.483804
H	-2.563083	-2.503064	-3.693426
H	-1.979553	-0.890117	-3.234074
H	-3.327858	-1.047588	-4.414532
H	-3.397366	-0.305454	1.223232
C	-3.058325	0.587814	0.691861
C	-3.698835	1.817784	0.724093
C	-1.824214	0.667249	-0.071607
C	-2.889563	2.775358	-0.075319
C	-1.733840	2.077339	-0.556496
C	-3.052643	4.138037	-0.350739
C	-0.779425	2.739730	-1.338967
C	-2.079577	4.802659	-1.136907
H	-3.913200	4.699240	0.044183
C	-0.962882	4.112758	-1.630540
H	0.097298	2.198046	-1.724302
H	-2.204908	5.874560	-1.359172
H	-0.213321	4.640739	-2.240223
C	-4.939496	2.115822	1.456902
C	-5.923495	2.993531	0.929886
C	-5.197081	1.504532	2.712447
C	-7.114781	3.244915	1.629255
H	-5.764266	3.454302	-0.056610
C	-6.385328	1.761842	3.410859
H	-4.432468	0.844965	3.151963
C	-7.350654	2.634070	2.873805

H	-7.867739	3.922902	1.196060
H	-6.556185	1.286362	4.390098
H	-8.281913	2.839184	3.425356
C	4.196417	1.385351	1.641151
C	5.218616	2.312245	1.950316
C	3.763448	0.483331	2.640885
C	5.805645	2.325670	3.227691
H	5.553565	3.032069	1.186452
C	4.357528	0.495894	3.915689
H	2.937622	-0.214097	2.423403
C	5.379256	1.415387	4.211858
H	6.600413	3.054349	3.456226
H	4.005540	-0.207535	4.687470
H	5.837157	1.430830	5.214134
C	3.629826	3.005033	-0.672162
C	4.548417	3.334525	-1.693532
C	2.814101	4.031070	-0.135686
C	4.653442	4.658103	-2.160123
H	5.188318	2.551129	-2.128810
C	2.931387	5.353639	-0.592252
H	2.079030	3.788798	0.649974
C	3.850246	5.671511	-1.609994
H	5.374858	4.897455	-2.958353
H	2.292907	6.139157	-0.156860
H	3.936604	6.707417	-1.975430
C	4.554050	0.283902	-1.019747
C	5.837457	-0.104211	-0.574346
C	4.118774	-0.145536	-2.297031
C	6.670112	-0.891220	-1.391074
H	6.191878	0.212671	0.418987
C	4.959249	-0.917164	-3.117336
H	3.104965	0.116283	-2.642551
C	6.237456	-1.295357	-2.666255
H	7.668510	-1.184693	-1.027611
H	4.607439	-1.233262	-4.112780
H	6.893725	-1.906545	-3.306678

Catalyst 2, II intermediate

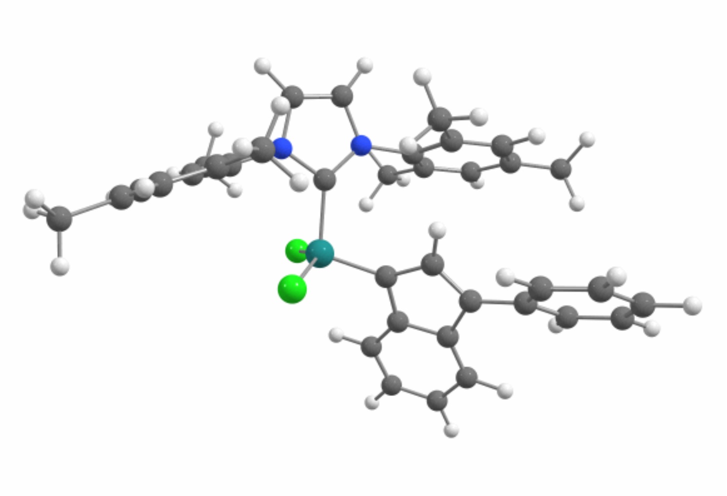

Zero-point correction= 0.582620 (Hartree/Particle)
Thermal correction to Energy= 0.624599
Thermal correction to Enthalpy= 0.625543
Thermal correction to Gibbs Free Energy= 0.502373
Sum of electronic and zero-point Energies= -2515.470702
Sum of electronic and thermal Energies= -2515.428723
Sum of electronic and thermal Enthalpies= -2515.427778
Sum of electronic and thermal Free Energies= -2515.550949

Solvent: -2516.46351409

Ru	1.057076	0.913437	-0.260394
Cl	1.603131	1.208044	-2.506861
Cl	1.576929	1.722430	1.843580
N	2.724823	-1.493348	-0.101852
N	0.714527	-2.134875	-0.694643
C	1.432684	-1.005228	-0.301347
C	2.797471	-2.860439	-0.370480
H	3.735385	-3.414685	-0.266288
C	1.546535	-3.260781	-0.736471
H	1.155200	-4.241501	-1.023732
C	3.859320	-0.746145	0.398107
C	4.815844	-0.233009	-0.514093
C	4.708814	-0.468504	-2.001527
H	5.569057	-0.015728	-2.531724
H	3.776049	-0.032245	-2.417941
H	4.694929	-1.553273	-2.242424
C	5.908608	0.479320	0.022225
H	6.654722	0.896390	-0.674570
C	6.082355	0.658883	1.407932
C	7.236920	1.470269	1.950337
H	7.616886	1.055074	2.906268
H	6.919854	2.516695	2.154688
H	8.080220	1.517089	1.232272
C	5.144608	0.067415	2.278842
H	5.282088	0.163170	3.368727
C	4.031559	-0.648329	1.802732
C	3.063977	-1.303116	2.759273
H	3.426535	-1.223314	3.802387
H	2.920046	-2.379424	2.525454
H	2.070526	-0.811060	2.706669
C	-0.708279	-2.301357	-0.901077
C	-1.470361	-2.836041	0.169993
C	-0.836746	-3.152051	1.507157
H	-1.609794	-3.380993	2.266236
H	-0.219278	-2.309906	1.884769
H	-0.158640	-4.030600	1.444320
C	-2.841082	-3.069875	-0.043159
H	-3.444905	-3.471106	0.787718
C	-3.460154	-2.795570	-1.279683
C	-4.945436	-3.008959	-1.462711
H	-5.225336	-3.053640	-2.534181
H	-5.520603	-2.176452	-1.001491
H	-5.286964	-3.945453	-0.975260
C	-2.658004	-2.306473	-2.327890
H	-3.115557	-2.115522	-3.312905
C	-1.276387	-2.065214	-2.175524
C	-0.444720	-1.609111	-3.347688
H	0.434235	-2.270525	-3.501794
H	-0.039333	-0.587035	-3.194714
H	-1.043671	-1.611440	-4.278942
H	-1.553168	-0.522939	1.223196
C	-1.772007	0.369010	0.629759
C	-2.987007	1.040231	0.628347
C	-0.804306	1.058683	-0.206215
C	-2.858849	2.217608	-0.268471
C	-1.520365	2.235990	-0.780487
C	-3.754753	3.238774	-0.609706

C	-1.109892	3.246315	-1.660395
C	-3.328632	4.262922	-1.489041
H	-4.774171	3.262733	-0.195080
C	-2.027522	4.262704	-2.014713
H	-0.090251	3.241316	-2.075272
H	-4.029941	5.067874	-1.761238
H	-1.712281	5.063070	-2.702512
C	-4.175058	0.660991	1.410028
C	-5.485585	0.786081	0.878072
C	-4.036206	0.130224	2.719475
C	-6.607201	0.391339	1.624691
H	-5.619112	1.165068	-0.146443
C	-5.158921	-0.256760	3.465212
H	-3.029832	0.054281	3.160326
C	-6.450830	-0.128785	2.921899
H	-7.613952	0.488240	1.187232
H	-5.026283	-0.652484	4.485005
H	-7.332838	-0.429495	3.509391

Catalyst 2, I-III TS

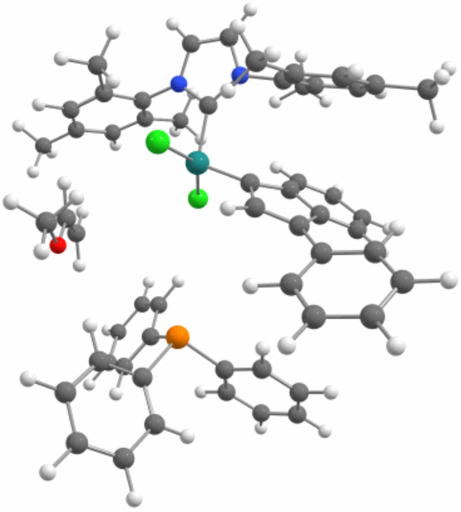

Zero-point correction= 0.931124 (Hartree/Particle)
Thermal correction to Energy= 0.997481
Thermal correction to Enthalpy= 0.998425
Thermal correction to Gibbs Free Energy= 0.812581
Sum of electronic and zero-point Energies= -3743.809010
Sum of electronic and thermal Energies= -3743.742653
Sum of electronic and thermal Enthalpies= -3743.741708
Sum of electronic and thermal Free Energies= -3743.927553

Solvent: -3745.46212023

Ru	-1,524151000	-0,683603000	0,210119000
Cl	-0,731250000	-1,362811000	-1,864176000
Cl	-1,440406000	-0,904481000	2,556459000
P	3,967387000	-0,130611000	-0,299263000
N	-4,082121000	-2,117077000	0,088492000
N	-4,538827000	0,024389000	0,185784000
C	-3,469576000	-0,863616000	0,100910000
C	-5,471218000	-2,000341000	0,162387000
H	-6,120100000	-2,881640000	0,160022000
C	-5,755508000	-0,668277000	0,222936000
H	-6,708147000	-0,134362000	0,289605000
C	-3,414194000	-3,396031000	-0,026744000
C	-3,163615000	-3,916073000	-1,322006000
C	-3,606583000	-3,182040000	-2,564446000
H	-3,422492000	-3,793754000	-3,468730000
H	-3,046833000	-2,230957000	-2,677954000
H	-4,689416000	-2,936561000	-2,529203000
C	-2,515860000	-5,163818000	-1,414420000
H	-2,299296000	-5,571034000	-2,415841000
C	-2,150568000	-5,904979000	-0,273818000
C	-1,415670000	-7,220821000	-0,392531000
H	-1,858337000	-7,996370000	0,266731000
H	-0,353284000	-7,104959000	-0,085317000
H	-1,423390000	-7,604705000	-1,431977000
C	-2,496349000	-5,389086000	0,991877000
H	-2,272527000	-5,980491000	1,895553000
C	-3,143670000	-4,147405000	1,147841000
C	-3,567138000	-3,672068000	2,516379000
H	-3,388209000	-4,458356000	3,275399000
H	-4,646259000	-3,410974000	2,543641000
H	-3,005650000	-2,761435000	2,817717000
C	-4,538387000	1,470253000	0,140952000
C	-4,310795000	2,213987000	1,324839000
C	-4,064555000	1,541471000	2,651452000
H	-3,968245000	2,291419000	3,460293000
H	-3,147088000	0,917228000	2,643726000
H	-4,902361000	0,860351000	2,915706000
C	-4,377989000	3,619573000	1,234583000
H	-4,192083000	4,211542000	2,146159000
C	-4,693382000	4,284007000	0,033845000
C	-4,728782000	5,793798000	-0,041546000
H	-5,625302000	6,154999000	-0,587385000
H	-3,841525000	6,182105000	-0,587544000
H	-4,729507000	6,255754000	0,965799000
C	-4,972649000	3,501730000	-1,104080000
H	-5,244271000	3,999297000	-2,049984000
C	-4,910307000	2,096198000	-1,075844000
C	-5,279142000	1,293990000	-2,304139000
H	-6,340947000	0,964466000	-2,265861000
H	-4,665942000	0,377432000	-2,407648000
H	-5,156111000	1,900902000	-3,222437000
H	-0,000831000	1,436705000	1,909946000
C	-0,318396000	1,853100000	0,945912000
C	-0,086796000	3,153068000	0,508304000
C	-1,192067000	1,149401000	0,012296000
C	-0,728591000	3,303114000	-0,825071000
C	-1,413105000	2,080275000	-1,122490000
C	-0,635912000	4,308735000	-1,795010000
C	-1,958647000	1,864843000	-2,390535000
C	-1,236382000	4,102638000	-3,061506000
H	-0,063911000	5,229973000	-1,605491000
C	-1,881131000	2,893269000	-3,359668000
H	-2,412038000	0,893855000	-2,633499000
H	-1,165037000	4,890181000	-3,828688000
H	-2,313569000	2,734331000	-4,360337000
C	0,657643000	4,179403000	1,248326000
C	0,335508000	5,558759000	1,143975000
C	1,717408000	3,806401000	2,119666000
C	1,042617000	6,521973000	1,879769000
H	-0,505245000	5,870355000	0,506575000
C	2,425058000	4,771667000	2,848487000
H	1,996370000	2,744312000	2,201431000
C	2,092517000	6,134881000	2,731657000
H	0,767543000	7,585275000	1,791553000
H	3,249475000	4,459010000	3,508923000

H	2,651343000	6,892684000	3,303438000
C	4,252021000	-1,608367000	-1,392971000
C	5,462209000	-2,338075000	-1,439046000
C	3,174107000	-2,019037000	-2,212165000
C	5,594697000	-3,446527000	-2,294213000
H	6,306061000	-2,037562000	-0,798031000
C	3,314145000	-3,120399000	-3,074280000
H	2,210814000	-1,484147000	-2,166018000
C	4,523053000	-3,837602000	-3,116913000
H	6,543233000	-4,007514000	-2,317845000
H	2,464219000	-3,427016000	-3,704863000
H	4,628297000	-4,707548000	-3,785228000
C	5,304640000	-0,342140000	0,979733000
C	6,475545000	0,445343000	1,042104000
C	5,085283000	-1,319986000	1,982084000
C	7,411069000	0,256168000	2,076316000
H	6,660501000	1,212217000	0,273829000
C	6,030100000	-1,515663000	3,002938000
H	4,169346000	-1,936009000	1,965353000
C	7,194500000	-0,726608000	3,056780000
H	8,319526000	0,879662000	2,109489000
H	5,850689000	-2,288537000	3,767996000
H	7,929823000	-0,877158000	3,863628000
C	4,631867000	1,261514000	-1,343233000
C	5,427822000	1,081923000	-2,496719000
C	4,267710000	2,576993000	-0,968799000
C	5,860289000	2,190354000	-3,246568000
H	5,709411000	0,065159000	-2,812772000
C	4,711458000	3,684425000	-1,710935000
H	3,621687000	2,735075000	-0,088822000
C	5,508107000	3,493683000	-2,854379000
H	6,478322000	2,032263000	-4,145430000
H	4,420867000	4,700998000	-1,400566000
H	5,847749000	4,360417000	-3,444087000
C	1,194729000	-3,869339000	1,755733000
C	1,500384000	-4,247812000	0,499117000
H	0,709272000	-4,668621000	-0,136469000
H	2,504474000	-4,100023000	0,073661000
H	0,174267000	-3,991805000	2,167946000
O	2,103359000	-3,304350000	2,603175000
C	1,604699000	-2,945487000	3,888994000
H	2,432067000	-2,443343000	4,426236000
H	0,743597000	-2,245251000	3,809294000
H	1,295459000	-3,844616000	4,471242000

Catalyst 2, II-III TS

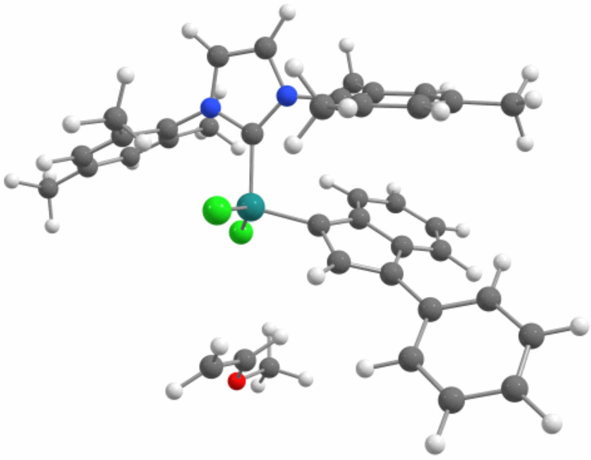

Zero-point correction= 0.664869 (Hartree/Particle)

Thermal correction to Energy= 0.713396

Ru	-0.947761000	0.541912000	-0.249519000
Cl	-1.727145000	1.334066000	1.793946000
Cl	-0.874535000	0.873043000	-2.569193000
N	-2.892070000	-1.639675000	-0.498875000
N	-0.893342000	-2.536490000	-0.575932000
C	-1.541040000	-1.318595000	-0.379337000
C	-3.066424000	-2.998620000	-0.764459000
H	-4.063030000	-3.433529000	-0.888528000
C	-1.823205000	-3.557280000	-0.809494000
H	-1.498833000	-4.587714000	-0.983055000
C	-4.004813000	-0.731419000	-0.317220000
C	-4.530025000	-0.563931000	0.990057000
C	-3.964919000	-1.322873000	2.166637000
H	-4.568581000	-1.143413000	3.077248000
H	-2.925829000	-0.997444000	2.379248000
H	-3.943975000	-2.417180000	1.976803000
C	-5.614661000	0.316542000	1.153601000
H	-6.021774000	0.468100000	2.167102000
C	-6.193864000	1.000408000	0.065360000
C	-7.320346000	1.985494000	0.279790000
H	-7.954389000	2.087928000	-0.623861000
H	-6.918124000	2.995607000	0.514778000
H	-7.969606000	1.689318000	1.128871000
C	-5.689721000	0.745556000	-1.224404000
H	-6.160286000	1.232871000	-2.094554000
C	-4.606285000	-0.128350000	-1.451500000
C	-4.149147000	-0.433257000	-2.857553000
H	-4.807328000	0.061756000	-3.597657000
H	-4.164172000	-1.524714000	-3.063910000
H	-3.108375000	-0.084865000	-3.032337000
C	0.510788000	-2.862459000	-0.448139000
C	1.376868000	-2.706758000	-1.558520000
C	0.881956000	-2.205876000	-2.891610000
H	1.691614000	-2.234661000	-3.646346000
H	0.493735000	-1.167454000	-2.834290000
H	0.042675000	-2.830316000	-3.267070000
C	2.722530000	-3.095575000	-1.395502000
H	3.409218000	-2.972006000	-2.249401000
C	3.207003000	-3.652487000	-0.196349000
C	4.662417000	-4.031472000	-0.038984000
H	5.196725000	-3.291566000	0.596354000
H	5.184765000	-4.070974000	-1.015575000
H	4.776336000	-5.019031000	0.454670000
C	2.295559000	-3.849484000	0.859580000
H	2.645100000	-4.310178000	1.798527000

Thermal correction to Enthalpy= 0.714340
Thermal correction to Gibbs Free Energy= 0.575487
Sum of electronic and zero-point Energies= -2708.353090
Sum of electronic and thermal Energies= -2708.304563
Sum of electronic and thermal Enthalpies= -2708.303619
Sum of electronic and thermal Free Energies= -2708.442472

Solvent: -2709.51740772

C	0.943077000	-3.474466000	0.756086000
C	-0.016658000	-3.783607000	1.883251000
H	-0.531569000	-4.754971000	1.712041000
H	-0.809006000	-3.017261000	1.987888000
H	0.520568000	-3.861848000	2.848559000
H	1.709726000	1.583429000	-1.570593000
C	1.896085000	1.114170000	-0.596303000
C	3.144621000	0.958423000	-0.000958000
C	0.874414000	0.401693000	0.165907000
C	2.950260000	0.209535000	1.267919000
C	1.563860000	-0.139042000	1.364677000
C	3.815477000	-0.046459000	2.339718000
C	1.054806000	-0.695771000	2.541571000
C	3.301519000	-0.659779000	3.508077000
H	4.871868000	0.261730000	2.302794000
C	1.937150000	-0.971938000	3.612355000
H	-0.023585000	-0.886430000	2.634581000
H	3.977034000	-0.863627000	4.354300000
H	1.546276000	-1.418302000	4.540647000
C	4.417473000	1.447331000	-0.546088000
C	5.632351000	0.736283000	-0.357652000
C	4.457238000	2.647043000	-1.306789000
C	6.834012000	1.204444000	-0.910507000
H	5.621107000	-0.213907000	0.196867000
C	5.660262000	3.114840000	-1.851901000
H	3.531458000	3.227744000	-1.442431000
C	6.855149000	2.396495000	-1.656561000
H	7.762636000	0.630215000	-0.762370000
H	5.668267000	4.053041000	-2.429245000
H	7.800559000	2.766060000	-2.084477000
C	0.168291000	4.410211000	0.151981000
C	-0.212782000	4.650967000	-1.120326000
H	0.088066000	3.959501000	-1.918876000
H	-0.847265000	5.514825000	-1.371820000
H	0.796403000	3.535208000	0.413229000
O	-0.179831000	5.214528000	1.191329000
C	0.219286000	4.759937000	2.481322000
H	-0.140285000	5.510449000	3.211398000
H	-0.231468000	3.771366000	2.720794000
H	1.328545000	4.684937000	2.564344000

Catalyst 2, III intermediate

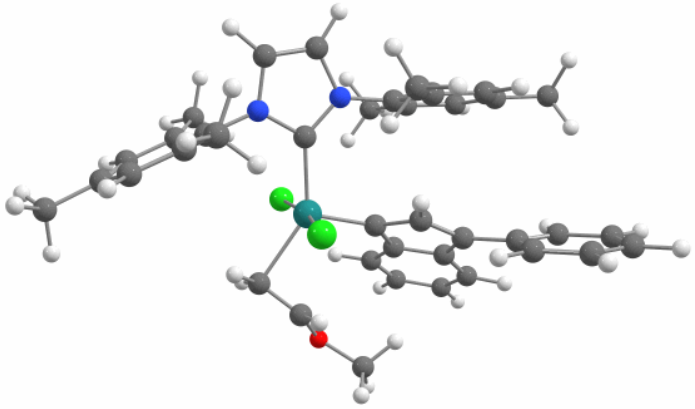

Zero-point correction= 0.667600 (Hartree/Particle)
Thermal correction to Energy= 0.714968
Thermal correction to Enthalpy= 0.715912
Thermal correction to Gibbs Free Energy= 0.583998
Sum of electronic and zero-point Energies= -2708.371476

Ru	1.079388000	0.698948000	0.100521000
Cl	1.714511000	1.231526000	-2.155156000
Cl	0.858176000	0.237787000	2.489223000
N	2.712811000	-1.787673000	-0.260734000
N	0.648680000	-2.331348000	-0.701662000
C	1.426967000	-1.263326000	-0.300211000
C	2.720627000	-3.134017000	-0.629135000
H	3.647728000	-3.714781000	-0.656361000
C	1.428998000	-3.476396000	-0.903724000
H	0.980110000	-4.421037000	-1.225240000
C	3.927489000	-1.089157000	0.114860000
C	4.764667000	-0.564445000	-0.906353000
C	4.463911000	-0.771748000	-2.369563000
H	5.251092000	-0.313868000	-2.999455000
H	3.488374000	-0.316638000	-2.643015000
H	4.412036000	-1.852865000	-2.621630000
C	5.935389000	0.111654000	-0.508007000
H	6.584267000	0.538856000	-1.290693000
C	6.310584000	0.234056000	0.844382000
C	7.546788000	1.009083000	1.241615000
H	8.004094000	0.606018000	2.168055000
H	7.298981000	2.075703000	1.438079000
H	8.314036000	0.996094000	0.441092000
C	5.509130000	-0.400229000	1.813836000
H	5.821956000	-0.381704000	2.871257000
C	4.327196000	-1.092021000	1.478342000
C	3.562781000	-1.851815000	2.532991000
H	4.142991000	-1.900699000	3.475082000
H	3.345218000	-2.890808000	2.206848000
H	2.587200000	-1.361520000	2.746944000
C	-0.784635000	-2.403161000	-0.870117000
C	-1.550736000	-2.963645000	0.183790000
C	-0.910167000	-3.345160000	1.498978000
H	-1.676123000	-3.666092000	2.231480000
H	-0.342086000	-2.495636000	1.937305000
H	-0.186980000	-4.180965000	1.380703000
C	-2.928432000	-3.145186000	-0.032627000
H	-3.539162000	-3.576134000	0.778110000
C	-3.545880000	-2.786645000	-1.249374000
C	-5.028223000	-3.006737000	-1.448918000
H	-5.350960000	-2.725165000	-2.470970000
H	-5.619735000	-2.406093000	-0.725229000
H	-5.303844000	-4.070513000	-1.285292000
C	-2.745539000	-2.230148000	-2.265095000
H	-3.209924000	-1.950353000	-3.225405000
C	-1.356248000	-2.039653000	-2.111393000
C	-0.515496000	-1.495807000	-3.239837000
H	0.279881000	-2.214685000	-3.533244000
H	0.004695000	-0.555415000	-2.954871000
H	-1.137844000	-1.294959000	-4.133343000
H	-1.537739000	-0.513154000	1.522242000

Sum of electronic and thermal Energies= -2708.324108
Sum of electronic and thermal Enthalpies= -2708.323164
Sum of electronic and thermal Free Energies= -2708.455078

Solvent: -2709.54119510

C	-1.791028000	0.217769000	0.749349000
C	-3.064420000	0.677561000	0.467347000
C	-0.787915000	0.861392000	-0.103128000
C	-2.950431000	1.682802000	-0.623532000
C	-1.564847000	1.795717000	-0.982467000
C	-3.906034000	2.514775000	-1.219021000
C	-1.169948000	2.718351000	-1.959222000
C	-3.496069000	3.438053000	-2.212608000
H	-4.962874000	2.474494000	-0.914786000
C	-2.148263000	3.530233000	-2.583775000
H	-0.108784000	2.801066000	-2.233354000
H	-4.246205000	4.089045000	-2.689981000
H	-1.838978000	4.249520000	-3.358778000
C	-4.291948000	0.279460000	1.175598000
C	-5.550887000	0.208104000	0.523063000
C	-4.239985000	-0.060448000	2.554155000
C	-6.705575000	-0.183987000	1.219911000
H	-5.615626000	0.428459000	-0.552639000
C	-5.394139000	-0.450384000	3.247653000
H	-3.278554000	0.012075000	3.086216000
C	-6.634718000	-0.512313000	2.585320000
H	-7.669912000	-0.237365000	0.689256000
H	-5.327850000	-0.699559000	4.318936000
H	-7.542450000	-0.813000000	3.132283000
C	2.104143000	2.640282000	0.596577000
C	0.923002000	3.156338000	1.114782000
H	2.773992000	2.122805000	1.307366000
H	2.552601000	3.100299000	-0.298342000
H	0.520154000	2.802176000	2.083474000
O	0.276307000	4.158961000	0.504185000
C	-0.984012000	4.543667000	1.072504000
H	-0.878847000	4.747983000	2.160149000
H	-1.295679000	5.466692000	0.550750000
H	-1.745482000	3.753920000	0.906542000

Catalyst 2, III-IV TS

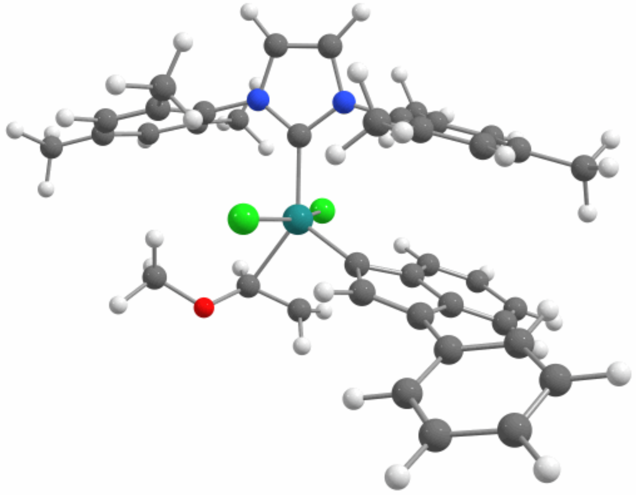

Zero-point correction= 0.667771 (Hartree/Particle)
Thermal correction to Energy= 0.713943
Thermal correction to Enthalpy= 0.714887
Thermal correction to Gibbs Free Energy= 0.587442
Sum of electronic and zero-point Energies= -2708.360995
Sum of electronic and thermal Energies= -2708.314823
Sum of electronic and thermal Enthalpies= -2708.313879
Sum of electronic and thermal Free Energies= -2708.441324

Ru	-0.784254000	0.268050000	0.707948000
Cl	-1.274082000	-1.418297000	2.396774000
Cl	-0.920068000	1.990663000	-1.006618000
N	-2.779309000	-1.131072000	-1.238819000
N	-0.770143000	-1.947234000	-1.442533000
C	-1.495135000	-1.006592000	-0.747771000
C	-2.842999000	-2.131424000	-2.214494000
H	-3.782719000	-2.381381000	-2.716475000
C	-1.582175000	-2.639454000	-2.345437000
H	-1.177423000	-3.423496000	-2.992625000
C	-3.945559000	-0.351105000	-0.871868000
C	-4.739215000	-0.771647000	0.227299000
C	-4.428798000	-2.030384000	0.998275000
H	-5.234406000	-2.252828000	1.724827000
H	-3.468680000	-1.949030000	1.554115000
H	-4.329967000	-2.903777000	0.318704000
C	-5.875669000	0.000104000	0.546336000
H	-6.496192000	-0.313208000	1.402747000
C	-6.256472000	1.126532000	-0.209153000
C	-7.461786000	1.952574000	0.181708000
H	-8.259981000	1.325968000	0.629536000
H	-7.890284000	2.490465000	-0.687825000
H	-7.190136000	2.720067000	0.939847000
C	-5.487107000	1.454082000	-1.343173000
H	-5.795560000	2.299438000	-1.980829000
C	-4.337929000	0.725001000	-1.710806000
C	-3.585114000	1.068797000	-2.972261000
H	-4.064856000	1.918321000	-3.496144000
H	-3.551166000	0.209809000	-3.676030000
H	-2.536360000	1.347933000	-2.732469000
C	0.643542000	-2.232160000	-1.313518000
C	1.562993000	-1.481491000	-2.085149000
C	1.106248000	-0.378512000	-3.008880000
H	1.968931000	0.076148000	-3.533163000
H	0.567705000	0.426073000	-2.460852000
H	0.403869000	-0.762223000	-3.780559000
C	2.927714000	-1.820443000	-1.979111000
H	3.658483000	-1.243009000	-2.569399000
C	3.377590000	-2.875482000	-1.162455000
C	4.848382000	-3.207150000	-1.049251000
H	5.025844000	-4.301451000	-1.105841000
H	5.252060000	-2.863481000	-0.071822000
H	5.442126000	-2.720471000	-1.848814000
C	2.419958000	-3.614947000	-0.439059000
H	2.750055000	-4.453608000	0.196328000
C	1.045377000	-3.317511000	-0.495502000
C	0.042578000	-4.125310000	0.292420000
H	-0.737307000	-4.570921000	-0.361560000
H	-0.483540000	-3.485837000	1.035441000
H	0.542296000	-4.951402000	0.834841000
H	1.428594000	2.359504000	-0.409951000
C	1.896322000	1.582221000	0.204343000
C	3.248635000	1.359163000	0.343176000
C	1.106995000	0.553126000	0.916548000
C	3.432920000	0.190484000	1.245128000
C	2.140730000	-0.304922000	1.606492000
C	4.590652000	-0.369714000	1.799591000

Solvent: -2709.53049907

C	2.029307000	-1.378643000	2.496435000
C	4.467090000	-1.451604000	2.702943000
H	5.585379000	0.037019000	1.562136000
C	3.201974000	-1.950865000	3.043482000
H	1.033487000	-1.760856000	2.766755000
H	5.373085000	-1.895228000	3.146614000
H	3.114307000	-2.791948000	3.749514000
C	4.314336000	2.147592000	-0.300808000
C	5.515689000	1.545483000	-0.756429000
C	4.144257000	3.540509000	-0.515025000
C	6.504397000	2.305974000	-1.400936000
H	5.656368000	0.461155000	-0.635554000
C	5.135428000	4.298741000	-1.153388000
H	3.225372000	4.029160000	-0.155405000
C	6.321712000	3.685790000	-1.598562000
H	7.424417000	1.814707000	-1.756392000
H	4.984197000	5.380050000	-1.300773000
H	7.101019000	4.282782000	-2.098390000
C	-0.024247000	1.523513000	2.501387000
C	-1.395848000	1.771458000	2.174283000
H	0.191906000	0.800266000	3.301674000
H	0.654359000	2.386180000	2.409244000
H	-2.180837000	1.165450000	2.678466000
O	-1.758289000	3.033812000	1.830404000
C	-3.095731000	3.191953000	1.354264000
H	-3.821389000	2.674398000	2.022609000
H	-3.307121000	4.277871000	1.353102000
H	-3.182007000	2.792209000	0.321814000

Catalyst 2, IV intermediate

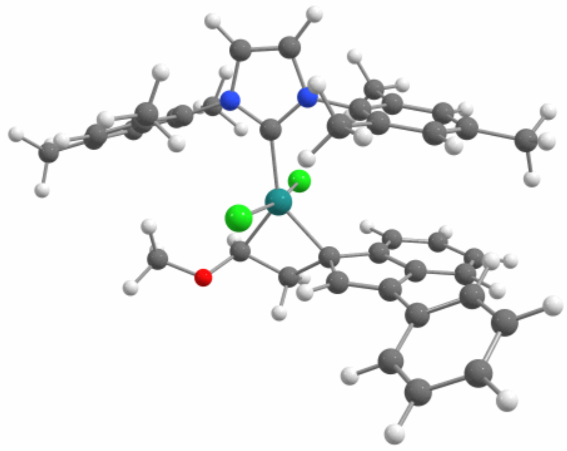

Zero-point correction= 0.669316 (Hartree/Particle)
Thermal correction to Energy= 0.715640
Thermal correction to Enthalpy= 0.716584
Thermal correction to Gibbs Free Energy= 0.588346
Sum of electronic and zero-point Energies= -2708.370150
Sum of electronic and thermal Energies= -2708.323825
Sum of electronic and thermal Enthalpies= -2708.322881
Sum of electronic and thermal Free Energies= -2708.451120

Solvent: -2709.53960006

Ru	0.754919	-0.258983	0.670456
Cl	1.210091	1.455576	2.329954
Cl	0.727686	-1.946052	-1.085172
N	2.749546	1.122081	-1.254410
N	0.725397	1.906050	-1.477354
C	1.464603	0.982023	-0.779669
C	2.802786	2.124463	-2.229471
H	3.743285	2.388929	-2.722276
C	1.534914	2.611093	-2.372171
H	1.121962	3.388771	-3.021785
C	3.930114	0.364210	-0.884478
C	4.727630	0.815619	0.197546
C	4.391388	2.067381	0.968443
H	5.188350	2.302640	1.700361
H	3.428194	1.968758	1.516866
H	4.282523	2.940641	0.289971
C	5.891272	0.077548	0.502913
H	6.516937	0.413825	1.346553
C	6.290881	-1.042714	-0.249882
C	7.534233	-1.825646	0.109621
H	8.135028	-2.071562	-0.790509
H	7.273449	-2.792038	0.594274
H	8.181705	-1.266491	0.814165
C	5.505147	-1.409418	-1.362097
H	5.821770	-2.256104	-1.994008
C	4.329477	-0.717481	-1.714090
C	3.548068	-1.107522	-2.943629
H	4.042580	-1.943654	-3.475080
H	3.452749	-0.259467	-3.654938
H	2.518870	-1.422989	-2.663527
C	-0.695930	2.169526	-1.352664
C	-1.599066	1.430414	-2.156260
C	-1.125257	0.360863	-3.108704
H	-1.975317	-0.056732	-3.681920
H	-0.621901	-0.473961	-2.570942
H	-0.388103	0.763005	-3.837096
C	-2.968311	1.753451	-2.057852
H	-3.684547	1.184215	-2.673237
C	-3.439110	2.782364	-1.220250
C	-4.915295	3.089068	-1.111427
H	-5.106171	4.182118	-1.090464
H	-5.330071	2.670110	-0.168419
H	-5.489059	2.654114	-1.954289
C	-2.497330	3.517859	-0.472907
H	-2.842549	4.342884	0.171983
C	-1.118243	3.240388	-0.523214
C	-0.136900	4.065859	0.272076
H	0.661136	4.494945	-0.370806
H	0.366169	3.444488	1.045640
H	-0.652099	4.904445	0.779485
H	-1.403535	-2.644824	0.025326
C	-1.868356	-1.818859	0.575593
C	-3.192794	-1.454827	0.524659
C	-1.079237	-0.884521	1.422553
C	-3.379784	-0.285722	1.419727
C	-2.112820	0.062950	1.981714
C	-4.545241	0.379854	1.832839
C	-2.026627	1.080930	2.940609
C	-4.447324	1.407771	2.795113
H	-5.530670	0.082931	1.442865
C	-3.200892	1.755027	3.341545

H	-1.048788	1.368119	3.354485
H	-5.358664	1.931077	3.127123
H	-3.134383	2.556352	4.094618
C	-4.237062	-2.130779	-0.270599
C	-5.293877	-1.406594	-0.879169
C	-4.190673	-3.533858	-0.476532
C	-6.260535	-2.058280	-1.662461
H	-5.334853	-0.313266	-0.764607
C	-5.157500	-4.183465	-1.257220
H	-3.389170	-4.118652	0.001250
C	-6.199704	-3.449516	-1.853479
H	-7.066481	-1.471740	-2.132450
H	-5.101485	-5.275044	-1.396775
H	-6.961059	-3.960868	-2.463789
C	-0.005554	-1.509320	2.410866
C	1.521005	-1.540114	1.993052
H	-0.077682	-0.978741	3.377858
H	-0.262318	-2.583181	2.493016
H	2.184661	-1.007377	2.714170
O	1.942205	-2.813122	1.718457
C	3.340043	-2.952470	1.481349
H	3.936072	-2.442095	2.273072
H	3.561720	-4.036432	1.494873
H	3.612033	-2.531077	0.490304

Catalyst 2, IV-V TS

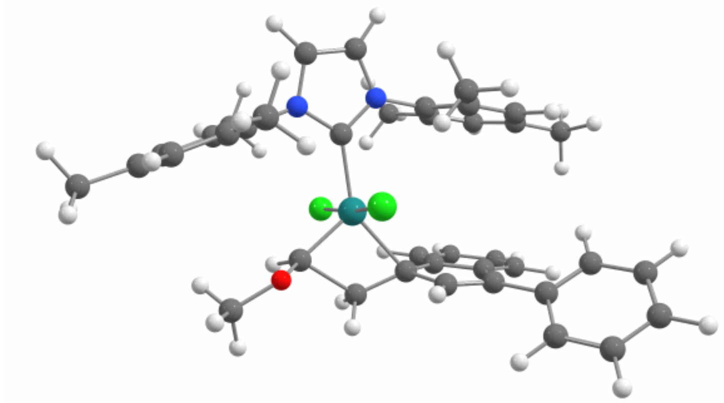

Zero-point correction= 0.667969 (Hartree/Particle)
Thermal correction to Energy= 0.714098
Thermal correction to Enthalpy= 0.715042
Thermal correction to Gibbs Free Energy= 0.586846
Sum of electronic and zero-point Energies= -2708.367129
Sum of electronic and thermal Energies= -2708.321001
Sum of electronic and thermal Enthalpies= -2708.320056
Sum of electronic and thermal Free Energies= -2708.448252

Solvent: -2709.53545127

Ru	0.708346000	-0.348227000	0.517352000
Cl	1.348988000	0.982807000	2.454036000
Cl	0.319371000	-1.595138000	-1.528053000
N	2.796956000	1.246484000	-1.157400000
N	0.811883000	2.131360000	-1.315073000
C	1.497810000	1.093920000	-0.729740000
C	2.911475000	2.360676000	-1.995886000
H	3.871316000	2.646064000	-2.437145000
C	1.666274000	2.914497000	-2.096012000
H	1.301194000	3.789807000	-2.641986000
C	3.943308000	0.428406000	-0.811764000
C	4.745729000	0.813156000	0.292510000
C	4.431378000	2.037571000	1.115129000
H	5.242100000	2.242700000	1.841137000
H	3.480613000	1.902440000	1.677802000
H	4.305978000	2.938306000	0.477531000
C	5.870077000	0.016223000	0.591546000
H	6.498266000	0.299153000	1.452919000
C	6.223130000	-1.105931000	-0.184156000
C	7.461876000	-1.914182000	0.135990000
H	7.703625000	-1.884851000	1.217783000
H	8.348050000	-1.515958000	-0.406195000
H	7.349249000	-2.975430000	-0.165724000
C	5.425527000	-1.417659000	-1.302974000
H	5.700237000	-2.272190000	-1.944030000
C	4.288899000	-0.660058000	-1.652585000
C	3.495128000	-0.991034000	-2.890640000
H	3.970543000	-1.818596000	-3.452233000
H	3.419645000	-0.115378000	-3.570491000
H	2.453970000	-1.288995000	-2.630743000
C	-0.597960000	2.430374000	-1.151085000
C	-1.516071000	1.951085000	-2.118835000
C	-1.066228000	1.135841000	-3.305484000
H	-1.922724000	0.897937000	-3.965554000
H	-0.599990000	0.180422000	-2.975701000
H	-0.308536000	1.678127000	-3.911074000
C	-2.875898000	2.280737000	-1.942633000
H	-3.605223000	1.906951000	-2.680286000
C	-3.322520000	3.075353000	-0.869796000
C	-4.789344000	3.390292000	-0.682629000
H	-4.952537000	4.470215000	-0.483175000
H	-5.198778000	2.836507000	0.190212000
H	-5.387860000	3.110225000	-1.572314000
C	-2.364739000	3.574787000	0.035496000
H	-2.690484000	4.224230000	0.864767000
C	-0.993334000	3.281601000	-0.086178000
C	0.008952000	3.876693000	0.871926000
H	0.847081000	4.369082000	0.334485000
H	0.458962000	3.094955000	1.523987000
H	-0.473515000	4.633609000	1.520290000
H	-1.565878000	-2.799423000	-0.218596000
C	-1.958175000	-2.005447000	0.426318000
C	-3.256301000	-1.555631000	0.456782000
C	-1.100761000	-1.232995000	1.358068000
C	-3.342177000	-0.473132000	1.467459000
C	-2.044087000	-0.273283000	2.033399000
C	-4.449695000	0.226679000	1.972802000
C	-1.869868000	0.626443000	3.093485000
C	-4.262588000	1.137930000	3.034183000
H	-5.459142000	0.042759000	1.574209000
C	-2.986501000	1.334595000	3.588222000
H	-0.867642000	0.796362000	3.513373000
H	-5.128263000	1.686913000	3.439009000
H	-2.851423000	2.043009000	4.421029000
C	-4.369638000	-2.084651000	-0.355527000
C	-5.370181000	-1.231541000	-0.886912000
C	-4.451147000	-3.469261000	-0.651685000

C	-6.407002000	-1.743850000	-1.683592000
H	-5.309056000	-0.148618000	-0.701625000
C	-5.488106000	-3.979688000	-1.445930000
H	-3.694094000	-4.150534000	-0.232488000
C	-6.473699000	-3.119704000	-1.964511000
H	-7.167615000	-1.060063000	-2.093970000
H	-5.532379000	-5.060321000	-1.656812000
H	-7.290798000	-3.521140000	-2.584881000
C	0.054368000	-1.886104000	2.031768000
C	1.764482000	-1.722721000	1.290426000
H	0.221442000	-1.554905000	3.072076000
H	0.047201000	-2.981859000	1.894920000
H	2.378673000	-1.453929000	2.184207000
O	2.181213000	-2.846610000	0.674201000
C	3.197949000	-3.602641000	1.348475000
H	2.806142000	-4.050765000	2.288553000
H	3.496746000	-4.412242000	0.657002000
H	4.081952000	-2.969087000	1.575660000

Catalyst 2, V intermediate

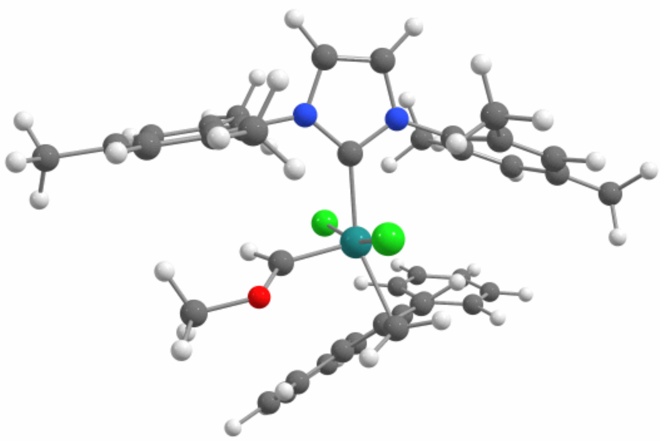

Zero-point correction= 0.667367 (Hartree/Particle)
Thermal correction to Energy= 0.714714
Thermal correction to Enthalpy= 0.715658
Thermal correction to Gibbs Free Energy= 0.582623
Sum of electronic and zero-point Energies= -2708.385755
Sum of electronic and thermal Energies= -2708.338409
Sum of electronic and thermal Enthalpies= -2708.337465
Sum of electronic and thermal Free Energies= -2708.470500

Solvent: -2709.55333611

Ru	0.406711000	0.055374000	-0.799877000
Cl	-0.288981000	-1.021562000	1.269443000
Cl	1.466427000	1.446403000	-2.515476000
N	2.565695000	1.402236000	1.140835000
N	0.791384000	2.618405000	0.812210000
C	1.361856000	1.408859000	0.472221000
C	2.734906000	2.578921000	1.876719000
H	3.633538000	2.755480000	2.475707000
C	1.619763000	3.341470000	1.669763000
H	1.332817000	4.325576000	2.053027000
C	3.568820000	0.358042000	1.174125000
C	3.561186000	-0.555371000	2.261236000
C	2.515089000	-0.483483000	3.345010000
H	2.680559000	-1.269747000	4.106839000
H	1.497139000	-0.613713000	2.914728000
H	2.530635000	0.500280000	3.861367000
C	4.579330000	-1.527847000	2.303588000
H	4.579246000	-2.250464000	3.136716000
C	5.601000000	-1.590168000	1.333302000
C	6.679127000	-2.647997000	1.419231000
H	7.456259000	-2.508278000	0.641687000
H	6.254712000	-3.667714000	1.294359000
H	7.181503000	-2.632546000	2.409585000
C	5.593993000	-0.633577000	0.299496000
H	6.403245000	-0.640272000	-0.449986000
C	4.595606000	0.360710000	0.198561000
C	4.653355000	1.404450000	-0.888606000
H	5.537328000	1.248416000	-1.536878000
H	4.724512000	2.426983000	-0.458485000
H	3.738848000	1.392523000	-1.523199000
C	-0.494404000	3.120030000	0.359992000
C	-0.528289000	4.049902000	-0.714671000
C	0.732247000	4.579307000	-1.352558000
H	0.489386000	5.287624000	-2.168218000
H	1.336950000	3.748461000	-1.775311000
H	1.365357000	5.117188000	-0.614216000
C	-1.790100000	4.506083000	-1.141397000
H	-1.831411000	5.214698000	-1.985452000
C	-2.988090000	4.101630000	-0.517099000
C	-4.326397000	4.581155000	-1.032727000
H	-5.133503000	4.422830000	-0.290062000
H	-4.614108000	4.035364000	-1.957988000
H	-4.300809000	5.659942000	-1.292131000
C	-2.897931000	3.258006000	0.605796000
H	-3.815518000	2.980313000	1.150314000
C	-1.663034000	2.770975000	1.086346000
C	-1.605649000	1.974199000	2.365122000
H	-0.888972000	2.424439000	3.084998000
H	-1.269590000	0.928504000	2.186851000
H	-2.601485000	1.941340000	2.847402000
H	-2.904806000	0.397982000	-1.025499000
C	-2.607270000	-0.660832000	-1.021361000
C	-3.238683000	-1.671504000	-0.322461000
C	-1.416283000	-1.178698000	-1.707580000
C	-2.502293000	-2.931104000	-0.574410000
C	-1.394329000	-2.636972000	-1.424997000
C	-2.769776000	-4.262639000	-0.205591000
C	-0.566616000	-3.661581000	-1.902253000
C	-1.920936000	-5.284943000	-0.671019000
H	-3.639649000	-4.510262000	0.422116000
C	-0.829295000	-4.990286000	-1.512791000
H	0.269447000	-3.435258000	-2.583681000
H	-2.122011000	-6.330137000	-0.385761000
H	-0.187915000	-5.806617000	-1.882299000
C	-4.445427000	-1.522073000	0.512786000
C	-4.568470000	-2.196732000	1.754151000
C	-5.507896000	-0.675147000	0.106769000
C	-5.707953000	-2.026972000	2.556710000
H	-3.739032000	-2.829229000	2.105296000
C	-6.646570000	-0.507972000	0.909788000
H	-5.439923000	-0.162940000	-0.866164000
C	-6.753730000	-1.184745000	2.138567000
H	-5.776616000	-2.553492000	3.522418000
H	-7.463712000	0.148184000	0.568456000
H	-7.649988000	-1.057576000	2.766563000

C	-0.655056000	-0.477681000	-2.667800000
C	1.706910000	-1.237228000	-0.898101000
H	0.029491000	-1.016767000	-3.341344000
H	-1.037059000	0.475699000	-3.066986000
H	1.913242000	-1.893162000	-0.015147000
O	2.408389000	-1.551265000	-1.982876000
C	3.334200000	-2.657574000	-1.883103000
H	2.816393000	-3.567378000	-1.514784000
H	3.718362000	-2.831534000	-2.904932000
H	4.171142000	-2.391546000	-1.205599000

Catalyst 2, V-VI TS

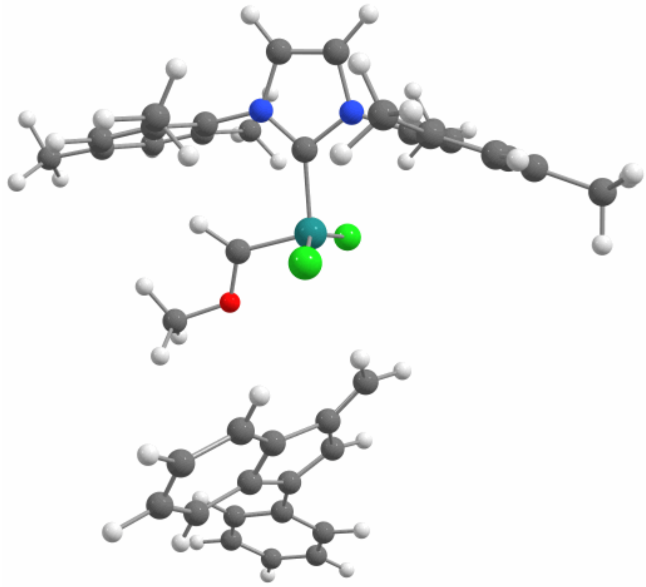

Zero-point correction= 0.665510 (Hartree/Particle)
Thermal correction to Energy= 0.713574
Thermal correction to Enthalpy= 0.714519
Thermal correction to Gibbs Free Energy= 0.575410
Sum of electronic and zero-point Energies= -2708.379623
Sum of electronic and thermal Energies= -2708.331559
Sum of electronic and thermal Enthalpies= -2708.330614
Sum of electronic and thermal Free Energies= -2708.469723

Solvent: -2709.53586546

Ru	1.047751000	-0.269813000	-0.062668000
Cl	0.602685000	-0.680866000	-2.330051000
Cl	1.012483000	-0.853825000	2.227195000
N	3.512033000	1.534915000	-0.356752000
N	3.963068000	-0.604196000	-0.273373000
C	2.894570000	0.295064000	-0.220426000
C	4.900188000	1.398406000	-0.488690000
H	5.548962000	2.271770000	-0.606938000
C	5.178424000	0.063850000	-0.437532000
H	6.125334000	-0.481142000	-0.502996000
C	2.885491000	2.831283000	-0.365526000
C	2.450308000	3.375692000	-1.598382000
C	2.589285000	2.602703000	-2.887509000
H	2.268177000	3.214365000	-3.752815000
H	1.974557000	1.675381000	-2.868610000
H	3.638958000	2.284615000	-3.061816000
C	1.858361000	4.655230000	-1.573410000
H	1.508484000	5.091734000	-2.523852000
C	1.703904000	5.387754000	-0.378520000
C	1.105734000	6.777234000	-0.390077000
H	0.550200000	6.992791000	0.545250000
H	0.414724000	6.920235000	-1.245084000
H	1.901041000	7.549925000	-0.479954000
C	2.153675000	4.805588000	0.825682000
H	2.036338000	5.359957000	1.772027000
C	2.750662000	3.529432000	0.860253000
C	3.189375000	2.903778000	2.163695000
H	3.037417000	3.601029000	3.010249000
H	4.262273000	2.617847000	2.142450000
H	2.618396000	1.972980000	2.373483000
C	3.876586000	-2.045027000	-0.166222000
C	4.050684000	-2.648093000	1.107690000
C	4.344273000	-1.828390000	2.340475000
H	4.611787000	-2.484321000	3.191697000
H	3.453721000	-1.231384000	2.631977000
H	5.183098000	-1.120089000	2.174191000
C	3.969134000	-4.051761000	1.183732000
H	4.091027000	-4.532119000	2.168927000
C	3.752776000	-4.854110000	0.045392000
C	3.614723000	-6.354692000	0.169106000
H	3.924496000	-6.874059000	-0.760271000
H	2.556822000	-6.637491000	0.365120000
H	4.218175000	-6.754783000	1.009225000
C	3.670929000	-4.219235000	-1.209076000
H	3.560075000	-4.832102000	-2.119210000
C	3.748655000	-2.818604000	-1.349888000
C	3.736082000	-2.185831000	-2.719784000
H	4.552307000	-1.442343000	-2.835928000
H	2.778413000	-1.650733000	-2.902658000
H	3.857043000	-2.956080000	-3.506296000
H	-4.311546000	-2.421998000	-1.359708000
C	-4.310332000	-1.812205000	-0.445336000
C	-5.340486000	-0.996586000	-0.020137000
C	-3.164793000	-1.713186000	0.464278000
C	-4.902658000	-0.323220000	1.231463000
C	-3.572769000	-0.756087000	1.523941000
C	-5.561757000	0.557032000	2.107594000
C	-2.894733000	-0.288696000	2.656803000
C	-4.876792000	1.022783000	3.249832000
H	-6.600658000	0.870373000	1.922467000
C	-3.556067000	0.613300000	3.518315000
H	-1.861207000	-0.608661000	2.867365000
H	-5.386330000	1.711386000	3.943258000
H	-3.037761000	0.988965000	4.415106000
C	-6.638714000	-0.822801000	-0.699956000
C	-7.275876000	0.442397000	-0.772460000
C	-7.272213000	-1.922447000	-1.333829000
C	-8.492711000	0.600455000	-1.454972000
C	-8.488668000	-1.763331000	-2.013719000
C	-9.106562000	-0.500868000	-2.077001000
H	-8.962937000	1.595747000	-1.506554000
H	-8.963998000	-2.634747000	-2.492224000
H	-10.063705000	-0.376868000	-2.608137000
C	-1.977564000	-2.370246000	0.351935000
C	0.195869000	1.346528000	0.072989000
H	-1.169400000	-2.235347000	1.090250000
H	-1.790840000	-3.058901000	-0.487074000

H	0.639858000	2.349295000	0.270651000
O	-1.135564000	1.357177000	-0.020618000
C	-1.877758000	2.553048000	0.283500000
H	-2.598825000	2.719570000	-0.539836000
H	-2.429445000	2.392790000	1.231929000
H	-1.194277000	3.424152000	0.375078000
H	-6.790397000	1.318613000	-0.316447000
H	-6.805853000	-2.918115000	-1.267850000

Catalyst 2, VI intermediate

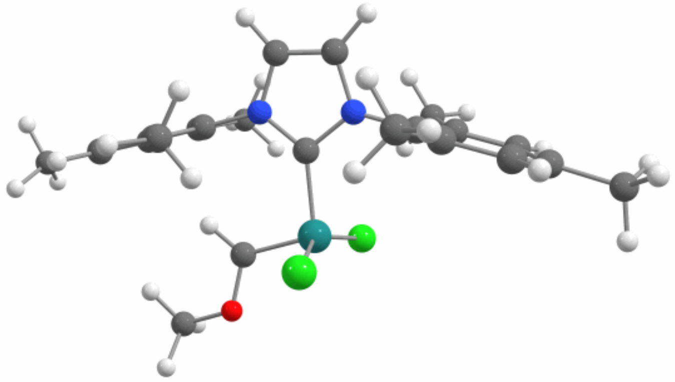

Zero-point correction= 0.444894 (Hartree/Particle)
Thermal correction to Energy= 0.479106
Thermal correction to Enthalpy= 0.480050
Thermal correction to Gibbs Free Energy= 0.374404
Sum of electronic and zero-point Energies= -2092.132448
Sum of electronic and thermal Energies= -2092.098236
Sum of electronic and thermal Enthalpies= -2092.097292
Sum of electronic and thermal Free Energies= -2092.202938

Solvent: -2092.92514393

Ru	-0.203429000	1.270513000	0.017566000
Cl	-0.764335000	1.708063000	-2.217983000
Cl	-0.503865000	1.615011000	2.319982000
N	0.904102000	-1.593623000	0.021518000
N	-1.279690000	-1.463983000	0.014382000
C	-0.132439000	-0.664508000	0.005681000
C	0.410605000	-2.905217000	0.037474000
H	1.085431000	-3.766604000	0.050255000
C	-0.950775000	-2.820742000	0.034435000
H	-1.724056000	-3.595264000	0.044350000
C	2.319624000	-1.332830000	0.008587000
C	2.986785000	-1.267152000	-1.239922000
C	2.222778000	-1.396182000	-2.536669000
H	2.902659000	-1.322629000	-3.407466000
H	1.452936000	-0.599496000	-2.627275000
H	1.685387000	-2.365976000	-2.602523000
C	4.377056000	-1.037062000	-1.229099000
H	4.909954000	-0.977805000	-2.193022000
C	5.099612000	-0.880245000	-0.027280000
C	6.599030000	-0.680541000	-0.046098000
H	7.128175000	-1.658827000	-0.071357000
H	6.953728000	-0.142224000	0.855837000
H	6.926698000	-0.113117000	-0.940850000
C	4.392872000	-0.949752000	1.191165000
H	4.939393000	-0.824217000	2.140906000
C	3.001916000	-1.177756000	1.239930000
C	2.260940000	-1.220134000	2.554824000
H	2.964368000	-1.163560000	3.408052000
H	1.666570000	-2.152213000	2.659814000
H	1.542412000	-0.374642000	2.638764000
C	-2.650278000	-0.998820000	0.004915000
C	-3.323842000	-0.818898000	1.241825000
C	-2.659105000	-1.125253000	2.561340000
H	-3.398871000	-1.106673000	3.385243000
H	-1.868757000	-0.376676000	2.788039000
H	-2.175158000	-2.124615000	2.554801000
C	-4.660014000	-0.373581000	1.199183000
H	-5.191947000	-0.219113000	2.152828000
C	-5.335866000	-0.141170000	-0.014741000
C	-6.752804000	0.386111000	-0.026803000
H	-7.315956000	0.030734000	-0.913613000
H	-6.758256000	1.498011000	-0.061239000
H	-7.310921000	0.085841000	0.883158000
C	-4.655148000	-0.414429000	-1.217770000
H	-5.182819000	-0.290471000	-2.178190000
C	-3.318830000	-0.858930000	-1.239739000
C	-2.643995000	-1.197843000	-2.546135000
H	-2.141035000	-2.186774000	-2.506481000
H	-1.870031000	-0.438473000	-2.790903000
H	-3.380252000	-1.218275000	-3.373139000
C	1.574518000	1.688190000	-0.071052000
H	2.445314000	1.010279000	-0.220310000
O	1.915663000	2.982038000	0.032753000
C	3.280164000	3.370493000	-0.203564000
H	3.403619000	3.673835000	-1.264194000
H	3.494854000	4.233673000	0.454564000
H	3.973834000	2.534889000	0.030803000

Catalyst 3, I intermediate

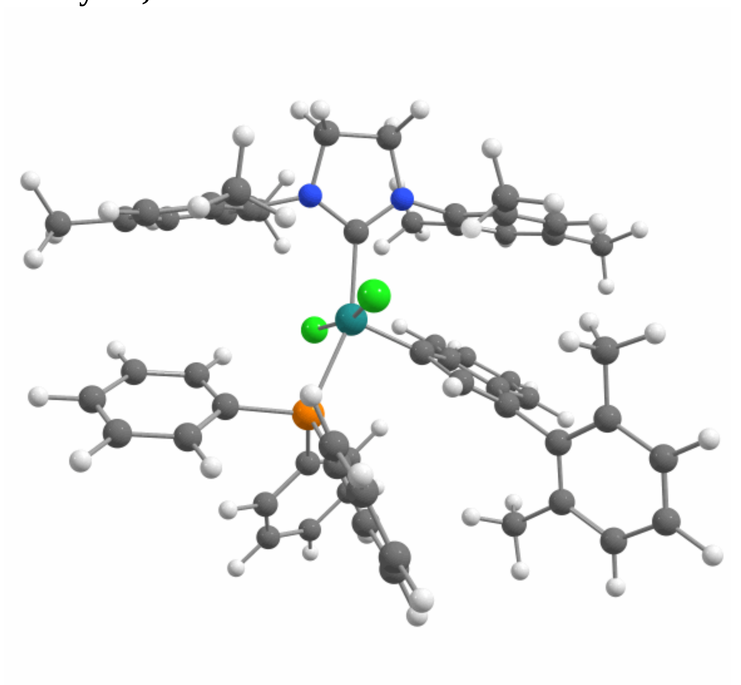

Zero-point correction= 0.926382 (Hartree/Particle)
Thermal correction to Energy= 0.988869
Thermal correction to Enthalpy= 0.989814
Thermal correction to Gibbs Free Energy= 0.826013
Sum of electronic and zero-point Energies= -3630.637629
Sum of electronic and thermal Energies= -3630.575141
Sum of electronic and thermal Enthalpies= -3630.574197
Sum of electronic and thermal Free Energies= -3630.737998

Solvent: -3632.25234193

Ru	-0,692398000	-0,363532000	-0,022507000
Cl	-1,467829000	-0,516576000	2,261795000
Cl	-0,446658000	-0,225905000	-2,442724000
P	-0,963640000	2,053244000	0,120891000
N	-2,505106000	-2,682508000	-0,795057000
N	-0,430068000	-3,415396000	-0,690725000
C	-1,226085000	-2,334780000	-0,452676000
C	-2,581096000	-4,018177000	-1,433009000
H	-2,794071000	-3,907609000	-2,518754000
H	-3,401684000	-4,613585000	-0,982420000
C	-1,188556000	-4,597475000	-1,165404000
H	-1,189078000	-5,387469000	-0,381219000
H	-0,712732000	-5,024932000	-2,071197000
C	-3,750096000	-1,985177000	-0,576540000
C	-4,423709000	-2,186186000	0,660427000
C	-3,817045000	-3,005060000	1,772963000
H	-4,573675000	-3,237917000	2,547826000
H	-2,981705000	-2,447885000	2,252198000
H	-3,398967000	-3,964142000	1,401527000
C	-5,705968000	-1,622798000	0,820975000
H	-6,229813000	-1,773182000	1,780076000
C	-6,345028000	-0,906566000	-0,208351000
C	-7,738697000	-0,346401000	-0,028830000
H	-7,795026000	0,712910000	-0,356360000
H	-8,070467000	-0,398602000	1,027461000
H	-8,478677000	-0,909294000	-0,638871000
C	-5,665271000	-0,761420000	-1,432765000
H	-6,152724000	-0,219656000	-2,260750000
C	-4,384791000	-1,305902000	-1,653562000
C	-3,762822000	-1,217834000	-3,026806000
H	-4,224813000	-0,399306000	-3,613810000
H	-3,933925000	-2,157972000	-3,598663000
H	-2,668349000	-1,040362000	-2,983586000
C	0,941200000	-3,642305000	-0,316916000
C	1,947723000	-3,605351000	-1,314847000
C	1,628252000	-3,215852000	-2,738025000
H	2,552604000	-3,133918000	-3,342634000
H	1,080449000	-2,250009000	-2,784130000
H	0,981078000	-3,973810000	-3,233143000
C	3,257784000	-3,973441000	-0,945013000
H	4,047639000	-3,953209000	-1,714630000
C	3,583190000	-4,379714000	0,363968000
C	5,004917000	-4,727165000	0,743643000
H	5,041473000	-5,555705000	1,480597000
H	5,510365000	-3,854007000	1,211659000
H	5,606370000	-5,022159000	-0,139829000
C	2,548365000	-4,432336000	1,318710000
H	2,777523000	-4,760908000	2,346055000
C	1,222430000	-4,081448000	1,002901000
C	0,126876000	-4,200586000	2,037489000
H	-0,589611000	-5,012406000	1,781350000
H	-0,464828000	-3,265748000	2,126779000
H	0,548564000	-4,440246000	3,033014000
H	1,785756000	0,885248000	-1,599178000
C	2,099694000	0,448216000	-0,644889000
C	3,377326000	0,474734000	-0,129159000
C	1,162429000	-0,227773000	0,278143000
C	3,353684000	-0,226959000	1,178514000
C	2,009896000	-0,638771000	1,451560000
C	4,390811000	-0,495864000	2,077979000
C	1,719938000	-1,268410000	2,667672000
C	4,086301000	-1,149207000	3,296180000
H	5,424748000	-0,195081000	1,842247000
C	2,764150000	-1,513539000	3,592137000
H	0,682870000	-1,540298000	2,906014000
H	4,891075000	-1,359569000	4,019147000
H	2,531634000	-2,001148000	4,552363000

C	4,583159000	1,107695000	-0,728111000
C	5,158689000	0,573989000	-1,918268000
C	5,159084000	2,257063000	-0,107488000
C	6,314484000	1,181944000	-2,450460000
C	6,309008000	2,838601000	-0,680319000
C	6,890876000	2,304455000	-1,839167000
H	6,766465000	0,760496000	-3,363544000
H	6,746579000	3,732794000	-0,206105000
H	7,792770000	2,768103000	-2,270228000
C	-2,756599000	2,499953000	-0,102513000
C	-3,198633000	3,502386000	-0,994191000
C	-3,696953000	1,860926000	0,739083000
C	-4,557570000	3,862425000	-1,039263000
H	-2,480840000	4,020607000	-1,646887000
C	-5,049225000	2,237336000	0,698875000
H	-3,361372000	1,078584000	1,440506000
C	-5,484808000	3,237138000	-0,188770000
H	-4,887262000	4,648800000	-1,737225000
H	-5,765739000	1,740664000	1,371001000
H	-6,546181000	3,532421000	-0,215436000
C	-0,062894000	3,133923000	-1,090378000
C	0,911112000	4,060902000	-0,654127000
C	-0,314870000	3,009589000	-2,478387000
C	1,616528000	4,845494000	-1,582924000
H	1,117701000	4,177060000	0,419985000
C	0,387575000	3,803993000	-3,400631000
H	-1,045437000	2,272028000	-2,838759000
C	1,356425000	4,721305000	-2,958109000
H	2,374125000	5,560255000	-1,224382000
H	0,178469000	3,695206000	-4,476872000
H	1,910076000	5,337341000	-3,684578000
C	-0,590440000	2,880828000	1,745150000
C	-1,252572000	4,075222000	2,110208000
C	0,388275000	2,345672000	2,607395000
C	-0,931445000	4,725486000	3,313357000
H	-2,026115000	4,501709000	1,452898000
C	0,710058000	3,002018000	3,808913000
H	0,889930000	1,402638000	2,348810000
C	0,051608000	4,190863000	4,165426000
H	-1,456713000	5,654586000	3,587208000
H	1,472973000	2,567857000	4,474155000
H	0,298904000	4,698834000	5,111471000
C	4,532501000	2,885021000	1,119029000
H	3,442777000	3,042893000	0,979738000
H	4,640214000	2,243825000	2,019178000
H	4,998728000	3,864597000	1,345688000
C	4,555489000	-0,627189000	-2,612096000
H	4,303952000	-1,433179000	-1,893259000
H	3,607930000	-0,366126000	-3,129063000
H	5,250708000	-1,036695000	-3,372451000

Catalyst 3, I-II TS

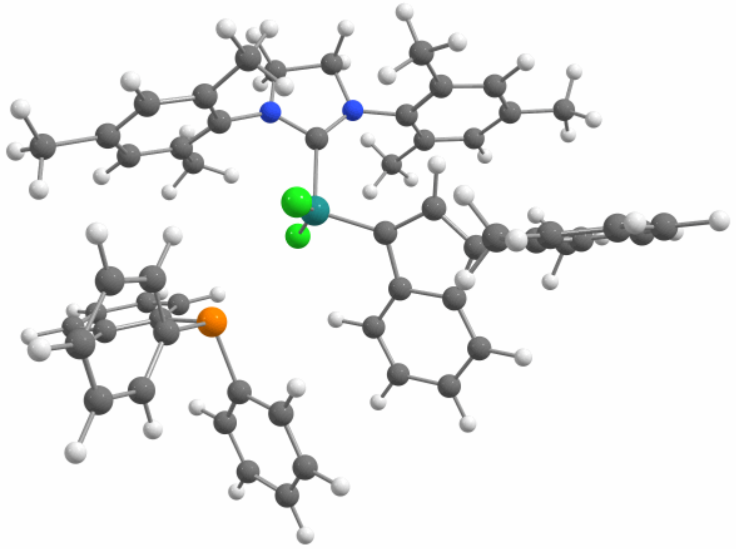

Zero-point correction= 0.925064 (Hartree/Particle)

Thermal correction to Energy= 0.988419

Ru	-0.095453	-0.626376	-0.224855
Cl	0.495224	-0.684776	-2.504213
Cl	0.302320	-0.330241	2.066966
P	3.135105	1.297588	-0.131389
N	0.209053	-3.473453	-0.152973
N	-1.894896	-3.060643	-0.716733
C	-0.725347	-2.463564	-0.303543
C	-0.264535	-4.765440	-0.679666
H	0.002344	-5.590577	0.012111
H	0.204171	-4.978779	-1.666808
C	-1.775387	-4.534863	-0.794170
H	-2.200693	-4.901953	-1.750328
H	-2.344531	-5.013852	0.034483
C	1.509381	-3.387715	0.462247
C	2.682481	-3.332413	-0.337240
C	2.634678	-3.397709	-1.845382
H	3.546697	-2.949502	-2.286300
H	1.759323	-2.863016	-2.262890
H	2.593143	-4.456394	-2.188038
C	3.931518	-3.294830	0.318081
H	4.843685	-3.224009	-0.297239
C	4.048280	-3.360911	1.718588
C	5.398950	-3.279581	2.392714
H	5.417867	-3.849136	3.344358
H	5.651565	-2.223943	2.636617
H	6.207479	-3.665824	1.739723
C	2.866135	-3.498261	2.475456
H	2.934999	-3.598974	3.571787
C	1.593549	-3.526092	1.876481
C	0.358539	-3.721728	2.722749
H	0.630421	-4.040046	3.748388
H	-0.320942	-4.487668	2.293355
H	-0.212905	-2.772310	2.799345
C	-3.227441	-2.536785	-0.864128
C	-4.118286	-2.617576	0.241000
C	-3.647209	-3.067514	1.607187
H	-4.347448	-2.729836	2.396520

Thermal correction to Enthalpy= 0.989363
Thermal correction to Gibbs Free Energy= 0.819336
Sum of electronic and zero-point Energies= -3630.616444
Sum of electronic and thermal Energies= -3630.553089
Sum of electronic and thermal Enthalpies= -3630.552145
Sum of electronic and thermal Free Energies= -3630.722172

Solvent: -3632.21954490

H	-2.638304	-2.676987	1.849712
H	-3.592789	-4.176738	1.680717
C	-5.466804	-2.268396	0.041801
H	-6.158877	-2.320084	0.899278
C	-5.953696	-1.856468	-1.215333
C	-7.418217	-1.527671	-1.401454
H	-8.047047	-2.439565	-1.305166
H	-7.616836	-1.087730	-2.398789
H	-7.772772	-0.810254	-0.632137
C	-5.041714	-1.781755	-2.285580
H	-5.400753	-1.461680	-3.278209
C	-3.680777	-2.125138	-2.142487
C	-2.751012	-2.083932	-3.329876
H	-2.349627	-3.093522	-3.567238
H	-1.868357	-1.437525	-3.141748
H	-3.276613	-1.712848	-4.231306
H	-3.159879	-0.439288	1.108218
C	-2.845089	0.413747	0.501068
C	-3.491676	1.635296	0.462843
C	-1.611748	0.459163	-0.281903
C	-2.716205	2.539159	-0.420816
C	-1.566583	1.827329	-0.889479
C	-2.943090	3.865102	-0.800686
C	-0.671398	2.446805	-1.770349
C	-2.028305	4.486176	-1.686963
H	-3.817442	4.416265	-0.417752
C	-0.914389	3.784436	-2.169866
H	0.203999	1.901227	-2.151710
H	-2.196795	5.529478	-1.998795
H	-0.209122	4.277441	-2.856348
C	-4.719189	2.031551	1.202914
C	-5.938254	2.232660	0.490932
C	-4.666242	2.229095	2.613766
C	-7.090612	2.615724	1.208588
C	-5.842018	2.618628	3.288857
C	-7.047273	2.808880	2.597206
H	-8.036133	2.763328	0.660651
H	-5.802350	2.780749	4.378667
H	-7.955667	3.112627	3.141980
C	3.971244	1.490532	1.520637
C	4.758903	2.612044	1.870615
C	3.794816	0.456817	2.469317
C	5.365049	2.692343	3.136451
H	4.895944	3.431624	1.147611
C	4.405977	0.539353	3.733516
H	3.154662	-0.405953	2.225924
C	5.191735	1.655344	4.070715
H	5.975522	3.572998	3.394429
H	4.249755	-0.268713	4.466253
H	5.661690	1.722421	5.065220
C	3.030316	3.070486	-0.685959
C	3.724255	3.597862	-1.797443
C	2.151601	3.918371	0.032032
C	3.553407	4.943574	-2.172772
H	4.410221	2.956328	-2.372196
C	1.996479	5.264365	-0.334520
H	1.586079	3.518516	0.890114
C	2.696214	5.782402	-1.440465
H	4.105258	5.339303	-3.040924
H	1.314411	5.910105	0.241227
H	2.569880	6.837425	-1.732001
C	4.509178	0.630692	-1.196494
C	5.860665	0.566970	-0.787996
C	4.159764	0.137987	-2.477030
C	6.841527	0.035985	-1.644640
H	6.149392	0.937530	0.207733
C	5.145751	-0.377590	-3.336538
H	3.103414	0.144427	-2.793333
C	6.489214	-0.432550	-2.922653
H	7.891076	-0.006361	-1.310597
H	4.858336	-0.748630	-4.333703
H	7.260208	-0.843818	-3.594048
C	-3.374587	2.055030	3.383901
H	-3.041605	0.996742	3.410539
H	-2.541523	2.622931	2.920247
H	-3.488179	2.400258	4.430832
C	-6.013524	2.015202	-1.005003
H	-5.408961	2.761332	-1.562598
H	-5.614980	1.018891	-1.289208
H	-7.058424	2.090505	-1.366385

Catalyst 3, II intermediate

Ru	-1.154419	-0.722485	-0.297766
Cl	-1.616556	-0.812247	-2.582423
Cl	-1.586901	-1.593167	1.808966
N	-3.035525	1.440626	-0.102601
N	-1.020523	2.328324	-0.353830
C	-1.686600	1.140215	-0.179947
C	-3.321674	2.852094	-0.414806
H	-4.033039	3.278370	0.322619
H	-3.783258	2.939491	-1.423781
C	-1.929157	3.496121	-0.354654
H	-1.714407	4.147592	-1.226473

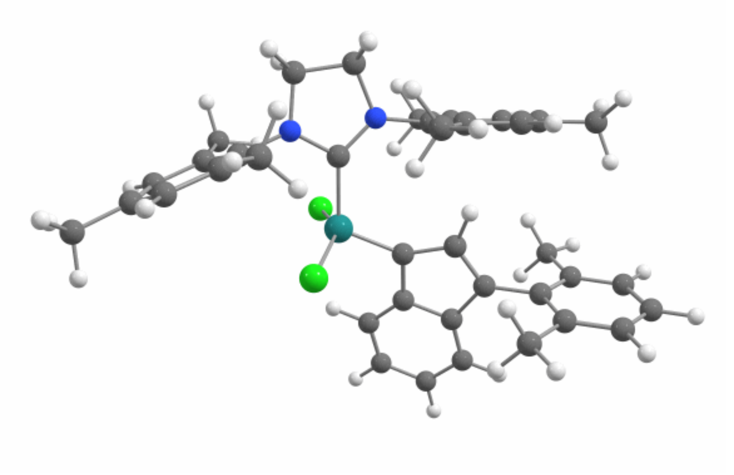

Zero-point correction= 0.657970 (Hartree/Particle)
Thermal correction to Energy= 0.704119
Thermal correction to Enthalpy= 0.705064
Thermal correction to Gibbs Free Energy= 0.573655
Sum of electronic and zero-point Energies= -2595.161486
Sum of electronic and thermal Energies= -2595.115337
Sum of electronic and thermal Enthalpies= -2595.114393
Sum of electronic and thermal Free Energies= -2595.245801

Solvent: -2596.25982851

H	-1.775207	4.105358	0.564783
C	-4.112153	0.558019	0.271174
C	-4.969539	0.005930	-0.717690
C	-4.847150	0.358570	-2.181450
H	-5.266417	-0.445322	-2.818430
H	-3.795419	0.515922	-2.488026
H	-5.423298	1.284011	-2.408445
C	-6.015621	-0.839603	-0.290832
H	-6.669833	-1.290960	-1.055742
C	-6.257838	-1.109409	1.068092
C	-7.356215	-2.055462	1.497377
H	-7.887409	-1.683512	2.397876
H	-6.938041	-3.052490	1.758419
H	-8.103504	-2.211173	0.693631
C	-5.442001	-0.471143	2.025102
H	-5.641739	-0.630372	3.098053
C	-4.379201	0.371660	1.657405
C	-3.560301	1.070266	2.716430
H	-4.045028	0.983761	3.708598
H	-3.427000	2.149705	2.492807
H	-2.550410	0.615613	2.790099
C	0.387327	2.608652	-0.331759
C	1.003635	2.914930	0.911161
C	0.255647	2.755963	2.215409
H	0.899497	3.022439	3.076013
H	-0.086793	1.708163	2.352756
H	-0.650753	3.396307	2.266475
C	2.352508	3.319501	0.904597
H	2.840251	3.552463	1.866058
C	3.096516	3.419959	-0.289691
C	4.555448	3.815546	-0.260040
H	4.890829	4.219427	-1.236373
H	5.196111	2.936856	-0.025830
H	4.755840	4.579700	0.518680
C	2.449300	3.108973	-1.502072
H	3.010421	3.189535	-2.448259
C	1.096960	2.711098	-1.553569
C	0.427978	2.410971	-2.873577
H	-0.398580	3.124550	-3.083885
H	-0.021068	1.395454	-2.884960
H	1.150398	2.485059	-3.709651
H	1.884182	0.750812	0.582146
C	1.870834	-0.269752	0.191167
C	2.966566	-1.115471	0.162652
C	0.693918	-0.953015	-0.326386
C	2.546618	-2.407766	-0.422072
C	1.156422	-2.317203	-0.745902
C	3.270768	-3.576964	-0.680200
C	0.506948	-3.401535	-1.352119
C	2.601790	-4.673274	-1.274464
H	4.341489	-3.639227	-0.426557
C	1.240781	-4.585051	-1.607989
H	-0.552397	-3.338801	-1.646098
H	3.156997	-5.601672	-1.483277
H	0.735217	-5.442197	-2.079758
C	4.347332	-0.815169	0.631514
C	5.322345	-0.367607	-0.304816
C	4.689462	-1.000439	2.000911
C	6.630065	-0.098641	0.151040
C	6.009291	-0.722132	2.414204
C	6.974144	-0.272882	1.500315
H	7.388125	0.247112	-0.571189
H	6.279621	-0.866659	3.473281
H	8.001027	-0.062683	1.840172
C	4.960403	-0.177181	-1.761640
H	4.575396	-1.116117	-2.212805
H	4.153765	0.576750	-1.881124
H	5.836374	0.153869	-2.354370
C	3.660588	-1.490791	2.996155
H	2.828558	-0.765487	3.116427
H	3.193102	-2.441755	2.664695
H	4.115068	-1.659029	3.992724

Catalyst 3, II-III TS

Ru	-0.978005000	0.361444000	-0.258441000
Cl	-1.721632000	1.140444000	1.808379000
Cl	-0.870643000	0.621410000	-2.590633000
N	-2.927177000	-1.705097000	-0.591468000
N	-0.937183000	-2.687478000	-0.574096000
C	-1.577884000	-1.483850000	-0.398573000
C	-3.211673000	-3.054945000	-1.108401000
H	-3.468562000	-3.011613000	-2.190590000
H	-4.071750000	-3.505307000	-0.571108000
C	-1.886678000	-3.789064000	-0.853759000
H	-1.943954000	-4.482068000	0.014893000
H	-1.540991000	-4.375414000	-1.729576000

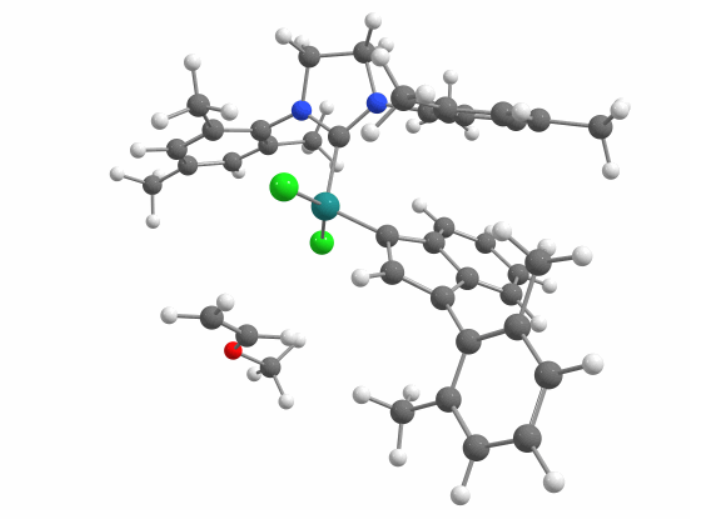

Zero-point correction= 0.740012 (Hartree/Particle)
Thermal correction to Energy= 0.792713
Thermal correction to Enthalpy= 0.793657
Thermal correction to Gibbs Free Energy= 0.645357
Sum of electronic and zero-point Energies= -2788.041511
Sum of electronic and thermal Energies= -2787.988810
Sum of electronic and thermal Enthalpies= -2787.987866
Sum of electronic and thermal Free Energies= -2788.136166

Solvent: -2789.31196880

C	-3.996004000	-0.774802000	-0.328833000
C	-4.491918000	-0.680416000	1.001589000
C	-3.920943000	-1.531287000	2.111099000
H	-4.540439000	-1.453475000	3.025862000
H	-2.893786000	-1.200381000	2.369993000
H	-3.867604000	-2.603044000	1.823996000
C	-5.542869000	0.215871000	1.260510000
H	-5.918051000	0.305640000	2.293722000
C	-6.128659000	0.995875000	0.242073000
C	-7.213794000	1.998863000	0.560917000
H	-7.841225000	2.222044000	-0.325388000
H	-6.770740000	2.960888000	0.900731000
H	-7.877105000	1.641909000	1.375439000
C	-5.664563000	0.820805000	-1.074259000
H	-6.135401000	1.391032000	-1.892602000
C	-4.616735000	-0.068400000	-1.393491000
C	-4.238599000	-0.294878000	-2.837996000
H	-4.531128000	0.571846000	-3.462816000
H	-4.768504000	-1.184180000	-3.247826000
H	-3.149042000	-0.447515000	-2.965871000
C	0.439145000	-3.048914000	-0.356473000
C	1.366633000	-2.961420000	-1.428448000
C	0.957344000	-2.447229000	-2.785986000
H	1.802525000	-2.501688000	-3.499681000
H	0.594433000	-1.399007000	-2.747548000
H	0.121583000	-3.045848000	-3.209559000
C	2.681778000	-3.416212000	-1.206225000
H	3.406631000	-3.349691000	-2.034821000
C	3.089902000	-3.973927000	0.021608000
C	4.517200000	-4.422059000	0.241953000
H	5.032830000	-4.636056000	-0.715832000
H	4.568444000	-5.332904000	0.873258000
H	5.103695000	-3.633770000	0.763470000
C	2.129635000	-4.092634000	1.043525000
H	2.418991000	-4.545777000	2.006655000
C	0.802994000	-3.649796000	0.877978000
C	-0.198175000	-3.873693000	1.990251000
H	-0.667767000	-4.879988000	1.909765000
H	-1.014631000	-3.125722000	1.981843000
H	0.295783000	-3.833152000	2.981167000
H	1.569465000	1.666388000	-1.528001000
C	1.802606000	1.154486000	-0.585437000
C	3.047099000	1.111123000	0.019944000
C	0.844950000	0.316315000	0.154467000
C	2.927473000	0.288561000	1.248767000
C	1.583540000	-0.195999000	1.333892000
C	3.840823000	0.053045000	2.281552000
C	1.148836000	-0.850793000	2.488884000
C	3.404889000	-0.659633000	3.425732000
H	4.870020000	0.442745000	2.219470000
C	2.073250000	-1.090839000	3.534559000
H	0.096127000	-1.149789000	2.585003000
H	4.111476000	-0.851001000	4.249248000
H	1.740615000	-1.612037000	4.446409000
C	4.296618000	1.779026000	-0.432546000
C	5.334301000	1.007890000	-1.031641000
C	4.456191000	3.183213000	-0.249083000
C	6.519136000	1.655089000	-1.437073000
C	5.660438000	3.789131000	-0.663840000
C	6.685680000	3.035790000	-1.254169000
H	7.319409000	1.060575000	-1.907871000
H	5.791195000	4.873583000	-0.514685000
H	7.618966000	3.527155000	-1.572732000
C	-1.220487000	4.338977000	-0.178697000
C	-1.599976000	4.282258000	-1.472229000
H	-0.987491000	3.729780000	-2.196929000
H	-2.534733000	4.754891000	-1.811017000
H	-0.288153000	3.856867000	0.176543000
O	-1.951651000	4.973799000	0.776485000
C	-1.388564000	4.980123000	2.084366000
H	-2.123963000	5.474146000	2.748274000
H	-1.205410000	3.946405000	2.452511000
H	-0.435954000	5.558614000	2.117216000
C	3.367889000	4.020175000	0.388375000
H	2.485052000	4.118931000	-0.278259000
H	3.000716000	3.568079000	1.333042000
H	3.733041000	5.042154000	0.612368000
C	5.150584000	-0.473353000	-1.272690000
H	4.890819000	-1.015698000	-0.339707000
H	4.313484000	-0.661569000	-1.977969000
H	6.067233000	-0.929229000	-1.696849000

Catalyst 3, III intermediate

Ru	1.199561000	0.562908000	0.030448000
Cl	1.909858000	0.872181000	-2.242886000
Cl	0.776756000	0.330028000	2.433811000
N	2.925035000	-1.797900000	-0.062384000
N	0.861352000	-2.525336000	-0.344074000
C	1.609211000	-1.406813000	-0.138656000
C	3.107967000	-3.256823000	-0.200618000

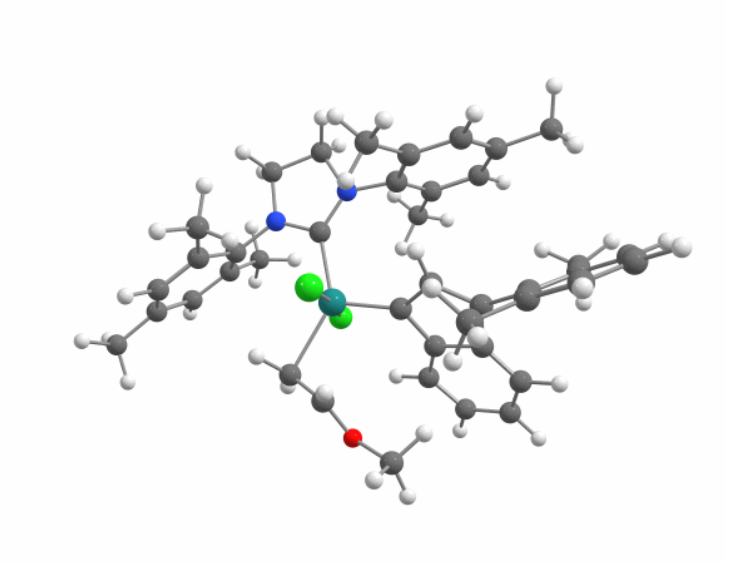

Zero-point correction= 0.742808 (Hartree/Particle)
Thermal correction to Energy= 0.794283
Thermal correction to Enthalpy= 0.795228
Thermal correction to Gibbs Free Energy= 0.655508
Sum of electronic and zero-point Energies= -2788.057814
Sum of electronic and thermal Energies= -2788.006339
Sum of electronic and thermal Enthalpies= -2788.005395
Sum of electronic and thermal Free Energies= -2788.145115

Solvent: -2789.33319311

H	3.529500000	-3.683205000	0.736362000
H	3.819017000	-3.483086000	-1.022214000
C	1.683051000	-3.750528000	-0.493420000
H	1.568115000	-4.155897000	-1.522409000
H	1.339116000	-4.533839000	0.213765000
C	4.074361000	-0.963661000	0.195785000
C	4.895296000	-0.551398000	-0.892640000
C	4.644152000	-1.025146000	-2.303797000
H	5.203269000	-0.406135000	-3.032413000
H	3.569244000	-0.968553000	-2.565172000
H	4.990810000	-2.075429000	-2.433530000
C	6.008516000	0.264813000	-0.614025000
H	6.631900000	0.607075000	-1.457198000
C	6.356913000	0.639554000	0.698850000
C	7.528196000	1.559705000	0.959257000
H	7.917914000	1.446734000	1.990989000
H	7.230661000	2.624374000	0.834814000
H	8.361386000	1.373375000	0.250825000
C	5.589220000	0.119350000	1.758117000
H	5.886485000	0.336400000	2.798001000
C	4.464713000	-0.704478000	1.540800000
C	3.753554000	-1.326825000	2.717078000
H	4.325662000	-1.157517000	3.650315000
H	3.635436000	-2.423665000	2.588783000
H	2.734630000	-0.901514000	2.850876000
C	-0.559406000	-2.691377000	-0.478161000
C	-1.295298000	-3.129437000	0.654853000
C	-0.648633000	-3.215790000	2.018302000
H	-1.389635000	-3.504520000	2.789316000
H	-0.194045000	-2.244202000	2.313370000
H	0.167554000	-3.970790000	2.047084000
C	-2.650059000	-3.463482000	0.477062000
H	-3.231523000	-3.800091000	1.351860000
C	-3.278723000	-3.386327000	-0.783129000
C	-4.730164000	-3.781624000	-0.938142000
H	-5.070232000	-3.691931000	-1.988792000
H	-5.387366000	-3.142536000	-0.310444000
H	-4.897952000	-4.831246000	-0.614474000
C	-2.518549000	-2.933721000	-1.879063000
H	-2.992601000	-2.864670000	-2.872643000
C	-1.157281000	-2.583245000	-1.757361000
C	-0.364125000	-2.119915000	-2.956153000
H	0.462255000	-2.823641000	-3.198585000
H	0.109284000	-1.129845000	-2.781302000
H	-1.009599000	-2.047030000	-3.853082000
H	-1.601151000	-0.529349000	1.282306000
C	-1.743270000	0.237397000	0.516540000
C	-2.947955000	0.829183000	0.191755000
C	-0.644355000	0.792998000	-0.285837000
C	-2.684366000	1.839416000	-0.861565000
C	-1.290703000	1.797339000	-1.195240000
C	-3.536611000	2.781418000	-1.447536000
C	-0.787724000	2.672722000	-2.166599000
C	-3.016330000	3.661769000	-2.427864000
H	-4.594789000	2.841425000	-1.145465000
C	-1.663810000	3.594395000	-2.792006000
H	0.276493000	2.634019000	-2.436951000
H	-3.680608000	4.400055000	-2.905517000
H	-1.268103000	4.274222000	-3.563381000
C	-4.275512000	0.567919000	0.805558000
C	-5.356863000	0.094488000	0.000331000
C	-4.472016000	0.800178000	2.201353000
C	-6.607510000	-0.144499000	0.607969000
C	-5.743217000	0.556534000	2.761434000
C	-6.805610000	0.085244000	1.977012000
H	-7.437950000	-0.522164000	-0.011658000
H	-5.897684000	0.748233000	3.836144000
H	-7.790912000	-0.102129000	2.433354000
C	2.302195000	2.482891000	0.422658000
C	1.162270000	3.151005000	0.852114000
H	2.902043000	1.975508000	1.201090000
H	2.822230000	2.824662000	-0.486453000
H	0.688047000	2.908802000	1.822728000
O	0.638261000	4.156175000	0.139501000
C	-0.599394000	4.704804000	0.616780000
H	-0.517237000	4.982073000	1.690165000
H	-0.794734000	5.610021000	0.013968000
H	-1.429055000	3.982221000	0.471300000
C	-3.361552000	1.326322000	3.086628000
H	-2.588360000	0.558663000	3.298131000
H	-2.823191000	2.172212000	2.612694000
H	-3.765560000	1.671659000	4.059278000
C	-5.183991000	-0.188801000	-1.476939000
H	-5.184787000	0.741796000	-2.082558000
H	-4.220738000	-0.697539000	-1.684431000
H	-6.006726000	-0.828577000	-1.854002000

Catalyst 3, III-IV TS

Ru	-0,869796000	-0,314341000	-0,600727000
Cl	-1,366072000	0,961840000	-2,616383000
Cl	-0,921219000	-1,621020000	1,450022000
N	-3,150450000	1,183246000	0,773533000
N	-1,277436000	2,345939000	0,869551000

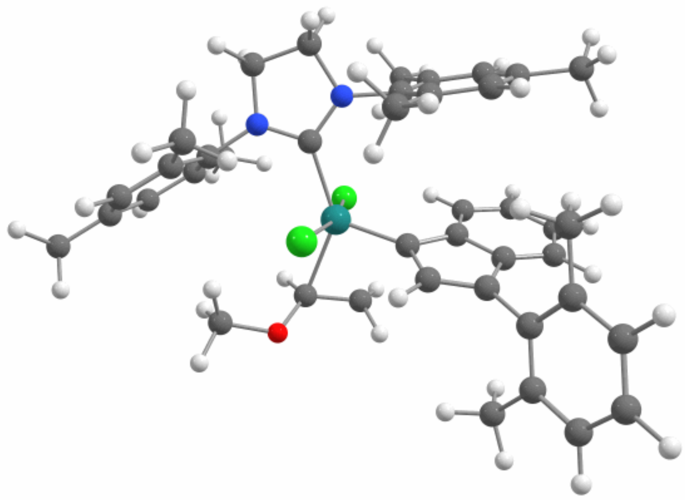

Zero-point correction= 0.742864 (Hartree/Particle)
Thermal correction to Energy= 0.793296
Thermal correction to Enthalpy= 0.794240
Thermal correction to Gibbs Free Energy= 0.657281
Sum of electronic and zero-point Energies= -2788.049791
Sum of electronic and thermal Energies= -2787.999359
Sum of electronic and thermal Enthalpies= -2787.998415
Sum of electronic and thermal Free Energies= -2788.135374

Solvent: -2789.32549123

C	-1,819905000	1,167222000	0,463267000
C	-3,584059000	2,500941000	1,297222000
H	-4,229600000	2,372527000	2,189259000
H	-4,173033000	3,041298000	0,523000000
C	-2,249049000	3,188927000	1,606283000
H	-2,205021000	4,237525000	1,249988000
H	-2,002515000	3,187345000	2,692160000
C	-4,133959000	0,138175000	0,615892000
C	-4,903410000	0,054619000	-0,575299000
C	-4,751763000	1,048053000	-1,702591000
H	-5,305720000	0,710168000	-2,600588000
H	-3,692185000	1,208521000	-1,991902000
H	-5,174119000	2,037259000	-1,416544000
C	-5,889056000	-0,953349000	-0,658998000
H	-6,480519000	-1,031208000	-1,586906000
C	-6,160799000	-1,831558000	0,405965000
C	-7,211677000	-2,912935000	0,285157000
H	-6,745315000	-3,918925000	0,200566000
H	-7,849950000	-2,766478000	-0,608903000
H	-7,870745000	-2,941378000	1,177984000
C	-5,431307000	-1,665194000	1,601417000
H	-5,655714000	-2,311711000	2,466618000
C	-4,430689000	-0,684844000	1,739813000
C	-3,722119000	-0,501974000	3,059449000
H	-4,104564000	-1,216503000	3,814289000
H	-3,867037000	0,522882000	3,465200000
H	-2,630676000	-0,668281000	2,936904000
C	0,097453000	2,773463000	0,813252000
C	0,999086000	2,352260000	1,824746000
C	0,578645000	1,387089000	2,907247000
H	1,387355000	1,255495000	3,652591000
H	0,314814000	0,387374000	2,495989000
H	-0,321000000	1,745853000	3,452343000
C	2,306332000	2,879691000	1,803057000
H	3,013469000	2,564266000	2,587927000
C	2,727801000	3,805374000	0,829173000
C	4,147875000	4,324214000	0,805421000
H	4,189282000	5,393268000	0,511346000
H	4,758420000	3,760235000	0,066280000
H	4,641657000	4,217713000	1,792334000
C	1,793576000	4,219970000	-0,140344000
H	2,097561000	4,954480000	-0,904917000
C	0,474881000	3,727246000	-0,168168000
C	-0,502292000	4,211782000	-1,213160000
H	-1,352817000	4,768407000	-0,761208000
H	-0,934940000	3,361105000	-1,782702000
H	-0,006262000	4,898393000	-1,926946000
H	1,418296000	-2,177080000	0,803185000
C	1,861542000	-1,435087000	0,129727000
C	3,200919000	-1,232864000	-0,087880000
C	1,039696000	-0,503486000	-0,692108000
C	3,348351000	-0,139756000	-1,081060000
C	2,046048000	0,303499000	-1,471796000
C	4,503238000	0,417702000	-1,638479000
C	1,921541000	1,313893000	-2,431617000
C	4,364717000	1,437019000	-2,610588000
H	5,499274000	0,060449000	-1,330245000
C	3,090489000	1,873434000	-3,002932000
H	0,922762000	1,651608000	-2,743784000
H	5,263772000	1,883992000	-3,064980000
H	2,992422000	2,662056000	-3,765804000
C	4,341798000	-1,988961000	0,502567000
C	5,064395000	-1,444758000	1,601359000
C	4,714611000	-3,246157000	-0,052176000
C	6,151472000	-2,170084000	2,131191000
C	5,808996000	-3,939067000	0,506151000
C	6,524526000	-3,408958000	1,589749000
H	6,709104000	-1,751070000	2,985044000
H	6,101847000	-4,910958000	0,075667000
H	7,377506000	-3,963279000	2,013011000
C	0,005310000	-1,827163000	-2,127171000
C	-1,328182000	-2,142043000	-1,720231000
H	0,143247000	-1,230045000	-3,040945000
H	0,772409000	-2,582061000	-1,897034000
H	-2,178057000	-1,720988000	-2,301918000
O	-1,552773000	-3,341839000	-1,129677000
C	-2,861810000	-3,549571000	-0,594586000
H	-3,645305000	-3,235757000	-1,321142000
H	-2,954934000	-4,634118000	-0,396974000
H	-2,977620000	-2,981700000	0,352316000
C	3,957374000	-3,836734000	-1,222345000
H	2,920642000	-4,114917000	-0,936468000
H	3,872625000	-3,114822000	-2,061691000
H	4,458559000	-4,748890000	-1,602704000
C	4,654296000	-0,123367000	2,210755000
H	4,600788000	0,681931000	1,448668000
H	3,641604000	-0,189636000	2,661960000
H	5,363232000	0,193154000	3,001648000

Catalyst 3, IV intermediate

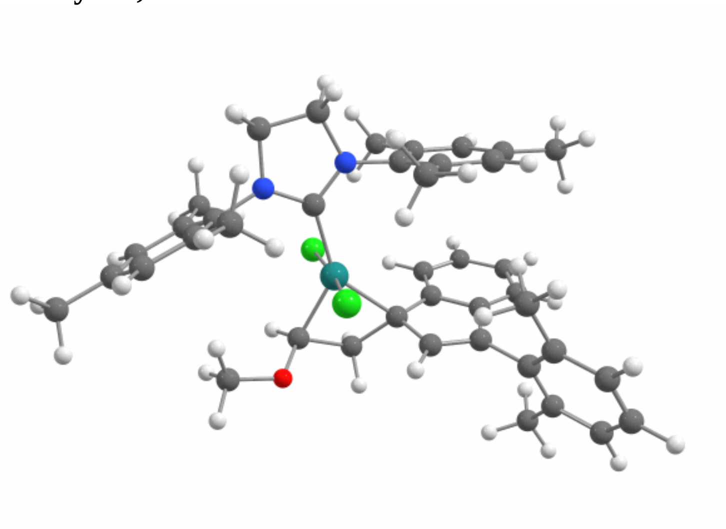

Zero-point correction= 0.744022 (Hartree/Particle)
Thermal correction to Energy= 0.794859
Thermal correction to Enthalpy= 0.795803
Thermal correction to Gibbs Free Energy= 0.656838
Sum of electronic and zero-point Energies= -2788.060418
Sum of electronic and thermal Energies= -2788.009582
Sum of electronic and thermal Enthalpies= -2788.008638
Sum of electronic and thermal Free Energies= -2788.147602

Solvent: -2789.33529889

Ru	-0.865935	-0.290061	-0.605633
Cl	-1.457508	0.982188	-2.592436
Cl	-0.622915	-1.542346	1.463885
N	-3.149924	1.143139	0.792311
N	-1.245951	2.255424	0.973476
C	-1.818155	1.116439	0.504914
C	-3.558503	2.432892	1.400465
H	-4.165750	2.252267	2.310944
H	-4.183171	3.008935	0.683357
C	-2.213395	3.115419	1.693835
H	-2.160399	4.155179	1.312217
H	-1.966002	3.138942	2.778178
C	-4.154494	0.124140	0.594621
C	-4.974075	0.152323	-0.565091
C	-4.823166	1.211867	-1.629452
H	-5.464078	0.983761	-2.503699
H	-3.774115	1.306598	-1.981894
H	-5.137735	2.208987	-1.248710
C	-5.993894	-0.817533	-0.679301
H	-6.624937	-0.808819	-1.583908
C	-6.247819	-1.766207	0.328090
C	-7.343533	-2.798472	0.179558
H	-8.001415	-2.822869	1.073730
H	-6.921462	-3.820840	0.066939
H	-7.977722	-2.599041	-0.706993
C	-5.458938	-1.716940	1.496083
H	-5.664545	-2.422668	2.318654
C	-4.422493	-0.778090	1.663230
C	-3.641873	-0.729647	2.952569
H	-4.059096	-1.439277	3.693377
H	-3.660039	0.283712	3.408301
H	-2.575732	-0.989837	2.770265
C	0.144837	2.642959	0.939304
C	0.993144	2.260484	2.012410
C	0.496426	1.402808	3.150920
H	1.277788	1.290254	3.927433
H	0.202793	0.388859	2.798434
H	-0.397819	1.843997	3.641964
C	2.323823	2.725210	1.997701
H	2.990540	2.438265	2.827835
C	2.818915	3.556121	0.974226
C	4.264158	3.997796	0.958300
H	4.374421	5.028782	0.563857
H	4.865267	3.331411	0.301362
H	4.717318	3.961946	1.969494
C	1.931078	3.954633	-0.043787
H	2.288319	4.630093	-0.838661
C	0.589170	3.529223	-0.078215
C	-0.340540	4.030323	-1.156591
H	-1.209574	4.578287	-0.730551
H	-0.751329	3.193032	-1.761254
H	0.187828	4.727536	-1.835709
H	1.489073	-2.546409	0.312972
C	1.890812	-1.693303	-0.245365
C	3.197260	-1.282421	-0.257294
C	1.052065	-0.873977	-1.171960
C	3.306340	-0.136723	-1.190846
C	2.027309	0.103131	-1.780531
C	4.420645	0.635513	-1.550131
C	1.886941	1.097637	-2.757289
C	4.267916	1.636797	-2.533633
H	5.400307	0.447509	-1.081386
C	3.017492	1.855704	-3.136841
H	0.901707	1.295913	-3.205057
H	5.136906	2.244179	-2.834823
H	2.910866	2.632368	-3.910915
C	4.335686	-1.901181	0.477897
C	4.429506	-1.768510	1.893457
C	5.322950	-2.638010	-0.240860
C	5.525986	-2.352133	2.561932
C	6.401475	-3.207094	0.467600
C	6.509531	-3.062505	1.858521
H	5.604000	-2.242250	3.656299
H	7.161996	-3.781082	-0.087321
H	7.359988	-3.510838	2.397060
C	0.083901	-1.711184	-2.106182
C	-1.426980	-1.888247	-1.657951
H	0.096089	-1.284532	-3.125805
H	0.468040	-2.749006	-2.072362
H	-2.147684	-1.613173	-2.462920
O	-1.654908	-3.130718	-1.130377
C	-3.023020	-3.471739	-0.925736
H	-3.632669	-3.248488	-1.831666
H	-3.054900	-4.558496	-0.719867
H	-3.444074	-2.919367	-0.059235
C	3.380032	-1.011527	2.676020
H	2.396540	-1.526635	2.657963
H	3.684530	-0.891040	3.735111
H	3.200367	-0.005178	2.244731
C	5.200110	-2.858689	-1.733298
H	4.233795	-3.343782	-1.988275
H	5.226883	-1.905177	-2.301117
H	6.017840	-3.504555	-2.110391

Catalyst 3, IV-V TS

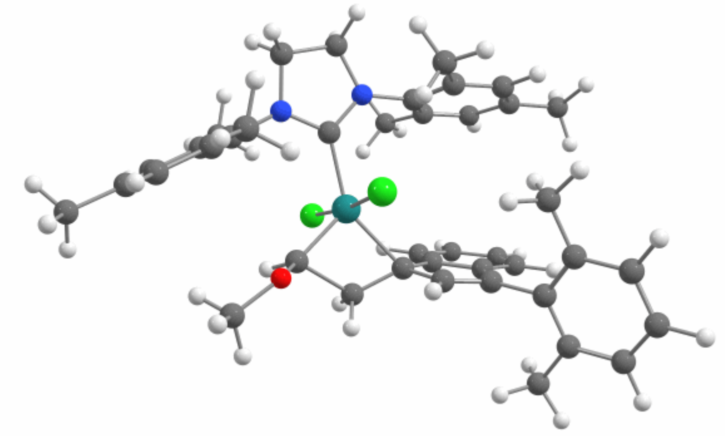

Zero-point correction= 0.742837 (Hartree/Particle)
Thermal correction to Energy= 0.793323
Thermal correction to Enthalpy= 0.794267
Thermal correction to Gibbs Free Energy= 0.656215
Sum of electronic and zero-point Energies= -2788.058980
Sum of electronic and thermal Energies= -2788.008494
Sum of electronic and thermal Enthalpies= -2788.007550
Sum of electronic and thermal Free Energies= -2788.145601

Solvent: -2789.33283941

Ru	0.847701000	-0.397512000	0.366194000
Cl	1.709346000	0.200482000	2.560048000
Cl	0.167582000	-0.848170000	-1.921560000
N	3.159267000	1.272279000	-0.699052000
N	1.265608000	2.408330000	-0.780040000
C	1.823041000	1.216436000	-0.440608000
C	3.544562000	2.540280000	-1.363711000
H	3.806870000	2.343462000	-2.426925000
H	4.433767000	2.985118000	-0.872748000
C	2.279206000	3.398102000	-1.214363000
H	2.386088000	4.191933000	-0.442085000
H	1.967198000	3.885503000	-2.160089000
C	4.155308000	0.243148000	-0.521045000
C	5.006762000	0.306879000	0.616923000
C	4.866120000	1.396556000	1.651987000
H	5.618968000	1.274780000	2.455204000
H	3.855703000	1.372711000	2.112380000
H	5.012825000	2.406639000	1.211293000
C	6.009642000	-0.672223000	0.752546000
H	6.663756000	-0.632865000	1.640049000
C	6.210501000	-1.680282000	-0.212725000
C	7.308339000	-2.707130000	-0.038269000
H	7.193989000	-3.266727000	0.914714000
H	8.309115000	-2.225007000	-0.004934000
H	7.317364000	-3.443124000	-0.866731000
C	5.383405000	-1.677512000	-1.351198000
H	5.545544000	-2.433207000	-2.138268000
C	4.362196000	-0.721347000	-1.542130000
C	3.552524000	-0.723474000	-2.814207000
H	3.894258000	-1.527225000	-3.495288000
H	3.652172000	0.239028000	-3.361770000
H	2.467179000	-0.868525000	-2.616803000
C	-0.106227000	2.828675000	-0.617388000
C	-0.948853000	2.862747000	-1.760293000
C	-0.464811000	2.407137000	-3.115214000
H	-1.263508000	2.514695000	-3.874745000
H	-0.152257000	1.341001000	-3.075577000
H	0.409530000	2.997489000	-3.466305000
C	-2.263666000	3.343644000	-1.598652000
H	-2.925328000	3.370629000	-2.480455000
C	-2.752706000	3.790897000	-0.355980000
C	-4.183279000	4.254034000	-0.198742000
H	-4.260415000	5.118923000	0.491976000
H	-4.813342000	3.442721000	0.227277000
H	-4.630939000	4.544973000	-1.170226000
C	-1.872467000	3.783489000	0.743311000
H	-2.223342000	4.159190000	1.718763000
C	-0.542497000	3.331013000	0.638823000
C	0.386392000	3.433135000	1.824984000
H	1.256955000	4.089326000	1.604654000
H	0.796589000	2.447040000	2.131131000
H	-0.141359000	3.868462000	2.695719000
H	-1.663723000	-2.498584000	-0.850170000
C	-1.965307000	-1.817829000	-0.045902000
C	-3.237369000	-1.368749000	0.191517000
C	-1.016625000	-1.317415000	0.987119000
C	-3.202349000	-0.515712000	1.401128000
C	-1.868465000	-0.491175000	1.914699000
C	-4.237707000	0.167725000	2.054950000
C	-1.592595000	0.196142000	3.104661000
C	-3.948167000	0.862651000	3.249155000
H	-5.261460000	0.144608000	1.647495000
C	-2.643054000	0.866765000	3.771056000
H	-0.565947000	0.232576000	3.499016000
H	-4.752924000	1.398246000	3.778483000
H	-2.429542000	1.403540000	4.709189000
C	-4.465294000	-1.700082000	-0.584415000
C	-4.687932000	-1.093322000	-1.853678000
C	-5.408579000	-2.626535000	-0.052832000
C	-5.861440000	-1.418817000	-2.565889000
C	-6.567391000	-2.925376000	-0.799071000
C	-6.796878000	-2.325648000	-2.046286000
H	-6.038803000	-0.946877000	-3.546512000
H	-7.294863000	-3.647242000	-0.392198000
H	-7.707827000	-2.568326000	-2.617018000
C	0.085823000	-2.228876000	1.436317000
C	1.704547000	-2.077377000	0.637742000
H	0.308886000	-2.170953000	2.516601000
H	-0.081843000	-3.255268000	1.063433000
H	2.414523000	-2.157898000	1.495770000
O	1.923042000	-2.999515000	-0.329942000
C	2.783393000	-4.090351000	0.023350000
H	2.303870000	-4.748278000	0.782762000
H	2.953037000	-4.671800000	-0.902118000
H	3.758435000	-3.723000000	0.410281000
C	-5.160531000	-3.313564000	1.272793000
H	-4.202144000	-3.875009000	1.261713000
H	-5.084153000	-2.586537000	2.108711000
H	-5.973713000	-4.027158000	1.513208000
C	-3.694228000	-0.109156000	-2.427436000
H	-3.487114000	0.720168000	-1.718941000
H	-2.708094000	-0.578738000	-2.626877000
H	-4.069115000	0.327654000	-3.374866000

Catalyst 3, V intermediate

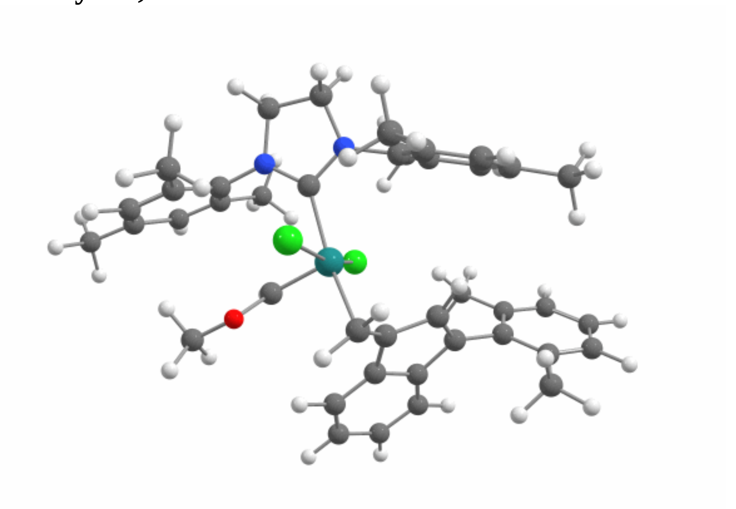

Zero-point correction= 0.742131 (Hartree/Particle)
Thermal correction to Energy= 0.792981
Thermal correction to Enthalpy= 0.793925
Thermal correction to Gibbs Free Energy= 0.653931
Sum of electronic and zero-point Energies= -2788.075157
Sum of electronic and thermal Energies= -2788.024306
Sum of electronic and thermal Enthalpies= -2788.023362
Sum of electronic and thermal Free Energies= -2788.163357

Solvent: -2789.34908896

Ru	0.664504000	-0.065641000	-0.736393000
Cl	-0.151347000	-0.712434000	1.456484000
Cl	1.862652000	0.905864000	-2.648412000
N	3.078678000	1.445053000	0.660748000
N	1.241689000	2.639173000	0.407682000
C	1.788992000	1.404693000	0.233613000
C	3.444108000	2.770494000	1.221631000
H	4.388549000	3.134869000	0.769878000
H	3.604943000	2.682943000	2.319053000
C	2.229954000	3.642622000	0.861511000
H	1.834113000	4.220054000	1.721777000
H	2.447521000	4.357827000	0.038421000
C	4.041142000	0.377736000	0.779318000
C	4.030998000	-0.438233000	1.945440000
C	2.992520000	-0.263321000	3.025825000
H	3.206411000	-0.924447000	3.888203000
H	1.968171000	-0.490938000	2.653900000
H	2.965165000	0.781303000	3.403795000
C	5.037054000	-1.415548000	2.082009000
H	5.028452000	-2.053180000	2.982259000
C	6.060638000	-1.580237000	1.126352000
C	7.140184000	-2.622129000	1.322451000
H	7.820461000	-2.677846000	0.449490000
H	6.707576000	-3.632211000	1.484648000
H	7.759206000	-2.394391000	2.217056000
C	6.059609000	-0.728217000	0.005458000
H	6.861077000	-0.823505000	-0.746375000
C	5.073915000	0.264180000	-0.188498000
C	5.148990000	1.183561000	-1.382587000
H	5.956944000	0.868696000	-2.071720000
H	5.369652000	2.230449000	-1.076788000
H	4.187073000	1.198802000	-1.939308000
C	-0.140346000	3.007627000	0.195542000
C	-0.549640000	3.598487000	-1.031402000
C	0.432057000	3.974556000	-2.114620000
H	-0.096043000	4.201343000	-3.061775000
H	1.165306000	3.166115000	-2.315296000
H	0.992915000	4.893128000	-1.829725000
C	-1.917762000	3.912407000	-1.188071000
H	-2.247277000	4.347766000	-2.146456000
C	-2.859550000	3.716476000	-0.161216000
C	-4.324548000	4.030328000	-0.363239000
H	-4.720965000	4.662516000	0.458955000
H	-4.931892000	3.099558000	-0.370098000
H	-4.503754000	4.558685000	-1.320690000
C	-2.392768000	3.234631000	1.079198000
H	-3.100610000	3.125337000	1.917552000
C	-1.044732000	2.893101000	1.291453000
C	-0.580407000	2.450147000	2.656776000
H	0.294164000	3.039199000	3.006577000
H	-0.280974000	1.379909000	2.636125000
H	-1.389406000	2.571348000	3.403151000
H	-2.625317000	0.602153000	-1.187452000
C	-2.457583000	-0.462417000	-0.968346000
C	-3.300328000	-1.279042000	-0.247276000
C	-1.287175000	-1.221313000	-1.448132000
C	-2.705494000	-2.633157000	-0.216168000
C	-1.475399000	-2.606364000	-0.938773000
C	-3.186488000	-3.837627000	0.326508000
C	-0.734590000	-3.781097000	-1.118926000
C	-2.425885000	-5.010716000	0.157378000
H	-4.148148000	-3.862777000	0.864024000
C	-1.212459000	-4.984391000	-0.559760000
H	0.204708000	-3.771745000	-1.695508000
H	-2.787236000	-5.961439000	0.581411000
H	-0.637357000	-5.914763000	-0.693273000
C	-4.625482000	-0.916621000	0.332403000
C	-4.791597000	-0.819543000	1.745226000
C	-5.733367000	-0.688322000	-0.536321000
C	-6.059676000	-0.475690000	2.260757000
C	-6.984641000	-0.352917000	0.022620000
C	-7.150358000	-0.242236000	1.411073000
H	-6.185204000	-0.390950000	3.353103000
H	-7.842730000	-0.183813000	-0.648959000
H	-8.134152000	0.021633000	1.831870000
C	-0.440817000	-0.807757000	-2.499819000
C	1.826132000	-1.481438000	-0.608995000
H	0.179386000	-1.540670000	-3.040070000
H	-0.701711000	0.090434000	-3.082267000
H	1.942938000	-2.020484000	0.365379000
O	2.521483000	-2.018067000	-1.606405000
C	3.390062000	-3.131133000	-1.298853000
H	2.878171000	-3.858499000	-0.634468000
H	3.637393000	-3.610712000	-2.263806000
H	4.314735000	-2.756635000	-0.814868000
C	-5.594887000	-0.815645000	-2.039618000
H	-5.004311000	0.018483000	-2.474151000
H	-5.068581000	-1.749784000	-2.324957000
H	-6.588968000	-0.812722000	-2.530428000
C	-3.636087000	-1.069323000	2.687189000
H	-3.390026000	-2.151508000	2.749430000
H	-2.706368000	-0.572777000	2.341515000
H	-3.874530000	-0.721574000	3.712509000

Catalyst 3, V-IV TS

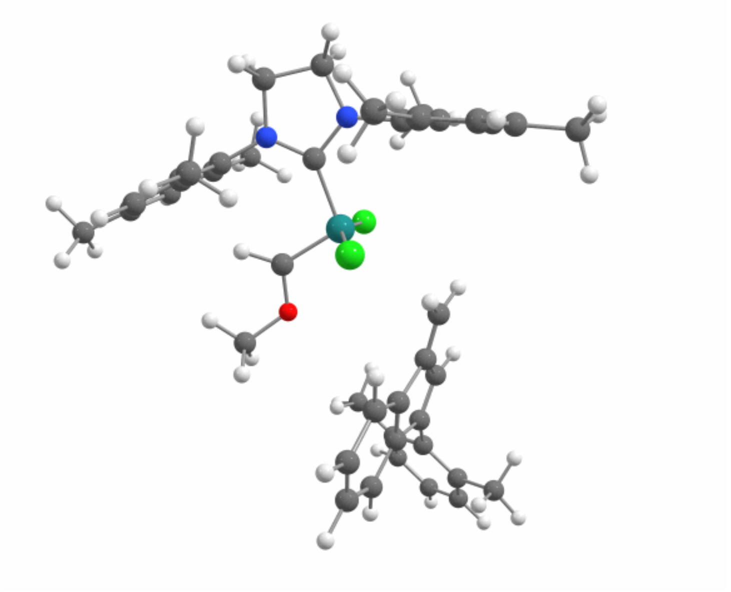

Zero-point correction= 0.741291 (Hartree/Particle)
Thermal correction to Energy= 0.793330
Thermal correction to Enthalpy= 0.794275
Thermal correction to Gibbs Free Energy= 0.647370
Sum of electronic and zero-point Energies= -2788.071307
Sum of electronic and thermal Energies= -2788.019267
Sum of electronic and thermal Enthalpies= -2788.018323
Sum of electronic and thermal Free Energies= -2788.165227

Solvent: -2789.33201149

Ru	-1.195299000	0.191153000	0.007808000
Cl	-0.530903000	0.197979000	-2.251951000
Cl	-1.189323000	0.923078000	2.259807000
N	-3.941744000	-1.005945000	-0.473857000
N	-3.848280000	1.205620000	-0.524763000
C	-3.093431000	0.066606000	-0.311637000
C	-5.342140000	-0.596339000	-0.700105000
H	-5.785741000	-1.167923000	-1.541225000
C	-5.211637000	0.905750000	-0.996970000
H	-5.309142000	1.128913000	-2.083927000
C	-3.658116000	-2.399705000	-0.293030000
C	-3.305190000	-3.181373000	-1.423177000
C	-3.145861000	-2.547205000	-2.783941000
H	-2.888086000	-3.305643000	-3.548700000
H	-2.347571000	-1.772803000	-2.772904000
H	-4.077471000	-2.038808000	-3.113026000
C	-3.073913000	-4.558611000	-1.231298000
H	-2.791476000	-5.172738000	-2.103047000
C	-3.192862000	-5.169505000	0.034115000
C	-2.984805000	-6.658771000	0.200142000
H	-2.644634000	-6.915063000	1.223903000
H	-2.240388000	-7.052235000	-0.521542000
H	-3.932711000	-7.213843000	0.023593000
C	-3.542383000	-4.358945000	1.134423000
H	-3.630866000	-4.814630000	2.135191000
C	-3.782063000	-2.976288000	0.998287000
C	-4.098720000	-2.124040000	2.205932000
H	-4.176398000	-2.744250000	3.120073000
H	-5.058020000	-1.576227000	2.090610000
H	-3.312866000	-1.355676000	2.374197000
C	-3.403485000	2.570192000	-0.390612000
C	-3.586414000	3.222338000	0.861149000
C	-4.234925000	2.516296000	2.027399000
H	-4.443544000	3.227530000	2.850490000
H	-3.566823000	1.721079000	2.420184000
H	-5.194461000	2.037117000	1.738720000
C	-3.167112000	4.560085000	0.983963000
H	-3.294503000	5.065060000	1.956236000
C	-2.603227000	5.270474000	-0.094811000
C	-2.111153000	6.689197000	0.082340000
H	-2.103280000	7.244178000	-0.877388000
H	-1.071125000	6.699707000	0.476950000
H	-2.736369000	7.252920000	0.804776000
C	-2.513046000	4.619842000	-1.339994000
H	-2.125061000	5.173178000	-2.211725000
C	-2.924370000	3.283377000	-1.524143000
C	-2.905939000	2.673633000	-2.905811000
H	-3.938196000	2.491084000	-3.278051000
H	-2.358757000	1.708366000	-2.926533000
H	-2.416024000	3.356253000	-3.627515000
H	3.904108000	1.613890000	-1.626597000
C	3.939072000	1.169269000	-0.621988000
C	4.973140000	0.419436000	-0.109623000
C	2.868033000	1.316497000	0.376505000
C	4.611314000	0.022283000	1.273279000
C	3.323957000	0.561732000	1.572424000
C	5.300880000	-0.748843000	2.222582000
C	2.721832000	0.323777000	2.814069000
C	4.693559000	-0.977527000	3.475785000
H	6.294347000	-1.166323000	1.991463000
C	3.419700000	-0.450800000	3.767227000
H	1.716792000	0.716210000	3.038546000
H	5.221811000	-1.576966000	4.235031000
H	2.961314000	-0.643951000	4.750448000
C	6.245466000	0.045066000	-0.789381000
C	6.271127000	-1.057548000	-1.689450000
C	7.431893000	0.785920000	-0.522897000
C	7.488657000	-1.398026000	-2.315243000
C	8.628505000	0.413521000	-1.169926000
C	8.660223000	-0.669576000	-2.060893000
H	7.512610000	-2.253041000	-3.011049000
H	9.547549000	0.988559000	-0.968625000
H	9.603376000	-0.948922000	-2.557615000
C	1.715115000	2.026137000	0.230606000
C	-0.752755000	-1.550032000	0.361124000
H	0.979825000	2.106233000	1.048038000
H	1.491145000	2.551274000	-0.710935000
H	-1.428603000	-2.423368000	0.508331000
O	0.542360000	-1.841114000	0.500340000
C	0.948674000	-3.150950000	0.937538000
H	1.738094000	-3.508122000	0.248531000
H	1.363280000	-3.068499000	1.962067000
H	0.087232000	-3.852231000	0.927497000
C	7.407150000	1.966437000	0.423442000
H	7.057200000	1.672585000	1.435617000
C	5.017065000	-1.856993000	-1.971483000
H	4.259429000	-1.255127000	-2.516278000
H	8.412471000	2.421559000	0.525539000
H	6.706355000	2.752435000	0.070655000
H	5.242920000	-2.753995000	-2.582301000
H	4.528110000	-2.189028000	-1.031868000
H	-5.960111000	1.521938000	-0.457273000
H	-5.958311000	-0.798876000	0.206444000

Catalyst 4, I intermediate

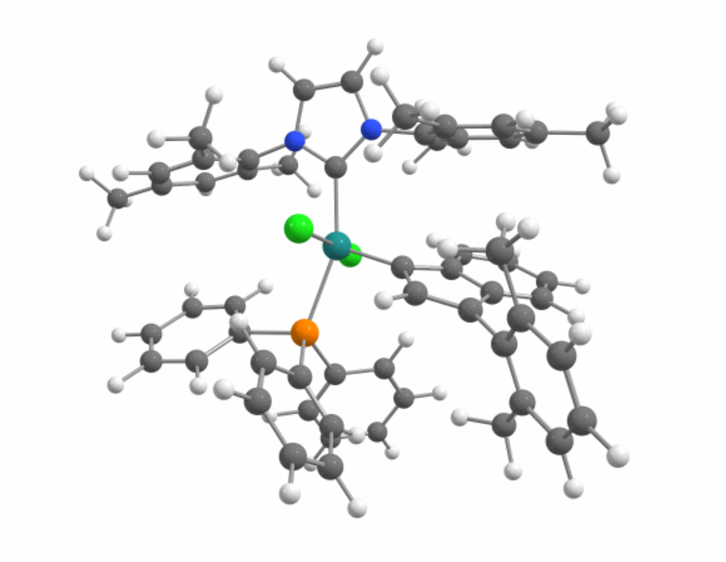

Zero-point correction= 0.904247 (Hartree/Particle)
Thermal correction to Energy= 0.966904
Thermal correction to Enthalpy= 0.967848
Thermal correction to Gibbs Free Energy= 0.802248
Sum of electronic and zero-point Energies= -3629.462940
Sum of electronic and thermal Energies= -3629.400282
Sum of electronic and thermal Enthalpies= -3629.399338
Sum of electronic and thermal Free Energies= -3629.564938

Solvent: -3631.04789518

Ru	-0,707728000	-0,365627000	0,003693000
Cl	-1,407711000	-0,552042000	2,312544000
Cl	-0,563014000	-0,189967000	-2,412573000
P	-0,963993000	2,044775000	0,181291000
N	-2,533152000	-2,806946000	-0,614362000
N	-0,481602000	-3,510631000	-0,477044000
C	-1,246323000	-2,368976000	-0,339389000
C	-2,552146000	-4,170674000	-0,917021000
H	-3,485058000	-4,687802000	-1,161489000
C	-1,265760000	-4,613132000	-0,828725000
H	-0,824676000	-5,603886000	-0,974479000
C	-3,784173000	-2,070135000	-0,590151000
C	-4,545470000	-2,067988000	0,607494000
C	-4,061141000	-2,748667000	1,863715000
H	-4,857758000	-2,763386000	2,632916000
H	-3,175777000	-2,221259000	2,282550000
H	-3,754201000	-3,797794000	1,666502000
C	-5,811280000	-1,445893000	0,577990000
H	-6,411224000	-1,436342000	1,503455000
C	-6,341105000	-0,874998000	-0,594046000
C	-7,698724000	-0,209879000	-0,602941000
H	-7,600582000	0,886569000	-0,757111000
H	-8,242580000	-0,369381000	0,349581000
H	-8,333818000	-0,594052000	-1,428849000
C	-5,578770000	-0,959167000	-1,775917000
H	-5,993045000	-0,559897000	-2,716789000
C	-4,310794000	-1,571022000	-1,812142000
C	-3,588924000	-1,747060000	-3,124765000
H	-4,176706000	-1,310188000	-3,955480000
H	-3,427294000	-2,822837000	-3,353578000
H	-2,586222000	-1,267486000	-3,107000000
C	0,928445000	-3,705811000	-0,218248000
C	1,848821000	-3,613547000	-1,289203000
C	1,405421000	-3,231011000	-2,679512000
H	2,268863000	-3,190254000	-3,371590000
H	0,891531000	-2,244717000	-2,691395000
H	0,680405000	-3,968210000	-3,088391000
C	3,194744000	-3,936575000	-1,016784000
H	3,926079000	-3,877316000	-1,839901000
C	3,626029000	-4,352189000	0,258219000
C	5,081871000	-4,656024000	0,530936000
H	5,199512000	-5,557478000	1,167469000
H	5,563460000	-3,812344000	1,071997000
H	5,651158000	-4,817921000	-0,406237000
C	2,664855000	-4,464720000	1,282731000
H	2,978483000	-4,803820000	2,283763000
C	1,307898000	-4,159372000	1,068737000
C	0,284361000	-4,348468000	2,164837000
H	-0,384053000	-5,210429000	1,947519000
H	-0,368543000	-3,458301000	2,280262000
H	0,777806000	-4,546858000	3,136044000
H	1,721834000	0,897143000	-1,634294000
C	2,063166000	0,449737000	-0,694123000
C	3,355817000	0,472316000	-0,217131000
C	1,154994000	-0,240454000	0,247820000
C	3,372340000	-0,243049000	1,083433000
C	2,038958000	-0,666666000	1,387886000
C	4,433917000	-0,513698000	1,953494000
C	1,783998000	-1,310400000	2,604076000
C	4,164492000	-1,178894000	3,173703000
H	5,459745000	-0,204230000	1,694721000
C	2,852869000	-1,555440000	3,500032000
H	0,754405000	-1,591806000	2,863870000
H	4,988774000	-1,388815000	3,874513000
H	2,648462000	-2,052974000	4,461567000
C	4,540373000	1,115102000	-0,846705000
C	5,075070000	0,601212000	-2,064381000
C	5,135358000	2,256327000	-0,228835000
C	6,211979000	1,218608000	-2,625703000
C	6,263974000	2,848720000	-0,831786000
C	6,807379000	2,332688000	-2,017251000
H	6,633102000	0,811996000	-3,560040000
H	6,715645000	3,737200000	-0,360077000
H	7,693526000	2,804428000	-2,471556000
C	-2,761572000	2,496222000	0,011118000
C	-3,221071000	3,532283000	-0,832077000
C	-3,686099000	1,821275000	0,841819000
C	-4,581245000	3,889826000	-0,839869000
H	-2,514989000	4,076958000	-1,476046000
C	-5,040613000	2,192470000	0,837396000
H	-3,334650000	1,015948000	1,508678000
C	-5,493093000	3,226502000	-0,001286000
H	-4,924706000	4,702842000	-1,499576000
H	-5,745578000	1,665428000	1,498899000
H	-6,555453000	3,519486000	0,001787000
C	-0,096884000	3,145991000	-1,037103000
C	0,894127000	4,060713000	-0,613481000
C	-0,393368000	3,051114000	-2,418665000
C	1,572821000	4,861738000	-1,548191000
H	1,134799000	4,154362000	0,455759000
C	0,283253000	3,861058000	-3,346650000
H	-1,140958000	2,326589000	-2,770066000
C	1,269409000	4,765864000	-2,916737000
H	2,343773000	5,567048000	-1,199535000
H	0,039250000	3,775155000	-4,417613000
H	1,802187000	5,394741000	-3,647800000
C	-0,544474000	2,849387000	1,806039000

C	-1,213918000	4,022206000	2,223851000
C	0,478157000	2,315860000	2,616979000
C	-0,858004000	4,652031000	3,428209000
H	-2,021303000	4,447696000	1,607968000
C	0,834539000	2,951470000	3,819699000
H	0,988484000	1,390155000	2,316158000
C	0,167744000	4,118607000	4,228934000
H	-1,389864000	5,564081000	3,743512000
H	1,631377000	2,518291000	4,444566000
H	0,442284000	4,610281000	5,176067000
C	4,551522000	2,864268000	1,028541000
C	4,447349000	-0,586983000	-2,759421000
H	4,188574000	-1,392674000	-2,043143000
H	3,500004000	-0,307265000	-3,267544000
H	5,129208000	-1,002362000	-3,528666000
H	3,452348000	2,991386000	0,943910000
H	4,722064000	2,225128000	1,920517000
H	5,000813000	3,856085000	1,235504000

Catalyst 4, I-II TS

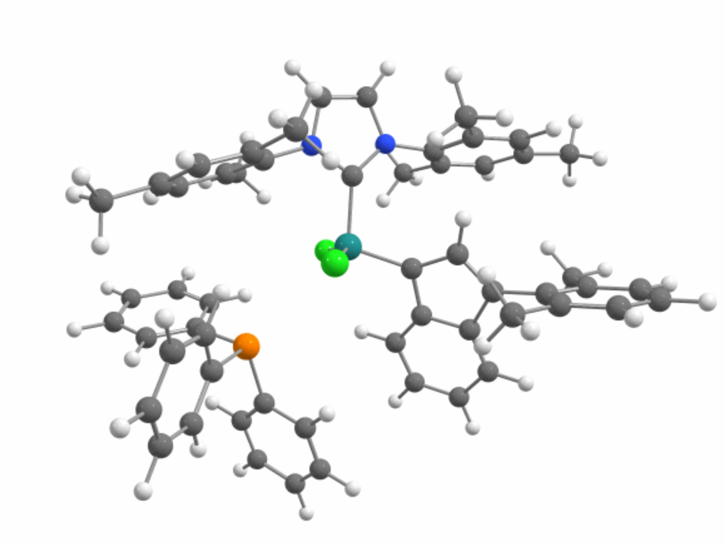

Zero-point correction= 0.902241 (Hartree/Particle)
Thermal correction to Energy= 0.965193
Thermal correction to Enthalpy= 0.966138
Thermal correction to Gibbs Free Energy= 0.796350
Sum of electronic and zero-point Energies= -3629.441071
Sum of electronic and thermal Energies= -3629.378119
Sum of electronic and thermal Enthalpies= -3629.377175
Sum of electronic and thermal Free Energies= -3629.546962

Solvent: -3631.01149402

Ru	-0.179271	-0.580017	-0.234075
Cl	0.437653	-0.647023	-2.497713
Cl	0.305889	-0.271427	2.031300
P	3.271803	1.268463	-0.190083
N	0.015685	-3.489050	0.095378
N	-2.002854	-3.059555	-0.637398
C	-0.833945	-2.423567	-0.216947
C	-0.602734	-4.717829	-0.134907
H	-0.081551	-5.662433	0.048922
C	-1.860385	-4.451925	-0.586295
H	-2.680373	-5.114481	-0.879514
C	1.352695	-3.419538	0.649721
C	2.469544	-3.438466	-0.224345
C	2.311739	-3.564202	-1.720516
H	3.301614	-3.624786	-2.212177
H	1.763337	-2.702539	-2.156327
H	1.739361	-4.478358	-1.988993
C	3.754317	-3.401992	0.356528
H	4.630702	-3.394074	-0.312075
C	3.948120	-3.399265	1.750993
C	5.336676	-3.324395	2.343740
H	5.399509	-3.864063	3.310719
H	5.621321	-2.266865	2.538384
H	6.098690	-3.746989	1.658426
C	2.809599	-3.465689	2.580400
H	2.939234	-3.512264	3.674677
C	1.502296	-3.486261	2.059173
C	0.309230	-3.594634	2.978089
H	0.632326	-3.778797	4.021176
H	-0.371214	-4.419360	2.678326
H	-0.276160	-2.650960	2.962154
C	-3.301713	-2.520530	-0.981654
C	-4.296564	-2.502142	0.028032
C	-3.991173	-2.928393	1.447438
H	-4.769761	-2.563819	2.145762
H	-3.006531	-2.555728	1.797182
H	-3.958361	-4.036470	1.537784
C	-5.600092	-2.107315	-0.331150
H	-6.377696	-2.076055	0.450051
C	-5.935302	-1.762603	-1.655131
C	-7.355828	-1.402841	-2.031015
H	-7.910266	-2.299968	-2.385606
H	-7.386427	-0.657979	-2.851837
H	-7.916787	-0.990969	-1.168352
C	-4.920560	-1.813902	-2.631717
H	-5.164973	-1.562173	-3.677372
C	-3.600165	-2.204491	-2.330319
C	-2.569260	-2.329958	-3.423311
H	-2.162917	-3.363165	-3.475853
H	-1.699303	-1.662168	-3.251116
H	-3.009739	-2.086773	-4.409662
H	-3.238690	-0.430983	1.128521
C	-2.927519	0.432813	0.534877
C	-3.558642	1.663183	0.545699
C	-1.703133	0.491714	-0.260078
C	-2.788167	2.583840	-0.324073
C	-1.652181	1.874717	-0.829520
C	-3.008303	3.921306	-0.667674
C	-0.763187	2.507071	-1.707958
C	-2.099852	4.556091	-1.550103
H	-3.872940	4.470209	-0.260192
C	-0.998665	3.857137	-2.067427
H	0.099927	1.961690	-2.118374
H	-2.263829	5.608068	-1.834080
H	-0.300774	4.360950	-2.753749
C	-4.765148	2.049385	1.325685
C	-6.004348	2.254545	0.651627
C	-4.671846	2.228945	2.736675
C	-7.137142	2.623981	1.406206
C	-5.829493	2.604245	3.450161
C	-7.054647	2.798710	2.795501
H	-8.098526	2.775062	0.887761
H	-5.759505	2.751463	4.540581

H	-7.948227	3.091262	3.370097
C	4.089736	1.493852	1.466571
C	4.900624	2.603398	1.798663
C	3.861047	0.498049	2.445191
C	5.481181	2.707584	3.075152
H	5.077271	3.394587	1.053003
C	4.448971	0.602476	3.718250
H	3.199441	-0.352910	2.216148
C	5.259856	1.706235	4.037228
H	6.110132	3.579259	3.318890
H	4.254685	-0.177178	4.472414
H	5.711091	1.791600	5.038956
C	3.326017	3.002773	-0.861797
C	4.225261	3.444633	-1.857755
C	2.370101	3.916187	-0.355088
C	4.177386	4.770783	-2.325719
H	4.972237	2.747914	-2.269081
C	2.335366	5.243715	-0.811742
H	1.644438	3.582243	0.405229
C	3.238312	5.675444	-1.801139
H	4.886339	5.099077	-3.103151
H	1.588943	5.941496	-0.399717
H	3.206270	6.714834	-2.165481
C	4.606236	0.426098	-1.177663
C	5.941496	0.280176	-0.738652
C	4.234065	-0.126676	-2.426885
C	6.885460	-0.392880	-1.535392
H	6.245973	0.697896	0.233694
C	5.183368	-0.785937	-3.226831
H	3.187324	-0.049428	-2.765639
C	6.511459	-0.923530	-2.782654
H	7.923211	-0.498206	-1.179038
H	4.879278	-1.203652	-4.200273
H	7.253527	-1.446850	-3.407040
C	-3.357765	2.048787	3.466309
H	-2.541266	2.629521	2.989197
C	-6.116964	2.055730	-0.844213
H	-5.734292	1.059013	-1.147913
H	-3.442857	2.375039	4.521967
H	-3.018741	0.991972	3.464666
H	-7.168953	2.144884	-1.181137
H	-5.517278	2.802243	-1.406668

Catalyst 4, II intermediate

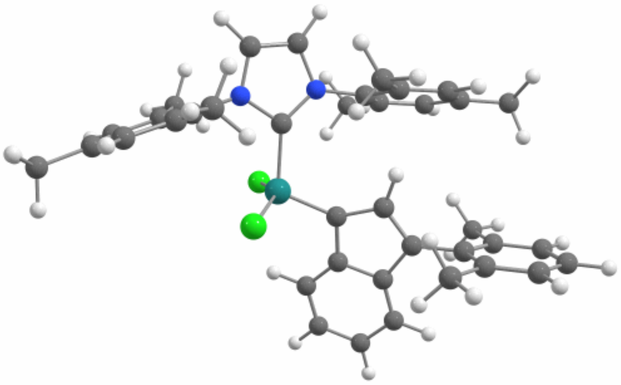

Zero-point correction= 0.636072 (Hartree/Particle)
Thermal correction to Energy= 0.681266
Thermal correction to Enthalpy= 0.682210
Thermal correction to Gibbs Free Energy= 0.554745
Sum of electronic and zero-point Energies= -2593.985055
Sum of electronic and thermal Energies= -2593.939861
Sum of electronic and thermal Enthalpies= -2593.938916
Sum of electronic and thermal Free Energies= -2594.066381

Solvent: -2595.05490694

Ru	-1.181232	-0.777452	-0.323334
Cl	-1.666779	-0.883047	-2.599658
Cl	-1.666298	-1.661434	1.762948
N	-2.996664	1.497918	0.018306
N	-0.999998	2.318622	-0.348795
C	-1.664102	1.109447	-0.160115
C	-3.136084	2.883017	-0.071926
H	-4.111369	3.366604	0.040946
C	-1.893958	3.395885	-0.297679
H	-1.546239	4.425391	-0.426570
C	-4.122521	0.646955	0.346878
C	-4.988829	0.203261	-0.684748
C	-4.802706	0.622095	-2.122945
H	-5.612828	0.212453	-2.756942
H	-3.831569	0.268362	-2.528765
H	-4.815665	1.728276	-2.228606
C	-6.080586	-0.608893	-0.313578
H	-6.755121	-0.973399	-1.106226
C	-6.345681	-0.946482	1.027273
C	-7.495438	-1.859220	1.388823
H	-7.954014	-1.576761	2.358484
H	-7.146018	-2.910514	1.489102
H	-8.287619	-1.849407	0.613364
C	-5.508361	-0.409067	2.025891
H	-5.726232	-0.620114	3.086067
C	-4.400640	0.401095	1.716915
C	-3.560806	1.011135	2.813512
H	-3.999999	0.799473	3.807637
H	-3.478231	2.113214	2.701367
H	-2.533496	0.591860	2.799351
C	0.414899	2.590980	-0.449315
C	1.093352	2.992014	0.730996
C	0.391896	3.006281	2.070603
H	1.095093	3.276279	2.882098
H	-0.045940	2.013412	2.308700
H	-0.446724	3.734735	2.092187
C	2.449265	3.353552	0.622375
H	2.990903	3.658154	1.533364
C	3.131268	3.327096	-0.612202
C	4.598500	3.683318	-0.687748
H	4.913382	3.908179	-1.726245
H	5.225845	2.841004	-0.321682
H	4.837552	4.562928	-0.054972
C	2.411768	2.943167	-1.760600
H	2.920744	2.940643	-2.739010
C	1.048362	2.581086	-1.713531
C	0.297927	2.225229	-2.972908
H	-0.606065	2.858741	-3.097479
H	-0.059884	1.173767	-2.956432
H	0.937903	2.362193	-3.865989
H	1.763376	0.716217	0.701799

C	1.802603	-0.286768	0.268690
C	2.927260	-1.093773	0.247342
C	0.669080	-0.983782	-0.322522
C	2.569881	-2.374845	-0.402045
C	1.189303	-2.315265	-0.772194
C	3.335420	-3.515546	-0.669605
C	0.593091	-3.396259	-1.436083
C	2.720317	-4.609419	-1.323346
H	4.396866	-3.558885	-0.375897
C	1.369999	-4.548765	-1.704286
H	-0.457786	-3.351718	-1.762233
H	3.309353	-5.514892	-1.540126
H	0.907364	-5.404128	-2.221187
C	4.276020	-0.765542	0.784215
C	5.312386	-0.378740	-0.113887
C	4.526476	-0.852924	2.183235
C	6.586802	-0.073310	0.408640
C	5.816217	-0.541476	2.662806
C	6.840153	-0.152472	1.786540
H	7.390789	0.228000	-0.283280
H	6.015729	-0.612615	3.744802
H	7.842125	0.085302	2.178578
C	5.047800	-0.278641	-1.600348
H	4.213643	0.422842	-1.813967
C	3.438176	-1.282604	3.143362
H	2.966197	-2.235940	2.826304
H	3.839980	-1.418949	4.167097
H	2.617381	-0.536830	3.196310
H	5.946535	0.070729	-2.146627
H	4.746130	-1.257255	-2.030051

Catalyst 4, II-III TS

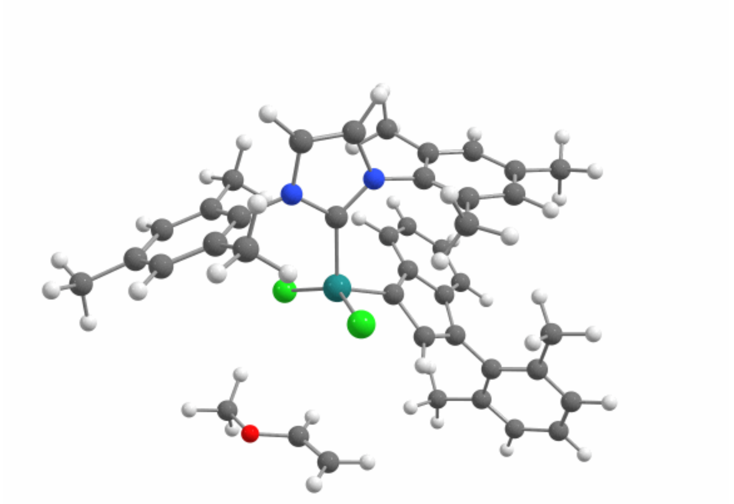

Zero-point correction= 0.717494 (Hartree/Particle)
Thermal correction to Energy= 0.769705
Thermal correction to Enthalpy= 0.770649
Thermal correction to Gibbs Free Energy= 0.622228
Sum of electronic and zero-point Energies= -2786.865391
Sum of electronic and thermal Energies= -2786.813181
Sum of electronic and thermal Enthalpies= -2786.812236
Sum of electronic and thermal Free Energies= -2786.960657

Solvent: -2788.10430935

Ru	-0,978323000	0,383197000	-0,260194000
Cl	-1,763700000	1,247196000	1,751246000
Cl	-0,925503000	0,642438000	-2,582983000
N	-2,974214000	-1,749642000	-0,459984000
N	-1,004900000	-2,708797000	-0,529289000
C	-1,612759000	-1,468106000	-0,345043000
C	-3,191311000	-3,104142000	-0,714429000
H	-4,201141000	-3,508738000	-0,833934000
C	-1,966623000	-3,701672000	-0,754887000
H	-1,674473000	-4,743062000	-0,919869000
C	-4,063632000	-0,815993000	-0,264717000
C	-4,559542000	-0,631985000	1,051592000
C	-3,984407000	-1,395431000	2,220510000
H	-4,552869000	-1,184225000	3,146833000
H	-2,928208000	-1,105841000	2,397424000
H	-4,009407000	-2,492129000	2,044088000
C	-5,626833000	0,266504000	1,231459000
H	-6,011563000	0,429868000	2,251879000
C	-6,217512000	0,952028000	0,150641000
C	-7,326929000	1,953454000	0,379013000
H	-8,005496000	2,020921000	-0,495276000
H	-6,909487000	2,970699000	0,547265000
H	-7,933714000	1,698683000	1,271647000
C	-5,742267000	0,682629000	-1,147248000
H	-6,221798000	1,172603000	-2,010952000
C	-4,677414000	-0,209464000	-1,390425000
C	-4,256153000	-0,532859000	-2,803482000
H	-4,893391000	0,003434000	-3,533021000
H	-4,340225000	-1,620673000	-3,014012000
H	-3,199146000	-0,246439000	-2,990446000
C	0,384681000	-3,088352000	-0,389279000
C	1,266129000	-2,967376000	-1,492568000
C	0,802447000	-2,445501000	-2,828508000
H	1,613458000	-2,513130000	-3,579304000
H	0,461391000	-1,390732000	-2,773037000
H	-0,063662000	-3,029354000	-3,208094000
C	2,591860000	-3,414095000	-1,320146000
H	3,286872000	-3,327190000	-2,171753000
C	3,045199000	-3,989701000	-0,116429000
C	4,487100000	-4,411896000	0,051801000
H	4,964752000	-4,640347000	-0,922308000
H	4,580285000	-5,305626000	0,702002000
H	5,080572000	-3,600243000	0,527410000
C	2,116834000	-4,153293000	0,929087000
H	2,438804000	-4,632370000	1,868589000
C	0,780281000	-3,724678000	0,814439000
C	-0,200477000	-4,009823000	1,930581000
H	-0,725309000	-4,976040000	1,760753000
H	-0,985030000	-3,233298000	2,018247000
H	0,321526000	-4,088778000	2,904387000
H	1,601260000	1,668467000	-1,450838000
C	1,819313000	1,104488000	-0,534878000
C	3,055068000	1,010051000	0,081328000
C	0,832719000	0,276840000	0,176395000
C	2,903127000	0,151784000	1,281444000

C	1,543761000	-0,291955000	1,346659000
C	3,810645000	-0,172010000	2,295997000
C	1,085146000	-0,974542000	2,476202000
C	3,348244000	-0,910977000	3,412289000
H	4,857498000	0,168713000	2,241869000
C	1,999495000	-1,289599000	3,510174000
H	0,022889000	-1,242652000	2,562819000
H	4,048808000	-1,166182000	4,223546000
H	1,647199000	-1,831383000	4,402366000
C	4,328447000	1,659673000	-0,334910000
C	5,107171000	1,101413000	-1,387603000
C	4,762646000	2,838737000	0,338159000
C	6,319954000	1,727636000	-1,742839000
C	5,979848000	3,434622000	-0,051797000
C	6,757064000	2,884773000	-1,081882000
H	6,928527000	1,293617000	-2,553363000
H	6,316508000	4,348782000	0,464376000
H	7,707150000	3,361379000	-1,371963000
C	-0,675930000	4,850241000	-0,045325000
C	-0,070556000	4,947341000	-1,246577000
H	1,003076000	4,731890000	-1,332184000
H	-0,633124000	5,225413000	-2,150669000
H	-0,117041000	4,562712000	0,867185000
O	-2,004572000	5,085369000	0,132570000
C	-2,460651000	5,056601000	1,481370000
H	-3,554340000	5,225397000	1,453761000
H	-2,261015000	4,070435000	1,956853000
H	-1,991425000	5,864363000	2,089765000
C	3,921245000	3,464581000	1,429297000
H	3,727320000	2,756494000	2,261881000
C	4,646389000	-0,139676000	-2,119835000
H	4,331301000	-0,938495000	-1,416922000
H	4,414309000	4,362959000	1,850434000
H	2,925266000	3,769262000	1,043830000
H	5,449597000	-0,541093000	-2,769618000
H	3,765611000	0,071952000	-2,763003000

Catalyst 4, III intermediate

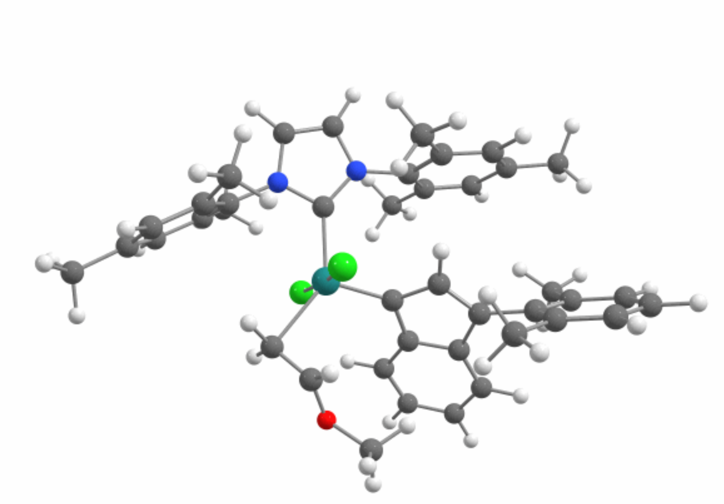

Zero-point correction= 0.720630 (Hartree/Particle)
Thermal correction to Energy= 0.771286
Thermal correction to Enthalpy= 0.772231
Thermal correction to Gibbs Free Energy= 0.634855
Sum of electronic and zero-point Energies= -2786.882383

Ru	1.209375000	0.591962000	0.032984000
Cl	1.929971000	0.926494000	-2.231502000
Cl	0.803811000	0.301791000	2.431825000
N	2.922301000	-1.837119000	-0.052228000
N	0.884031000	-2.543451000	-0.356829000
C	1.608743000	-1.389562000	-0.148437000
C	2.994949000	-3.223033000	-0.197766000
H	3.950036000	-3.755064000	-0.148806000
C	1.719959000	-3.667553000	-0.388575000
H	1.315949000	-4.671886000	-0.547437000
C	4.102091000	-1.027612000	0.184250000
C	4.909037000	-0.647367000	-0.922544000
C	4.611935000	-1.120751000	-2.323316000
H	5.363798000	-0.729724000	-3.035995000
H	3.608346000	-0.775351000	-2.650085000
H	4.626514000	-2.230173000	-2.387000000
C	6.041368000	0.148348000	-0.662330000
H	6.664757000	0.465718000	-1.514869000
C	6.409444000	0.530013000	0.643737000
C	7.602621000	1.427952000	0.881080000
H	7.989313000	1.332710000	1.915607000
H	7.330811000	2.495620000	0.727689000
H	8.431093000	1.202779000	0.178366000
C	5.645090000	0.035741000	1.717896000
H	5.958993000	0.256180000	2.751933000
C	4.502910000	-0.769270000	1.522496000
C	3.791549000	-1.379844000	2.703877000
H	4.363490000	-1.202672000	3.635515000
H	3.671060000	-2.476877000	2.578367000
H	2.772701000	-0.951750000	2.832428000
C	-0.542042000	-2.720741000	-0.500833000
C	-1.275252000	-3.165832000	0.627967000
C	-0.626317000	-3.277310000	1.988223000
H	-1.366561000	-3.577875000	2.755082000
H	-0.171151000	-2.310202000	2.298224000
H	0.191925000	-4.029393000	1.995565000
C	-2.631388000	-3.488721000	0.439533000
H	-3.218044000	-3.832585000	1.307770000
C	-3.253728000	-3.393303000	-0.822819000
C	-4.706804000	-3.778342000	-0.987104000
H	-5.041056000	-3.681799000	-2.038936000
H	-5.362432000	-3.137418000	-0.359698000
H	-4.882692000	-4.828108000	-0.668568000
C	-2.486780000	-2.936110000	-1.912500000
H	-2.955280000	-2.857911000	-2.907797000
C	-1.123902000	-2.594829000	-1.782906000
C	-0.312189000	-2.137905000	-2.971017000
H	0.532719000	-2.831205000	-3.173895000
H	0.139544000	-1.136358000	-2.801263000
H	-0.938218000	-2.090260000	-3.883004000
H	-1.583850000	-0.562822000	1.252189000
C	-1.731282000	0.217960000	0.501373000
C	-2.939579000	0.807905000	0.186239000
C	-0.634536000	0.803912000	-0.282202000
C	-2.681151000	1.843486000	-0.844232000

Sum of electronic and thermal Energies= -2786.831727
Sum of electronic and thermal Enthalpies= -2786.830783
Sum of electronic and thermal Free Energies= -2786.968159

Solvent: -2788.12932149

C	-1.285823000	1.821746000	-1.171389000
C	-3.538863000	2.789339000	-1.415890000
C	-0.784645000	2.720190000	-2.122050000
C	-3.020858000	3.693653000	-2.375496000
H	-4.599250000	2.834140000	-1.119004000
C	-1.665848000	3.646029000	-2.733837000
H	0.281653000	2.696597000	-2.386445000
H	-3.689131000	4.435527000	-2.841871000
H	-1.272691000	4.345154000	-3.489076000
C	-4.266609000	0.526052000	0.792189000
C	-5.342681000	0.060440000	-0.024494000
C	-4.468078000	0.731605000	2.191411000
C	-6.593377000	-0.197637000	0.575217000
C	-5.739038000	0.469232000	2.743549000
C	-6.796330000	0.005629000	1.947770000
H	-7.419953000	-0.568798000	-0.053440000
H	-5.897459000	0.639966000	3.821205000
H	-7.781570000	-0.196239000	2.398005000
C	2.301187000	2.511563000	0.443903000
C	1.157804000	3.150617000	0.906601000
H	2.918870000	1.992731000	1.201000000
H	2.804719000	2.883974000	-0.462522000
H	0.703652000	2.879445000	1.879151000
O	0.607967000	4.164830000	0.226035000
C	-0.633798000	4.677922000	0.730358000
H	-0.549088000	4.917448000	1.812604000
H	-0.846323000	5.601341000	0.161841000
H	-1.454036000	3.949085000	0.563994000
C	-3.363176000	1.248276000	3.089142000
H	-2.823707000	2.100929000	2.628949000
C	-5.163581000	-0.194657000	-1.506152000
H	-4.196383000	-0.693328000	-1.719713000
H	-5.981082000	-0.832504000	-1.897560000
H	-5.168301000	0.746765000	-2.094725000
H	-3.772726000	1.580058000	4.064193000
H	-2.589989000	0.478945000	3.294808000

Catalyst 4, III-IV TS

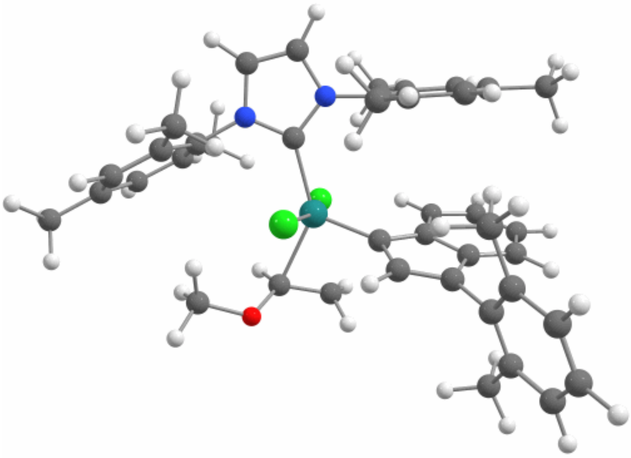

Zero-point correction= 0.720246 (Hartree/Particle)
Thermal correction to Energy= 0.770173
Thermal correction to Enthalpy= 0.771118
Thermal correction to Gibbs Free Energy= 0.634969
Sum of electronic and zero-point Energies= -2786.874954
Sum of electronic and thermal Energies= -2786.825027
Sum of electronic and thermal Enthalpies= -2786.824083

Ru	-0.871167000	-0.328321000	-0.626693000
Cl	-1.399432000	0.969695000	-2.616981000
Cl	-0.891370000	-1.649552000	1.420162000
N	-3.165807000	1.158548000	0.856767000
N	-1.315460000	2.298538000	0.986466000
C	-1.832297000	1.121152000	0.498512000
C	-3.463113000	2.339093000	1.545203000
H	-4.474116000	2.551681000	1.906055000
C	-2.301405000	3.051039000	1.631701000
H	-2.072116000	4.016319000	2.093237000
C	-4.175924000	0.141967000	0.631253000
C	-4.935622000	0.174875000	-0.565969000
C	-4.752303000	1.259089000	-1.599107000
H	-5.481414000	1.140004000	-2.424025000
H	-3.728435000	1.256478000	-2.032986000
H	-4.906740000	2.265251000	-1.152770000
C	-5.930683000	-0.811775000	-0.736160000
H	-6.522587000	-0.801867000	-1.666674000
C	-6.211637000	-1.774832000	0.251291000
C	-7.269355000	-2.834278000	0.034499000
H	-7.881517000	-2.991630000	0.946546000
H	-6.808575000	-3.815238000	-0.215491000
H	-7.951510000	-2.568892000	-0.797808000
C	-5.488057000	-1.715190000	1.460269000
H	-5.725306000	-2.426892000	2.268836000
C	-4.478797000	-0.759237000	1.686988000
C	-3.776409000	-0.685320000	3.019725000
H	-4.174555000	-1.447998000	3.716801000
H	-3.903236000	0.311070000	3.494675000
H	-2.686453000	-0.859614000	2.888706000
C	0.055378000	2.758779000	0.916349000
C	0.963500000	2.331266000	1.915594000
C	0.544084000	1.368133000	2.999154000
H	1.358825000	1.223518000	3.734776000
H	0.265040000	0.374411000	2.582270000
H	-0.348219000	1.739004000	3.548023000
C	2.268928000	2.861737000	1.876798000
H	2.988298000	2.546985000	2.651017000
C	2.672226000	3.792759000	0.899402000
C	4.091370000	4.311618000	0.854991000
H	4.130362000	5.373299000	0.535730000
H	4.695323000	3.728874000	0.125188000
H	4.592044000	4.226807000	1.840539000
C	1.722038000	4.213722000	-0.052104000
H	2.012056000	4.956553000	-0.813726000
C	0.403613000	3.718246000	-0.065904000

Sum of electronic and thermal Free Energies= -2786.960231

Solvent: -2788.12068301

C	-0.596254000	4.204413000	-1.088032000
H	-1.470618000	4.696773000	-0.610283000
H	-0.992387000	3.359463000	-1.693143000
H	-0.130363000	4.938758000	-1.773614000
H	1.442070000	-2.220939000	0.714672000
C	1.872737000	-1.439460000	0.079055000
C	3.209803000	-1.197888000	-0.117416000
C	1.037123000	-0.501037000	-0.717269000
C	3.338451000	-0.064016000	-1.068559000
C	2.029163000	0.351742000	-1.464944000
C	4.481275000	0.570040000	-1.566339000
C	1.884836000	1.395405000	-2.385710000
C	4.323720000	1.621360000	-2.500790000
H	5.483846000	0.249873000	-1.239048000
C	3.042428000	2.020393000	-2.909795000
H	0.879916000	1.711004000	-2.701885000
H	5.214205000	2.124006000	-2.911891000
H	2.930081000	2.833294000	-3.644678000
C	4.359919000	-1.931281000	0.479265000
C	4.684833000	-1.755854000	1.854977000
C	5.134839000	-2.807031000	-0.336859000
C	5.795750000	-2.443955000	2.385814000
C	6.234341000	-3.478721000	0.236043000
C	6.569027000	-3.297675000	1.586019000
H	6.054775000	-2.300953000	3.447902000
H	6.830797000	-4.159959000	-0.393030000
H	7.433828000	-3.827039000	2.017221000
C	0.000485000	-1.811227000	-2.173172000
C	-1.325623000	-2.143174000	-1.755610000
H	0.125733000	-1.206812000	-3.084114000
H	0.777929000	-2.558588000	-1.952840000
H	-2.184504000	-1.724730000	-2.326393000
O	-1.536397000	-3.351985000	-1.177076000
C	-2.839211000	-3.572125000	-0.633083000
H	-3.630563000	-3.258455000	-1.351579000
H	-2.924393000	-4.658541000	-0.442196000
H	-2.951909000	-3.011795000	0.318642000
C	4.762586000	-3.059235000	-1.781971000
H	4.755750000	-2.125087000	-2.381273000
C	3.861015000	-0.849176000	2.742891000
H	2.859005000	-1.281616000	2.948478000
H	4.363430000	-0.682737000	3.716798000
H	3.680748000	0.136836000	2.267233000
H	5.468438000	-3.767539000	-2.259309000
H	3.741395000	-3.489442000	-1.862208000

Catalyst 4, IV intermediate

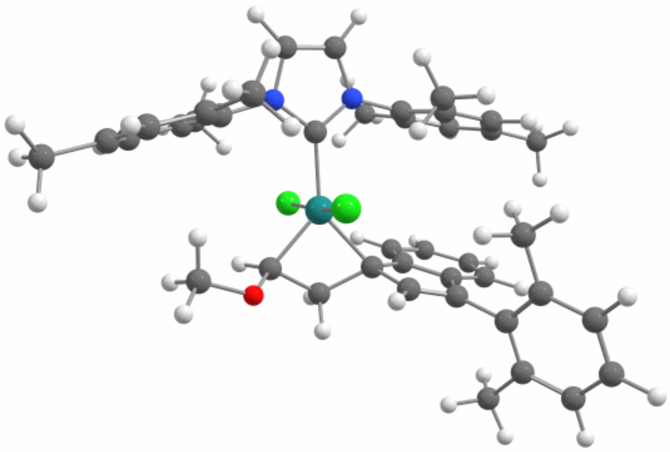

Ru	-0.869270000	-0.295449000	-0.617150000
Cl	-1.493433000	0.963851000	-2.597441000
Cl	-0.602756000	-1.511845000	1.475418000
N	-3.150414000	1.142529000	0.895710000
N	-1.275943000	2.249004000	1.044952000
C	-1.822004000	1.096211000	0.533574000
C	-3.420974000	2.313257000	1.612936000
H	-4.426723000	2.533680000	1.983374000
C	-2.246510000	3.002777000	1.710186000
H	-1.998503000	3.955107000	2.188669000
C	-4.185941000	0.154234000	0.655532000
C	-4.994945000	0.271207000	-0.502558000
C	-4.811029000	1.391506000	-1.495810000
H	-5.581563000	1.340707000	-2.289338000
H	-3.807959000	1.356865000	-1.975366000
H	-4.895403000	2.384062000	-1.002976000
C	-6.028391000	-0.674802000	-0.675444000
H	-6.659872000	-0.599989000	-1.576435000
C	-6.295455000	-1.678963000	0.273774000
C	-7.405045000	-2.685014000	0.062326000
H	-8.046732000	-2.776197000	0.963649000
H	-6.995740000	-3.698437000	-0.141752000
H	-8.052606000	-2.408786000	-0.793487000
C	-5.509443000	-1.711575000	1.444482000
H	-5.728118000	-2.461468000	2.223214000
C	-4.459456000	-0.800107000	1.671731000
C	-3.681224000	-0.831732000	2.962730000
H	-4.087479000	-1.600716000	3.647996000
H	-3.718861000	0.147342000	3.486467000
H	-2.609862000	-1.059738000	2.765735000
C	0.110165000	2.672013000	0.979190000
C	0.977710000	2.299321000	2.036631000
C	0.500192000	1.454245000	3.191821000
H	1.291908000	1.354902000	3.959274000
H	0.208117000	0.435070000	2.851622000
H	-0.393673000	1.898212000	3.680440000
C	2.304521000	2.771587000	1.984129000
H	2.993419000	2.496101000	2.799716000
C	2.767723000	3.595216000	0.939390000
C	4.209937000	4.043248000	0.882856000
H	4.306795000	5.063394000	0.458279000
H	4.800520000	3.361312000	0.232404000
H	4.683133000	4.036538000	1.885461000
C	1.852866000	3.982287000	-0.059285000
H	2.186376000	4.654832000	-0.866579000
C	0.512190000	3.550378000	-0.058396000
C	-0.454158000	4.037810000	-1.109864000
H	-1.341580000	4.527945000	-0.654724000

Zero-point correction= 0.722052 (Hartree/Particle)
Thermal correction to Energy= 0.771975
Thermal correction to Enthalpy= 0.772919
Thermal correction to Gibbs Free Energy= 0.637074
Sum of electronic and zero-point Energies= -2786.884560
Sum of electronic and thermal Energies= -2786.834638
Sum of electronic and thermal Enthalpies= -2786.833694
Sum of electronic and thermal Free Energies= -2786.969539

Solvent: -2788.13078493

H	-0.834217000	3.198363000	-1.732638000
H	0.034229000	4.774352000	-1.776936000
H	1.497327000	-2.553576000	0.307968000
C	1.892323000	-1.697388000	-0.250493000
C	3.195477000	-1.276236000	-0.262851000
C	1.046597000	-0.881175000	-1.173253000
C	3.295319000	-0.128495000	-1.195169000
C	2.013690000	0.103233000	-1.782230000
C	4.403130000	0.652431000	-1.555784000
C	1.862782000	1.099164000	-2.755885000
C	4.240393000	1.654611000	-2.536998000
H	5.385403000	0.470765000	-1.089978000
C	2.986877000	1.866275000	-3.136570000
H	0.874806000	1.290694000	-3.200965000
H	5.104339000	2.268512000	-2.839530000
H	2.873041000	2.643728000	-3.908819000
C	4.338653000	-1.884268000	0.473841000
C	4.433829000	-1.741736000	1.888295000
C	5.328762000	-2.619723000	-0.242063000
C	5.533752000	-2.316432000	2.558849000
C	6.410888000	-3.179528000	0.468307000
C	6.519550000	-3.026638000	1.858313000
H	5.612683000	-2.199786000	3.652446000
H	7.173738000	-3.752755000	-0.084199000
H	7.372511000	-3.468205000	2.398475000
C	0.077317000	-1.718254000	-2.105134000
C	-1.432368000	-1.901024000	-1.647806000
H	0.086363000	-1.297896000	-3.127504000
H	0.461534000	-2.755781000	-2.066279000
H	-2.155690000	-1.640693000	-2.455802000
O	-1.652203000	-3.140496000	-1.107736000
C	-3.019618000	-3.489304000	-0.913623000
H	-3.615443000	-3.308322000	-1.838233000
H	-3.043463000	-4.567726000	-0.667166000
H	-3.462991000	-2.909717000	-0.076315000
C	3.382290000	-0.983278000	2.666843000
H	2.397272000	-1.495055000	2.642373000
C	5.205412000	-2.847498000	-1.733382000
H	4.242319000	-3.340648000	-1.985027000
H	3.681806000	-0.865073000	3.727589000
H	3.207729000	0.024578000	2.236425000
H	6.027348000	-3.488705000	-2.109272000
H	5.224298000	-1.895934000	-2.304846000

Catalyst 4, IV-V TS

Ru	0.843422	-0.351056	0.464357
Cl	1.628110	0.527373	2.589442
Cl	0.263961	-1.087076	-1.780437
N	3.162750	1.219426	-0.855613
N	1.321499	2.383396	-0.924457
C	1.830359	1.175986	-0.509593
C	3.471108	2.434438	-1.475838
H	4.484007	2.655125	-1.826211
C	2.316924	3.163831	-1.519569
H	2.101243	4.161861	-1.913143
C	4.170952	0.201112	-0.630872
C	4.998869	0.301152	0.517261
C	4.851704	1.427402	1.510399
H	5.647697	1.378768	2.278667
H	3.865380	1.375910	2.022016
H	4.913686	2.420503	1.016662
C	5.989534	-0.685423	0.695504
H	6.632590	-0.624988	1.589675

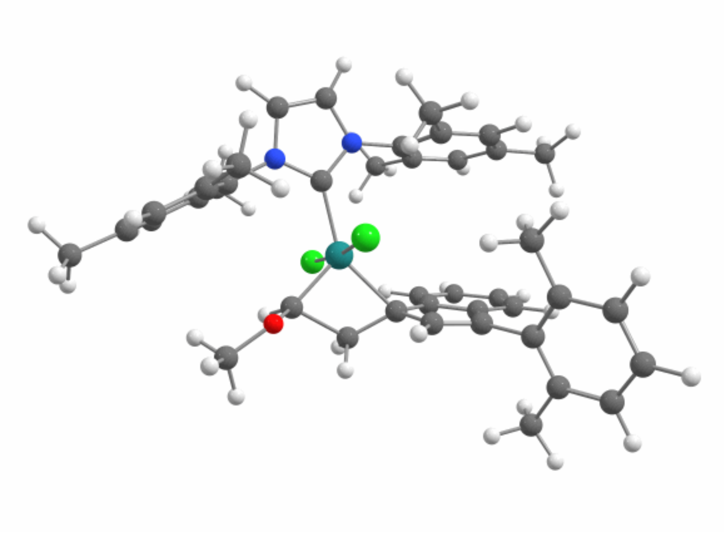

Zero-point correction= 0.720827 (Hartree/Particle)
Thermal correction to Energy= 0.770510
Thermal correction to Enthalpy= 0.771454
Thermal correction to Gibbs Free Energy= 0.635281
Sum of electronic and zero-point Energies= -2786.883452
Sum of electronic and thermal Energies= -2786.833770
Sum of electronic and thermal Enthalpies= -2786.832826
Sum of electronic and thermal Free Energies= -2786.968999

Solvent: -2788.12787566

C	6.193269	-1.723046	-0.236754
C	7.294250	-2.740877	-0.031519
H	7.388026	-3.034066	1.034450
H	8.280402	-2.328088	-0.339096
H	7.122661	-3.658408	-0.629339
C	5.383962	-1.748458	-1.388938
H	5.548924	-2.530108	-2.149386
C	4.375582	-0.790140	-1.623380
C	3.576019	-0.811917	-2.900610
H	3.941873	-1.605942	-3.580229
H	3.647260	0.157866	-3.438506
H	2.495390	-0.988325	-2.698355
C	-0.043603	2.851319	-0.778971
C	-0.926898	2.724003	-1.880312
C	-0.489553	2.094952	-3.180176
H	-1.309148	2.121139	-3.924132
H	-0.189289	1.035508	-3.021108
H	0.384970	2.621847	-3.618948
C	-2.234499	3.226796	-1.725940
H	-2.934528	3.134413	-2.572701
C	-2.665839	3.847583	-0.537119
C	-4.090805	4.327354	-0.381102
H	-4.146045	5.263682	0.211233
H	-4.700522	3.567184	0.154750
H	-4.574689	4.507395	-1.362117
C	-1.735171	3.999620	0.509504
H	-2.040691	4.515332	1.434704
C	-0.411579	3.527349	0.412561
C	0.578052	3.776190	1.524262
H	1.489977	4.287960	1.148284
H	0.915813	2.828126	1.998087
H	0.130047	4.415095	2.309884
H	-1.602061	-2.604370	-0.584544
C	-1.941697	-1.831523	0.114391
C	-3.224706	-1.369635	0.241936
C	-1.037783	-1.190102	1.109568
C	-3.243355	-0.367552	1.331609
C	-1.930427	-0.259989	1.887320
C	-4.308487	0.381088	1.853130
C	-1.704393	0.582110	2.984641
C	-4.069464	1.229688	2.955796
H	-5.315968	0.291871	1.415338
C	-2.784636	1.320505	3.518770
H	-0.693175	0.682938	3.406785
H	-4.898271	1.818184	3.381821
H	-2.610964	1.978925	4.384802
C	-4.417016	-1.805976	-0.538004
C	-4.600195	-1.340796	-1.871471
C	-5.364494	-2.690133	0.053720
C	-5.737760	-1.765682	-2.589377
C	-6.487917	-3.090414	-0.699206
C	-6.677283	-2.632263	-2.011468
H	-5.883428	-1.405041	-3.621092
H	-7.219311	-3.779209	-0.244777
H	-7.559848	-2.954613	-2.587316
C	0.047030	-2.033314	1.721554
C	1.681369	-1.986836	0.970102
H	0.222368	-1.838223	2.794853
H	-0.114621	-3.099146	1.479342
H	2.367227	-1.958472	1.850884
O	1.928537	-3.020683	0.129324
C	2.830842	-4.023819	0.613429
H	2.382439	-4.592900	1.458784
H	3.015997	-4.715284	-0.229705
H	3.794615	-3.574141	0.936862
C	-3.604159	-0.396079	-2.505152
H	-3.500554	0.537325	-1.911882
H	-2.584531	-0.832636	-2.552494
H	-3.915421	-0.119935	-3.532597
C	-5.156847	-3.225617	1.453944
H	-4.198422	-3.781439	1.533850
H	-5.105866	-2.410275	2.206020
H	-5.976598	-3.911536	1.746848

Catalyst 4, V intermediate

Ru	0.653642000	-0.029574000	-0.754936000
Cl	-0.097993000	-0.789800000	1.427316000
Cl	1.788536000	1.056561000	-2.640142000
N	3.030271000	1.424435000	0.831365000
N	1.251117000	2.651318000	0.578237000

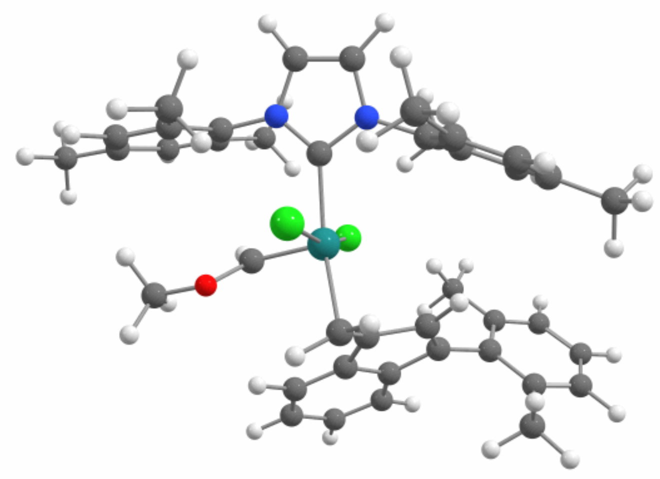

Zero-point correction= 0.720337 (Hartree/Particle)
Thermal correction to Energy= 0.771084
Thermal correction to Enthalpy= 0.772028
Thermal correction to Gibbs Free Energy= 0.632307
Sum of electronic and zero-point Energies= -2786.900438
Sum of electronic and thermal Energies= -2786.849691
Sum of electronic and thermal Enthalpies= -2786.848746
Sum of electronic and thermal Free Energies= -2786.988468

Solvent: -2788.14443088

C	1.755589000	1.394408000	0.311826000
C	3.308376000	2.670700000	1.401117000
H	4.274031000	2.884225000	1.869575000
C	2.190381000	3.440402000	1.240320000
H	1.970599000	4.470595000	1.537239000
C	4.005161000	0.353902000	0.888429000
C	4.006397000	-0.501098000	2.022463000
C	2.998246000	-0.344171000	3.133194000
H	3.212605000	-1.046723000	3.961755000
H	1.962597000	-0.531790000	2.769654000
H	3.009957000	0.686742000	3.547384000
C	5.003268000	-1.494390000	2.090516000
H	5.011087000	-2.169116000	2.962986000
C	5.998405000	-1.630574000	1.100514000
C	7.061565000	-2.700252000	1.220740000
H	7.773775000	-2.670170000	0.372349000
H	6.612654000	-3.716164000	1.252903000
H	7.646231000	-2.582136000	2.158026000
C	5.984499000	-0.730196000	0.017735000
H	6.771049000	-0.796938000	-0.752586000
C	5.006589000	0.280875000	-0.110791000
C	5.059393000	1.261146000	-1.255950000
H	5.894664000	1.019552000	-1.941564000
H	5.211876000	2.299851000	-0.890345000
H	4.108660000	1.260158000	-1.834216000
C	-0.095742000	3.112502000	0.287001000
C	-0.342845000	3.835388000	-0.908952000
C	0.765741000	4.234027000	-1.850415000
H	0.365019000	4.824782000	-2.696928000
H	1.291067000	3.342522000	-2.257384000
H	1.527162000	4.856999000	-1.333029000
C	-1.673330000	4.231122000	-1.165272000
H	-1.884679000	4.776541000	-2.100120000
C	-2.722866000	3.976562000	-0.262361000
C	-4.145539000	4.382904000	-0.574279000
H	-4.584219000	4.986958000	0.247766000
H	-4.796269000	3.490220000	-0.695116000
H	-4.208296000	4.976225000	-1.507978000
C	-2.407523000	3.353677000	0.962872000
H	-3.201563000	3.198226000	1.711796000
C	-1.101402000	2.932232000	1.276278000
C	-0.784061000	2.364768000	2.637006000
H	0.019054000	2.946131000	3.138804000
H	-0.438076000	1.311110000	2.559817000
H	-1.679224000	2.388886000	3.288008000
H	-2.634653000	0.600150000	-1.135273000
C	-2.447721000	-0.468513000	-0.954695000
C	-3.271170000	-1.321222000	-0.253097000
C	-1.272003000	-1.193656000	-1.473992000
C	-2.658188000	-2.666915000	-0.276560000
C	-1.436290000	-2.597801000	-1.010374000
C	-3.117169000	-3.896571000	0.227674000
C	-0.680871000	-3.754764000	-1.237823000
C	-2.342006000	-5.051915000	0.010649000
H	-4.073155000	-3.954521000	0.772699000
C	-1.135815000	-4.983273000	-0.715971000
H	0.252274000	-3.711233000	-1.822456000
H	-2.685926000	-6.021894000	0.404507000
H	-0.548785000	-5.900064000	-0.886450000
C	-4.592241000	-0.995342000	0.356746000
C	-4.741571000	-0.959557000	1.774378000
C	-5.712072000	-0.737019000	-0.487839000
C	-6.004880000	-0.644718000	2.319203000
C	-6.958106000	-0.433621000	0.099856000
C	-7.107265000	-0.382301000	1.493492000
H	-6.117200000	-0.606288000	3.415598000
H	-7.825251000	-0.241337000	-0.553618000
H	-8.087100000	-0.141960000	1.937026000
C	-0.452023000	-0.733117000	-2.528928000
C	1.854225000	-1.417745000	-0.716134000
H	0.169996000	-1.438742000	-3.102332000
H	-0.741839000	0.176087000	-3.079669000
H	2.026082000	-1.987729000	0.232067000
O	2.519750000	-1.903483000	-1.758818000
C	3.417263000	-3.013440000	-1.547283000
H	3.050980000	-3.668541000	-0.729072000
H	3.457100000	-3.578274000	-2.497452000
H	4.424665000	-2.624445000	-1.295678000
C	-5.591862000	-0.798307000	-1.996917000
H	-5.058363000	-1.712649000	-2.329043000
C	-3.574008000	-1.242867000	2.691980000
H	-2.648063000	-0.734586000	2.353367000
H	-3.799392000	-0.932108000	3.732046000
H	-3.328001000	-2.326715000	2.712945000
H	-6.592348000	-0.786471000	-2.474433000
H	-5.017169000	0.060915000	-2.403134000

Catalyst 4, V-VI TS

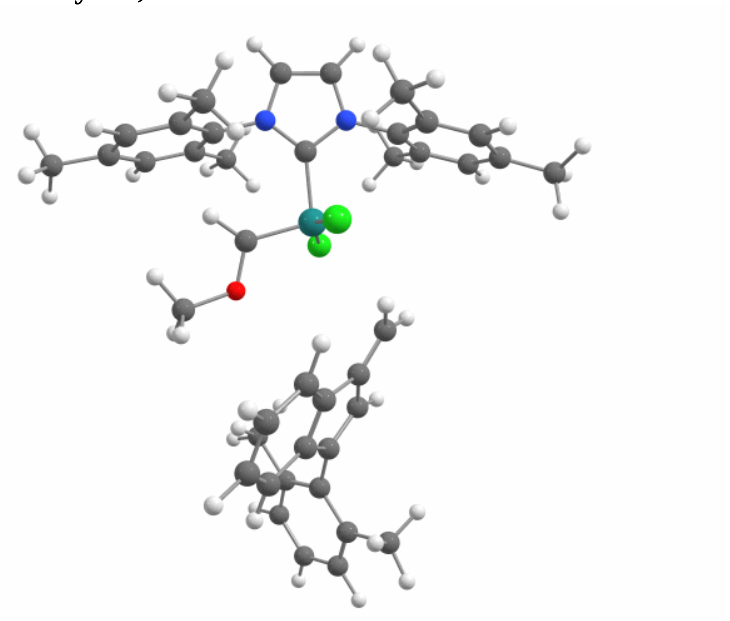

Zero-point correction= 0.718699 (Hartree/Particle)
Thermal correction to Energy= 0.770095
Thermal correction to Enthalpy= 0.771039
Thermal correction to Gibbs Free Energy= 0.624897
Sum of electronic and zero-point Energies= -2786.894785
Sum of electronic and thermal Energies= -2786.843388
Sum of electronic and thermal Enthalpies= -2786.842444
Sum of electronic and thermal Free Energies= -2786.988587

Solvent: -2788.12672217

Ru	-1.186577000	0.194935000	0.033893000
Cl	-0.514038000	0.307376000	-2.219396000
Cl	-1.246410000	0.866231000	2.305440000
N	-3.960550000	-1.030185000	-0.445797000
N	-3.925853000	1.156506000	-0.442460000
C	-3.095831000	0.045076000	-0.273654000
C	-5.265429000	-0.595010000	-0.710535000
H	-6.080820000	-1.306474000	-0.873573000
C	-5.240572000	0.769035000	-0.710126000
H	-6.031384000	1.507338000	-0.875813000
C	-3.646860000	-2.432445000	-0.351581000
C	-3.260188000	-3.130704000	-1.521674000
C	-3.122015000	-2.418961000	-2.845462000
H	-2.867584000	-3.131364000	-3.654043000
H	-2.327019000	-1.641458000	-2.800667000
H	-4.060966000	-1.898077000	-3.129486000
C	-2.984443000	-4.507622000	-1.397721000
H	-2.676652000	-5.065818000	-2.297805000
C	-3.091720000	-5.186650000	-0.166300000
C	-2.832088000	-6.674474000	-0.078192000
H	-2.521149000	-6.978205000	0.941691000
H	-2.046158000	-6.996641000	-0.791228000
H	-3.750121000	-7.252149000	-0.325524000
C	-3.480323000	-4.449982000	0.972328000
H	-3.564922000	-4.962470000	1.945355000
C	-3.766372000	-3.071022000	0.907315000
C	-4.139643000	-2.287586000	2.144252000
H	-4.216047000	-2.951221000	3.027249000
H	-5.111916000	-1.764913000	2.022360000
H	-3.383404000	-1.501986000	2.363269000
C	-3.531701000	2.545265000	-0.336787000
C	-3.697845000	3.208400000	0.908522000
C	-4.288090000	2.505538000	2.106422000
H	-4.446944000	3.217482000	2.939548000
H	-3.605734000	1.704620000	2.462516000
H	-5.264282000	2.033727000	1.865605000
C	-3.320845000	4.562367000	0.984228000
H	-3.433185000	5.085576000	1.948529000
C	-2.821084000	5.266235000	-0.130417000
C	-2.378976000	6.706419000	-0.000354000
H	-2.389077000	7.229569000	-0.977638000
H	-1.340961000	6.765981000	0.394961000
H	-3.024751000	7.271328000	0.702970000
C	-2.750588000	4.592336000	-1.364488000
H	-2.417395000	5.140618000	-2.261489000
C	-3.117501000	3.237480000	-1.504536000
C	-3.107305000	2.579636000	-2.862644000
H	-4.057828000	2.042239000	-3.063991000
H	-2.288628000	1.830732000	-2.940597000
H	-2.963040000	3.334362000	-3.660171000
H	3.919997000	1.865508000	-1.439181000
C	3.934250000	1.301247000	-0.496027000
C	4.954320000	0.486260000	-0.061712000
C	2.846501000	1.334025000	0.494621000
C	4.564417000	-0.075967000	1.254458000
C	3.276755000	0.436265000	1.597706000
C	5.230495000	-0.967363000	2.110800000
C	2.652587000	0.055609000	2.792061000
C	4.599569000	-1.343334000	3.315824000
H	6.223849000	-1.364188000	1.845412000
C	3.326726000	-0.840360000	3.651819000
H	1.650030000	0.433588000	3.050729000
H	5.109245000	-2.038346000	4.002894000
H	2.852064000	-1.145373000	4.598354000
C	6.241258000	0.196934000	-0.755591000
C	6.301432000	-0.825719000	-1.743515000
C	7.407132000	0.939829000	-0.415019000
C	7.531951000	-1.084099000	-2.383109000
C	8.617975000	0.650773000	-1.078080000
C	8.683438000	-0.352491000	-2.056500000
H	7.581341000	-1.876370000	-3.148441000
H	9.521480000	1.227084000	-0.818610000
H	9.637148000	-0.566779000	-2.565224000
C	1.699081000	2.062281000	0.413527000
C	-0.733279000	-1.552197000	0.336938000
H	0.948780000	2.051311000	1.221295000
H	1.495066000	2.695751000	-0.463965000
H	-1.399905000	-2.433309000	0.483101000
O	0.566546000	-1.835897000	0.454438000
C	0.994213000	-3.146289000	0.867866000
H	1.688988000	-3.540169000	0.100390000
H	1.529012000	-3.047214000	1.833368000
H	0.122926000	-3.826589000	0.977681000
C	7.346666000	2.032665000	0.629854000
H	6.983202000	1.645774000	1.605322000
C	5.068908000	-1.627061000	-2.103782000
H	4.283247000	-0.989953000	-2.561617000
H	8.342618000	2.491375000	0.790919000
H	6.640300000	2.836873000	0.333914000
H	5.313240000	-2.436928000	-2.819927000
H	4.606958000	-2.086423000	-1.204569000

Catalyst 5, I intermediate

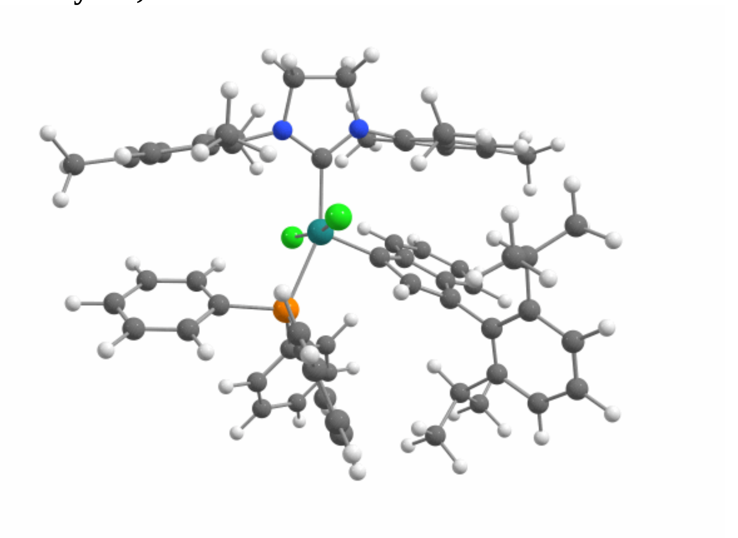

Zero-point correction= 1.036344 (Hartree/Particle)
Thermal correction to Energy= 1.105058
Thermal correction to Enthalpy= 1.106002
Thermal correction to Gibbs Free Energy= 0.928283
Sum of electronic and zero-point Energies= -3787.650717
Sum of electronic and thermal Energies= -3787.582003
Sum of electronic and thermal Enthalpies= -3787.581058
Sum of electronic and thermal Free Energies= -3787.758778

Solvent: -3789.42559716

Ru	0.970495000	0.399445000	0.048190000
Cl	1.723281000	0.424355000	2.344898000
Cl	0.674413000	0.426096000	-2.367951000
P	1.217385000	-2.018660000	0.068406000
N	2.863918000	2.668589000	-0.668776000
N	0.845877000	3.511605000	-0.412065000
C	1.582388000	2.373089000	-0.287649000
C	2.996002000	4.040292000	-1.214160000
H	3.164646000	3.996018000	-2.312124000
H	3.865120000	4.555228000	-0.755145000
C	1.649955000	4.676708000	-0.853412000
H	1.726633000	5.417894000	-0.027066000
H	1.160194000	5.180113000	-1.711793000
C	4.070854000	1.883593000	-0.563256000
C	4.805287000	1.939629000	0.654239000
C	4.299182000	2.700958000	1.854644000
H	5.104075000	2.833772000	2.604043000
H	3.456697000	2.156741000	2.336254000
H	3.919034000	3.706583000	1.577464000
C	6.055649000	1.291097000	0.711334000
H	6.626385000	1.332246000	1.654455000
C	6.607090000	0.625693000	-0.399070000
C	7.959513000	-0.046427000	-0.325702000
H	7.883316000	-1.124434000	-0.582265000
H	8.404684000	0.033096000	0.686146000
H	8.674401000	0.405209000	-1.046781000
C	5.873917000	0.629073000	-1.601011000
H	6.297328000	0.136468000	-2.492662000
C	4.623737000	1.266987000	-1.721174000
C	3.954924000	1.345209000	-3.072727000
H	4.327870000	0.543515000	-3.740937000
H	4.189482000	2.311898000	-3.573374000
H	2.851890000	1.252401000	-3.000917000
C	-0.490457000	3.795276000	0.040488000
C	-1.545943000	3.837393000	-0.904115000
C	-1.316543000	3.477031000	-2.352024000
H	-2.260803000	3.538506000	-2.927061000
H	-0.892194000	2.455657000	-2.460085000
H	-0.592576000	4.169023000	-2.836221000
C	-2.816017000	4.255746000	-0.458092000
H	-3.645581000	4.284529000	-1.183679000
C	-3.051890000	4.647595000	0.873902000
C	-4.432416000	5.053611000	1.337040000
H	-4.390639000	5.879916000	2.076422000
H	-4.944348000	4.199666000	1.832873000
H	-5.072138000	5.377139000	0.491400000
C	-1.967652000	4.627330000	1.773366000
H	-2.126558000	4.943429000	2.817826000
C	-0.680414000	4.212454000	1.383456000
C	0.464733000	4.234071000	2.370457000
H	1.213312000	5.016883000	2.115571000
H	1.007435000	3.266146000	2.400549000
H	0.099487000	4.455990000	3.392272000
H	-1.557239000	-0.792856000	-1.526327000
C	-1.853471000	-0.335270000	-0.575737000
C	-3.137828000	-0.284271000	-0.073255000
C	-0.888007000	0.297506000	0.351239000
C	-3.082629000	0.414327000	1.235048000
C	-1.718887000	0.742627000	1.522903000
C	-4.110855000	0.739855000	2.125468000
C	-1.403972000	1.340263000	2.749019000
C	-3.778963000	1.353833000	3.356886000
H	-5.158390000	0.511389000	1.871991000
C	-2.439907000	1.631238000	3.669603000
H	-0.355510000	1.551927000	2.997756000
H	-4.576214000	1.602744000	4.075953000
H	-2.186722000	2.088932000	4.639242000
C	-4.393423000	-0.827050000	-0.670126000
C	-4.959269000	-0.235401000	-1.842161000
C	-5.048134000	-1.936015000	-0.046014000
C	-6.161328000	-0.762940000	-2.358985000
C	-6.251553000	-2.420122000	-0.597854000
C	-6.809860000	-1.841767000	-1.745139000
H	-6.602938000	-0.313859000	-3.263180000
H	-6.755780000	-3.279229000	-0.126135000
H	-7.749669000	-2.235746000	-2.164727000
C	3.019613000	-2.484512000	-0.060069000
C	3.502053000	-3.431470000	-0.991001000
C	3.911312000	-1.939498000	0.893040000
C	4.849025000	-3.835165000	-0.959953000
H	2.823828000	-3.875146000	-1.734378000
C	5.251203000	-2.358620000	0.926724000
H	3.544056000	-1.196353000	1.620529000
C	5.724580000	-3.308808000	0.004564000
H	5.208554000	-4.579334000	-1.688798000
H	5.929150000	-1.936198000	1.684778000
H	6.774265000	-3.642598000	0.039820000

C	0.419134000	-3.032569000	-1.267065000
C	-0.501737000	-4.059892000	-0.961083000
C	0.730961000	-2.777669000	-2.624902000
C	-1.094575000	-4.816692000	-1.987475000
H	-0.756919000	-4.278315000	0.086008000
C	0.138115000	-3.540748000	-3.645337000
H	1.424043000	-1.965228000	-2.883503000
C	-0.777290000	-4.560332000	-3.331907000
H	-1.807889000	-5.615462000	-1.729142000
H	0.392328000	-3.328003000	-4.696037000
H	-1.242623000	-5.154715000	-4.134437000
C	0.735106000	-2.932789000	1.617615000
C	1.368234000	-4.151028000	1.955604000
C	-0.286510000	-2.435825000	2.452695000
C	0.977893000	-4.861154000	3.103092000
H	2.174361000	-4.549001000	1.319974000
C	-0.675197000	-3.149848000	3.600483000
H	-0.772073000	-1.477746000	2.218212000
C	-0.044981000	-4.361922000	3.929158000
H	1.481284000	-5.808429000	3.354757000
H	-1.468101000	-2.742349000	4.247213000
H	-0.345214000	-4.915010000	4.833659000
C	-4.441967000	-2.677196000	1.150846000
H	-3.526896000	-2.129952000	1.459414000
C	-4.306303000	0.947813000	-2.561060000
H	-3.488943000	1.322382000	-1.910815000
C	-3.665675000	0.502363000	-3.893715000
H	-3.165140000	1.356891000	-4.396298000
H	-2.903204000	-0.286404000	-3.733829000
H	-4.429916000	0.098783000	-4.592548000
C	-5.291677000	2.114624000	-2.780509000
H	-6.115327000	1.842742000	-3.474551000
H	-5.751330000	2.443737000	-1.825739000
H	-4.768650000	2.986334000	-3.228364000
C	-5.387665000	-2.727203000	2.367920000
H	-6.318987000	-3.289434000	2.142070000
H	-4.894417000	-3.236712000	3.222544000
H	-5.679647000	-1.711490000	2.702904000
C	-3.999828000	-4.099223000	0.741107000
H	-3.469883000	-4.602122000	1.577685000
H	-4.871558000	-4.730481000	0.464935000
H	-3.317592000	-4.067163000	-0.131680000

Catalyst 5, I-II TS

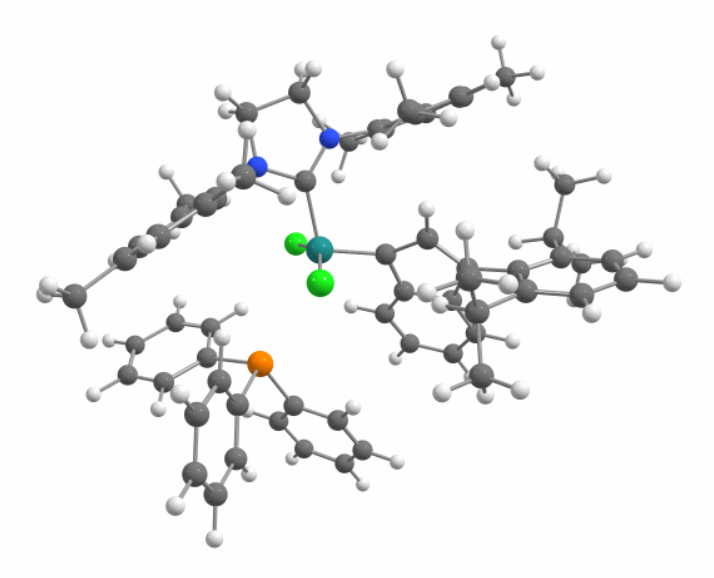

Zero-point correction= 1.035018 (Hartree/Particle)
Thermal correction to Energy= 1.103740
Thermal correction to Enthalpy= 1.104684
Thermal correction to Gibbs Free Energy= 0.923503
Sum of electronic and zero-point Energies= -3787.628677
Sum of electronic and thermal Energies= -3787.559955
Sum of electronic and thermal Enthalpies= -3787.559011
Sum of electronic and thermal Free Energies= -3787.740192

Ru	0.229198000	-0.808130000	-0.305040000
Cl	0.881534000	-1.160699000	-2.540492000
Cl	0.490635000	-0.161154000	1.942916000
P	3.126697000	1.397376000	-0.440014000
N	0.760219000	-3.553644000	0.385549000
N	-1.320880000	-3.458460000	-0.363550000
C	-0.227823000	-2.683433000	-0.043601000
C	0.409186000	-4.967275000	0.165929000
H	0.695196000	-5.582579000	1.043464000
H	0.948672000	-5.367411000	-0.722762000
C	-1.102745000	-4.890725000	-0.053728000
H	-1.456283000	-5.521334000	-0.894456000
H	-1.679721000	-5.180365000	0.853700000
C	2.055876000	-3.242748000	0.935496000
C	3.204401000	-3.219793000	0.100988000
C	3.144922000	-3.551355000	-1.371968000
H	3.953619000	-3.032459000	-1.923403000
H	2.184589000	-3.260670000	-1.838447000
H	3.295079000	-4.643827000	-1.526208000
C	4.456308000	-2.960514000	0.699857000
H	5.346648000	-2.909523000	0.051482000
C	4.603504000	-2.787091000	2.087403000
C	5.950591000	-2.473800000	2.697333000
H	6.058723000	-2.921961000	3.706407000
H	6.080900000	-1.374917000	2.811340000
H	6.783704000	-2.838443000	2.063385000
C	3.452274000	-2.904956000	2.895099000
H	3.549943000	-2.819795000	3.990629000
C	2.177201000	-3.139162000	2.350450000
C	0.978669000	-3.305138000	3.253491000
H	1.284475000	-3.304710000	4.318125000
H	0.439780000	-4.256793000	3.056063000
H	0.260123000	-2.473347000	3.100939000
C	-2.660317000	-3.129485000	-0.776746000
C	-3.681167000	-3.055616000	0.206040000
C	-3.374214000	-3.118648000	1.687211000
H	-4.110425000	-2.527719000	2.268345000
H	-2.361480000	-2.738615000	1.926439000
H	-3.436579000	-4.162303000	2.069215000
C	-5.022730000	-2.974610000	-0.222314000
H	-5.818307000	-2.923590000	0.540237000
C	-5.373148000	-2.985867000	-1.584927000
C	-6.819307000	-2.995285000	-2.027719000
H	-7.122567000	-4.013130000	-2.358682000
H	-6.989140000	-2.315090000	-2.887519000
H	-7.504400000	-2.694996000	-1.210522000
C	-4.332763000	-3.043974000	-2.534519000
H	-4.583184000	-3.052714000	-3.608847000
C	-2.978074000	-3.132311000	-2.162852000

Solvent: -3789.38920398	C	-1.908215000	-3.281271000	-3.215279000
	H	-1.343787000	-4.230991000	-3.089394000
	H	-1.155590000	-2.467434000	-3.158370000
	H	-2.351179000	-3.285479000	-4.230375000
	H	-2.801287000	-0.657050000	1.091690000
	C	-2.566808000	0.119224000	0.357577000
	C	-3.223170000	1.334407000	0.260661000
	C	-1.388877000	0.102036000	-0.510775000
	C	-2.550854000	2.133742000	-0.794780000
	C	-1.442738000	1.378313000	-1.293001000
	C	-2.832784000	3.406746000	-1.298548000
	C	-0.656720000	1.889266000	-2.333409000
	C	-2.033448000	3.916347000	-2.351342000
	H	-3.664108000	4.000034000	-0.885704000
	C	-0.969658000	3.162968000	-2.868541000
	H	0.186345000	1.304301000	-2.729567000
	H	-2.252214000	4.913562000	-2.765826000
	H	-0.355275000	3.569887000	-3.686344000
	C	-4.320796000	1.853709000	1.126075000
	C	-5.614537000	2.102215000	0.569558000
	C	-4.065384000	2.145894000	2.504231000
	C	-6.628780000	2.618964000	1.401150000
	C	-5.116872000	2.661054000	3.291725000
	C	-6.388168000	2.896378000	2.753519000
	H	-7.631846000	2.799944000	0.982100000
	H	-4.928513000	2.891512000	4.352908000
	H	-7.193693000	3.299235000	3.388697000
	C	3.887702000	1.895072000	1.184690000
	C	4.519644000	3.142027000	1.399492000
	C	3.822021000	0.970024000	2.252024000
	C	5.081680000	3.451789000	2.650338000
	H	4.572439000	3.878608000	0.582683000
	C	4.390676000	1.281527000	3.500015000
	H	3.300790000	0.010326000	2.112510000
	C	5.021291000	2.521657000	3.703343000
	H	5.569896000	4.428200000	2.802024000
	H	4.322482000	0.553639000	4.324494000
	H	5.457493000	2.767833000	4.684941000
	C	2.911995000	3.057324000	-1.251895000
	C	3.690565000	3.516906000	-2.337129000
	C	1.883522000	3.890671000	-0.749782000
	C	3.453318000	4.785149000	-2.898679000
	H	4.494561000	2.883453000	-2.742399000
	C	1.661257000	5.162889000	-1.300508000
	H	1.253129000	3.539380000	0.083879000
	C	2.444461000	5.614201000	-2.379034000
	H	4.070882000	5.129405000	-3.744196000
	H	0.861700000	5.800563000	-0.891261000
	H	2.265377000	6.609880000	-2.815699000
	C	4.588259000	0.696566000	-1.357317000
	C	5.916455000	0.758416000	-0.878532000
	C	4.337177000	0.045346000	-2.589204000
	C	6.969861000	0.191451000	-1.618536000
	H	6.130276000	1.257501000	0.079179000
	C	5.394656000	-0.506775000	-3.332920000
	H	3.301876000	-0.044288000	-2.956748000
	C	6.714075000	-0.438280000	-2.849293000
	H	8.000016000	0.249037000	-1.230779000
	H	5.182461000	-1.003270000	-4.293702000
	H	7.541373000	-0.877591000	-3.429729000
	C	-2.688027000	1.973094000	3.149940000
	H	-1.996580000	1.561976000	2.388595000
	C	-5.955362000	1.752883000	-0.882318000
	H	-5.009685000	1.479666000	-1.395423000
	C	-2.719354000	0.961113000	4.313914000
	H	-3.121946000	-0.019758000	3.986562000
	H	-1.694230000	0.791686000	4.704394000
	H	-3.349316000	1.317742000	5.157578000
	C	-2.103102000	3.328394000	3.603563000
	H	-1.074158000	3.191796000	3.997699000
	H	-2.051224000	4.046941000	2.759619000
	H	-2.712678000	3.795818000	4.406868000
	C	-6.875897000	0.515446000	-0.940017000
	H	-7.089264000	0.233605000	-1.992648000
	H	-6.404364000	-0.355093000	-0.441637000
	H	-7.847944000	0.712564000	-0.438881000
	C	-6.576893000	2.934081000	-1.655117000
	H	-6.735270000	2.660449000	-2.719631000
	H	-7.565406000	3.225804000	-1.240506000
	H	-5.925194000	3.831123000	-1.630449000

**Catalyst 1, II intermediate**	Ru	-1.485517000	-0.662920000	-0.388532000
	Cl	-1.931623000	-0.651875000	-2.677874000
	Cl	-1.875790000	-1.627847000	1.687953000
	N	-3.488056000	1.381900000	-0.082738000
	N	-1.519259000	2.385064000	-0.241350000
	C	-2.124600000	1.156017000	-0.164147000
	C	-3.839358000	2.797196000	-0.296932000
	H	-4.594174000	3.129255000	0.445292000
	H	-4.272811000	2.939459000	-1.312369000
	C	-2.485438000	3.501120000	-0.140283000
	H	-2.290505000	4.251827000	-0.933071000

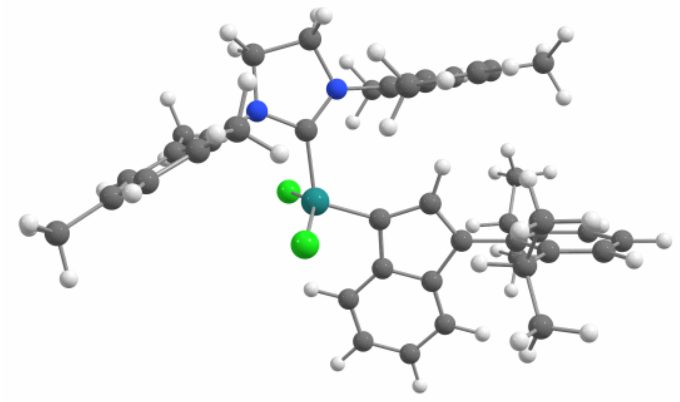

Zero-point correction= 0.767762 (Hartree/Particle)
Thermal correction to Energy= 0.819329
Thermal correction to Enthalpy= 0.820274
Thermal correction to Gibbs Free Energy= 0.677291
Sum of electronic and zero-point Energies= -2752.176357
Sum of electronic and thermal Energies= -2752.124790
Sum of electronic and thermal Enthalpies= -2752.123846
Sum of electronic and thermal Free Energies= -2752.266828

Solvent: -2753.43214969

H	-2.378941000	4.014838000	0.842536000
C	-4.527412000	0.422759000	0.197654000
C	-5.332495000	-0.096716000	-0.851232000
C	-5.196937000	0.372031000	-2.280778000
H	-5.579421000	-0.391858000	-2.986308000
H	-4.145856000	0.585252000	-2.554106000
H	-5.798804000	1.294305000	-2.445434000
C	-6.344510000	-1.020412000	-0.514176000
H	-6.956969000	-1.446300000	-1.326781000
C	-6.606651000	-1.397556000	0.815482000
C	-7.664362000	-2.426857000	1.143456000
H	-8.195575000	-2.182739000	2.086424000
H	-7.207239000	-3.431632000	1.280821000
H	-8.416844000	-2.516110000	0.334273000
C	-5.850564000	-0.787519000	1.837484000
H	-6.071166000	-1.029029000	2.890797000
C	-4.822745000	0.130379000	1.559442000
C	-4.076560000	0.804537000	2.685924000
H	-4.559248000	0.591732000	3.659797000
H	-4.041708000	1.907089000	2.555409000
H	-3.030195000	0.438942000	2.736836000
C	-0.128854000	2.734957000	-0.176529000
C	0.462810000	2.956659000	1.095756000
C	-0.279143000	2.625126000	2.370465000
H	0.346125000	2.836250000	3.259486000
H	-0.566710000	1.552671000	2.397047000
H	-1.217726000	3.210141000	2.476766000
C	1.778620000	3.456718000	1.139422000
H	2.245931000	3.632132000	2.123048000
C	2.510649000	3.737678000	-0.033584000
C	3.929959000	4.252422000	0.048800000
H	4.246542000	4.735258000	-0.897238000
H	4.640259000	3.421398000	0.251235000
H	4.049557000	4.988405000	0.870402000
C	1.891231000	3.496512000	-1.275797000
H	2.444525000	3.713317000	-2.204820000
C	0.574824000	3.000165000	-1.376628000
C	-0.066566000	2.764546000	-2.723313000
H	-0.951482000	3.420210000	-2.876377000
H	-0.428315000	1.719268000	-2.824315000
H	0.647244000	2.971540000	-3.544286000
H	1.571611000	0.902900000	0.420322000
C	1.554081000	-0.124275000	0.046096000
C	2.653494000	-0.969305000	0.008826000
C	0.369942000	-0.822896000	-0.429085000
C	2.222409000	-2.276562000	-0.533317000
C	0.829008000	-2.192385000	-0.839330000
C	2.937480000	-3.458709000	-0.757515000
C	0.169153000	-3.290715000	-1.409283000
C	2.258916000	-4.568976000	-1.312690000
H	4.007989000	-3.521362000	-0.503925000
C	0.896026000	-4.483245000	-1.639748000
H	-0.893572000	-3.235244000	-1.693239000
H	2.807438000	-5.506601000	-1.496089000
H	0.382822000	-5.350784000	-2.083410000
C	4.046101000	-0.668600000	0.450892000
C	5.046592000	-0.372764000	-0.523800000
C	4.378254000	-0.707138000	1.838419000
C	6.357460000	-0.090284000	-0.086828000
C	5.703691000	-0.417313000	2.225243000
C	6.687103000	-0.106900000	1.275924000
H	7.136634000	0.146899000	-0.829692000
H	5.972696000	-0.440761000	3.293910000
H	7.716819000	0.116769000	1.598777000
C	4.728211000	-0.338244000	-2.021673000
H	3.660432000	-0.619362000	-2.141281000
C	3.348148000	-1.081748000	2.907777000
H	2.386652000	-1.277182000	2.389282000
C	3.101557000	0.075289000	3.898003000
H	4.017167000	0.324579000	4.476493000
H	2.778001000	0.994774000	3.367825000
H	2.309280000	-0.196281000	4.627227000
C	3.740592000	-2.379635000	3.645469000
H	2.948697000	-2.672332000	4.366861000
H	3.879806000	-3.220326000	2.935202000
H	4.686089000	-2.259385000	4.216797000
C	4.889923000	1.081804000	-2.604148000
H	5.941022000	1.435159000	-2.531807000
H	4.604030000	1.100107000	-3.677332000
H	4.248614000	1.809716000	-2.067086000
C	5.565761000	-1.362186000	-2.815972000
H	5.425680000	-2.391071000	-2.426550000
H	5.269657000	-1.361960000	-3.886278000
H	6.651135000	-1.128701000	-2.770858000

Catalyst 5, II-III TS

Ru	-1.228260000	0.237043000	-0.285706000
Cl	-1.951465000	1.318699000	1.646515000
Cl	-1.060175000	0.243324000	-2.623895000
N	-3.322416000	-1.700101000	-0.480860000
N	-1.415544000	-2.822114000	-0.306283000
C	-1.965898000	-1.562594000	-0.263229000
C	-3.699534000	-3.069377000	-0.872806000
H	-3.939910000	-3.111967000	-1.958534000
H	-4.597209000	-3.401626000	-0.311353000

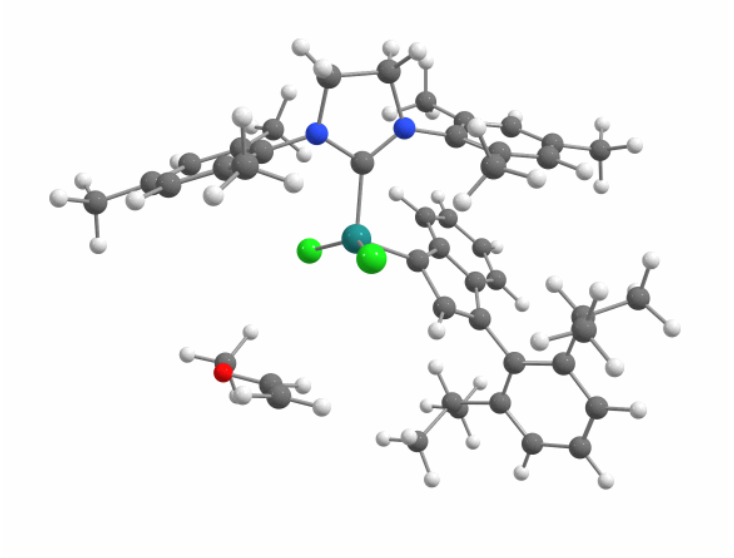

Zero-point correction= 0.849710 (Hartree/Particle)
Thermal correction to Energy= 0.907920
Thermal correction to Enthalpy= 0.908864
Thermal correction to Gibbs Free Energy= 0.748595
Sum of electronic and zero-point Energies= -2945.057083
Sum of electronic and thermal Energies= -2944.998873
Sum of electronic and thermal Enthalpies= -2944.997929
Sum of electronic and thermal Free Energies= -2945.158198

Solvent: -2946.48454111

C	-2.436215000	-3.872039000	-0.525763000
H	-2.561450000	-4.490424000	0.390718000
H	-2.115124000	-4.547857000	-1.344793000
C	-4.326690000	-0.681582000	-0.311764000
C	-4.811464000	-0.430855000	1.001734000
C	-4.286206000	-1.205806000	2.186952000
H	-4.859964000	-0.963907000	3.102763000
H	-3.221887000	-0.958224000	2.379317000
H	-4.352685000	-2.302913000	2.022822000
C	-5.810984000	0.542967000	1.170913000
H	-6.178116000	0.751903000	2.189718000
C	-6.358323000	1.249037000	0.080293000
C	-7.393109000	2.330240000	0.294452000
H	-8.069574000	2.430012000	-0.578456000
H	-6.903999000	3.318171000	0.442728000
H	-8.011989000	2.133807000	1.193578000
C	-5.904551000	0.925736000	-1.212016000
H	-6.344378000	1.440382000	-2.082735000
C	-4.905339000	-0.043023000	-1.441253000
C	-4.525769000	-0.413136000	-2.855522000
H	-4.839627000	0.376766000	-3.565876000
H	-5.033127000	-1.352698000	-3.170547000
H	-3.432278000	-0.556242000	-2.969298000
C	-0.075356000	-3.261159000	-0.017353000
C	0.884849000	-3.328909000	-1.062254000
C	0.544311000	-2.922825000	-2.474275000
H	1.400845000	-3.101556000	-3.153115000
H	0.252141000	-1.854653000	-2.548251000
H	-0.318856000	-3.505211000	-2.864057000
C	2.161400000	-3.840939000	-0.757998000
H	2.910556000	-3.894998000	-1.565562000
C	2.499713000	-4.311637000	0.526717000
C	3.891871000	-4.816706000	0.830126000
H	4.369967000	-5.264696000	-0.064591000
H	3.886415000	-5.576588000	1.637756000
H	4.547009000	-3.984231000	1.168483000
C	1.503828000	-4.287002000	1.519768000
H	1.735232000	-4.674935000	2.525988000
C	0.213170000	-3.778549000	1.272852000
C	-0.829939000	-3.847894000	2.366939000
H	-1.344523000	-4.835207000	2.366268000
H	-1.609615000	-3.068440000	2.261855000
H	-0.363592000	-3.735024000	3.365778000
H	1.422679000	1.294194000	-1.552256000
C	1.600860000	0.823838000	-0.576978000
C	2.816532000	0.775481000	0.084022000
C	0.573606000	0.100597000	0.189054000
C	2.612666000	0.046458000	1.359851000
C	1.241481000	-0.357088000	1.431199000
C	3.483704000	-0.182870000	2.429938000
C	0.734510000	-0.900707000	2.613764000
C	2.972243000	-0.786273000	3.605285000
H	4.539075000	0.128667000	2.370624000
C	1.612397000	-1.121157000	3.702965000
H	-0.336844000	-1.129133000	2.697125000
H	3.643448000	-0.967133000	4.460160000
H	1.222273000	-1.554000000	4.637987000
C	4.109934000	1.382042000	-0.348547000
C	4.952162000	0.701550000	-1.276588000
C	4.495452000	2.650110000	0.181766000
C	6.161755000	1.311826000	-1.668047000
C	5.717015000	3.217294000	-0.236235000
C	6.545926000	2.558154000	-1.154408000
H	6.817489000	0.798919000	-2.389754000
H	6.020935000	4.199527000	0.160274000
H	7.495514000	3.018307000	-1.472021000
C	-1.538029000	4.510998000	-0.626707000
C	-1.427297000	4.280213000	-1.950853000
H	-0.460285000	3.973532000	-2.370702000
H	-2.292270000	4.379164000	-2.624025000
H	-0.674112000	4.398007000	0.057144000
O	-2.704410000	4.894690000	-0.039536000
C	-2.651936000	5.081189000	1.370854000
H	-3.666574000	5.385070000	1.693025000
H	-2.372995000	4.139958000	1.894095000
H	-1.934519000	5.887229000	1.651895000
C	3.591033000	3.425656000	1.144280000
H	2.752700000	2.754693000	1.427742000
C	4.558379000	-0.653486000	-1.869380000
H	3.701732000	-1.037278000	-1.274451000
C	4.313630000	3.825998000	2.446328000
H	5.147040000	4.536122000	2.258316000
H	3.608424000	4.323760000	3.144806000
H	4.735865000	2.940726000	2.964818000
C	2.969217000	4.654981000	0.446465000
H	2.405240000	4.360855000	-0.462253000
H	2.269701000	5.183178000	1.128660000
H	3.750006000	5.382061000	0.136076000
C	5.690624000	-1.695553000	-1.768254000
H	5.337703000	-2.688330000	-2.119121000
H	6.566192000	-1.423271000	-2.395342000
H	6.047313000	-1.809082000	-0.723753000
C	4.070471000	-0.498392000	-3.326336000
H	3.758356000	-1.478218000	-3.746484000
H	3.203444000	0.189890000	-3.392352000
H	4.873885000	-0.092213000	-3.977756000

Catalyst 5, III intermediate

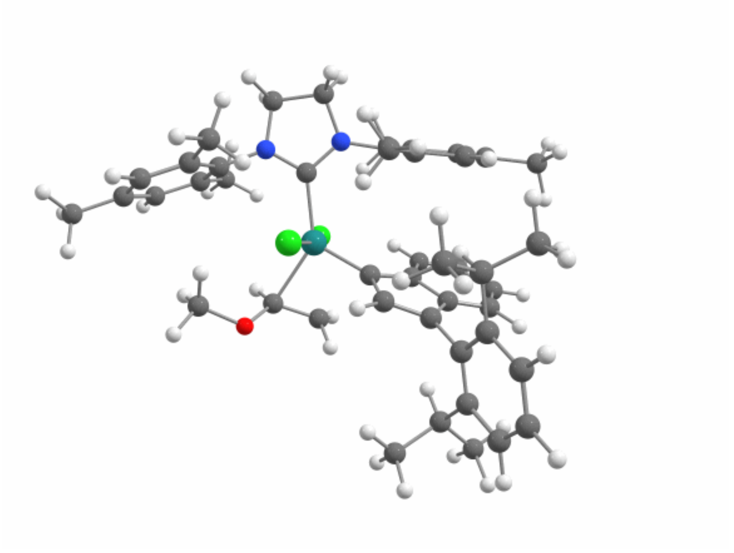

Zero-point correction= 0.851854 (Hartree/Particle)
Thermal correction to Energy= 0.909191
Thermal correction to Enthalpy= 0.910135
Thermal correction to Gibbs Free Energy= 0.757000
Sum of electronic and zero-point Energies= -2945.068605
Sum of electronic and thermal Energies= -2945.011268
Sum of electronic and thermal Enthalpies= -2945.010324
Sum of electronic and thermal Free Energies= -2945.163459

Solvent: -2946.49897354

Ru	-1.482215000	-0.498282000	-0.100449000
Cl	-2.244906000	-0.707862000	-2.362539000
Cl	-0.961581000	-0.360623000	2.294889000
N	-3.352249000	1.732837000	-0.101846000
N	-1.341594000	2.629496000	-0.243232000
C	-2.006294000	1.445252000	-0.151116000
C	-3.640301000	3.180079000	-0.133460000
H	-4.102629000	3.503486000	0.825689000
H	-4.356443000	3.415813000	-0.947832000
C	-2.252435000	3.794243000	-0.363611000
H	-2.143969000	4.251385000	-1.371652000
H	-1.987046000	4.569786000	0.384617000
C	-4.440539000	0.809848000	0.113246000
C	-5.251074000	0.411144000	-0.986123000
C	-5.072194000	0.989950000	-2.369191000
H	-5.443664000	0.288866000	-3.142063000
H	-4.010813000	1.202413000	-2.593289000
H	-5.660228000	1.930485000	-2.471768000
C	-6.306218000	-0.490049000	-0.742012000
H	-6.920551000	-0.821830000	-1.596025000
C	-6.609736000	-0.962396000	0.549513000
C	-7.716586000	-1.968287000	0.771345000
H	-8.158919000	-1.875437000	1.783971000
H	-7.331330000	-3.007707000	0.677232000
H	-8.529302000	-1.855173000	0.025266000
C	-5.855724000	-0.461140000	1.628661000
H	-6.119590000	-0.759723000	2.657322000
C	-4.787162000	0.440527000	1.445556000
C	-4.083885000	1.024884000	2.646127000
H	-4.619381000	0.757942000	3.578226000
H	-4.036107000	2.133143000	2.591189000
H	-3.036625000	0.658054000	2.729946000
C	0.062369000	2.933162000	-0.273464000
C	0.680246000	3.360691000	0.932601000
C	-0.034845000	3.253125000	2.258902000
H	0.592505000	3.653285000	3.079255000
H	-0.289448000	2.194757000	2.490112000
H	-0.993120000	3.816416000	2.265750000
C	1.984638000	3.882479000	0.857681000
H	2.470554000	4.225629000	1.786757000
C	2.672325000	4.005821000	-0.367685000
C	4.048748000	4.632627000	-0.407269000
H	4.423571000	4.739885000	-1.444238000
H	4.786867000	4.024419000	0.158088000
H	4.040913000	5.641381000	0.058021000
C	2.037782000	3.539850000	-1.535794000
H	2.562232000	3.615780000	-2.502801000
C	0.733723000	3.000635000	-1.517864000
C	0.064603000	2.535052000	-2.790035000
H	-0.776522000	3.203436000	-3.079619000
H	-0.364580000	1.516693000	-2.681779000
H	0.781558000	2.526658000	-3.633956000
H	1.407461000	0.738720000	0.949195000
C	1.493912000	-0.093420000	0.243710000
C	2.663008000	-0.776188000	-0.046891000
C	0.355387000	-0.660919000	-0.488170000
C	2.328511000	-1.841856000	-1.023697000
C	0.938747000	-1.737839000	-1.356755000
C	3.120921000	-2.864773000	-1.556723000
C	0.384642000	-2.625268000	-2.288875000
C	2.549443000	-3.758506000	-2.494545000
H	4.174153000	-2.968854000	-1.251682000
C	1.204515000	-3.624259000	-2.869163000
H	-0.675371000	-2.534521000	-2.564684000
H	3.168044000	-4.558137000	-2.933080000
H	0.769448000	-4.312598000	-3.611199000
C	4.010566000	-0.584701000	0.557266000
C	5.140772000	-0.278637000	-0.270377000
C	4.187996000	-0.739707000	1.971712000
C	6.406070000	-0.120609000	0.330756000
C	5.477130000	-0.569975000	2.518766000
C	6.580173000	-0.261021000	1.713879000
H	7.275390000	0.128478000	-0.299013000
H	5.619001000	-0.690676000	3.605032000
H	7.577779000	-0.131265000	2.163895000
C	-2.553434000	-2.457641000	0.249729000
C	-1.438213000	-3.153809000	0.693656000
H	-3.159416000	-1.948413000	1.023260000
H	-3.068322000	-2.780236000	-0.669130000
H	-0.966255000	-2.917617000	1.666747000
O	-0.921848000	-4.163631000	-0.016564000
C	0.312651000	-4.720809000	0.459368000
H	0.245411000	-4.953527000	1.544202000
H	0.477369000	-5.653025000	-0.110535000
H	1.153965000	-4.021811000	0.271239000
C	3.048311000	-1.121126000	2.922494000
H	2.130831000	-1.270729000	2.319865000
C	5.020498000	-0.035745000	-1.779987000
H	3.993315000	-0.316032000	-2.089437000
C	6.003475000	-0.890022000	-2.606999000
H	5.896668000	-1.972529000	-2.391484000
H	5.823740000	-0.742403000	-3.692790000
H	7.060717000	-0.612161000	-2.409309000
C	5.194266000	1.461350000	-2.111389000
H	6.207575000	1.824129000	-1.835101000
H	5.057619000	1.639782000	-3.199484000
H	4.450614000	2.074864000	-1.567238000

C	2.739470000	0.010544000	3.924585000
H	2.498569000	0.957477000	3.400035000
H	1.865285000	-0.256409000	4.554084000
H	3.600307000	0.208603000	4.599529000
C	3.334533000	-2.450504000	3.654106000
H	3.533617000	-3.273893000	2.936907000
H	4.215096000	-2.375425000	4.327707000
H	2.464149000	-2.742582000	4.279138000

Catalyst 5, III-IV TS

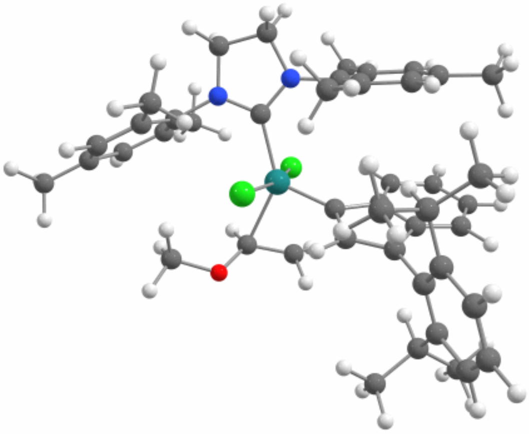

Zero-point correction= 0.852114 (Hartree/Particle)
Thermal correction to Energy= 0.908216
Thermal correction to Enthalpy= 0.909160
Thermal correction to Gibbs Free Energy= 0.758698
Sum of electronic and zero-point Energies= -2945.066306
Sum of electronic and thermal Energies= -2945.010205
Sum of electronic and thermal Enthalpies= -2945.009260
Sum of electronic and thermal Free Energies= -2945.159722

Solvent: -2946.49978843

Ru	-1.180509000	-0.321486000	-0.609768000
Cl	-1.799762000	0.670430000	-2.745887000
Cl	-1.084043000	-1.400699000	1.569307000
N	-3.630187000	1.042284000	0.620235000
N	-1.895742000	2.406447000	0.591680000
C	-2.305109000	1.142115000	0.303731000
C	-4.205799000	2.347452000	1.025681000
H	-4.830769000	2.231826000	1.933941000
H	-4.853789000	2.745547000	0.213285000
C	-2.954758000	3.204608000	1.253645000
H	-3.026108000	4.212344000	0.798023000
H	-2.710335000	3.334702000	2.332089000
C	-4.494103000	-0.114094000	0.570590000
C	-5.247991000	-0.391626000	-0.601917000
C	-5.190913000	0.495830000	-1.822121000
H	-5.791567000	0.065755000	-2.647539000
H	-4.153760000	0.648325000	-2.189514000
H	-5.611393000	1.502315000	-1.603628000
C	-6.120283000	-1.501504000	-0.582540000
H	-6.699465000	-1.727560000	-1.493833000
C	-6.297258000	-2.300996000	0.562208000
C	-7.219257000	-3.499963000	0.547174000
H	-7.953528000	-3.443300000	-0.281346000
H	-7.779745000	-3.596275000	1.499880000
H	-6.645992000	-4.444131000	0.415204000
C	-5.592627000	-1.945394000	1.730193000
H	-5.748655000	-2.527535000	2.654125000
C	-4.703318000	-0.854109000	1.768494000
C	-4.022416000	-0.476005000	3.061135000
H	-4.340496000	-1.145952000	3.883681000
H	-4.265017000	0.565133000	3.366020000
H	-2.919051000	-0.549432000	2.952440000
C	-0.576716000	2.972828000	0.471463000
C	0.374118000	2.751151000	1.500761000
C	0.064406000	1.872287000	2.688656000
H	0.885506000	1.913653000	3.430504000
H	-0.089382000	0.809978000	2.395346000
H	-0.866504000	2.190586000	3.205353000
C	1.623818000	3.394678000	1.393345000
H	2.371640000	3.225086000	2.185693000
C	1.937003000	4.255654000	0.323752000
C	3.299081000	4.902007000	0.214212000
H	3.236713000	5.919076000	-0.224518000
H	3.962385000	4.301509000	-0.446401000
H	3.798451000	4.979801000	1.200937000
C	0.951287000	4.478949000	-0.657472000
H	1.168796000	5.160924000	-1.496485000
C	-0.310737000	3.856216000	-0.607373000
C	-1.345910000	4.130258000	-1.672864000
H	-2.231542000	4.665350000	-1.264042000
H	-1.713352000	3.185112000	-2.127918000
H	-0.925241000	4.766327000	-2.476339000
H	1.302688000	-1.833761000	0.901706000
C	1.660157000	-1.083922000	0.187394000
C	2.969949000	-0.752719000	-0.056166000
C	0.740007000	-0.316677000	-0.697932000
C	2.992004000	0.285556000	-1.117820000
C	1.649032000	0.544310000	-1.534398000
C	4.073562000	0.953973000	-1.700152000
C	1.409113000	1.473206000	-2.552870000
C	3.819557000	1.885859000	-2.734561000
H	5.102304000	0.750311000	-1.361962000
C	2.505281000	2.134711000	-3.157710000
H	0.378658000	1.668862000	-2.882342000
H	4.660795000	2.414025000	-3.212077000
H	2.318007000	2.856306000	-3.968695000
C	4.194174000	-1.346381000	0.558355000
C	4.606900000	-0.976550000	1.872321000
C	4.957216000	-2.288527000	-0.197876000
C	5.775267000	-1.561291000	2.405748000
C	6.117749000	-2.843770000	0.377870000
C	6.528448000	-2.484778000	1.669390000
H	6.102543000	-1.285911000	3.421457000
H	6.708616000	-3.578439000	-0.192568000
H	7.438080000	-2.929475000	2.104420000
C	-0.127911000	-1.872035000	-1.971643000
C	-1.424065000	-2.286958000	-1.524192000
H	-0.050206000	-1.370235000	-2.947949000
H	0.710679000	-2.517962000	-1.669772000
H	-2.311718000	-2.034935000	-2.146224000
O	-1.512550000	-3.438616000	-0.811749000
C	-2.787744000	-3.734799000	-0.239011000
H	-3.607661000	-3.551146000	-0.969908000
H	-2.771392000	-4.806080000	0.037271000
H	-2.946074000	-3.112278000	0.666346000

C	4.497859000	-2.763746000	-1.579563000
H	3.700081000	-2.070891000	-1.921045000
C	3.814960000	0.020560000	2.721447000
H	3.033137000	0.462661000	2.068716000
C	4.694650000	1.177591000	3.238144000
H	5.204834000	1.703782000	2.405088000
H	4.078000000	1.921018000	3.786953000
H	5.477251000	0.823284000	3.942508000
C	3.089675000	-0.694311000	3.882274000
H	2.385626000	-1.464045000	3.506224000
H	3.811241000	-1.197343000	4.561485000
H	2.505207000	0.029673000	4.489012000
C	5.616811000	-2.718400000	-2.638478000
H	6.436949000	-3.432532000	-2.411298000
H	5.215934000	-2.989307000	-3.637892000
H	6.062835000	-1.705405000	-2.714877000
C	3.867433000	-4.171339000	-1.487301000
H	3.478949000	-4.497093000	-2.475833000
H	4.613388000	-4.923589000	-1.152193000
H	3.026828000	-4.190320000	-0.763393000

Catalyst 5, IV intermediate

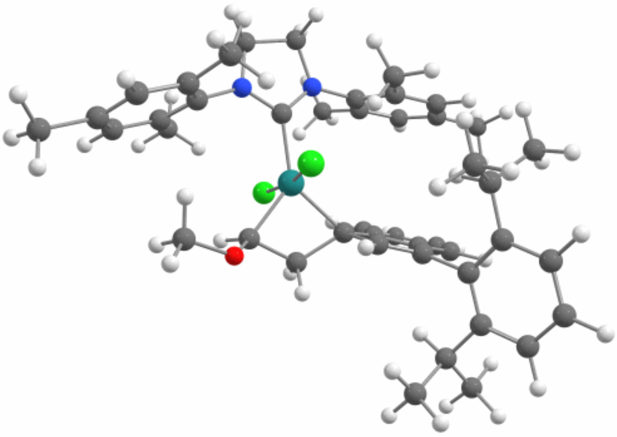

Zero-point correction= 0.853710 (Hartree/Particle)
Thermal correction to Energy= 0.909912
Thermal correction to Enthalpy= 0.910856
Thermal correction to Gibbs Free Energy= 0.761411
Sum of electronic and zero-point Energies= -2945.075372
Sum of electronic and thermal Energies= -2945.019170
Sum of electronic and thermal Enthalpies= -2945.018226
Sum of electronic and thermal Free Energies= -2945.167671

Solvent: -2946.50962304

Ru	-1.171934000	-0.244758000	-0.641673000
Cl	-1.819119000	0.808018000	-2.736303000
Cl	-0.901106000	-1.327470000	1.517403000
N	-3.650029000	0.953893000	0.656828000
N	-1.936061000	2.351518000	0.685313000
C	-2.332600000	1.099068000	0.340327000
C	-4.243495000	2.231654000	1.122733000
H	-4.858307000	2.063015000	2.029602000
H	-4.905239000	2.651303000	0.332875000
C	-3.006795000	3.103922000	1.379372000
H	-3.097763000	4.125055000	0.958025000
H	-2.762454000	3.200962000	2.460970000
C	-4.497985000	-0.212037000	0.571110000
C	-5.270413000	-0.441419000	-0.598582000
C	-5.219599000	0.488820000	-1.786393000
H	-5.813087000	0.082406000	-2.628722000
H	-4.182592000	0.665121000	-2.143595000
H	-5.651226000	1.482955000	-1.534906000
C	-6.147225000	-1.547562000	-0.607631000
H	-6.742749000	-1.736115000	-1.516834000
C	-6.306371000	-2.391490000	0.507241000
C	-7.240013000	-3.580759000	0.466310000
H	-7.834503000	-3.664164000	1.399767000
H	-6.673274000	-4.532035000	0.362100000
H	-7.945009000	-3.520654000	-0.386780000
C	-5.571523000	-2.091773000	1.672613000
H	-5.706241000	-2.715347000	2.572470000
C	-4.677048000	-1.005776000	1.738865000
C	-3.953315000	-0.696706000	3.026143000
H	-4.276036000	-1.382659000	3.833431000
H	-4.147106000	0.341935000	3.371092000
H	-2.855066000	-0.802002000	2.887082000
C	-0.621264000	2.933574000	0.562662000
C	0.328347000	2.725232000	1.597011000
C	0.014117000	1.873885000	2.803093000
H	0.835266000	1.928849000	3.543788000
H	-0.141059000	0.806324000	2.528813000
H	-0.914589000	2.209006000	3.313130000
C	1.580852000	3.361307000	1.479789000
H	2.328543000	3.197636000	2.272902000
C	1.896837000	4.205238000	0.398170000
C	3.262943000	4.839125000	0.274624000
H	3.204391000	5.860641000	-0.154370000
H	3.907141000	4.236438000	-0.402617000
H	3.778264000	4.901870000	1.254080000
C	0.906114000	4.431059000	-0.577681000
H	1.121000000	5.111127000	-1.418642000
C	-0.361250000	3.821490000	-0.515702000
C	-1.406874000	4.125598000	-1.560932000
H	-2.315420000	4.587236000	-1.115774000
H	-1.729707000	3.199880000	-2.084118000
H	-1.013451000	4.835347000	-2.314740000
H	1.374563000	-2.165134000	0.533136000
C	1.713219000	-1.318357000	-0.074737000
C	2.987364000	-0.819336000	-0.122471000
C	0.820001000	-0.631524000	-1.062814000
C	3.011929000	0.276379000	-1.119664000
C	1.718838000	0.390856000	-1.715520000
C	4.073884000	1.092124000	-1.535208000
C	1.506676000	1.320509000	-2.742083000
C	3.849912000	2.024752000	-2.571183000
H	5.067478000	0.989431000	-1.070022000
C	2.582702000	2.130980000	-3.170024000
H	0.509485000	1.423884000	-3.196104000
H	4.676432000	2.666522000	-2.917399000
H	2.419980000	2.857162000	-3.982382000
C	4.194558000	-1.339410000	0.590059000

C	4.429566000	-1.032242000	1.962373000
C	5.119098000	-2.158758000	-0.129509000
C	5.580675000	-1.555740000	2.589460000
C	6.256837000	-2.654194000	0.538926000
C	6.490495000	-2.357688000	1.889095000
H	5.767865000	-1.327551000	3.651294000
H	6.969381000	-3.293753000	-0.006155000
H	7.383890000	-2.755549000	2.397152000
C	0.004617000	-1.640665000	-1.992098000
C	-1.484333000	-1.974850000	-1.590001000
H	0.021871000	-1.275840000	-3.035145000
H	0.516374000	-2.614809000	-1.872342000
H	-2.203634000	-1.841804000	-2.431950000
O	-1.582913000	-3.197552000	-0.982665000
C	-2.910481000	-3.645748000	-0.723848000
H	-3.552063000	-3.546494000	-1.629945000
H	-2.838875000	-4.712440000	-0.438658000
H	-3.364496000	-3.068615000	0.109502000
C	3.464174000	-0.169775000	2.777798000
H	2.724091000	0.259314000	2.069663000
C	4.860680000	-2.574028000	-1.581513000
H	4.057737000	-1.918246000	-1.977213000
C	4.333858000	-4.025309000	-1.643868000
H	3.418350000	-4.147680000	-1.029117000
H	4.089335000	-4.314178000	-2.688586000
H	5.090575000	-4.743887000	-1.261953000
C	6.088512000	-2.385120000	-2.493909000
H	6.465049000	-1.342240000	-2.456978000
H	6.926649000	-3.057794000	-2.212513000
H	5.826430000	-2.614552000	-3.548339000
C	4.179178000	1.007605000	3.472313000
H	4.914142000	0.662513000	4.230871000
H	4.723887000	1.640979000	2.741932000
H	3.445451000	1.649946000	4.004531000
C	2.679966000	-1.026880000	3.795051000
H	1.964415000	-0.401605000	4.369566000
H	2.095116000	-1.820238000	3.287845000
H	3.363458000	-1.515106000	4.522907000

Catalyst 5, IV-V TS

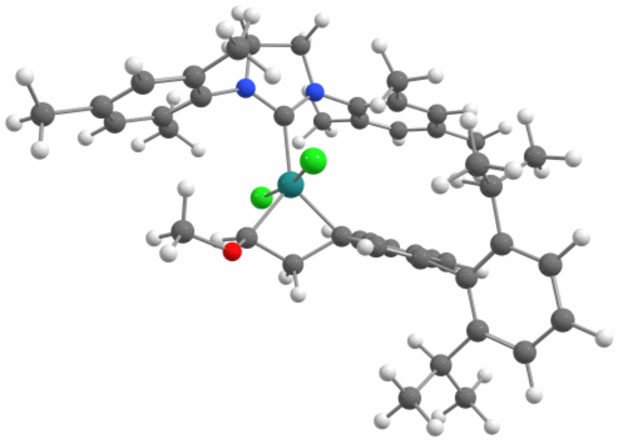

Zero-point correction= 0.852520 (Hartree/Particle)
Thermal correction to Energy= 0.908468
Thermal correction to Enthalpy= 0.909412
Thermal correction to Gibbs Free Energy= 0.759054
Sum of electronic and zero-point Energies= -2945.073067
Sum of electronic and thermal Energies= -2945.017119
Sum of electronic and thermal Enthalpies= -2945.016175
Sum of electronic and thermal Free Energies= -2945.166533

Solvent: -2946.50352083

Ru	-1.142884000	-0.289280000	-0.484122000
Cl	-1.954048000	0.352550000	-2.685854000
Cl	-0.509029000	-0.807690000	1.801101000
N	-3.662825000	0.930765000	0.728641000
N	-2.013152000	2.399998000	0.692222000
C	-2.360185000	1.124044000	0.384236000
C	-4.278492000	2.143106000	1.320477000
H	-4.650172000	1.919745000	2.342820000
H	-5.148172000	2.468954000	0.710822000
C	-3.123377000	3.161077000	1.311072000
H	-3.347118000	4.066370000	0.707938000
H	-2.836020000	3.498970000	2.329407000
C	-4.472663000	-0.255321000	0.578629000
C	-5.277938000	-0.398879000	-0.584086000
C	-5.287671000	0.638657000	-1.680256000
H	-6.030434000	0.378821000	-2.459650000
H	-4.289201000	0.723956000	-2.162181000
H	-5.551374000	1.646108000	-1.292058000
C	-6.096701000	-1.542323000	-0.687894000
H	-6.714024000	-1.665059000	-1.593925000
C	-6.167282000	-2.511074000	0.332636000
C	-7.090790000	-3.703835000	0.210990000
H	-6.726320000	-4.567906000	0.802866000
H	-7.205688000	-4.029081000	-0.843157000
H	-8.108976000	-3.459195000	0.587007000
C	-5.399269000	-2.303217000	1.494656000
H	-5.461252000	-3.031143000	2.321189000
C	-4.560321000	-1.180705000	1.653508000
C	-3.801655000	-0.979259000	2.940930000
H	-4.053293000	-1.770677000	3.673671000
H	-4.043655000	-0.001140000	3.410273000
H	-2.702461000	-0.991400000	2.767542000
C	-0.760565000	3.067380000	0.426226000
C	0.212621000	3.151345000	1.457380000
C	-0.012053000	2.525555000	2.812168000
H	0.840589000	2.735279000	3.486946000
H	-0.137753000	1.423252000	2.727841000
H	-0.927235000	2.922236000	3.303051000
C	1.401753000	3.858765000	1.188497000
H	2.168494000	3.916211000	1.978463000
C	1.633208000	4.497533000	-0.044558000
C	2.937015000	5.208905000	-0.324925000
H	2.782544000	6.120224000	-0.938635000
H	3.626921000	4.547077000	-0.893269000
H	3.455677000	5.502637000	0.609771000
C	0.617200000	4.445395000	-1.018541000
H	0.761608000	4.969499000	-1.977999000
C	-0.591145000	3.753476000	-0.807685000
C	-1.669239000	3.773585000	-1.864327000
H	-2.646942000	4.104829000	-1.453080000
H	-1.829553000	2.763872000	-2.302174000
H	-1.399971000	4.468275000	-2.683887000
H	1.533646000	-2.246244000	0.704173000
C	1.802045000	-1.459458000	-0.010301000
C	3.043745000	-0.899383000	-0.156139000

C	0.852585000	-0.939717000	-1.036069000
C	2.974624000	0.077662000	-1.267416000
C	1.660199000	0.051378000	-1.829550000
C	3.970392000	0.906629000	-1.803634000
C	1.363064000	0.845396000	-2.945277000
C	3.659071000	1.707379000	-2.923732000
H	4.980456000	0.915968000	-1.363463000
C	2.373244000	1.669319000	-3.490730000
H	0.349860000	0.837750000	-3.374044000
H	4.432741000	2.359229000	-3.361142000
H	2.143729000	2.291765000	-4.370246000
C	4.291012000	-1.255408000	0.586185000
C	4.515220000	-0.778081000	1.910820000
C	5.262315000	-2.088865000	-0.051106000
C	5.704991000	-1.148521000	2.574145000
C	6.435224000	-2.430289000	0.651937000
C	6.659530000	-1.965402000	1.955818000
H	5.886204000	-0.786269000	3.599151000
H	7.185652000	-3.079675000	0.173330000
H	7.581897000	-2.243226000	2.491315000
C	-0.109907000	-1.923688000	-1.634957000
C	-1.758459000	-2.042601000	-0.902476000
H	-0.287756000	-1.791369000	-2.717205000
H	0.163550000	-2.954711000	-1.347340000
H	-2.420481000	-2.156094000	-1.794306000
O	-1.882595000	-3.051646000	-0.007903000
C	-2.638504000	-4.189515000	-0.443158000
H	-2.113486000	-4.730887000	-1.262227000
H	-2.731535000	-4.859355000	0.431935000
H	-3.652867000	-3.888674000	-0.783122000
C	5.014961000	-2.680670000	-1.442350000
H	4.198692000	-2.093341000	-1.912146000
C	3.509917000	0.119348000	2.636035000
H	2.684527000	0.340549000	1.928029000
C	4.143492000	1.462616000	3.054968000
H	3.387422000	2.114611000	3.541755000
H	4.971281000	1.321706000	3.782829000
H	4.554473000	2.008376000	2.180661000
C	2.881132000	-0.604114000	3.845862000
H	3.646657000	-0.866661000	4.607861000
H	2.122437000	0.041701000	4.335370000
H	2.372153000	-1.538460000	3.535043000
C	6.237268000	-2.580430000	-2.375331000
H	7.086070000	-3.204905000	-2.023446000
H	5.974876000	-2.934056000	-3.394738000
H	6.599066000	-1.535124000	-2.460506000
C	4.517646000	-4.139259000	-1.328158000
H	4.283659000	-4.558530000	-2.330224000
H	5.287649000	-4.788768000	-0.858991000
H	3.601470000	-4.203736000	-0.705655000

Catalyst 5, V intermediate

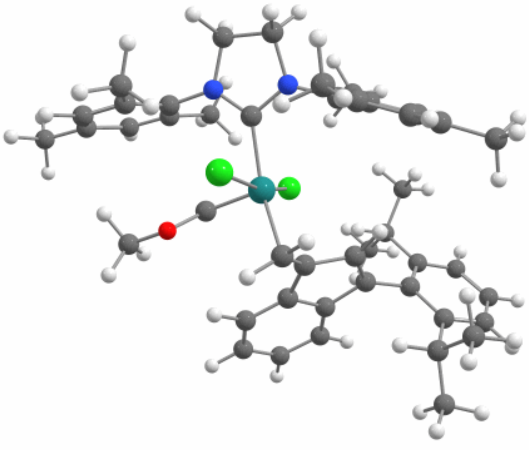

Zero-point correction= 0.851711 (Hartree/Particle)
Thermal correction to Energy= 0.909025
Thermal correction to Enthalpy= 0.909969
Thermal correction to Gibbs Free Energy= 0.755053
Sum of electronic and zero-point Energies= -2945.090226
Sum of electronic and thermal Energies= -2945.032912

Ru	1.019847000	0.018609000	-0.734240000
Cl	0.168573000	-0.543506000	1.472192000
Cl	2.317597000	0.780265000	-2.677267000
N	3.626917000	1.198841000	0.630016000
N	1.963823000	2.623852000	0.369016000
C	2.342956000	1.325793000	0.204772000
C	4.164222000	2.466588000	1.185488000
H	5.148139000	2.701661000	0.732066000
H	4.311927000	2.362072000	2.283198000
C	3.075296000	3.489573000	0.822466000
H	2.758042000	4.115021000	1.681894000
H	3.385459000	4.170490000	0.000078000
C	4.457694000	0.022968000	0.704414000
C	4.370791000	-0.818229000	1.848883000
C	3.384230000	-0.545208000	2.957218000
H	3.524958000	-1.257512000	3.793505000
H	2.331401000	-0.621132000	2.602965000
H	3.505048000	0.480675000	3.367404000
C	5.255109000	-1.911793000	1.938252000
H	5.187757000	-2.568324000	2.822295000
C	6.232565000	-2.171185000	0.955614000
C	7.171529000	-3.349642000	1.093335000
H	7.914007000	-3.380530000	0.271431000
H	6.617597000	-4.313216000	1.086252000
H	7.728415000	-3.311280000	2.053711000
C	6.315363000	-1.292160000	-0.141026000
H	7.087479000	-1.460185000	-0.910703000
C	5.453458000	-0.183172000	-0.286499000
C	5.627208000	0.763600000	-1.449004000
H	6.337067000	0.349434000	-2.191638000
H	6.040804000	1.741675000	-1.115593000
H	4.659175000	0.962644000	-1.956385000
C	0.646370000	3.174164000	0.144491000
C	0.330325000	3.808999000	-1.089457000
C	1.357659000	4.026614000	-2.173636000
H	0.870761000	4.342180000	-3.117384000
H	1.949120000	3.110598000	-2.380918000
H	2.061913000	4.839555000	-1.886457000
C	-0.974224000	4.323013000	-1.251833000
H	-1.233113000	4.795698000	-2.214173000
C	-1.934964000	4.283135000	-0.224139000
C	-3.336384000	4.808592000	-0.437478000
H	-3.700169000	5.371819000	0.446631000

Sum of electronic and thermal Enthalpies= -2945.031968
Sum of electronic and thermal Free Energies= -2945.186884

Solvent: -2946.51990736

H	-4.051350000	3.972580000	-0.600417000
H	-3.396427000	5.473859000	-1.321899000
C	-1.546788000	3.748502000	1.021085000
H	-2.260329000	3.764797000	1.861708000
C	-0.266279000	3.205671000	1.238239000
C	0.120991000	2.703995000	2.607048000
H	1.102701000	3.106958000	2.934100000
H	0.200182000	1.594731000	2.605951000
H	-0.634829000	2.999275000	3.360198000
H	-2.315446000	0.943474000	-0.913570000
C	-2.199937000	-0.145527000	-0.816629000
C	-3.082514000	-0.995537000	-0.188155000
C	-1.086915000	-0.907794000	-1.415400000
C	-2.561858000	-2.373659000	-0.320129000
C	-1.343620000	-2.331185000	-1.060968000
C	-3.103401000	-3.603083000	0.093899000
C	-0.680001000	-3.518300000	-1.397624000
C	-2.415480000	-4.789919000	-0.225111000
H	-4.055941000	-3.634559000	0.646604000
C	-1.217715000	-4.749139000	-0.967301000
H	0.243505000	-3.495283000	-1.997622000
H	-2.823929000	-5.761310000	0.097074000
H	-0.703056000	-5.689077000	-1.224012000
C	-4.409802000	-0.660908000	0.415500000
C	-4.552489000	-0.523562000	1.828644000
C	-5.548015000	-0.519342000	-0.436799000
C	-5.826258000	-0.228323000	2.358997000
C	-6.798679000	-0.214666000	0.140254000
C	-6.942322000	-0.068783000	1.526752000
H	-5.944157000	-0.118191000	3.449455000
H	-7.679946000	-0.095743000	-0.511138000
H	-7.928318000	0.165734000	1.960034000
C	-0.262507000	-0.441869000	-2.462810000
C	1.985243000	-1.541215000	-0.638284000
H	0.258165000	-1.152202000	-3.123938000
H	-0.477649000	0.537912000	-2.921801000
H	2.010736000	-2.115430000	0.322479000
O	2.617302000	-2.142708000	-1.638668000
C	3.327809000	-3.368385000	-1.361485000
H	2.827299000	-3.940724000	-0.552469000
H	3.328532000	-3.956178000	-2.298524000
H	4.369040000	-3.126682000	-1.066873000
C	-5.450881000	-0.689856000	-1.956080000
H	-4.426924000	-1.051978000	-2.182835000
C	-3.363184000	-0.682831000	2.777144000
H	-2.453527000	-0.821944000	2.156339000
C	-3.510371000	-1.927619000	3.677606000
H	-3.619729000	-2.852621000	3.075645000
H	-2.613580000	-2.050181000	4.321328000
H	-4.396399000	-1.850658000	4.344932000
C	-3.132216000	0.585957000	3.623174000
H	-3.978722000	0.787303000	4.315064000
H	-2.215018000	0.477821000	4.239330000
H	-3.004000000	1.477982000	2.976530000
C	-5.627470000	0.658967000	-2.686062000
H	-5.512422000	0.533576000	-3.784029000
H	-6.633593000	1.093261000	-2.500514000
H	-4.874906000	1.400765000	-2.347407000
C	-6.437699000	-1.748954000	-2.489823000
H	-6.295228000	-2.724452000	-1.981194000
H	-7.496010000	-1.443531000	-2.344102000
H	-6.287358000	-1.907247000	-3.578897000

Catalyst 5, V-VI TS

Ru	1.704948000	-0.311091000	0.087863000
Cl	1.022230000	-0.706180000	-2.122830000
Cl	1.769564000	-0.790344000	2.404196000
N	4.353589000	1.148231000	-0.202850000
N	4.459582000	-1.057732000	-0.356518000
C	3.588614000	0.002514000	-0.174523000
C	5.768483000	0.879707000	-0.529388000
H	6.441541000	1.445037000	0.147699000
H	5.994206000	1.199383000	-1.572989000
C	5.872523000	-0.641860000	-0.349964000
H	6.435014000	-1.138217000	-1.167614000
H	6.357713000	-0.919319000	0.613617000
C	3.919285000	2.511196000	-0.115095000
C	3.539713000	3.204402000	-1.294245000
C	3.484838000	2.499221000	-2.629677000
H	3.162907000	3.192263000	-3.431188000
H	2.775882000	1.643208000	-2.604691000
H	4.473581000	2.086242000	-2.923455000
C	3.165542000	4.558005000	-1.175344000
H	2.862348000	5.103929000	-2.084819000
C	3.163465000	5.228805000	0.065637000
C	2.800953000	6.694946000	0.153059000
H	2.414074000	6.963953000	1.156628000
H	2.036334000	6.974581000	-0.600055000
H	3.690988000	7.335113000	-0.036309000
C	3.538126000	4.503695000	1.215601000
H	3.531607000	5.006689000	2.197330000
C	3.922531000	3.148766000	1.152102000
C	4.281865000	2.381602000	2.403089000

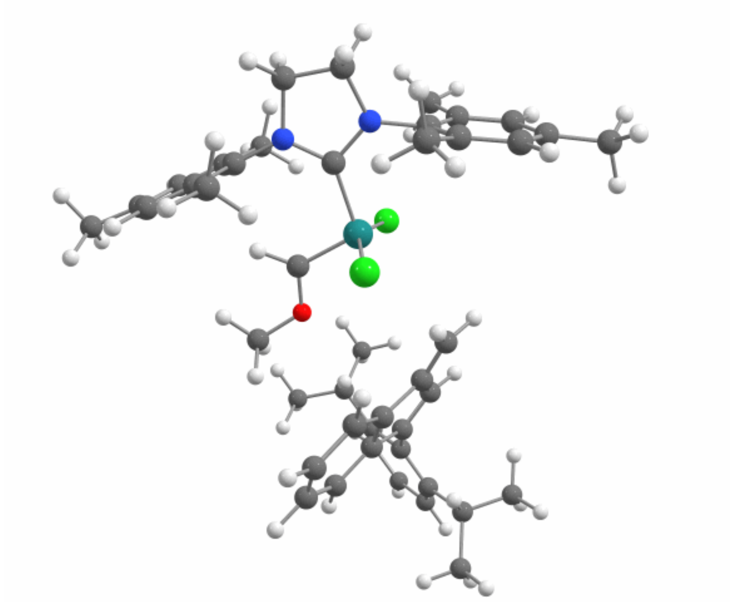

Zero-point correction= 0.850354 (Hartree/Particle)
Thermal correction to Energy= 0.908282
Thermal correction to Enthalpy= 0.909226
Thermal correction to Gibbs Free Energy= 0.746686
Sum of electronic and zero-point Energies= -2945.088085
Sum of electronic and thermal Energies= -2945.030157
Sum of electronic and thermal Enthalpies= -2945.029212
Sum of electronic and thermal Free Energies= -2945.191752

Solvent: -2946.50673672

H	4.259061000	3.039508000	3.293713000
H	5.297772000	1.937046000	2.336960000
H	3.577029000	1.538404000	2.573036000
C	4.114634000	-2.456516000	-0.378250000
C	4.156951000	-3.219737000	0.821370000
C	4.625494000	-2.630087000	2.130937000
H	4.253135000	-3.225428000	2.987392000
H	4.270793000	-1.591680000	2.270967000
H	5.737660000	-2.637078000	2.186777000
C	3.825978000	-4.588054000	0.750902000
H	3.838397000	-5.179796000	1.681744000
C	3.500820000	-5.220895000	-0.464016000
C	3.104152000	-6.679571000	-0.502717000
H	3.430677000	-7.168591000	-1.443231000
H	1.998610000	-6.789878000	-0.446538000
H	3.532101000	-7.244960000	0.349709000
C	3.554729000	-4.453697000	-1.644705000
H	3.355271000	-4.939911000	-2.614480000
C	3.871215000	-3.081551000	-1.634122000
C	3.974512000	-2.317397000	-2.931520000
H	4.885588000	-1.684414000	-2.967081000
H	3.099913000	-1.643190000	-3.058617000
H	4.004678000	-3.011758000	-3.794124000
H	-3.543082000	-2.036750000	-1.106979000
C	-3.516198000	-1.383939000	-0.223306000
C	-4.476917000	-0.461607000	0.124377000
C	-2.422442000	-1.382576000	0.761624000
C	-4.042801000	0.202253000	1.377740000
C	-2.787049000	-0.353135000	1.768752000
C	-4.647151000	1.213033000	2.142667000
C	-2.130431000	0.107029000	2.916886000
C	-3.985124000	1.667304000	3.303102000
H	-5.617444000	1.640315000	1.842162000
C	-2.741534000	1.123753000	3.684218000
H	-1.147398000	-0.298567000	3.205786000
H	-4.446185000	2.456488000	3.919086000
H	-2.240221000	1.493103000	4.593321000
C	-5.744056000	-0.147626000	-0.602202000
C	-5.745595000	0.827636000	-1.642220000
C	-6.950519000	-0.817178000	-0.239369000
C	-6.953398000	1.096224000	-2.320261000
C	-8.133616000	-0.516240000	-0.945297000
C	-8.138794000	0.430496000	-1.979809000
H	-6.964933000	1.841755000	-3.132035000
H	-9.069988000	-1.033526000	-0.681090000
H	-9.072749000	0.652150000	-2.521350000
C	-1.316421000	-2.175072000	0.745721000
C	1.052591000	1.395780000	0.226979000
H	-0.547336000	-2.112708000	1.533044000
H	-1.157420000	-2.904372000	-0.064204000
H	1.620568000	2.353370000	0.257619000
O	-0.267921000	1.550741000	0.336254000
C	-0.830715000	2.851134000	0.593574000
H	-1.562974000	3.073651000	-0.206840000
H	-1.353437000	2.812725000	1.570101000
H	-0.034473000	3.625548000	0.609831000
C	-6.964861000	-1.881072000	0.861621000
H	-6.016426000	-1.775573000	1.429818000
C	-4.467447000	1.564911000	-2.050798000
H	-3.684758000	1.297708000	-1.309833000
C	-3.964257000	1.092142000	-3.431586000
H	-4.700165000	1.324384000	-4.231414000
H	-3.007180000	1.590250000	-3.695272000
H	-3.788818000	-0.002820000	-3.442919000
C	-4.636474000	3.097832000	-2.010666000
H	-5.379627000	3.454353000	-2.755679000
H	-4.971577000	3.443364000	-1.010838000
H	-3.674512000	3.602218000	-2.245032000
C	-6.974797000	-3.300376000	0.252342000
H	-6.102695000	-3.458034000	-0.414768000
H	-6.940732000	-4.073925000	1.049001000
H	-7.893568000	-3.471442000	-0.348998000
C	-8.120513000	-1.696187000	1.864857000
H	-8.115113000	-0.679282000	2.308548000
H	-9.113787000	-1.854039000	1.392859000
H	-8.033315000	-2.430060000	2.693558000

Catalyst 6, I intermediate

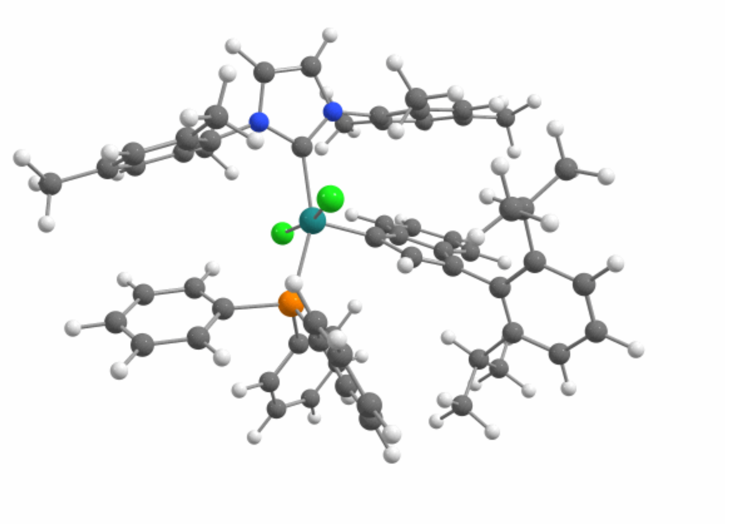

Zero-point correction= 1.013739 (Hartree/Particle)
Thermal correction to Energy= 1.081939
Thermal correction to Enthalpy= 1.082883
Thermal correction to Gibbs Free Energy= 0.905987
Sum of electronic and zero-point Energies= -3786.476780
Sum of electronic and thermal Energies= -3786.408580
Sum of electronic and thermal Enthalpies= -3786.407636
Sum of electronic and thermal Free Energies= -3786.584532

Solvent: -3788.22109082

Ru	0.982909000	0.380041000	0.061092000
Cl	1.720186000	0.418535000	2.363340000
Cl	0.738880000	0.392841000	-2.355100000
P	1.199863000	-2.033382000	0.097378000
N	2.913916000	2.746778000	-0.518946000
N	0.919882000	3.566986000	-0.253393000
C	1.616069000	2.376945000	-0.199353000
C	3.003730000	4.117940000	-0.770931000
H	3.955371000	4.587289000	-1.038482000
C	1.753053000	4.633440000	-0.602880000
H	1.367248000	5.653752000	-0.689547000
C	4.125525000	1.946223000	-0.548042000
C	4.905950000	1.867629000	0.634813000
C	4.467093000	2.516918000	1.923649000
H	5.268415000	2.456656000	2.685735000
H	3.558470000	2.017732000	2.326814000
H	4.214062000	3.588344000	1.774909000
C	6.145785000	1.200179000	0.558744000
H	6.760875000	1.133160000	1.471766000
C	6.632928000	0.654072000	-0.643506000
C	7.961996000	-0.063900000	-0.698970000
H	7.812635000	-1.161483000	-0.795246000
H	8.564392000	0.113579000	0.214421000
H	8.562370000	0.257022000	-1.575754000
C	5.852725000	0.808772000	-1.806212000
H	6.234368000	0.428815000	-2.768648000
C	4.608701000	1.469897000	-1.796183000
C	3.870513000	1.722125000	-3.087065000
H	4.407522000	1.264415000	-3.940673000
H	3.778617000	2.811291000	-3.290643000
H	2.837153000	1.314192000	-3.053923000
C	-0.456758000	3.837631000	0.098146000
C	-1.441696000	3.849659000	-0.917549000
C	-1.101402000	3.509921000	-2.347631000
H	-1.998681000	3.579660000	-2.992539000
H	-0.671230000	2.488372000	-2.439869000
H	-0.338619000	4.207778000	-2.756721000
C	-2.747237000	4.235279000	-0.550550000
H	-3.529765000	4.244996000	-1.326693000
C	-3.075005000	4.622927000	0.763567000
C	-4.491856000	4.994016000	1.137640000
H	-4.517484000	5.819586000	1.878434000
H	-5.012642000	4.126695000	1.599695000
H	-5.084013000	5.302912000	0.252871000
C	-2.049553000	4.637023000	1.729906000
H	-2.281397000	4.954305000	2.760014000
C	-0.730140000	4.255949000	1.422934000
C	0.362854000	4.325261000	2.464681000
H	1.083684000	5.142148000	2.241119000
H	0.950074000	3.384207000	2.511698000
H	-0.060883000	4.521052000	3.468895000
H	-1.537683000	-0.795182000	-1.530600000
C	-1.839606000	-0.328982000	-0.586013000
C	-3.129956000	-0.254162000	-0.102474000
C	-0.878106000	0.298600000	0.347739000
C	-3.081537000	0.450857000	1.203359000
C	-1.717162000	0.763378000	1.505376000
C	-4.114755000	0.793066000	2.081808000
C	-1.406780000	1.358804000	2.733682000
C	-3.787772000	1.405271000	3.315482000
H	-5.162583000	0.577212000	1.818615000
C	-2.448592000	1.665113000	3.642571000
H	-0.357839000	1.557180000	2.992687000
H	-4.589428000	1.666216000	4.025343000
H	-2.200011000	2.120723000	4.614414000
C	-4.383337000	-0.777734000	-0.719943000
C	-4.908071000	-0.191431000	-1.914161000
C	-5.074875000	-1.865151000	-0.097393000
C	-6.107908000	-0.702220000	-2.452490000
C	-6.274102000	-2.332818000	-0.671857000
C	-6.792699000	-1.759317000	-1.840009000
H	-6.518343000	-0.257250000	-3.373195000
H	-6.806211000	-3.175625000	-0.201460000
H	-7.729834000	-2.140298000	-2.277204000
C	2.997541000	-2.504976000	-0.059949000
C	3.455726000	-3.477895000	-0.976409000
C	3.915394000	-1.928886000	0.848961000
C	4.804448000	-3.876189000	-0.974568000
H	2.756749000	-3.944251000	-1.685927000
C	5.258237000	-2.340617000	0.852541000
H	3.566856000	-1.168898000	1.568287000
C	5.707246000	-3.316558000	-0.054757000
H	5.144873000	-4.640813000	-1.691254000
H	5.957437000	-1.892020000	1.575440000
H	6.759121000	-3.645085000	-0.042340000
C	0.374337000	-3.070122000	-1.205454000
C	-0.528430000	-4.102042000	-0.862823000
C	0.651621000	-2.832374000	-2.574078000
C	-1.137816000	-4.879368000	-1.863954000
H	-0.755729000	-4.308268000	0.193177000
C	0.040648000	-3.614684000	-3.568984000
H	1.330831000	-2.017157000	-2.860245000
C	-0.856408000	-4.638763000	-3.219216000

H	-1.835679000	-5.682092000	-1.577214000
H	0.267435000	-3.414569000	-4.628435000
H	-1.335101000	-5.249062000	-4.001697000
C	0.738499000	-2.919396000	1.668490000
C	1.393166000	-4.116373000	2.038841000
C	-0.295116000	-2.420944000	2.488179000
C	1.012451000	-4.804480000	3.203130000
H	2.208522000	-4.515508000	1.415861000
C	-0.675184000	-3.113469000	3.651603000
H	-0.796196000	-1.477454000	2.228124000
C	-0.023086000	-4.304774000	4.012664000
H	1.533109000	-5.735123000	3.480448000
H	-1.477920000	-2.705169000	4.285607000
H	-0.316337000	-4.840820000	4.929623000
C	-4.512267000	-2.603030000	1.122771000
H	-3.599765000	-2.064382000	1.452896000
C	-4.212751000	0.967056000	-2.634002000
H	-3.411494000	1.342210000	-1.963997000
C	-3.535326000	0.485266000	-3.935649000
H	-3.000861000	1.321029000	-4.434805000
H	-2.794962000	-0.315267000	-3.736321000
H	-4.283392000	0.084219000	-4.653120000
C	-5.170404000	2.144526000	-2.911918000
H	-5.972971000	1.871446000	-3.629762000
H	-5.658978000	2.501277000	-1.981786000
H	-4.616837000	2.997633000	-3.358943000
C	-5.492809000	-2.631891000	2.312605000
H	-6.421851000	-3.189326000	2.066545000
H	-5.028359000	-3.136178000	3.186259000
H	-5.786432000	-1.610558000	2.628638000
C	-4.075549000	-4.033787000	0.738272000
H	-3.585214000	-4.538360000	1.597732000
H	-4.945446000	-4.654158000	0.433128000
H	-3.359741000	-4.018061000	-0.107624000

Catalyst 6, I-II TS

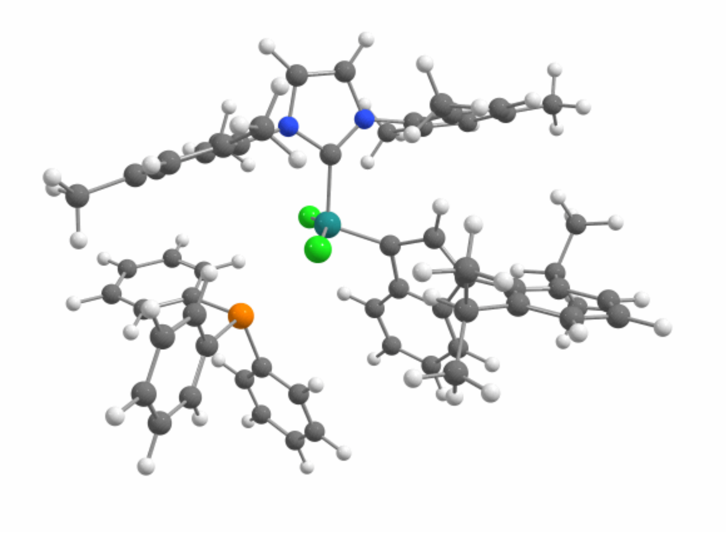

Zero-point correction= 1.012322 (Hartree/Particle)
Thermal correction to Energy= 1.080603
Thermal correction to Enthalpy= 1.081548
Thermal correction to Gibbs Free Energy= 0.900900
Sum of electronic and zero-point Energies= -3786.453284
Sum of electronic and thermal Energies= -3786.385003
Sum of electronic and thermal Enthalpies= -3786.384058
Sum of electronic and thermal Free Energies= -3786.564705

Solvent: -3788.18444743

Ru	0.251562000	-0.743614000	-0.330153000
Cl	0.873411000	-1.055953000	-2.583175000
Cl	0.468407000	-0.174375000	1.949319000
P	3.100935000	1.396522000	-0.377295000
N	0.811376000	-3.538639000	0.365503000
N	-1.251772000	-3.470381000	-0.357548000
C	-0.172535000	-2.639769000	-0.063076000
C	0.354704000	-4.855315000	0.319854000
H	0.996199000	-5.692455000	0.612070000
C	-0.931359000	-4.814183000	-0.125345000
H	-1.662589000	-5.607917000	-0.305324000
C	2.144895000	-3.243250000	0.850256000
C	3.233681000	-3.260891000	-0.059423000
C	3.055539000	-3.614836000	-1.516234000
H	4.033623000	-3.628027000	-2.034344000
H	2.394453000	-2.896705000	-2.045402000
H	2.594771000	-4.620027000	-1.629906000
C	4.522075000	-3.008041000	0.457077000
H	5.372701000	-2.991652000	-0.243702000
C	4.753642000	-2.802931000	1.829960000
C	6.137871000	-2.492038000	2.350771000
H	6.314633000	-2.949626000	3.345920000
H	6.271533000	-1.394054000	2.468126000
H	6.927062000	-2.847187000	1.658255000
C	3.655098000	-2.895331000	2.709385000
H	3.821986000	-2.797068000	3.795232000
C	2.343818000	-3.125199000	2.251769000
C	1.205162000	-3.271372000	3.231777000
H	1.584315000	-3.292850000	4.272102000
H	0.627219000	-4.203318000	3.057415000
H	0.503018000	-2.416076000	3.135956000
C	-2.613783000	-3.177914000	-0.752199000
C	-3.602639000	-3.165129000	0.262299000
C	-3.230849000	-3.271490000	1.725179000
H	-4.079221000	-2.973489000	2.371811000
H	-2.355705000	-2.640285000	1.983364000
H	-2.956655000	-4.314622000	1.997109000
C	-4.955167000	-3.108658000	-0.130500000
H	-5.732936000	-3.102357000	0.651341000
C	-5.336117000	-3.090997000	-1.486546000
C	-6.792362000	-3.134223000	-1.893232000
H	-7.082171000	-4.161991000	-2.205194000
H	-6.997515000	-2.466260000	-2.754399000
H	-7.462969000	-2.840071000	-1.061770000
C	-4.319215000	-3.092108000	-2.462576000
H	-4.596114000	-3.079309000	-3.530178000
C	-2.951796000	-3.151079000	-2.128366000
C	-1.899833000	-3.249298000	-3.203994000
H	-1.337062000	-4.205256000	-3.128931000
H	-1.146226000	-2.438187000	-3.121634000
H	-2.359548000	-3.204887000	-4.210420000
H	-2.849580000	-0.723646000	0.992845000
C	-2.593466000	0.091756000	0.309879000
C	-3.268889000	1.298588000	0.239831000
C	-1.386794000	0.131024000	-0.515631000
C	-2.572649000	2.155824000	-0.752342000
C	-1.437481000	1.438855000	-1.245732000
C	-2.860985000	3.443679000	-1.213233000
C	-0.630840000	2.002490000	-2.242094000

	C	-2.038552000	4.008298000	-2.218996000
	H	-3.717038000	4.004927000	-0.806151000
	C	-0.948371000	3.292308000	-2.734345000
	H	0.229882000	1.445060000	-2.639905000
	H	-2.261689000	5.017770000	-2.600118000
	H	-0.317650000	3.740224000	-3.517481000
	C	-4.418595000	1.751714000	1.073550000
	C	-5.689819000	1.993623000	0.464340000
	C	-4.240176000	1.980416000	2.475198000
	C	-6.760022000	2.435020000	1.268365000
	C	-5.344889000	2.423180000	3.233383000
	C	-6.595475000	2.647071000	2.643936000
	H	-7.746325000	2.609492000	0.808815000
	H	-5.215733000	2.605600000	4.312602000
	H	-7.443982000	2.991663000	3.257046000
	C	3.839928000	1.874450000	1.263023000
	C	4.458696000	3.123022000	1.505086000
	C	3.782028000	0.926681000	2.310521000
	C	5.014183000	3.412415000	2.763738000
	H	4.507599000	3.876506000	0.703765000
	C	4.345473000	1.217362000	3.565704000
	H	3.271374000	-0.035179000	2.149619000
	C	4.961877000	2.459664000	3.796762000
	H	5.492036000	4.390388000	2.936757000
	H	4.285588000	0.470671000	4.373867000
	H	5.393980000	2.689505000	4.784104000
	C	2.898642000	3.064623000	-1.174165000
	C	3.658167000	3.510756000	-2.278233000
	C	1.900682000	3.917611000	-0.643843000
	C	3.433516000	4.785569000	-2.829959000
	H	4.438236000	2.862066000	-2.705513000
	C	1.692222000	5.196345000	-1.184670000
	H	1.284879000	3.577837000	0.205332000
	C	2.457029000	5.634664000	-2.281623000
	H	4.036459000	5.119228000	-3.690109000
	H	0.917596000	5.849659000	-0.752771000
	H	2.288978000	6.635849000	-2.709958000
	C	4.577571000	0.704267000	-1.278065000
	C	5.900113000	0.786022000	-0.786117000
	C	4.347281000	0.044069000	-2.508809000
	C	6.967986000	0.229313000	-1.512690000
	H	6.098039000	1.292567000	0.171007000
	C	5.419210000	-0.499228000	-3.238324000
	H	3.317253000	-0.058539000	-2.887629000
	C	6.732491000	-0.410951000	-2.742219000
	H	7.993490000	0.303060000	-1.115460000
	H	5.222788000	-1.004053000	-4.198070000
	H	7.571224000	-0.842665000	-3.311897000
	C	-2.888415000	1.821990000	3.177157000
	H	-2.153205000	1.453089000	2.435234000
	C	-5.942848000	1.714552000	-1.020621000
	H	-4.960232000	1.519837000	-1.498392000
	C	-2.941305000	0.775447000	4.309172000
	H	-3.301253000	-0.205785000	3.936467000
	H	-1.929672000	0.622736000	4.739776000
	H	-3.616290000	1.089176000	5.134810000
	C	-2.363035000	3.178799000	3.695240000
	H	-1.350051000	3.058935000	4.133820000
	H	-2.292548000	3.922863000	2.875176000
	H	-3.023178000	3.605004000	4.481275000
	C	-6.789804000	0.437218000	-1.198423000
	H	-6.936129000	0.210513000	-2.275897000
	H	-6.294375000	-0.436079000	-0.729319000
	H	-7.794518000	0.550717000	-0.737279000
	C	-6.585796000	2.907982000	-1.755620000
	H	-6.672921000	2.692970000	-2.841528000
	H	-7.609220000	3.123196000	-1.380854000
	H	-5.985798000	3.833790000	-1.641237000

**Catalyst 6, II intermediate**	Ru	-1.493813000	-0.706781000	-0.360586000
	Cl	-1.932980000	-0.746721000	-2.650407000
	Cl	-1.930862000	-1.605368000	1.734012000
	N	-3.481043000	1.421886000	0.016820000
	N	-1.526026000	2.382924000	-0.184880000
	C	-2.119007000	1.128815000	-0.124455000
	C	-3.700642000	2.799689000	0.030567000
	H	-4.708195000	3.214414000	0.133415000
	C	-2.484044000	3.400733000	-0.093404000
	H	-2.193160000	4.455166000	-0.123733000
	C	-4.571827000	0.489345000	0.222323000
	C	-5.355330000	0.074622000	-0.885479000
	C	-5.122952000	0.609257000	-2.277780000
	H	-5.881135000	0.211200000	-2.979819000
	H	-4.117331000	0.333699000	-2.658978000
	H	-5.187836000	1.718417000	-2.301055000
	C	-6.418420000	-0.817433000	-0.633717000
	H	-7.026543000	-1.160524000	-1.487321000
	C	-6.739634000	-1.258718000	0.664060000

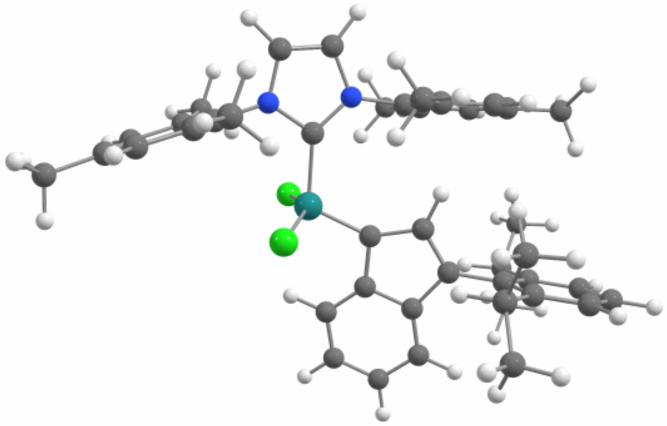

Zero-point correction= 0.745683 (Hartree/Particle)
Thermal correction to Energy= 0.796429
Thermal correction to Enthalpy= 0.797373
Thermal correction to Gibbs Free Energy= 0.657321
Sum of electronic and zero-point Energies= -2750.999927
Sum of electronic and thermal Energies= -2750.949181
Sum of electronic and thermal Enthalpies= -2750.948236
Sum of electronic and thermal Free Energies= -2751.088289

Solvent: -2752.22678036

C	-7.853603000	-2.254104000	0.895793000
H	-8.378557000	-2.066839000	1.854819000
H	-7.451539000	-3.290206000	0.942864000
H	-8.602699000	-2.230154000	0.078881000
C	-5.995190000	-0.742518000	1.743921000
H	-6.264191000	-1.030421000	2.773844000
C	-4.919899000	0.145059000	1.555264000
C	-4.198137000	0.742848000	2.738892000
H	-4.654489000	0.402933000	3.688683000
H	-4.235106000	1.853204000	2.719103000
H	-3.131557000	0.438517000	2.744535000
C	-0.129360000	2.741997000	-0.226339000
C	0.517736000	3.032797000	1.001627000
C	-0.178878000	2.807246000	2.323603000
H	0.474075000	3.095942000	3.169682000
H	-0.456578000	1.739136000	2.450705000
H	-1.119466000	3.392794000	2.401812000
C	1.838076000	3.518742000	0.949551000
H	2.354046000	3.751331000	1.896072000
C	2.512409000	3.719447000	-0.273784000
C	3.938946000	4.220006000	-0.291588000
H	4.222136000	4.616900000	-1.286722000
H	4.647919000	3.399520000	-0.046614000
H	4.098830000	5.021083000	0.459374000
C	1.826975000	3.421501000	-1.468113000
H	2.331181000	3.585241000	-2.434889000
C	0.501531000	2.937884000	-1.476546000
C	-0.222157000	2.666462000	-2.772976000
H	-1.120048000	3.313210000	-2.877515000
H	-0.581723000	1.616929000	-2.830011000
H	0.436988000	2.858380000	-3.641573000
H	1.556936000	0.908637000	0.402491000
C	1.543416000	-0.133588000	0.070911000
C	2.649046000	-0.971679000	0.056018000
C	0.361334000	-0.857641000	-0.370144000
C	2.222125000	-2.303093000	-0.427090000
C	0.826040000	-2.240683000	-0.724523000
C	2.942578000	-3.489920000	-0.606033000
C	0.167961000	-3.366724000	-1.240142000
C	2.266344000	-4.627131000	-1.105945000
H	4.015527000	-3.534781000	-0.359208000
C	0.899909000	-4.563803000	-1.424503000
H	-0.898003000	-3.331547000	-1.516019000
H	2.818838000	-5.568875000	-1.252531000
H	0.388316000	-5.453054000	-1.824873000
C	4.045580000	-0.640774000	0.462403000
C	5.032348000	-0.399069000	-0.541222000
C	4.395378000	-0.590798000	1.845218000
C	6.345478000	-0.078711000	-0.138527000
C	5.722425000	-0.266277000	2.197230000
C	6.691439000	-0.006720000	1.218461000
H	7.113845000	0.119249000	-0.903702000
H	6.004166000	-0.223414000	3.261970000
H	7.722760000	0.245770000	1.513733000
C	4.695355000	-0.454184000	-2.034683000
H	3.632305000	-0.762396000	-2.125282000
C	3.381422000	-0.906166000	2.948477000
H	2.427431000	-1.183190000	2.453606000
C	3.093104000	0.327397000	3.829315000
H	4.002346000	0.666868000	4.370788000
H	2.727674000	1.178774000	3.218847000
H	2.318116000	0.093589000	4.589666000
C	3.820102000	-2.114894000	3.802116000
H	3.037990000	-2.367105000	4.548937000
H	3.990799000	-3.012440000	3.173023000
H	4.759292000	-1.909212000	4.359206000
C	4.819589000	0.934726000	-2.695840000
H	5.863423000	1.313902000	-2.655925000
H	4.521288000	0.887580000	-3.764739000
H	4.168005000	1.677029000	-2.191961000
C	5.543761000	-1.502590000	-2.784187000
H	5.430721000	-2.511727000	-2.338265000
H	5.233204000	-1.566100000	-3.848460000
H	6.624566000	-1.245356000	-2.768259000

Catalyst 6, II-III TS

Ru	-1.218375000	0.246447000	-0.293998000
Cl	-1.961757000	1.305912000	1.641314000
Cl	-1.092303000	0.262229000	-2.630497000
N	-3.345778000	-1.760697000	-0.329861000
N	-1.445834000	-2.850251000	-0.256289000
C	-1.970295000	-1.559772000	-0.210013000
C	-3.650550000	-3.116489000	-0.454287000
H	-4.683565000	-3.462753000	-0.557689000
C	-2.468995000	-3.794912000	-0.406202000
H	-2.246209000	-4.864709000	-0.460766000
C	-4.372588000	-0.742701000	-0.251143000
C	-4.884016000	-0.404673000	1.028101000

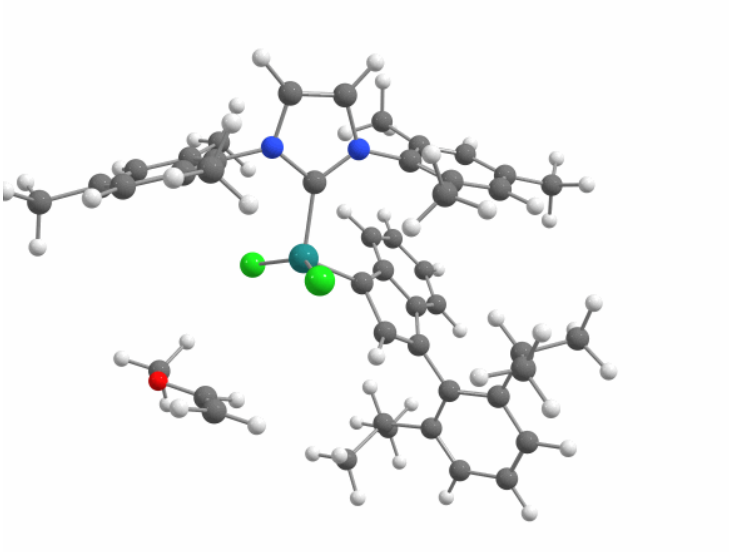

Zero-point correction= 0.827114 (Hartree/Particle)
Thermal correction to Energy= 0.884796
Thermal correction to Enthalpy= 0.885740
Thermal correction to Gibbs Free Energy= 0.726304
Sum of electronic and zero-point Energies= -2943.881162
Sum of electronic and thermal Energies= -2943.823480
Sum of electronic and thermal Enthalpies= -2943.822536
Sum of electronic and thermal Free Energies= -2943.981972

Solvent: -2945.27822670

C	-4.391249000	-1.092374000	2.278961000
H	-4.969812000	-0.761799000	3.163229000
H	-3.323804000	-0.852997000	2.464233000
H	-4.482835000	-2.197009000	2.202083000
C	-5.889883000	0.576770000	1.094275000
H	-6.285077000	0.859772000	2.084024000
C	-6.407308000	1.197050000	-0.060596000
C	-7.447175000	2.289416000	0.042766000
H	-8.140897000	2.279466000	-0.822497000
H	-6.962894000	3.290558000	0.059930000
H	-8.047182000	2.199014000	0.970779000
C	-5.925044000	0.774870000	-1.314552000
H	-6.350939000	1.212392000	-2.232783000
C	-4.920302000	-0.205741000	-1.444677000
C	-4.493120000	-0.687501000	-2.809923000
H	-5.099719000	-0.205412000	-3.600878000
H	-4.613969000	-1.787374000	-2.910480000
H	-3.423513000	-0.460403000	-3.006899000
C	-0.088749000	-3.304750000	-0.041011000
C	0.824986000	-3.348347000	-1.123489000
C	0.426046000	-2.935208000	-2.517181000
H	1.255330000	-3.106453000	-3.230542000
H	0.129012000	-1.867339000	-2.573157000
H	-0.451731000	-3.518773000	-2.870070000
C	2.112710000	-3.861739000	-0.869040000
H	2.833470000	-3.901549000	-1.702724000
C	2.495411000	-4.351950000	0.395650000
C	3.898711000	-4.855699000	0.644788000
H	4.361637000	-5.256196000	-0.279712000
H	3.917974000	-5.652304000	1.416107000
H	4.552170000	-4.032863000	1.008857000
C	1.532748000	-4.354780000	1.422312000
H	1.797446000	-4.766045000	2.410618000
C	0.232979000	-3.848542000	1.228009000
C	-0.791806000	-3.958539000	2.335591000
H	-1.379915000	-4.898193000	2.241545000
H	-1.521446000	-3.125578000	2.325562000
H	-0.299736000	-3.980547000	3.327750000
H	1.415029000	1.347163000	-1.520311000
C	1.598487000	0.847285000	-0.560650000
C	2.818234000	0.780501000	0.091505000
C	0.578857000	0.094615000	0.186755000
C	2.623317000	0.011243000	1.344082000
C	1.253440000	-0.399367000	1.410473000
C	3.500860000	-0.247569000	2.402570000
C	0.754652000	-0.978221000	2.580228000
C	2.997910000	-0.887522000	3.561626000
H	4.554877000	0.069273000	2.347288000
C	1.639118000	-1.228853000	3.656983000
H	-0.315852000	-1.210820000	2.664919000
H	3.674292000	-1.091932000	4.407078000
H	1.255241000	-1.690150000	4.580866000
C	4.108101000	1.402490000	-0.329432000
C	4.948271000	0.750853000	-1.279751000
C	4.492434000	2.655859000	0.235399000
C	6.154784000	1.375303000	-1.658319000
C	5.710792000	3.238145000	-0.170958000
C	6.537672000	2.607648000	-1.110865000
H	6.809003000	0.884970000	-2.396927000
H	6.013717000	4.209154000	0.252996000
H	7.484827000	3.079147000	-1.418949000
C	-1.600098000	4.524032000	-0.623375000
C	-1.495775000	4.313852000	-1.951439000
H	-0.528137000	4.024535000	-2.382140000
H	-2.366413000	4.412657000	-2.617323000
H	-0.730049000	4.412034000	0.052871000
O	-2.767344000	4.883196000	-0.022352000
C	-2.703269000	5.064232000	1.388293000
H	-3.722051000	5.338018000	1.724088000
H	-2.392202000	4.128504000	1.903328000
H	-2.005245000	5.888528000	1.665070000
C	3.589809000	3.400363000	1.223875000
H	2.758976000	2.715887000	1.496869000
C	4.554189000	-0.587608000	-1.909099000
H	3.701417000	-0.989692000	-1.320752000
C	4.319063000	3.774437000	2.529965000
H	5.144550000	4.496730000	2.353821000
H	3.614916000	4.248013000	3.246079000
H	4.753347000	2.880308000	3.022635000
C	2.953364000	4.641493000	0.561007000
H	2.383827000	4.364889000	-0.349795000
H	2.255891000	5.148149000	1.261419000
H	3.726426000	5.381257000	0.261370000
C	5.688823000	-1.629915000	-1.843670000
H	5.335994000	-2.613008000	-2.220894000
H	6.560683000	-1.337831000	-2.467051000
H	6.051180000	-1.772403000	-0.804662000
C	4.057641000	-0.392213000	-3.358250000
H	3.741899000	-1.359817000	-3.803305000
H	3.191025000	0.298442000	-3.399939000
H	4.857479000	0.031252000	-4.003018000

Catalyst 6, III intermediate

Ru	-1.494819000	-0.535003000	-0.037463000
Cl	-2.300789000	-0.905577000	-2.258696000

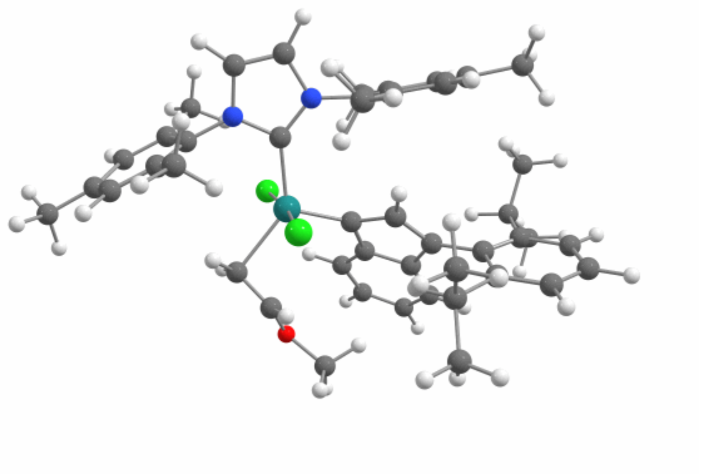

Zero-point correction= 0.829533 (Hartree/Particle)
Thermal correction to Energy= 0.886189
Thermal correction to Enthalpy= 0.887134
Thermal correction to Gibbs Free Energy= 0.735825
Sum of electronic and zero-point Energies= -2943.893288
Sum of electronic and thermal Energies= -2943.836632
Sum of electronic and thermal Enthalpies= -2943.835688
Sum of electronic and thermal Free Energies= -2943.986996

Solvent: -2945.29470157

Cl	-0.973964000	-0.219454000	2.337037000
N	-3.356365000	1.773079000	-0.079253000
N	-1.363293000	2.640712000	-0.223652000
C	-2.009577000	1.423809000	-0.154783000
C	-3.521748000	3.158294000	-0.101099000
H	-4.514042000	3.616231000	-0.044827000
C	-2.276474000	3.703572000	-0.195216000
H	-1.939235000	4.742950000	-0.250108000
C	-4.492856000	0.883952000	0.060593000
C	-5.264853000	0.567189000	-1.089170000
C	-4.955571000	1.159720000	-2.441439000
H	-5.684865000	0.810905000	-3.198077000
H	-3.938261000	0.860956000	-2.771933000
H	-4.992286000	2.269980000	-2.417889000
C	-6.366361000	-0.293039000	-0.918943000
H	-6.962161000	-0.562178000	-1.807058000
C	-6.736237000	-0.802433000	0.342350000
C	-7.893965000	-1.765016000	0.480492000
H	-8.313415000	-1.758511000	1.506641000
H	-7.569634000	-2.806812000	0.263883000
H	-8.711734000	-1.527684000	-0.230543000
C	-6.004744000	-0.378675000	1.468511000
H	-6.320672000	-0.705904000	2.473305000
C	-4.894621000	0.485848000	1.363553000
C	-4.219521000	1.014138000	2.605601000
H	-4.770524000	0.697020000	3.512402000
H	-4.183839000	2.124631000	2.598910000
H	-3.167548000	0.661726000	2.695519000
C	0.046126000	2.949425000	-0.292541000
C	0.685477000	3.411691000	0.886377000
C	-0.010942000	3.380232000	2.226333000
H	0.659947000	3.750030000	3.025954000
H	-0.332027000	2.345879000	2.481773000
H	-0.926150000	4.010360000	2.234026000
C	1.999058000	3.902405000	0.764445000
H	2.509269000	4.270845000	1.670262000
C	2.665518000	3.962316000	-0.476884000
C	4.060284000	4.541348000	-0.563000000
H	4.375550000	4.703041000	-1.612722000
H	4.804993000	3.866535000	-0.088571000
H	4.123340000	5.514321000	-0.031915000
C	1.996040000	3.473388000	-1.616314000
H	2.497491000	3.512317000	-2.597329000
C	0.683236000	2.960380000	-1.554201000
C	-0.030256000	2.476475000	-2.794190000
H	-0.921544000	3.101916000	-3.019027000
H	-0.399463000	1.435685000	-2.674916000
H	0.638726000	2.512397000	-3.675591000
H	1.406116000	0.792217000	0.871920000
C	1.484776000	-0.083996000	0.220381000
C	2.655684000	-0.769729000	-0.056994000
C	0.336215000	-0.713903000	-0.441690000
C	2.311923000	-1.901975000	-0.951541000
C	0.912648000	-1.838537000	-1.252376000
C	3.105494000	-2.946694000	-1.438672000
C	0.347975000	-2.790029000	-2.111897000
C	2.523248000	-3.905783000	-2.302227000
H	4.167394000	-3.017068000	-1.155053000
C	1.167472000	-3.812868000	-2.649317000
H	-0.719849000	-2.731456000	-2.364809000
H	3.141759000	-4.724001000	-2.705091000
H	0.724058000	-4.552697000	-3.334686000
C	4.018164000	-0.515751000	0.488571000
C	5.112877000	-0.235041000	-0.394079000
C	4.245463000	-0.583857000	1.902400000
C	6.391755000	-0.008040000	0.154301000
C	5.546116000	-0.348580000	2.395253000
C	6.613567000	-0.059040000	1.536796000
H	7.233770000	0.221335000	-0.518724000
H	5.726219000	-0.402413000	3.481299000
H	7.621078000	0.123228000	1.944544000
C	-2.544932000	-2.471087000	0.449971000
C	-1.413747000	-3.117020000	0.929077000
H	-3.150310000	-1.916093000	1.191664000
H	-3.066730000	-2.867975000	-0.435396000
H	-0.935385000	-2.805545000	1.877465000
O	-0.892703000	-4.169790000	0.287846000
C	0.355362000	-4.675445000	0.784999000
H	0.318157000	-4.796074000	1.889282000
H	0.508094000	-5.661115000	0.309185000
H	1.188711000	-3.998512000	0.504100000
C	3.149541000	-0.948779000	2.909708000
H	2.212807000	-1.133304000	2.347937000
C	4.942629000	-0.100501000	-1.913062000
H	3.910080000	-0.412574000	-2.169321000
C	5.910936000	-1.004119000	-2.704816000
H	5.828142000	-2.067814000	-2.403120000
H	5.693878000	-0.943525000	-3.792136000
H	6.969436000	-0.697884000	-2.563922000
C	5.092600000	1.369005000	-2.358955000
H	6.108751000	1.758404000	-2.135083000
H	4.928364000	1.465325000	-3.453566000
H	4.356930000	2.015956000	-1.843685000
C	2.858760000	0.208777000	3.886900000
H	2.579804000	1.133651000	3.342323000
H	2.014520000	-0.053164000	4.557998000
H	3.739897000	0.443385000	4.522707000
C	3.489534000	-2.249626000	3.669375000
H	3.670534000	-3.091911000	2.969554000
H	4.398289000	-2.138627000	4.299034000
H	2.653177000	-2.535367000	4.342064000

Catalyst 6, III-IV TS

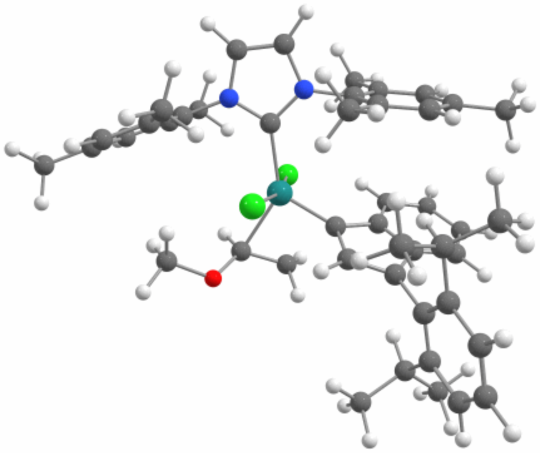

Zero-point correction= 0.830183 (Hartree/Particle)
Thermal correction to Energy= 0.885433
Thermal correction to Enthalpy= 0.886378
Thermal correction to Gibbs Free Energy= 0.738362
Sum of electronic and zero-point Energies= -2943.890690
Sum of electronic and thermal Energies= -2943.835440
Sum of electronic and thermal Enthalpies= -2943.834496
Sum of electronic and thermal Free Energies= -2943.982511

Solvent: -2945.29484701

Ru	-1.189537000	-0.368922000	-0.567891000
Cl	-1.863905000	0.497768000	-2.738351000
Cl	-1.038012000	-1.304775000	1.676434000
N	-3.646748000	1.098369000	0.670060000
N	-1.910346000	2.411265000	0.627132000
C	-2.311968000	1.132771000	0.318037000
C	-4.057182000	2.332674000	1.184183000
H	-5.086376000	2.499301000	1.516670000
C	-2.967061000	3.154253000	1.161706000
H	-2.832591000	4.193585000	1.476552000
C	-4.562435000	-0.024201000	0.588260000
C	-5.318395000	-0.214942000	-0.596547000
C	-5.219209000	0.729164000	-1.768747000
H	-5.970059000	0.473435000	-2.541388000
H	-4.210155000	0.702630000	-2.236168000
H	-5.395902000	1.780322000	-1.455030000
C	-6.222818000	-1.298262000	-0.627173000
H	-6.811038000	-1.462705000	-1.545533000
C	-6.420409000	-2.145227000	0.479820000
C	-7.379538000	-3.312909000	0.412187000
H	-6.832129000	-4.273833000	0.293643000
H	-8.076612000	-3.222833000	-0.444756000
H	-7.981983000	-3.400396000	1.340019000
C	-5.706799000	-1.866323000	1.663639000
H	-5.881919000	-2.484869000	2.559917000
C	-4.785209000	-0.804996000	1.754048000
C	-4.094619000	-0.496292000	3.059154000
H	-4.413305000	-1.203690000	3.849203000
H	-4.326151000	0.532398000	3.409634000
H	-2.991427000	-0.570505000	2.941095000
C	-0.592384000	2.986752000	0.461382000
C	0.357476000	2.821488000	1.498436000
C	0.043172000	2.016713000	2.735352000
H	0.859279000	2.104257000	3.478470000
H	-0.104395000	0.938667000	2.499188000
H	-0.895514000	2.362862000	3.218555000
C	1.605445000	3.458657000	1.343046000
H	2.358536000	3.340107000	2.139299000
C	1.909960000	4.250603000	0.218797000
C	3.270223000	4.890807000	0.060687000
H	3.199537000	5.894261000	-0.407274000
H	3.918333000	4.269868000	-0.595860000
H	3.790184000	4.997794000	1.033758000
C	0.917925000	4.415545000	-0.768338000
H	1.129125000	5.046422000	-1.647735000
C	-0.344594000	3.799107000	-0.672625000
C	-1.394789000	4.008496000	-1.737393000
H	-2.301406000	4.506399000	-1.330442000
H	-1.724625000	3.038985000	-2.171222000
H	-1.002958000	4.642785000	-2.556238000
H	1.337609000	-1.784999000	0.981136000
C	1.673118000	-1.069949000	0.221676000
C	2.974210000	-0.737137000	-0.062614000
C	0.728254000	-0.362186000	-0.686902000
C	2.964840000	0.244134000	-1.177685000
C	1.612397000	0.461504000	-1.586471000
C	4.027630000	0.896134000	-1.811080000
C	1.343561000	1.329024000	-2.651066000
C	3.744470000	1.768466000	-2.888848000
H	5.064078000	0.724600000	-1.478713000
C	2.420608000	1.974208000	-3.305733000
H	0.305604000	1.489634000	-2.976547000
H	4.570458000	2.283414000	-3.405792000
H	2.211055000	2.647979000	-4.151696000
C	4.216288000	-1.287610000	0.556967000
C	4.653178000	-0.844556000	1.840264000
C	4.970388000	-2.264613000	-0.162941000
C	5.833644000	-1.396208000	2.381980000
C	6.144210000	-2.783971000	0.419306000
C	6.576767000	-2.355668000	1.682120000
H	6.178999000	-1.066531000	3.375175000
H	6.727965000	-3.545313000	-0.122728000
H	7.496115000	-2.773388000	2.123415000
C	-0.153935000	-1.979911000	-1.854513000
C	-1.436871000	-2.377545000	-1.351176000
H	-0.103147000	-1.541967000	-2.863059000
H	0.695919000	-2.602128000	-1.534475000
H	-2.338570000	-2.177313000	-1.972033000
O	-1.498162000	-3.486567000	-0.569093000
C	-2.757516000	-3.761929000	0.045950000
H	-3.596134000	-3.609845000	-0.671146000
H	-2.732078000	-4.821215000	0.365080000
H	-2.896636000	-3.104291000	0.929491000
C	4.491078000	-2.812321000	-1.510709000
H	3.679682000	-2.145407000	-1.871074000
C	3.870167000	0.192254000	2.649112000
H	3.099577000	0.623746000	1.975610000
C	4.760131000	1.354155000	3.135859000
H	5.289690000	1.842814000	2.292072000
H	4.146633000	2.125177000	3.648863000
H	5.527154000	1.015634000	3.864669000
C	3.127191000	-0.475379000	3.826931000
H	2.411806000	-1.243835000	3.470413000

H	3.838232000	-0.970167000	4.523085000
H	2.552179000	0.275452000	4.409425000
C	5.591003000	-2.806073000	-2.590452000
H	6.422749000	-3.500145000	-2.344217000
H	5.175937000	-3.128016000	-3.568733000
H	6.024393000	-1.793317000	-2.722245000
C	3.878976000	-4.220414000	-1.338683000
H	3.477879000	-4.598730000	-2.303176000
H	4.639146000	-4.947157000	-0.980001000
H	3.050961000	-4.212893000	-0.600222000

Catalyst 6, IV intermediate

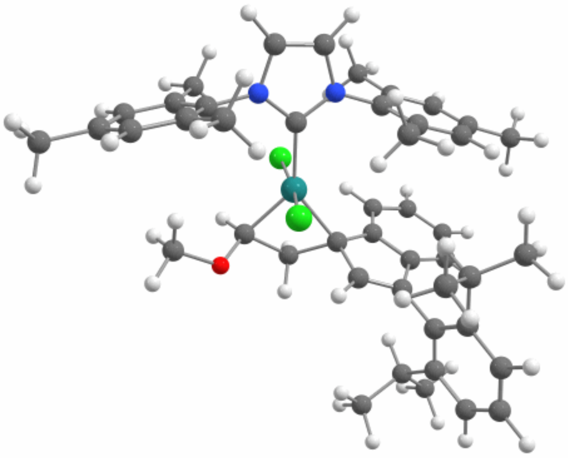

Zero-point correction= 0.831293 (Hartree/Particle)
Thermal correction to Energy= 0.886854
Thermal correction to Enthalpy= 0.887798
Thermal correction to Gibbs Free Energy= 0.738685
Sum of electronic and zero-point Energies= -2943.899905
Sum of electronic and thermal Energies= -2943.844344
Sum of electronic and thermal Enthalpies= -2943.843400
Sum of electronic and thermal Free Energies= -2943.992512

Solvent: -2945.30522242

Ru	-1.173835000	-0.247147000	-0.652484000
Cl	-1.842563000	0.804843000	-2.735840000
Cl	-0.892570000	-1.309789000	1.519234000
N	-3.654393000	0.958098000	0.762480000
N	-1.973021000	2.347250000	0.756136000
C	-2.338731000	1.079630000	0.372683000
C	-4.095172000	2.139616000	1.368951000
H	-5.120324000	2.241452000	1.737839000
C	-3.039049000	3.005394000	1.373038000
H	-2.934795000	4.025180000	1.755887000
C	-4.534894000	-0.188100000	0.633475000
C	-5.305129000	-0.331769000	-0.547758000
C	-5.232043000	0.669913000	-1.673376000
H	-5.959428000	0.418854000	-2.469552000
H	-4.217615000	0.716795000	-2.127004000
H	-5.463714000	1.695507000	-1.313124000
C	-6.196068000	-1.424155000	-0.617788000
H	-6.797545000	-1.551487000	-1.533368000
C	-6.362596000	-2.327912000	0.448407000
C	-7.315754000	-3.497851000	0.345170000
H	-7.956806000	-3.580442000	1.247642000
H	-6.763288000	-4.458770000	0.256911000
H	-7.977154000	-3.411786000	-0.539741000
C	-5.623373000	-2.105529000	1.628687000
H	-5.765582000	-2.777656000	2.491459000
C	-4.713832000	-1.037573000	1.757823000
C	-3.983089000	-0.806321000	3.057171000
H	-4.295447000	-1.545790000	3.819791000
H	-4.180872000	0.208074000	3.464535000
H	-2.884811000	-0.894234000	2.904282000
C	-0.670932000	2.963362000	0.595392000
C	0.301419000	2.759485000	1.605841000
C	0.009417000	1.918296000	2.823670000
H	0.844689000	1.975859000	3.547885000
H	-0.153522000	0.849309000	2.557474000
H	-0.912063000	2.260750000	3.341852000
C	1.545863000	3.401849000	1.448319000
H	2.317316000	3.247543000	2.220055000
C	1.823712000	4.241473000	0.352210000
C	3.182836000	4.880089000	0.185367000
H	3.110654000	5.885813000	-0.277008000
H	3.819743000	4.257683000	-0.480837000
H	3.713335000	4.979078000	1.153688000
C	0.803617000	4.459387000	-0.595217000
H	0.989455000	5.138186000	-1.443901000
C	-0.458861000	3.843671000	-0.495686000
C	-1.540602000	4.132031000	-1.507681000
H	-2.463433000	4.520945000	-1.026375000
H	-1.822989000	3.208484000	-2.059102000
H	-1.198399000	4.886951000	-2.241971000
H	1.380116000	-2.172188000	0.528037000
C	1.714481000	-1.322529000	-0.078218000
C	2.986029000	-0.817044000	-0.125504000
C	0.816388000	-0.633582000	-1.060185000
C	3.004590000	0.282909000	-1.118298000
C	1.709303000	0.396202000	-1.709294000
C	4.062566000	1.103413000	-1.534589000
C	1.489016000	1.331374000	-2.729033000
C	3.831353000	2.040375000	-2.565241000
H	5.058660000	1.001146000	-1.074660000
C	2.560937000	2.147120000	-3.157445000
H	0.489012000	1.434367000	-3.177301000
H	4.654938000	2.685284000	-2.912671000
H	2.392761000	2.877368000	-3.965026000
C	4.195939000	-1.330914000	0.586863000
C	4.437045000	-1.007439000	1.954160000
C	5.116533000	-2.158830000	-0.127444000
C	5.589885000	-1.525208000	2.582777000
C	6.256115000	-2.648519000	0.542143000
C	6.495344000	-2.337005000	1.888000000
H	5.781951000	-1.284772000	3.641038000
H	6.965966000	-3.294783000	0.001483000
H	7.390134000	-2.730480000	2.397012000
C	0.000845000	-1.637706000	-1.995429000
C	-1.487448000	-1.977477000	-1.589952000
H	0.020239000	-1.274499000	-3.039134000
H	0.512015000	-2.611783000	-1.873409000
H	-2.206567000	-1.853068000	-2.433743000
O	-1.581095000	-3.199942000	-0.979170000
C	-2.907522000	-3.648749000	-0.717280000
H	-3.550859000	-3.552867000	-1.622692000

H	-2.835054000	-4.714624000	-0.429161000
H	-3.361285000	-3.070202000	0.115397000
C	3.475610000	-0.133523000	2.762047000
H	2.731722000	0.284953000	2.051431000
C	4.852131000	-2.586407000	-1.574731000
H	4.051793000	-1.929484000	-1.974118000
C	4.316907000	-4.035145000	-1.622238000
H	3.401761000	-4.146491000	-1.004851000
H	4.069075000	-4.332910000	-2.663679000
H	5.070319000	-4.754057000	-1.234459000
C	6.078534000	-2.413153000	-2.492124000
H	6.461950000	-1.372466000	-2.465243000
H	6.913132000	-3.088828000	-2.207396000
H	5.811873000	-2.650179000	-3.543728000
C	4.194009000	1.053904000	3.435584000
H	4.933209000	0.719521000	4.194796000
H	4.734657000	1.676875000	2.693296000
H	3.463066000	1.703508000	3.962874000
C	2.696230000	-0.975862000	3.795183000
H	1.984446000	-0.341967000	4.364875000
H	2.107944000	-1.775232000	3.301558000
H	3.382937000	-1.454994000	4.526061000

Catalyst 6, IV-V TS

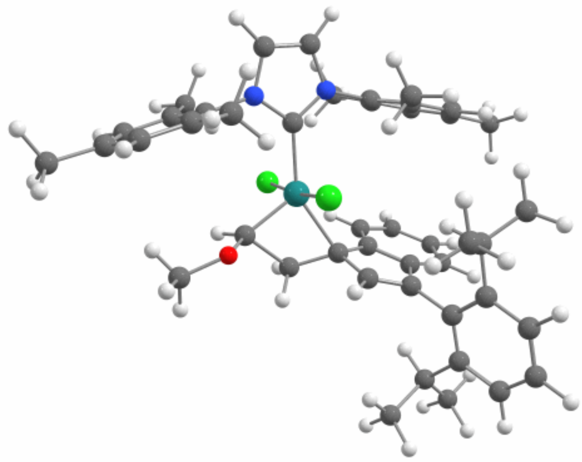

Zero-point correction= 0.830336 (Hartree/Particle)
Thermal correction to Energy= 0.885507
Thermal correction to Enthalpy= 0.886451
Thermal correction to Gibbs Free Energy= 0.737923
Sum of electronic and zero-point Energies= -2943.897901
Sum of electronic and thermal Energies= -2943.842731
Sum of electronic and thermal Enthalpies= -2943.841787
Sum of electronic and thermal Free Energies= -2943.990315

Solvent: -2945.29922466

Ru	-1.145651000	-0.283610000	-0.521475000
Cl	-1.961216000	0.427731000	-2.698660000
Cl	-0.528491000	-0.848669000	1.758222000
N	-3.681788000	0.952857000	0.747135000
N	-2.074961000	2.423833000	0.688840000
C	-2.366532000	1.118495000	0.372369000
C	-4.192505000	2.136766000	1.288450000
H	-5.225399000	2.202350000	1.643864000
C	-3.183844000	3.057733000	1.255719000
H	-3.141436000	4.102581000	1.578222000
C	-4.500948000	-0.237343000	0.614532000
C	-5.300575000	-0.387629000	-0.547418000
C	-5.316495000	0.649542000	-1.642866000
H	-6.077946000	0.400722000	-2.407384000
H	-4.325294000	0.719036000	-2.143826000
H	-5.549959000	1.659970000	-1.244595000
C	-6.109862000	-1.539031000	-0.638957000
H	-6.729672000	-1.673407000	-1.541420000
C	-6.167468000	-2.500200000	0.389864000
C	-7.080271000	-3.702306000	0.280565000
H	-8.088116000	-3.475286000	0.693538000
H	-6.686522000	-4.569864000	0.848124000
H	-7.225287000	-4.014058000	-0.773756000
C	-5.398771000	-2.278550000	1.549462000
H	-5.452476000	-3.001047000	2.381039000
C	-4.568872000	-1.148396000	1.699129000
C	-3.805170000	-0.920409000	2.978218000
H	-4.064185000	-1.687815000	3.733225000
H	-4.028636000	0.077073000	3.413436000
H	-2.707094000	-0.955507000	2.796395000
C	-0.818675000	3.113683000	0.466660000
C	0.130568000	3.158427000	1.518564000
C	-0.133177000	2.505111000	2.852812000
H	0.681303000	2.731950000	3.567713000
H	-0.216250000	1.399854000	2.747023000
H	-1.086099000	2.859557000	3.301002000
C	1.329122000	3.861868000	1.282675000
H	2.083488000	3.897679000	2.085575000
C	1.583917000	4.524702000	0.066517000
C	2.898625000	5.229328000	-0.177782000
H	2.764588000	6.146555000	-0.787223000
H	3.596522000	4.566756000	-0.735368000
H	3.397660000	5.511401000	0.771058000
C	0.583270000	4.505335000	-0.924914000
H	0.747301000	5.050107000	-1.869400000
C	-0.634178000	3.819193000	-0.750209000
C	-1.703154000	3.866783000	-1.814510000
H	-2.679323000	4.197968000	-1.400258000
H	-1.868064000	2.864223000	-2.267939000
H	-1.419463000	4.570330000	-2.621234000
H	1.516820000	-2.276260000	0.641562000
C	1.788983000	-1.467708000	-0.046731000
C	3.030324000	-0.899983000	-0.163621000
C	0.845804000	-0.914856000	-1.061118000
C	2.968836000	0.110782000	-1.244993000
C	1.659546000	0.098145000	-1.819144000
C	3.965649000	0.960463000	-1.745736000
C	1.367781000	0.926840000	-2.910850000
C	3.660377000	1.795261000	-2.842629000
H	4.971801000	0.960794000	-1.296600000
C	2.379521000	1.771013000	-3.421583000
H	0.357395000	0.930457000	-3.346688000
H	4.435188000	2.463511000	-3.252431000
H	2.155266000	2.420388000	-4.282792000
C	4.270292000	-1.269291000	0.584124000
C	4.474007000	-0.827782000	1.924480000
C	5.255639000	-2.077589000	-0.063681000
C	5.658914000	-1.206647000	2.591691000
C	6.422885000	-2.428866000	0.643853000

C	6.627838000	-1.998056000	1.962556000
H	5.824457000	-0.871735000	3.628572000
H	7.184094000	-3.059220000	0.156877000
H	7.546085000	-2.283065000	2.501347000
C	-0.112817000	-1.884245000	-1.705646000
C	-1.741932000	-2.031944000	-0.999438000
H	-0.269470000	-1.717092000	-2.786702000
H	0.176257000	-2.920462000	-1.452949000
H	-2.407113000	-2.121916000	-1.891695000
O	-1.873132000	-3.061477000	-0.124900000
C	-2.647099000	-4.178232000	-0.579827000
H	-2.140067000	-4.704917000	-1.419778000
H	-2.737097000	-4.869988000	0.278480000
H	-3.662722000	-3.858609000	-0.899138000
C	5.028944000	-2.633879000	-1.472878000
H	4.209066000	-2.044467000	-1.933703000
C	3.453456000	0.043233000	2.660507000
H	2.625835000	0.261204000	1.954385000
C	4.065845000	1.390492000	3.098193000
H	3.299328000	2.023481000	3.593781000
H	4.896244000	1.252149000	3.823707000
H	4.467862000	1.954634000	2.231375000
C	2.832524000	-0.704886000	3.859195000
H	3.598989000	-0.965504000	4.620898000
H	2.062634000	-0.076589000	4.353999000
H	2.338492000	-1.642753000	3.535002000
C	6.258326000	-2.491645000	-2.391044000
H	7.112244000	-3.114069000	-2.048063000
H	6.010285000	-2.820606000	-3.422258000
H	6.606650000	-1.439677000	-2.444097000
C	4.550157000	-4.101467000	-1.403720000
H	4.331417000	-4.496036000	-2.419185000
H	5.324304000	-4.753480000	-0.944984000
H	3.629244000	-4.195068000	-0.792039000

Catalyst 6, V intermediate

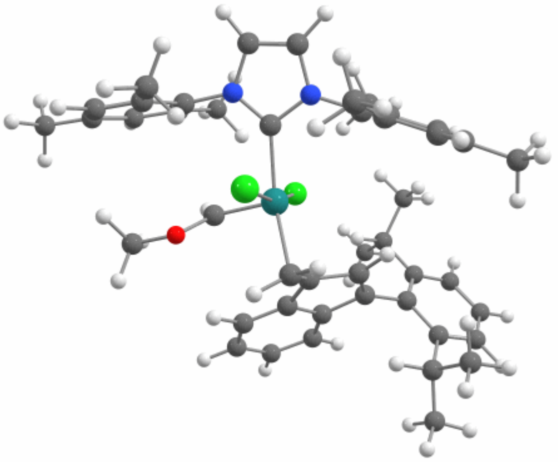

Zero-point correction= 0.829705 (Hartree/Particle)
Thermal correction to Energy= 0.886106
Thermal correction to Enthalpy= 0.887050
Thermal correction to Gibbs Free Energy= 0.734195
Sum of electronic and zero-point Energies= -2943.915636
Sum of electronic and thermal Energies= -2943.859235
Sum of electronic and thermal Enthalpies= -2943.858291
Sum of electronic and thermal Free Energies= -2944.011146

Solvent: -2945.31683176

Ru	1.010155000	-0.002551000	-0.749677000
Cl	0.179884000	-0.527323000	1.474556000
Cl	2.257269000	0.805377000	-2.704585000
N	3.603853000	1.232887000	0.666902000
N	1.969088000	2.648407000	0.430617000
C	2.308607000	1.329089000	0.209890000
C	4.055691000	2.463096000	1.152943000
H	5.063078000	2.581600000	1.563524000
C	3.028709000	3.351980000	1.001977000
H	2.945529000	4.414214000	1.251990000
C	4.458846000	0.064611000	0.717644000
C	4.388722000	-0.779261000	1.857388000
C	3.421359000	-0.507953000	2.982315000
H	3.574736000	-1.222329000	3.814339000
H	2.363256000	-0.582850000	2.642567000
H	3.550480000	0.519488000	3.385604000
C	5.280616000	-1.868540000	1.919768000
H	5.234529000	-2.532695000	2.799250000
C	6.240029000	-2.112839000	0.915252000
C	7.185088000	-3.289485000	1.022984000
H	7.930529000	-3.293607000	0.203191000
H	6.635651000	-4.255047000	0.985766000
H	7.737713000	-3.276260000	1.986272000
C	6.301981000	-1.222498000	-0.174104000
H	7.064405000	-1.376429000	-0.956033000
C	5.432904000	-0.116218000	-0.295186000
C	5.572914000	0.849873000	-1.445298000
H	6.350391000	0.506980000	-2.155336000
H	5.864722000	1.861723000	-1.089186000
H	4.611924000	0.962833000	-1.993843000
C	0.672680000	3.258804000	0.193790000
C	0.430344000	3.933112000	-1.031837000
C	1.501890000	4.106718000	-2.078646000
H	1.116176000	4.686899000	-2.939324000
H	1.876184000	3.127268000	-2.449341000
H	2.377551000	4.653944000	-1.666726000
C	-0.845055000	4.508440000	-1.215769000
H	-1.053446000	5.022056000	-2.169130000
C	-1.837111000	4.477997000	-0.217143000
C	-3.207945000	5.069196000	-0.455513000
H	-3.582522000	5.606338000	0.440117000
H	-3.947829000	4.270916000	-0.683398000
H	-3.208739000	5.775378000	-1.309774000
C	-1.511318000	3.892057000	1.022974000
H	-2.248896000	3.914905000	1.842249000
C	-0.260866000	3.291081000	1.264993000
C	0.074733000	2.744270000	2.629929000
H	1.042224000	3.140596000	3.004532000
H	0.160198000	1.635661000	2.597366000
H	-0.711041000	3.012737000	3.361664000
H	-2.296744000	0.850402000	-1.031342000
C	-2.172834000	-0.229612000	-0.867662000
C	-3.058091000	-1.048183000	-0.202166000
C	-1.040809000	-1.014407000	-1.399413000
C	-2.523915000	-2.426728000	-0.244737000
C	-1.291128000	-2.415849000	-0.962540000
C	-3.063784000	-3.635351000	0.228906000
C	-0.610226000	-3.614195000	-1.213066000
C	-2.359113000	-4.832029000	-0.004533000

H	-4.028321000	-3.643805000	0.761210000
C	-1.145389000	-4.822771000	-0.721290000
H	0.325374000	-3.618681000	-1.794637000
H	-2.766143000	-5.786780000	0.365539000
H	-0.616129000	-5.770712000	-0.909843000
C	-4.393347000	-0.684812000	0.365437000
C	-4.560087000	-0.510775000	1.772110000
C	-5.513938000	-0.548853000	-0.511055000
C	-5.838107000	-0.181621000	2.271132000
C	-6.769467000	-0.209027000	0.035041000
C	-6.935800000	-0.024739000	1.414414000
H	-5.973848000	-0.041187000	3.356029000
H	-7.637333000	-0.093687000	-0.634639000
H	-7.925413000	0.237163000	1.823048000
C	-0.214427000	-0.608222000	-2.472041000
C	2.020540000	-1.530519000	-0.630872000
H	0.330271000	-1.356829000	-3.068899000
H	-0.447890000	0.325126000	-3.010728000
H	2.085400000	-2.081991000	0.341834000
O	2.650680000	-2.137087000	-1.629798000
C	3.374989000	-3.355189000	-1.365988000
H	3.083492000	-3.782599000	-0.383595000
H	3.134661000	-4.067481000	-2.178864000
H	4.460206000	-3.130884000	-1.371624000
C	-5.393723000	-0.764669000	-2.022982000
H	-4.375560000	-1.159331000	-2.219531000
C	-3.390407000	-0.658313000	2.746818000
H	-2.474714000	-0.848432000	2.148914000
C	-3.584438000	-1.854526000	3.702667000
H	-3.710372000	-2.804281000	3.144096000
H	-2.700758000	-1.969592000	4.365579000
H	-4.477267000	-1.722307000	4.351881000
C	-3.141408000	0.642754000	3.537193000
H	-3.995804000	0.895870000	4.201780000
H	-2.238745000	0.540843000	4.175429000
H	-2.978333000	1.499885000	2.852526000
C	-5.521121000	0.566176000	-2.794837000
H	-5.387300000	0.405839000	-3.886114000
H	-6.519189000	1.031197000	-2.643187000
H	-4.757151000	1.298884000	-2.462201000
C	-6.397345000	-1.813294000	-2.545945000
H	-6.290200000	-2.777160000	-2.007251000
H	-7.450012000	-1.477111000	-2.430500000
H	-6.230119000	-2.006390000	-3.626898000

Catalyst 6, V-VI TS

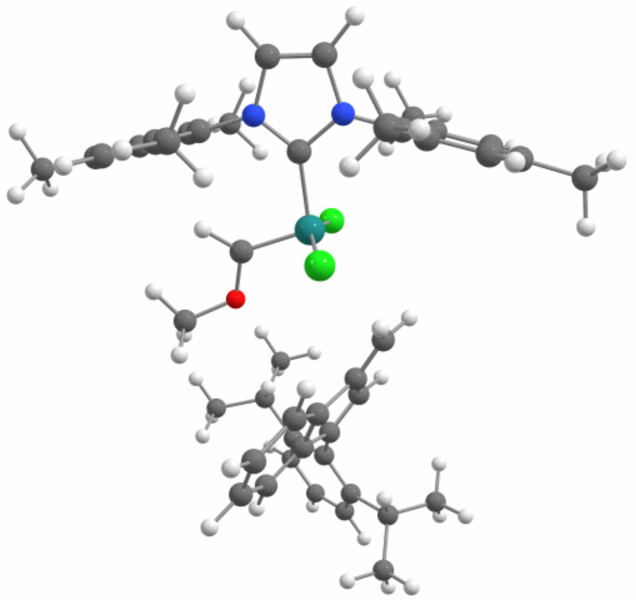

Zero-point correction= 0.828328 (Hartree/Particle)
Thermal correction to Energy= 0.885305
Thermal correction to Enthalpy= 0.886249
Thermal correction to Gibbs Free Energy= 0.727384
Sum of electronic and zero-point Energies= -2943.911209
Sum of electronic and thermal Energies= -2943.854232
Sum of electronic and thermal Enthalpies= -2943.853288

Ru	-1.668057000	0.254565000	0.032122000
Cl	-1.018724000	0.226887000	-2.230485000
Cl	-1.735267000	1.079685000	2.252138000
N	-4.427309000	-1.046282000	-0.323950000
N	-4.430061000	1.135846000	-0.471400000
C	-3.578449000	0.052560000	-0.239000000
C	-5.742693000	-0.651311000	-0.599490000
H	-6.547570000	-1.385675000	-0.702014000
C	-5.741210000	0.709633000	-0.693637000
H	-6.546697000	1.421843000	-0.898236000
C	-4.092321000	-2.435412000	-0.143104000
C	-3.702923000	-3.200571000	-1.269527000
C	-3.581788000	-2.571091000	-2.635680000
H	-3.330280000	-3.329774000	-3.401893000
H	-2.791556000	-1.787755000	-2.645175000
H	-4.526231000	-2.073042000	-2.941406000
C	-3.408452000	-4.563451000	-1.061856000
H	-3.099111000	-5.172613000	-1.927684000
C	-3.500228000	-5.166126000	0.210000000
C	-3.222230000	-6.642240000	0.389695000
H	-2.889200000	-6.875518000	1.421144000
H	-2.446220000	-7.002739000	-0.315704000
H	-4.138461000	-7.243730000	0.198810000
C	-3.893083000	-4.364824000	1.302728000
H	-3.966954000	-4.816402000	2.306315000
C	-4.198074000	-2.996578000	1.153560000
C	-4.579018000	-2.143674000	2.341109000
H	-4.651461000	-2.753816000	3.262146000
H	-5.554873000	-1.636465000	2.187014000
H	-3.829517000	-1.340589000	2.513761000
C	-4.060222000	2.535315000	-0.469726000
C	-4.232197000	3.283974000	0.725314000
C	-4.799528000	2.658487000	1.975974000
H	-4.980765000	3.427591000	2.751774000
H	-4.091116000	1.909733000	2.390272000
H	-5.759748000	2.137497000	1.776301000
C	-3.881001000	4.646843000	0.700675000
H	-3.998255000	5.236223000	1.625361000
C	-3.400451000	5.277325000	-0.465037000
C	-2.984458000	6.730874000	-0.442767000
H	-3.026489000	7.186546000	-1.452550000
H	-1.938966000	6.836492000	-0.077805000
H	-3.625239000	7.330267000	0.236042000
C	-3.324547000	4.517341000	-1.647698000
H	-3.007616000	5.005618000	-2.584407000
C	-3.667345000	3.149666000	-1.687630000
C	-3.659330000	2.398497000	-2.996437000
H	-4.608279000	1.843983000	-3.154726000
H	-2.836408000	1.651087000	-3.027254000

Sum of electronic and thermal Free Energies= -2944.012153<br><br>Solvent: -2945.29935828	H	-3.523937000	3.096851000	-3.845184000
	H	3.481278000	1.604711000	-1.530720000
	C	3.463244000	1.160706000	-0.525590000
	C	4.459327000	0.391056000	0.031775000
	C	2.353026000	1.330127000	0.425613000
	C	4.028880000	0.001529000	1.397007000
	C	2.741712000	0.568068000	1.640669000
	C	4.663806000	-0.779360000	2.375838000
	C	2.084303000	0.346744000	2.857293000
	C	3.999937000	-0.994171000	3.602511000
	H	5.659222000	-1.213337000	2.188554000
	C	2.725762000	-0.440883000	3.839228000
	H	1.080213000	0.762490000	3.041243000
	H	4.484240000	-1.601406000	4.384489000
	H	2.223498000	-0.620478000	4.803465000
	C	5.762539000	-0.003419000	-0.583267000
	C	5.848749000	-1.187911000	-1.372416000
	C	6.920002000	0.799856000	-0.359308000
	C	7.090584000	-1.533827000	-1.945430000
	C	8.140150000	0.414279000	-0.952291000
	C	8.228656000	-0.741450000	-1.741065000
	H	7.168521000	-2.442401000	-2.564498000
	H	9.039301000	1.031786000	-0.794431000
	H	9.190396000	-1.026950000	-2.197387000
	C	1.222271000	2.064186000	0.231861000
	C	-1.188614000	-1.461941000	0.447118000
	H	0.459374000	2.165100000	1.021678000
	H	1.047817000	2.592767000	-0.718243000
	H	-1.845450000	-2.333939000	0.671089000
	O	0.115318000	-1.731176000	0.555606000
	C	0.552943000	-2.985172000	1.110302000
	H	1.340014000	-3.391623000	0.446703000
	H	0.980848000	-2.796300000	2.115455000
	H	-0.294892000	-3.699680000	1.180394000
	C	6.850435000	2.081511000	0.474818000
	H	5.856230000	2.097263000	0.968801000
	C	4.624336000	-2.071164000	-1.625701000
	H	3.805339000	-1.687630000	-0.981424000
	C	4.142196000	-1.954562000	-3.087784000
	H	4.914750000	-2.317923000	-3.799315000
	H	3.222453000	-2.555757000	-3.250171000
	H	3.909773000	-0.902803000	-3.351954000
	C	4.869931000	-3.542327000	-1.231889000
	H	5.653172000	-4.016451000	-1.861351000
	H	5.190421000	-3.630907000	-0.173268000
	H	3.942315000	-4.140003000	-1.361437000
	C	6.930088000	3.333124000	-0.425888000
	H	6.125017000	3.330685000	-1.189174000
	H	6.828358000	4.261232000	0.176134000
	H	7.901897000	3.382662000	-0.962691000
	C	7.914124000	2.124335000	1.590739000
	H	7.845725000	1.234589000	2.249976000
	H	8.946637000	2.163881000	1.182186000
	H	7.776820000	3.027278000	2.222461000
